# The Lineage-Specific Evolution of Aquaporin Gene Clusters Facilitated Tetrapod Terrestrial Adaptation

**DOI:** 10.1371/journal.pone.0113686

**Published:** 2014-11-26

**Authors:** Roderick Nigel Finn, François Chauvigné, Jón Baldur Hlidberg, Christopher P. Cutler, Joan Cerdà

**Affiliations:** 1 Department of Biology, University of Bergen, Bergen, Norway; 2 Institute of Marine Research, Bergen, Norway; 3 Institut de Recerca i Tecnologia Agroalimentàries (IRTA)-Institut de Ciències del Mar, CSIC, Barcelona, Spain; 4 www.fauna.is, Reykjavik, Iceland; 5 Department of Biology, Georgia Southern University, Statesboro, Georgia, United States of America; 6 Mount Desert Island Biological Laboratory, Salisbury Cove, Maine, United States of America; University of Lausanne, Switzerland

## Abstract

A major physiological barrier for aquatic organisms adapting to terrestrial life is dessication in the aerial environment. This barrier was nevertheless overcome by the Devonian ancestors of extant Tetrapoda, but the origin of specific molecular mechanisms that solved this water problem remains largely unknown. Here we show that an ancient aquaporin gene cluster evolved specifically in the sarcopterygian lineage, and subsequently diverged into paralogous forms of AQP2, -5, or -6 to mediate water conservation in extant Tetrapoda. To determine the origin of these apomorphic genomic traits, we combined aquaporin sequencing from jawless and jawed vertebrates with broad taxon assembly of >2,000 transcripts amongst 131 deuterostome genomes and developed a model based upon Bayesian inference that traces their convergent roots to stem subfamilies in basal Metazoa and Prokaryota. This approach uncovered an unexpected diversity of aquaporins in every lineage investigated, and revealed that the vertebrate superfamily consists of 17 classes of aquaporins (Aqp0 - Aqp16). The oldest orthologs associated with water conservation in modern Tetrapoda are traced to a cluster of three *aqp2*-like genes in Actinistia that likely arose >500 Ma through duplication of an *aqp0-like* gene present in a jawless ancestor. In sea lamprey, we show that *aqp0* first arose in a protocluster comprised of a novel *aqp14* paralog and a fused *aqp01* gene. To corroborate these findings, we conducted phylogenetic analyses of five syntenic nuclear receptor subfamilies, which, together with observations of extensive genome rearrangements, support the coincident loss of ancestral *aqp2-like* orthologs in Actinopterygii. We thus conclude that the divergence of sarcopterygian-specific aquaporin gene clusters was permissive for the evolution of water conservation mechanisms that facilitated tetrapod terrestrial adaptation.

## Introduction

Adaptive transitions of organisms between aquatic and terrestrial environments are surprisingly rare during evolution. Available data on biodiversity in water and land reveal that only a restricted subset of the major clades of plants, animals, fungi and microbes have radiated on land [1] The emergence of Tetrapoda from their ancestral aquatic habitats during the Devonian was thus a key transitionary Period that ultimately led to the successful radiation of vertebrates in terrestrial environments [2]. To achieve this success, the ancient ancestors of modern Tetrapoda had to overcome major physiological challenges that included locomotion, breathing and dessication in an aerial environment.

The former problem of locomotion was solved through the evolution of limbs from fins, and considerable research has thus focused on the genes and signalling pathways that alternatively specify the chiridian limbs of tetrapods or the rayed fins of actinopterygian fishes [3]–[8]. These investigations revealed, however, that the same transcription factors, including members of the homeobox (*hox*) clusters, T-box (*tbox*) clusters and retinoic acid receptors (*rar*), are involved in the embryonic formation of the limbs and fins of each lineage. A solution to the second problem of aerial respiration was realised through the early evolution of an air-filled organ that differentiates from the anterior gut as a ventral lung in Sarcopterygii and the most basal actinopterygian fishes (Polypteriformes), or as a dorsal swimbladder in all other Actinopterygii [9]–[11]. The evolution of air-breathing was thus not restricted to the prototetrapod lineage, but has existed since the Paleozoic origin of the osteichthyan fishes (Euteleostomi), with 46 actinopterygian families also having extant species that display this trait [12]. As for the specification of sarcopterygian limbs and actinopterygian fins, recent evidence has shown that conserved transcription factors, including homeobox (*nkx2.1*), forkhead box (*foxa2*), wingless (*wnt7b*), and GATA binding proteins (*gata6*), direct the development of both sarcopterygian lungs and actinopterygian swimbladders [11]. Consequently, although chiridian limbs are specific to Tetrapoda, and lungs are specific to all Sarcopterygii, the genes involved in their formation are not, they are ubiquitous among all jawed vertebrates (Gnathostomata).

A solution to the adaptive problem of water conservation was first postulated >150 years ago when Claude Bernard introduced the concept of the “milieu interieur” [13]. Chief among the physiological mechanisms that control water homeostasis in Mammalia was the evolution of the kidney and the counter-current mechanism of water conservation (Smith 1953). Extant Mammalia have evolved a tertiary kidney (metanephros) and an impressive counter-current mechanism of water conservation (osmolar urine/plasma ratio: U/P≤25) that in certain groups, such as members of the Rodentia, obviates the need for drinking [14]. The process is mediated through neurohypophysial secretion of vasopressin and type-2 receptor (AVPR2)-mediated trafficking of aquaporin-2 (AQP2) water channels to the apical membrane of the principal cells of renal collecting ducts (antidiuresis) (Deen et al. 1994; Boone and Deen 2008). Although birds also evolved a metanephros and a functional AQP2 mechanism, they show a lesser ability to concentrate urine (U/P≤2.5) [15]. The reduced U/P ratios are due primarily to a paucity of long medullary loops of Henle and the lack of a urea habitus [16]. Reptiles also lack a urea habitus and did not evolve long medullary loops of Henle and thus do not have urine concentrating abilities. As members of the Sauropsida, the reptiles and birds evolved a uric acid habitus [17], [18], a defining metabolic feature of this clade. The almost insoluble nature of this end-product of protein metabolism substantially reduced the need for water excretion and likely alleviated the selection pressure on the evolution of their kidneys. Possibly as an additional adaptation to compensate for the poor counter-current urine concentration ability of the metanephros, sauropsids also evolved a nasal or lingual gland, a homologous organ to the rectal gland of Chondrichthyes (chimaeras, sharks and rays), that secretes concentrated saline (NaCl) [19]–[21]. In the avian lineage, it has been shown that a different water channel (AQP5) is expressed in this organ, and that under osmotic stress avian AQP5 is transcriptionally downregulated in the nasal gland in order to promote hyperosmotic salt secretion and water conservation [22].

In contrast to Mammalia and Sauropsida, extant Amphibia have a secondary kidney (mesonephros), which like the mesonephri of Teleostei, is unable to concentrate their urine above that of their blood plasma osmolarity (U/P≤1). Studies of aquaporins in Amphibia have, however, revealed that AQP2-like (AQP-h2k, HC-2) and AQP5-like (AQP-x5) channels are involved in water conservation [23], [24]. AQP-h2k is localised in the kidney tubules in response to arginine vasotocin (AVT) [25], [26], the vasopressin ortholog that co-evolved in the vertebrate lineage [27], and AQP-x5 mediates fluid secretions in the mucous and small granular glands [28]. The function of AQP-x5 is suggested to aid in the maintenance of moist skin, cutaneous gas exchange and thermoregulation [24], and is thus partly reminiscent of mammalian exocrine sweat glands, which also express AQP5 [29]. In Amphibia, such as hylid treefrogs and bufonid desert toads, additional anuran-specific water channels (*AQP-h3* and *AQP-h2*; *AQPa2*-type) have been identified that respectively respond to AVT in the ventral skin and urinary bladder to mediate water uptake and water conservation [24], [30]–[35].

From the above, it is clear that AQP2, -5, and the anuran AQPa2-type water channels are intricately involved in the mechanistic basis of water conservation in the three extant clades of terrestrial vertebrates, the Amphibia, Sauropsida and Mammalia. Amongst fish, a recent functional analysis of zebrafish aquaporins revealed that the tertiary structures including the six transmembrane domains, two NPA motifs and aromatic/arginine (ar/R) selectivity filters as well as the permeation properties are similar to the mammalian counterparts [36]. Consequently, both piscine and mammalian aquaporins can be phylogenetically and functionally classified as water-selective classical aquaporins (AQP0, -1, -2, -4, -5 and -6), an ammoniaporin (AQP8), which transports water, ammonia and urea, unorthodox aquaporins (AQP11, and -12) for which cell permation properties have yet to be identified, and classical aquaglyceroporins (Glp: AQP3, -7, -9 and -10), which facilitate the transport of water, arsenic, urea and polyols such as glycerol [36]–[41]. However, although the genomes of the five species of Teleostei studied were found to retain a larger repertoire of aquaporins due to an independent genomic duplication event at the root of the crown clade, they appeared to lack orthologs of AQP2, -5, or -6 [36], [42], [43]. By contrast, a study of lungfishes (Dipnoi) revealed that this ancient lineage of sarcopterygian fishes possess an AVT-AVPR2-AQP2-like system similar to Mammalia [44]. Under drought conditions, the lungfishes estivate and activate this system to regulate an Aqp0-related channel (Aqp0p) to promote antidiuresis [44]. Based upon the preliminary evidence that AQP2, -5, or -a2-related orthologs are absent in actinopterygian fishes [36], [42], [43], we hypothesised that the evolution of these water channels may represent a genomic apomorphy that was positively selected in basal Sarcopterygii as a prelude to terrestrial adaptation. To investigate this hypothesis, we combined selected aquaporin sequencing from agnathan (jawless) and gnathostome (jawed) vertebrates with the assembly of >5200 peptide fragments and their corresponding exon-like sequences and used Bayesian inference to reconstruct the aquaporin superfamilies encoded in 131 deuterostome genomes (see Figure S1 for species interrelationships). The systematic approach of assigning contiguous exon-like fragments to specific subclasses uncovered an unexpected diversity of aquaporins in basal Deuterostomia, and several novel subfamilies of water channel in Vertebrata (Aqp14, -15 and -16). To explain this diversity and the origin of the deuterostome aquaporins, we used Bayesian inference to develope a convergent model that traces the deep evolutionary origins to highly diversified stem subfamilies in Radiata, Porifera, Bacteria and Archaea.

## Materials and Methods

### Biological samples

Specimens of Smaller spotted catshark were obtained from L'Aquàrium de Barcelona, (Barcelona, Spain) and immediately sacrificed following the procedures related to the care and use of the fish approved by the Ethics Committee of the Institut de Recerca i Tecnologia Agroalimentàries (IRTA, Spain) in accordance with the “Guiding Principles for the Care and Use of Laboratory Animals”. Frozen samples of eyes from US and Iberian Peninsula sea lampreys were obtained from Hammond Bay Biological Station (Michigan, USA), Center for Molecular and Comparative Endocrinology, University of New Hampshire (New Hampshire, USA), and Centre of Environmental Biology, University of Lisbon (Lisbon, Portugal). Specimens of spiny dogfish and Atlantic hagfish were provided by the Mount Desert Island Biological Laboratory (MDIBL; Salisbury Cove, ME, USA) and were treated in accordance with IACUC regulations.

### Isolation of aquaporin transcripts

To obtain sequences related to the ancestors of AQP2, -5 or -6, aquaporin encoding cDNAs were isolated by RT-PCR following RNA extraction from visual, metabolic and osmoregulatory tissues, including eye, liver, rectal gland and kidney, using the RNeasy Minikit (Qiagen GmbH, Hilden, Germany), and treated with DNAse using the RNase-Free DNase kit (Qiagen) following the manufacturer's instructions. Total RNA (5 µg) was reverse transcribed using 0.5 µg oligo(dT)17, 1 mM dNTPs, 40 IU RNAse inhibitor (Roche Applied Science, Mannheim, Germany), and 10 IU SuperScript II Reverse Transcriptase enzyme (Life Technologies Corp. Carlsbad, CA), for 1.5 h at 42°C. The PCR was carried out with 0.5-2 µl of the RT reaction in a final volume of 50 µl containing 1 x PCR buffer plus Mg^2+^, 0.2 mM dNTPs, 1 IU of Taq polymerase (Roche), and 1 µM of primers. Degenerate or specific primers based on available genomic sequences were used to amplify partial cDNAs (Table S1). The PCR products were cloned into the pGEM-T Easy Vector (Promega) and sequenced by BigDye Terminator v3.1 cycle sequencing on ABI PRISM 377 DNA analyzer (Applied Biosystems). The 5′end bearing the C-terminus of sea lamprey *aqp01* was cloned by 5′-RACE (Life Technologies Corp.) (Table S1). The spiny dogfish *aqp15* and Atlantic hagfish *glp* cDNAs were also amplified by RT-PCR using total RNA extracted (as previosuly described; [45]) from kidney and esophagus respectively, and using inosine containing degenerate primers (Table S1). Total RNA (4.5 µl) was reverse transcribed using 100 pmole oligo(dT)26, 1 mM dNTPs, 10 IU SUPERase.In^TM^ thermostable RNAse inhibitor, and 100 IU SuperScript III Reverse Transcriptase enzyme (Life Technologies Corp. Carlsbad, CA), for >1 h at 50°C. The degenerate PCR was carried out with 0.5–1 µl of the RT reaction in a final volume of 20 µl containing 1 x standard PCR buffer, 0.2 mM dNTPs, 1.25 IU of Taq polymerase (New England Biolabs, Ipswich, MA), and 5 µM primers. Degenerate or specific primers based on available sequences were used to amplify partial cDNAs. 5′ or 3′ RACE cDNA sequences were amplified using a Marathon cDNA synthesis kit (Clontech, Mountain View, CA) and using Phusion DNA Polymerase (New England Biolabs, Ipswich, MA). cDNA fragments were cloned using a TOPO TA Cloning Kit for Sequencing (Life Technologies Corp. Carlsbad, CA) and Sanger sequenced by MDIBL (Salisbury Cove, ME) or the Clemson University Genomics Institute (CUGI; Clemson, SC).

The nucleotide sequences of smaller-spotted catshark *aqp0*, *-1*, *-4*, spiny dogfish *aqp15*, sea lamprey *aqp01*, *-3L1*, *-3L2*, and Atlantic hagfish *glp* are deposited in GenBank under accession numbers KJ784515, KJ784516, KJ784517, KJ815007, KJ784520, KJ784518, KJ784519 and KJ815008, respectively.

### Phylogenetic and Syntenic Analyses

Aquaporin orthologs were initially retrieved from public databases (ensembl and GenBank), and the deduced amino acid sequences aligned with default t-coffee v9.01 [46] or L-INS-I MAFFT v7.058b [47] algorithms. These data were converted to codon alignments using Pal2Nal [48] and analysed using Bayesian (Mr Bayes v3.2.2; [49]) and maximum likelihood (PAUP v4b10-x86-macosx; [50]) protocols as described previously [51], [52]). Phylogenetic sorting was then achieved by arranging the sequences in accordance with the resulting tree topologies, and errors in the automated alignment were identified and corrected manually using MacVector (MacVector Inc, Cambridge, UK). Phylogenetic analyses of the deuterostome superfamily were performed on the conserved transmembrane regions between human AQP6 Lys^21^-Pro^237^, following removal of the N- and C-termini, while phylogenetic analyes of the separate classes of aquaporin were performed on full-length sequences. For Bayesian analyses the following models were tested for the codon alignments: nucmodel  =  4by4 with nst  =  2 or codon with nst  =  6; rates  =  gamma and invgamma, respectively; and for amino acid alignments: aamodel  =  mixed. Based upon the resulting tree topologies and posterior probabilities, no significant differences were noted between nst  = 2 or 6, and subsequent analyses utilised the more tractable setting of nst  = 2. Markov chain Monte Carlo (MCMC) algorithms were run with 3 heated chains and 1 cold chain with 30 million MCMC generations for the superfamily alignment. Computation time thus varied from several days to 3.5 months for a given alignment. Each run was examined for convergence using Tracer version 1.5 (tree.bio.ed.ac.uk/software/tracer/), and majority rule consensus trees summarized with a burnin of 25%. All trees generated were processed with Archaeopteryx [53] and rendered with Geneious (Biomatters Ltd, New Zealand).

To establish the presence or absence of orthologs in specific taxonomic groups, and to complete partial or poorly predicted sequences, the tblastn algorithm was used to identify 5394 non-redundant aquaporin proteins or protein fragments from whole genome-shotgun (WGS), transcriptome shotgun assemblies (TSA) and expressed sequence tag (EST) databases (www.ncbi.nlm.nih.gov). We initially used full-length aquaporins as reference for the tblastn algorithm, and subsequently used aquaporin regions corresponding to translated exons to retrieve novel sequences. Contiguous nucleotide sequences were then retrieved and trimmed to match each protein fragment, and subsequently concatenated to construct a putative cDNA for each gene. Deduced amino acid sequences from the putative cDNAs were manually incorporated into the alignments previously established using t-coffee or MAFFT, which was then converted to a codon alignment using Pal2Nal. Separate analyses of 10 – 30 million MCMC generations were performed on full-length data sets comprised of classical aquaporins (Aqp0, -1, -2, -4, -5, -5L, -6, -14 and -15), Aqp8 and -16, unorthodox aquaporins (Aqp11 and -12), and aquaglyceroporins (Aqp3, -7, -9, 10 and -13). A full list of accession numbers is provided in Table S2.

To validate the topology of the resulting trees, aquaporins were localised to chromosomes in assembled genomes, and syntenic analyses conducted for conserved flanking genes using the Genomicus (www.dyogen.ens.fr) and ensembl genome browsers or manually assembled via *in silico* chromosomal and contig walking. This approach identified several conserved nuclear receptors (*nr1b*, *-1d*, *-1f*, *-1i* and *nr4a*), which we then examined in a phylogenetic framework as described above based upon 2235 assembled sequence fragments (see Table S3 for accession numbers). These latter analyses were used to evaluate the historical genome duplication events and gene losses associated with aquaporin evolution. Divergence times of the different lineages are based upon previously published timetree data [54].

### Accession numbers

Accession numbers of novel sequences: KJ784515, KJ784516, KJ784517, KJ784518, KJ784519, KJ784520, KJ815007, KJ815008.

## Results

### Four Major Grades of Deuterostome Aquaporin have Parazoan-Radiata Origins

**Figure 1:**
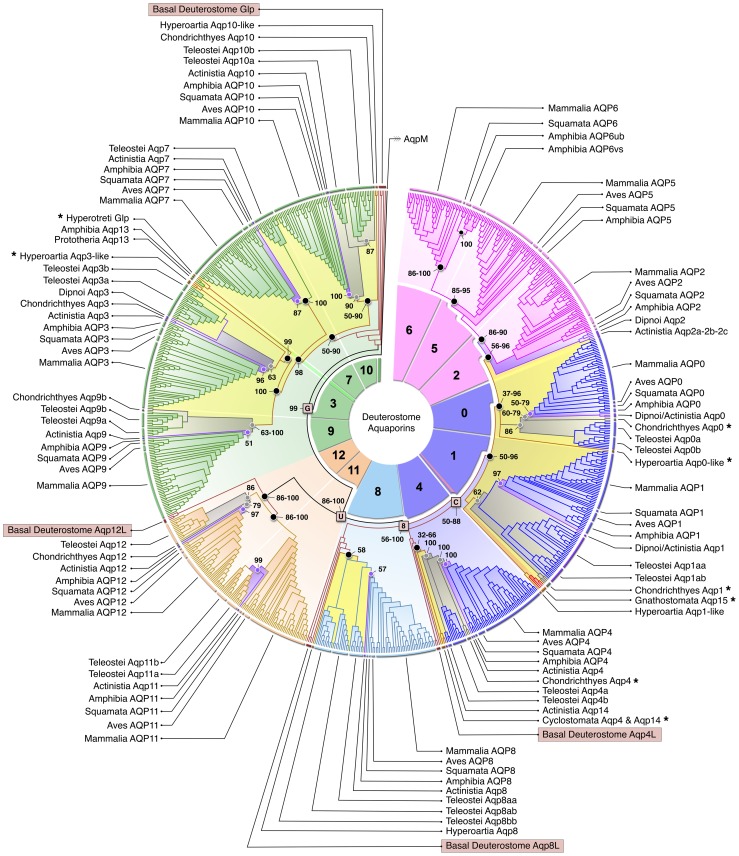
Preliminary Molecular Phylogeny of the Deuterostome Aquaporin Superfamily. The Bayesian majority rule consensus tree of the codon alignment is rooted with *aqpM*. The aquaporin subfamilies annotated in the central coil are separated into four major grades: aquaglyceroporins (G), unorthodox aquaporins (U), aquaporin 8-type (8) and classical aquaporins (C). Posterior probabilities are shown at selected nodes. * indicates sequencing of taxon. Evolutionary older nodes associated with Cyclostomata, Chondrichthyes and Actinisia are respectively shaded in yellow, grey and magenta. Teleost and tetrapod subclusters are shaded according to the aquaporin grade, except for sarcopterygian Aqp2, -5, and -6 paralogs, which are shaded in pink.

Initial experiments using degenerate primers to identify aquaporins in Chondrichthyes, Hyperoartia (lampreys) and Hyperotreti (hagfishes) resulted in the isolation of four putative classical aquaporins from the two species of shark studied (smaller-spotted catshark, *Scyliorhinus canicula*; and spiny dogfish, *Squalus acanthias*), two partial aquaglyceroporins from the sea lamprey (*Petromyzon marinus*) and a full-length putative aquaglyceroporin from the Atlantic hagfish (*Myxine glutinosa*). Each sequence was incorporated into the superfamily alignment composed of 852 non-redundant aquaporin transcripts assembled from basal Deuterostomia (Echinodermata), Protochordata (Cephalochordata and Tunicata), Agnatha (Hyperotreti and Hyperoartia), and Gnathostomata (Chondrichthyes, Teleostei, Actinistia, Amphibia, Sauropsida and Mammalia). Incremental Bayesian analyses of the deduced amino acid and codon alignments revealed that the thirteen subfamilies (*AQP0* - *AQP12*) established for Mammalia [55], [56] and the duplicated counterparts amongst the 10 corresponding subfamilies identified in Teleostei [36], [42], [43] are well resolved and can be traced to four major grades of water channel in Echinodermata and Protochordata (Figure 1). The term grade is used to reflect the polyphyletic nature of the four major aquaporin subdivisions. We thus initially phylogenetically classified the four major grades of deuterostome water channels based on their functional properties as classical, water-specific aquaporins (Aqp4, -1, -0, -2, -5, -6), aquaglyceroporins (Glp: including Aqp3, -7, -9, and 10), aquaammoniaporins (Aqp8-type), or unorthodox aquaporins (Aqp11, -12) [37]–[40].

Surprisingly, however, the genomes of Echinodermata, Hemichordata and Cephalochordata are found to encode multiple copies of the different grades of aquaporin. To validate these observations, we researched the basal deuterostome WGS, TSA and EST databases, and separately analysed 81 assembled orthologs. These latter analyses revealed the existence of five *aqp4*-like, two to three *aqp8*-like, two *aqp12*-like and three *glps* in Echinodermata, and between one to five paralogs of each grade in the Protochordata (Figure S2). Based upon the loci and clustering of the encoded transcripts, and the low sequence conservation (23–36% deduced amino acid identities between paralogs), it is apparent that duplication of the echinoderm aquaporins occurred prior to the divergence of the echinozoan and asterozoan lineages, with at least two gene couplets, including *aqp4l2*-*aqp4l3* and *aqp4l4*-*aqp4l5*, which are respectively colocated on purple sea urchin Scaffold664 and Scaffold916, evolving via tandem duplication. Tandem duplication is also the likely origin of the two *aqp12-like* paralogs in Cephalochordata, which are juxtaposed in scaffold 207_Cont30099 of the Florida lancelet (*Branchiostoma floridae*).

To investigate the putative origin and establish a potential root for the four major aquaporin subdivisions, we searched for ancestral eukaryotic sequences within the genomes of Cnidaria (anemones and corals), Porifera (sponges), Fungi and Protists, and to deeper prokaryotic origins in Bacteria and Archaea. The results revealed that expansion of the superfamily into four major grades occurred prior to the evolution of Bilateria (see Text S1, Figure S3–S4), and that Prokaryota encode a surprising diversity of water channels, including a novel clade (AqpN) in Bacteria and Archaea (see Text S1, Figure S5 and Table S2 for the details). The latter data further indicate that there is no clear phylogenetic root for aquaporins, however, AqpM may be considered a good approximation for metazoan trees due to its central position in the mid-rooted prokaryotic tree (Figure S5).

The systematic approach of assembling contiguous exon-like fragments to putative transcripts and establishing their orthology via Bayesian analyses identified an extensive array of non-redundant members of the aquaporin superfamily, including 590 Glps, 286 unorthodox, 221 Aqp8-type and 1004 classical aquaporins in deuterostome organisms (see Text S1). These data revealed that Vertebrata encode a broader repertoire of Glps than previously documented (Figure S6), including several tandem duplicates and a novel subfamily (Aqp13) (Figure S7), that unorthodox aquaporins (Aqp11, and -12) are ubiquitous in Gnathostomata (Figure S8), and that tetraploid Teleostei such as the Atlantic salmon encode up to eight Aqp8-type channels, while diploid Tetrapoda encode only one but retain an additional subfamily (Aqp16) (see Text S1, Figure S9–S10 for the details). In conjunction with the selected sequencing, the assembled data also revealed that two novel classical aquaporin subfamilies (Aqp14 and -15) exist in diverse lineages of vertebrate, which appear to be degraded in Eutheria (see next section).

### Diversification and Lineage-Level Loss of Novel Aquaporin Subfamilies in Vertebrata

Phylogenetic analyses of the classical aquaporin-like sequences retrieved from the sea lamprey genome reveal that one can be classified as *aqp4*, while three more partial sequences cluster as *aqp4*-like, *aqp1*-like and *aqp0*-like subfamily members (Figure 1). The chondrichthyan classical aquaporin sequences isolated from the kidney, rectal gland and eye in the present study, respectively clustered as members of the *aqp4*, *-1*, and *-0* subfamilies, while the fourth full-length sequence isolated from the kidney of the spiny dogfish clustered on a sister branch to the *aqp1* subfamily together with the sea lamprey *aqp1*-like sequence, the previously analysed zebrafish *aqp5/1b* duplicate [36], [57] and a novel coelacanth (*Latimeria chalumnae*) *aqp1*-related ortholog (Figure 1). Confirmation that the newly isolated spiny dogfish *aqp1*-related sequence and the novel coelacanth *aqp1*-related ortholog are not derived isoforms of a gnathostome *aqp1* channel was achieved by assembling the full compliment of aquaporins from the ghost shark (*Callorhinchus milii*), little skate (*Leucoraja erinacea*) and coelacanth genomes. The Bayesian analyses shown in Figure 1 indicated that the genome of each species encodes an orthologous *aqp4*, *-1* and *-0* channel, suggesting that the *aqp4*-like and *aqp1*-like channels might represent novel aquaporins not yet identified in Mammalia or any other organism. Equally striking is the observation that three basal *aqp2*-like paralogs retrieved from the actinistian coelacanth genome (2.6 Mb locus, Scaffold JH126563) co-cluster with the two dipnoan *aqp0p* orthologs previously identified in lungfish [44], just below the *AQP2*, *-5* and *-6* orthologs assembled from tetrapod genomes. The cluster pattern of the tetrapod *AQP2* and *-5* orthologs was sometimes reversed with equal levels of posterior probability. The preliminary phylogenetic data nevertheless seemed to support our earlier analyses [36], [42], [43] that Teleostei lack functional orthologs of *AQP2*, *-5* and *-6*. In addition the present observation that two novel classical aquaporins exist in basal gnathostome and agnathan animals suggested that these orthologs might represent ancestral forms of *AQP2*, *-5* or *-6* found in Tetrapoda.

**Figure 2:**
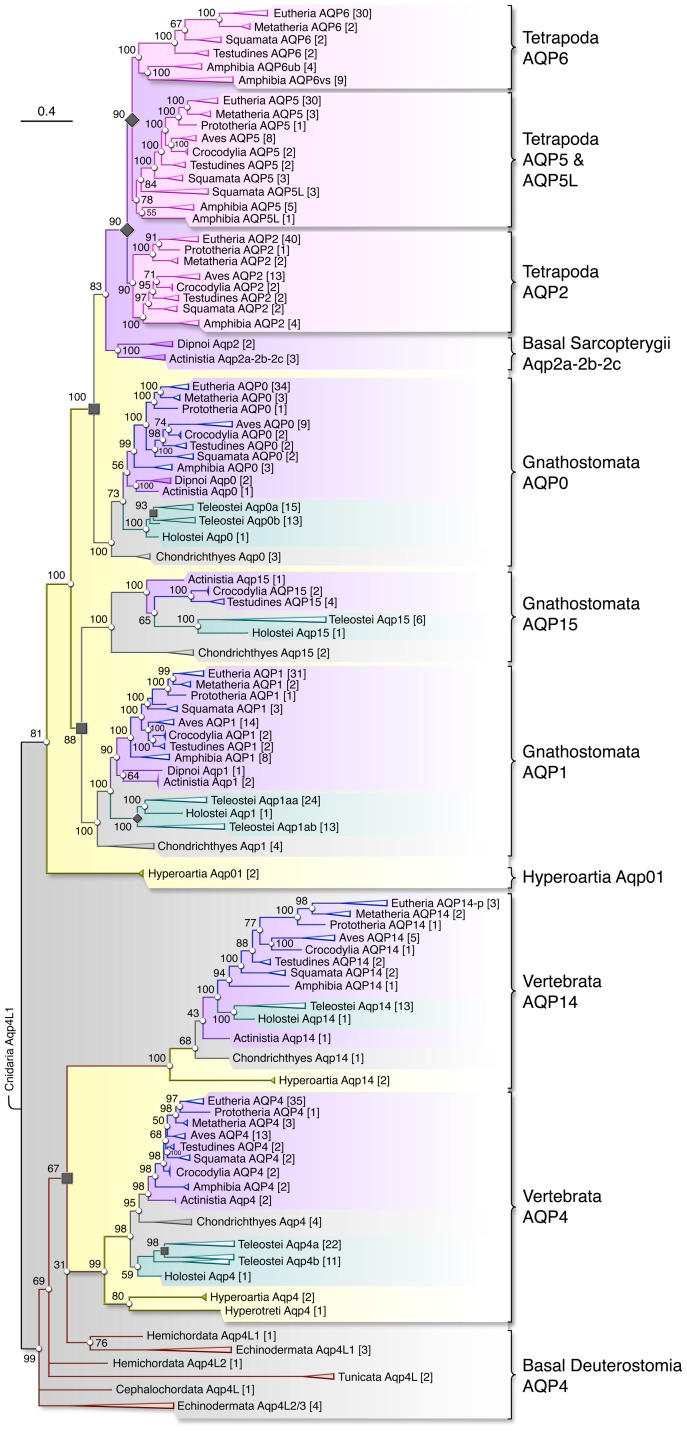
Molecular Phylogeny of Deuterostome Classical Aquaporins. Bayesian majority rule consensus tree of the codon alignment is rooted with cnidarian *aqp4L1*. Posterior probabilities are shown at each node, with the number of taxa analysed given in square brackets. The scale bar represents the rate of nucleotide substitution per site. Chondrichthyan, actinopteryian and sarcopteygian subclusters are respectively shaded in light grey, cyan and magenta, while evolutionary older nodes associated with Cyclostomata and basal Deuterostomia are respectively shaded in yellow and dark grey. The tetrapod AQP2, -5, -5-like (5L), and -6 paralogs are shaded in pink. See Figure S11 for the fully annotated tree.

**Figure 3:**
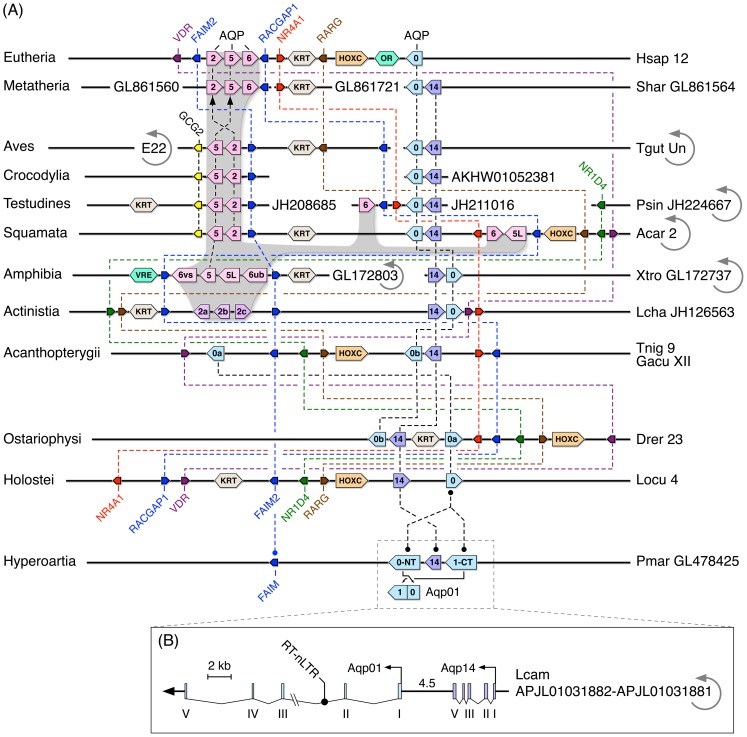
Syntenic arrangement of the vertebrate aquaporin gene clusters. (**A**) Synteny is shown in relation to conserved nuclear receptors, and the keratin (KRT), olfactory receptor (OR), vomeronasal receptor (VRE), homeobox C (HOXC) superclusters. Circular arrows indicate that the linkage group is flipped, and coding direction is indicated by the pointed end of the gene symbols. (**B**) Genomic structure of the Arctic lamprey *aqp01* and *-14* paralogs.

To validate the intial findings outlined in Figure 1, we extended the assembly of aquaporin repertoires to include 28 available teleost genomes, together with that of the holostean spotted gar (*Lepisosteus oculatus*). This resulted in the identification of 2104 peptides, which, together with the corresponding nucleotides, were assembled into 673 actinopterygian-related aquaporins. In addition, to discern whether the novel aquaporins isolated or assembled from lampreys, sharks and the coelacanth are specific to these animals, or represent ancestral orthologs that are lost in modern taxonomic lineages, we expanded our search of the vertebrate genomes to include 89 tetrapod genomes, and examined the syntenic relationships of the vertebrate *aqp0*, *-2*, *-5* and *-6* loci (see below). This allowed two separate data sets consisting of 485 full-length non-redundant classical aquaporin proteins and transcripts to be aligned and analysed using Bayesian protocols. The resulting trees (Figure 2 and Figure S11) were rooted with cnidarian *aqp4L1*, which we identified as a basal metazoan classical aquaporin (see Text S1), and revealed that extant gnathostome genomes encode eight subfamilies of classical aquaporin rather than six as previously reported [55], [56], [58]. The most basal subfamilies in Vertebrata include canonical *aqp4* and the novel *aqp4*-like orthologs in Cyclostomata and all major clades of Gnathostomata. By localising the sea lamprey *aqp4*-like sequence to scaffold GL478425, we noted that it is encoded upstream of the *aqp0*-like sequence, and downstream of the *aqp1*-like sequence (Figure 3A, Figure S14). This prompted us to search for exons of an aquaporin homolog upstream of *aqp0* in each gnathostome lineage, and in doing so we identified 68 orthologs of the sea lamprey *aqp4*-like gene, including near complete forms in the genomes of the oldest mammalian lineages, Prototheria and Metatheria. In Eutheria, however, we have currently only found exon fragments in members of the Xenarthra, Cetartiodactyla, Chiroptera and Carnivora. The C-terminal fragment identified in the genome of the bottlenosed dolphin (*Tursiops truncatus*) also contains a premature stop codon, supporting the notion that this branch of aquaporins is degraded to pseudogenes and is thus functionally extinct in Eutheria. Considering that the novel *aqp4*-like orthologs cluster on a sister branch to the canonical *aqp4* transcripts, and that both are encoded in the genomes of Cyclostomata and Gnathostomata, we named the novel subfamily Aqp14 as a putative product of early chordate WGD. Although members of Aqp14 subfamily retain the cononical NPA and NPAR motifs, the low conservation of the primary structures (40–70% amino acid identity compared to methatherian Aqp14; 25–36% amino acid identity compared to human AQP0, -1, -2, -4, -5 or -6) indicates that Aqp14 channels did not evolve under strong purifying selection. Indeed, the divergence of Aqp14 channels from other classical aquaporins is clearly reflected in the longer branch lengths, but is also evident in the absence of aromatic residues in the ar/R selectivity filter [59], [60]. In lieu of the aromatic F on the α-helical transmembrane domain 2 (TMD2) and H on TMD5, the Aqp14 selectivity filter presents residues with non polar aliphatic side chains including A, V or L (TMD2), and A, V, L, I (TMD5), with some avian and metatherian orthologs encoding polar, uncharged T in the latter position (Table 1). Despite these differences, comparison of the five residues (P1–P5) that putatively distinguish between the molecular selectivity of aquaporins and aquaglycroporins [61] confirms that vertebrate Aqp14 channels retain the signature P2–P5 residues of classical water-specific aquaporins (Table S4).

**Table 1:** Classical aquaporin aromatic-arginine constriction residues in Deuterostomia.

	AQP4				AQP14				AQP1				AQP15				AQP0				AQP2				AQP5				AQP6			
	P1	P2	P3	P4	P1	P2	P3	P4	P1	P2	P3	P4	P1	P2	P3	P4	P1	P2	P3	P4	P1	P2	P3	P4	P1	P2	P3	P4	P1	P2	P3	P4
Eutheria	F	H	A	R					F	H	C	R					F	H	A	R	F	H	C	R	F	H	C	R	F	H	C	R
Metatheria	F	H	A	R	G	T	G	R	F	H	C	R					F	H	A	R	F	H	C	R	F	H	C	R	F	H	C	R
Sauropsida	F	H	A	R	A-G	A-T	G	R	F	H	C	R	F	H	C	R	F	H	A	R	F	H	C	R	F	H	C	R	F	H	C	R
Amphibia	F	H	A	R	A	A	G	R	F	H	C	R					F	H	A	R	F	H	C	R	F	H	C	R	F	H	C	R
Dipnoi	F	H	A	R					F	H	C	R					F	H	A	R	F	H	T	R								
Actinistia	F	H	A	R	A	I	G	R	F	H	C	R	F	H	C	R	F	H	A	R	F	H	T-A	R								
Teleostei (a)	F	H	A	R	V-A	I-V	A-G	R	F	H	C	R	F	H-N	C	R	F	H	A	R												
Teleostei (b)	F	H	A	R					F	H	C	R					F	H	A	R												
Holostei	F	H	A	R	A	V	G	R	F	H	C	R	F	H	C	R	F	H	A	R												
Chondrichthyes	F	H	A	R	A	V	G	R	F	H	C	R	F	H	C	R	F	H	C	R												
Cyclostomata	F	H	A-S	R	A	I	G	R																								
Basal Deuterostomia	F-N-I	H-L	A-S	R																												

Residues conserved with AQP4 are highlighted in light blue, residues conserved with AQP1 are highlighted in pink.

Residues P1 - P4 (F77, H201, A210, R216, respectively) are identified after following alignment of orthologs to human AQP4 (ENSP00000311165).

Orthologous ar/R residues conserved to human AQP4 or AQP1 are highlighted in blue and pink, respectively. Taxonomically conserved residues for Aqp14 are highlighted in grey.

We next focused on establishing the orthology of the novel *aqp1*-related transcript isolated from the kidney of the spiny dogfish. The deduced amino acid sequence encodes the six canonical α-helical TMDs, the two NPA motifs, the signature ar/R residues of a classical aquaporin and the P1–P5 residues conserved in tetrapod AQP2, -5 or -6 channels. BLASTp returned hits that were equally related (∼50% amino acid identity) to AQP0, -1 or -5 indicating that this chondrichthyan ortholog could represent an ancestral form of the tetrapod channels. However, we successfully identified orthologous chondricthyan, actinopterygian and sarcopterygian sequences, which when assembled and submitted to Bayesian inference, revealed that the spiny dogfish water channel is an ancestral form of a novel *aqp1*-related subfamily not found in Mammalia. Based upon the topological position of the subcluster, and syntenic relationship in the genomes of Eutelostomi (Figure S12), we named this subfamily *aqp15*, a duplicate of *aqp1* that putatively evolved from an ancient WGD event.

### The origins of vertebrate Aqp0-like and Aqp1-like Integral Membrane Proteins

**Figure 4:**
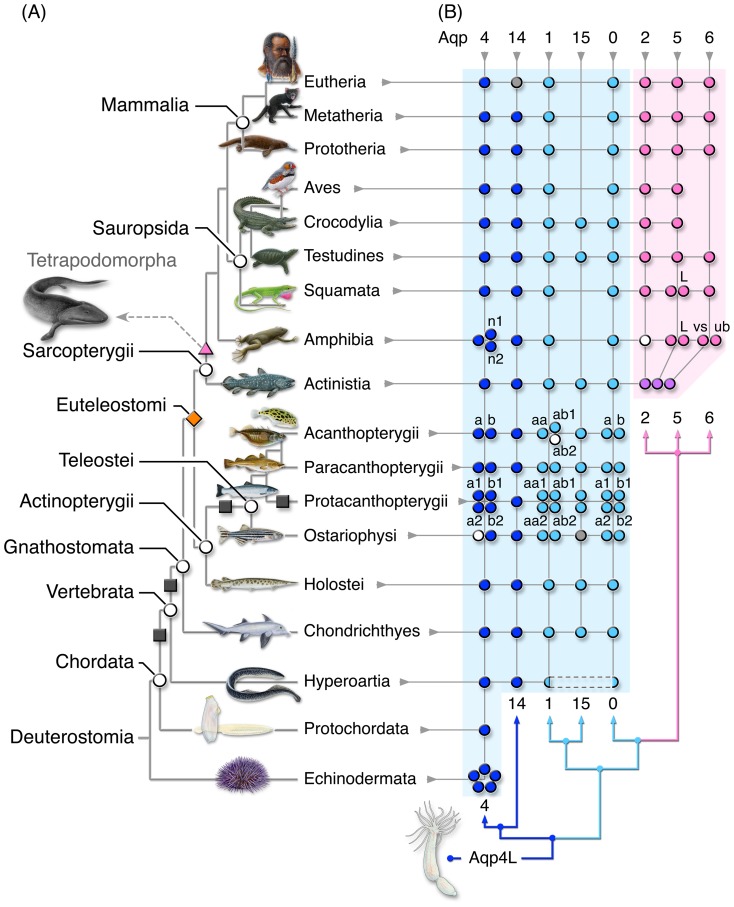
Evolutionary Distribution of Deuterostome Classical Aquaporins. **(A**) Phylogenetic relationships of the major deuterostome groups studied. (**B**) Prevalence of classical aquaporin genes identified in each lineage showing postscript nomenclature for duplicated forms. Coloured dots are specific for the species shown, white dots are found in the same lineage, but a different species, while grey dots indicate pseudogenes.

Having ruled out *aqp14* and *-15* genes as direct ancestors of tetrapod *AQP2*, *-5* or *-6*, we re-examined the partial sequences identified in lampreys. In the current version of the sea lamprey genome, the *aqp0*-like exons downstream of *aqp14* encode the N-terminal hemipore, while the *aqp1*-like exons upstream of *aqp14* encode the C-terminal hemipore (Figure 3A, Figure S14), indicating that the remaining hemipores of each integral membrane protein should be respectively located in the 3′ prime and 5′ prime regions of the DNA. However, although we were able to assemble full-length transcripts encoding both hemipores for *aqp4* (320 deduced amino acids) and *aqp14* (299 deduced amino acids) from the Arctic lamprey genome, only two and three exons are respectively retrieved for the partial *aqp0*-like and *aqp1*-like fragments in this latter species (Figure 3B). As for the sea lamprey, the *aqp0*-like exons are located downstream of *aqp14*. However, in contrast to the sea lamprey, the Arctic lamprey *aqp14*, *aqp0*-like and *aqp1*-like fragments are encoded in-frame on the reverse strand of two consecutive DNA fragments (APJL01031881, APJL01031882). This alternative arrangement suggested that the five exons might code for a single gene, rather than two as initially surmised. To test this possibilty, we designed forward and reverse primers for the two *aqp0* NPA motifs of the smaller-spotted catshark to be used for RT-PCR experiments on total RNA isolated from the eyes of the shark and the eye and kidney tissues of the two species of Hyperoartia, the sea lamprey and the European brook lamprey (*Lampetra planeri*). This experiment revealed a clear band of the expected size (∼570 bp) for the smaller-spotted catshark, but none for the lampreys (Figure S13). Subsequent sequencing of the 570 bp band confirmed its identity as smaller-spotted catshark *aqp0*. We therefore used the more complete exon data for the Arctic lamprey to design gene-specific primers for the *aqp0*-like and *aqp1*-like fragments and confirmed that each is expressed in a mixture of eye and kidney tissues obtained from the sea lamprey (data not shown). We cloned the *aqp1*-like C-terminal hemipore by RT-PCR and the use of 5′-prime RACE to isolate the N-terminal hemipore resulted in the amplification of a product, which when sequenced, is identical to the *aqp0*-like fragment, revealing that despite the non-contiguous arrangement of the exons in the sea lamprey genome, they are encoded by a single gene. Bayesian analyses of the concatenated lamprey nucleotides in a codon alignment consisting of 148 *aqp0* and 210 *aqp1* transcripts assembled from Gnathostomata, place the hyperoartian transcripts at the base of *aqp0* rather than *aqp1* with 80–86% prosterior probability (data not shown). However, as shown in Figure 2, when submitted to Bayesian inference in the broader context of all gnathostome classical aquaporins, the hyperoartian transcripts form a monophyletic root (81% posterior probability) to the gnathostome classical aquaporins comprised of *aqp1*, *-15*, *aqp0* and *-2*-like subfamilies. This surprising result indicated that seven classical aquaporins (*aqp0*, *-1*, *-2*, *-5*, *-5L*, *-6*, and *-15*), encoded by four exons in Gnathostomata, may have evolved from a single ancestral form encoded by five exons as noted for extant Agnatha. Closer inspection of the *aqp0*-like DNA of the Arctic lamprey, however, revealed an RNA-directed DNA polymerase from a jockey-like mobile element encoded in the second intron. We therfore cannot rule out the possibility that two independent classical aquaporins (*aqp0* and *aqp1*) evolved in Agnatha but have subsequently fused due to genomic rearrangements in lampreys. The most ancestral form identified in extant Hyperoartia is therefore annotated as *aqp01*. To explain this diversity, an overview of the classical aquaporins identified in Deuterostomia is shown in Figure 4.

### Genomic Rearrangements Unlink Hox Clusters as Conserved Markers of Aquaporin Evolution

To corroborate the preliminary phylogenetic analyses, we compared the karyotypic loci of the vertebrate aquaporin superfamily and examined the syntenic relationships of genes and superclusters flanking the Primate *AQP0*, *-2*, *-5*, and *-6* paralogs (Figure 3A and Figure S14). The HOX clusters are conserved amongst different metazoan Phyla, and have thus been used as markers of genome evolution [62]–[65]. In the present context the teleost HoxAa and HoxAb clusters are respectively linked with *aqp10a* and *-10b*, and the HoxBb and HoxBa clusters are respectively linked with the *aqp8aa*, *-8ab* and *-8bb* paralogs, providing support for both tandem duplication and an R3 WGD origin of these aquaporins. However, for the majority of vertebrate aquaporins, the lack of linkage conservation to the HOX clusters limits comparison of their duplication history. For example, with the exception of the fused chromosomes in the metatherian opossum (*Monodelphis domestica*), the linkage of the HOXB cluster to *AQP10* is not conserved in Tetrapoda. In the green anole (*Anolis carolensis*), the HOXB cluster is distally linked to *AQP1*, but in Eutheria, it is the HOXA cluster that is proximally linked to *AQP1* and *AQP4*, and while the latter linkage between *AQP1* and *AQP4* is not conserved in Glires, it may represent an ancestral condition since these paralogs are also linked in some avian and actinopterygian genomes. A similar state appears to have evolved in ancestral Euteleostomi for *aqp3* and *-7*, which are closely linked in the genomes of Actinopterygii and Sarcopterygii. A major exception is the HOXC cluster, which has remained proximally linked to gnathostome *aqp0* and *-14* at least since the Ordovician (>455 Ma), while the data for Hyperoartia reveal that the linkage between *aqp01* and *-14* has existed since the Cambrian.

A more holistic view of genomic rearrangements becomes apparent when mapping distantly related gnathostome linkage groups (LGs) encoding *aqp0* to the human karyotype (Figure S15). This analysis revealed that much of the gene content of Hsap4 is conserved from the *aqp0*-bearing orthologons of holostean fishes and galliform birds, such as the turkey (*Meleagris gallopavo*). Broader rearrangements occurred in the genomes of Teleostei after the lineage diverged from Holostei. Thereafter, teleost gene distribution of the *aqp0a* and *-0b* LGs remained relatively stable for >200 million years of evolution. Major rearrangements are also apparent from the *aqp0*-linkage map of the iguanian green anole compared to the turkey, while in Mammalia, approximately 70% of the gene content of Hsap12 was established >105 Ma, prior to the divergence of Afrotheria and Boreoeutheria. In the central regions of Hsap12 (48.2–57.7 Mb) we found that, despite the extensive genomic rearrangements, 33 genes in addition to the HOXC, keratin (KRT) and olfactory receptor (OR) clusters have remained syntenic to *AQP0* for >455 Ma.

### Convergent Loss of Syntenic Nuclear Receptors Reflects Aquaporin Evolution

Amongst the syntenic flanking genes, are four nuclear receptors, retinoic acid receptor G (*RARG*), Rev-erb (*NR1D4*), vitamin D receptor (*VDR*), and *NR4A1*. As a relatively large superfamily with broad distribution, nuclear receptors are regarded as good markers of animal genome evolution [66], so to compare their duplication history to the aquaporins in Deuterostomia, we examined the phylogenetic relationships of the syntenic nuclear receptors and included a fifth subfamily, the rar-related orphan receptors (*ROR*), since they are syntenic with the aquaglyeroporins. The intriguing results revealed remarkable parallels to the lineage-level loss of deuterostome aquaporins (Table 2, Figures S16–S20 and Table S3 for accession numbers). Beyond the loss of duplicates in the aftermath of the fish-specific WGD, the syntenic loss of *NR1D4* in avian and mammalian lineages and the related loss of *RORD* in Eutheria is highly reminiscent of the loss of *AQP6* in Archosauria and *AQP14* in Eutheria. Similarly the absence of the constitutive androstane receptor (*CAR*) in the genomes of Chondrichthyes and Actinopterygii mirrors the absence of *AQP2*, *-5* or *-6* in these same lineages, while Sauropsida have alternatively lost the paralogous pregnane-X receptor (*PXR*). In contrast the presence of tetraparalogous *RORs* that maintain highly conserved syntenies to the aqualgyeroporins of Euteleostomi, including the duplicated *aqp9a*, and *-9b* in Teleostei, suggests that positive and negative selection forces have converged to shape the genomic repertoires of nuclear receptors and aquaporins in Vertebrata.

**Table 2:** Presence and absence of gnathostome nuclear receptor subfamilies with syntenic paralogs (•) to aqp0, -14, -2, -5, -5L, -6, or (:) aquaglyceroporins.

	Chondrichthyes	Holostei	Teleostei (a)	Teleostei (b)	Actinistia	Amphibia	Squamata	Testudines	Crocodylia	Aves	Prototheria	Metatheria	Eutheria
RARA	+	+	+	+	+	+	+	+	+	+	+	+	+
RARB	+	+	+	+	+	+	+	+	+	+	+	+	+
• RARG	+	+	+	+	+	+	+	+	+	+	+	+	+
NR1D1	+	+	+	-	+	+	+	+	+	+	+	+	+
NR1D2	+	+	+	+	+	+	+	+	+	+	+	+	+
• NR1D4		+	+	+	+	+	+	+	+	-	-	-	-
CAR	-	-	-	-	+	+	+	+	+	+		+	+
PXR	+	+	+	+	+	+	-	-	-	-		+	+
• VDR	+	+	+	+	+	+	+	+	+	+	+	+	+
• NR4A1	+	+	+	+	+	+	+	+	+	+	+	+	+
NR4A2	+	+	+	+	+	+	+	+	+	+	+	+	+
NR4A3	+	+	+	-	+	+	+	+	+	+	+	+	+
: RORA	+	+	+	+	+	+	+	+	+	+	+	+	+
: RORC	+	+	+	-	+	+	+	+	+	+	+	+	+
: RORB	+	+	+	-	+	+	+	+	+	+	+	+	+
RORD	+	+	+	+	+	+	+	+	+	+		+	-
N analysed	32	18	215	130	17	31	32	32	16	67	12	31	90

### Apomorphic Aquaporin Gene Clusters in Sarcopterygii

**Figure 5:**
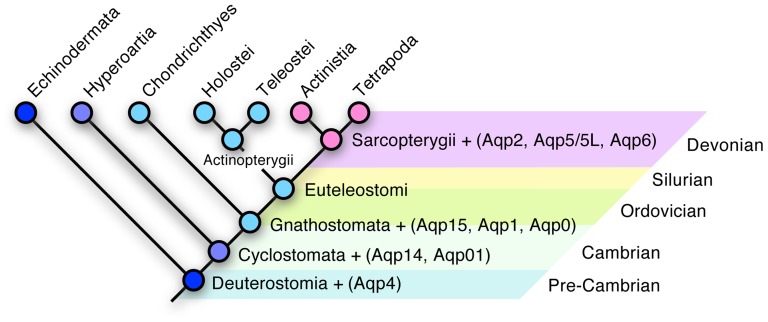
Evolution of the Aquaporin Apormorphy in Sarcopterygii. Schematic illustration showing aquaporin subfamilies identified in deuterstome phyla in relation to geological time.

Integration of the phylogenetic and syntenic analyses thus demonstrates that the tetrapod *AQP2*, *-5* and *-6* paralogs represent a genomic apomorphy specific to the Sarcopterygian lineage (Table 3, Figure 5). The most ancestral forms are traced to a cluster of three *aqp2*-like genes encoded in the coelacanth genome between Fas apoptotic inhibitory molecule 2 (*faim2*) and Rac GTPase activating protein 1 (*racgap1*) (Figure 3A and Figure S14) with phylogenetically related *aqp2*-like orthologs (formerly *aqp0p*, [44]) in the ancient lineage of dipnoan lungfishes (Figure 2). The genomic arrangement of the coelacanth aquaporin cluster is highly reminiscent of the mammalian clusters first noted in humans [67]. However, although the primary structures of the three coelacanth paralogs have diverged significantly (33–40% amino acid substitution), the Bayesian analyses do not provide statistical support for a preferential orthology to either AQP2, -5, or -6. Nevertheless, comparison of the coelacanth and lungfish orthologs to the human primary structures revealed that the basal sarcopterygian channels are collectively more akin to AQP2 (56±3.7%), rather than AQP5 (54±1.5%), or AQP6 (47±1.3%), and we therefore annotated the paralogs as Aqp2a, -2b and -2c. To probe the basis for the reduced evolutionary rate of the coelacanth genes in relation to the amphibian cluster, we used Geneconv [68] to search for replicated nucleotide regions potentially resulting from gene conversion. No outer fragments are detected in either group, however, a significant 86 nucleotide (nt) inner fragment (nt 157–242: *aqp2a*; Bonferroni-corrected Karlin-Altschul Pvalue  = 0.00012) is identified in the coelacanth *aqp2a* and *-2c* transcripts. This segment codes for the C-terminal region of TMD2 and α-helix 3, a peptide that includes the first NPA motif.

**Table 3:** Prevalence of aquaporins identified in deuterostome animals.

		AQP																	∑	∑
	Animal	0	1	2	3^a^	4	5	6	7	8	9	10	11	12	13	14	15	16	classes	paralogues
Eutheria	Human	1	1	1	1	1	1	1	5^b^	1	1^c^	1	1	2					13	18
Metatheria	Tasmanian devil	1	1	1	1	1	1	1	1	1	1	1	1	1		1			14	14
Prototheria	Platypus	1	1	1	1	1	1	1	1	1	1	1	1	1	1	1			15	15
Aves	Zebra finch	1	1	1	1	1	1		1	1	1	1	1	1		1			13	13
Crocodylia	American alligator	1	1	1	1	1	1		1	1	1	1	1	1		1	1	1	15	15
Testudines	Chinese softshell turtle	1	1	1	1	1	1	1	1	1	1		1	1		1	1	1	15	15
Serpentes	Burmese python	1	1	1	1	1	2	1	1	1	1	1	1	1		1			14	15
Iguania	Green anole	1	1	1	1	1	2	1	2	1	1	1	1	1		1			14	16
Amphibia	Western clawed frog	1	1		1	3	2	2	1	1	1	1	1	1	1	1		1	15	19
Actinistia	Coelacanth	1	1	3	1	1			1	1	1	1	1	1		1	1		13	15
Acanthopterygii	Nile tilapia	2	3		2	2			1	2	2	2	2	1		1	1		12	21
Paracanthopterygii	Atlantic cod	2	2		2	2			1	3	2	2	2	1		1			11	20
Protacanthopterygii	Atlantic salmon	4	4		4	4			1	8	4	4	4	2		1	2		12	42
Ostariophysi	Zebrafish	2	2		2	1			1	4	2	2	1	1		1	1		12	20
Elopomorpha	Japanese eel	2	2		2	4			1	5	2	3	2	1		1	1		12	26
Holostei	Spotted gar	1	1		1	1				1	1	2	2	1		1	1		11	13
Chondrichthyes	Little skate	1	1		2	1					1	1	1	1		1	1		10	11
Hyperoartia	Sea lamprey	1^d^			2	1				1		2		1^e^		1			7	9
Tunicata	Vase tunicate				1	1				1									3	3
Cephalochordata	Amphioxus				2	2				5				2					4	11
Hemichordata	Acorn worm				1	5				1				2					4	9
Echinozoa	Purple sea urchin				3	5				3				2					4	13
Asterozoa	Bat star				2	4				2				2					4	10
Number of	exons	4	4	4	6	4	4	4	6	5-6	6	6	2–3	3–4	6	5–6	4–5	4–5		
Number of orthologs	examined	173	223	111	183	212	106	88	114	214	144	141	147	139	8	69	22	7		

^a^ Basal deuterostome glps are listed under AQP3.

^b^ In addition to a functional *AQP7*, humans encode four pseudogenes (*AQP7p1*, *-7p2*, *-7p3*, and *-7p4*).

^c^ Amongst eutherian genomes, chiropteran bats are an exception with at least 3 copies of *AQP9* encoded by 1, 5 or 6 exons.

^d^ The *aqp01* gene in Arctic lamprey is encoded with 5 exons.

^e^ This ortholog is not found in the current version of the sea lamprey genome (Pmar7), but fragments are present in the Arctic lamprey genome (LetJap1.0).

### Amphibia Utilise Ancestral Forms of AQP6 for Water Conservation

Closer examination of the *AQP2*, *-5* and *-6* gene loci in Tetrapoda (Figure 3A and Figure S14), reveals that they comprise diverse clusters of four tightly linked genes in the Western-clawed frog (*Xenopus tropicalis: AQP6ub-5L-5-6vs*), split sets of binary clusters (*AQP2-5* and *AQP5L-6*) in the green anole or a split binary cluster and singleton (*AQP2-5* and *AQP6*) in the Chinese softshell turtle (*Pelodiscus sinensis*), binary clusters (*AQP2-5*) in the archosaurian alligators and birds, and a ternary cluster (*AQP2-5-6*) in Mammalia. These observations also confirmed that canonical *AQP2* is not encoded in the genome of the Western-clawed frog [24] or its congener the African clawed frog. The Bayesian analyes show that the wholly aquatic Western-clawed frog has two paralogs of *AQP5*, a canonical form (*AQP5*, formerly annotated as *AQP-x5*, [28]) and a related paralog (*AQP5L*, formerly annotated as *AQP2*) encoded between two *AQP6* genes, the urinary bladder-type (*AQP6ub*) and the ventral skin type (*AQP6vs*) formerly identified as (h2-like and h3-like) type-a2 anuran specific aquaporins [23], [24], [32]. Amongst other Anura the Bayesian data provide statistical support for three paralogs of *AQP6* (see Table S2), the urinary bladder type (*AQP6ub*) and two paralogs (*AQP6vs1*, *-6vs2*) that mediate cutaneous hydration through the ventral skin of semi-aquatic and semi-terrestrial anurans [33], [35]. Canonical AQP2 (formerly annotated as AQP-h2k or HC-2, [25], [26]) is found in the genomes of Anura, including the arboreal Pacific treefrog (*Pseudacris regilla*) and the aquatic green frog (*Rana clamitans*), but the absence of an assembled linkage map currently precludes comparison of the loci.

### Loss and Functional Divergence of AQP6 in Archosauria and Mammalia

The heterogeneous lengths of the branches shown in Figure 2 illustrate the rapid evolutionary rate of the eutherian *AQP6* transcripts, which, based upon the human values, have respectively undergone 1.56- and 1.39-fold higher substitution rates compared to the amphibian *AQP6ub* and *AQP6vs* transcripts. The rate of divergence is approximately twice that of the human *AQP2* and *-5* channels, and 3.17-fold higher compared to the coelacanth *aqp2* orthologs. Part of this diversification includes the evolution of an extended N-terminal internalising domain (ENID: 10 amino acids in Laurasiatheria and Glires, 13 amino acids in Scandentia and Primates), which determines the intracellular localisation of AQP6 [69], [70], but also the divergence of the C-terminal regions including non-synonymous codon substitutions of putative phosphorylation sites (Table S5), which in AQP2 have been shown to be important for AVPR2-mediated intracellular trafficking (Moeller and Fenton 2012). The high levels of nucleotide exchange combined with genomic rearrangements may have resulted in the loss of *AQP6* in the Triassic ancestors of the Archosauria. Amongst fourteen avian and two crocodylian genomes, we located the *AQP2-5* binary clusters proximally linked to a second glucagon gene (*GCG2*), but we have not found *AQP6* in these lineages. By contrast we identified novel *AQP5*-like genes (*AQP5L*) in the genomes of Iguania (green anole) and Serpentes (Burmese python, *Python molurus bivittatus*, and king cobra, *Ophiophagus hannah*). Unlike the linkage map in the green anole, the novel king cobra gene is arranged in a ternary cluster (*AQP2-5-5L*) flanked by *FAIM2* and *GCG2*, while *AQP6* is found in a separate DNA fragment flanked by *RACGAP1* (Figure S21).

## Discussion

In the present study we examine the origin and diversification of deuterostome aquaporins, and show that the selective evolution of the major channels mediating water conservation in Amphibia, Sauropsida and Mammalia contrast the ubiquity of pattern forming genes that specify the limbs, fins, lungs or swimbladders of Eutelostomi. The gene clusters harbouring paralogous forms of *AQP2*, *-5* or *-6* are specific to Sarcopterygii, the lineage that gave rise to the terrestrial vertebrates. This facet implies that aquaporins played a pivotal role for terrestrial radiation.

**Figure 6:**
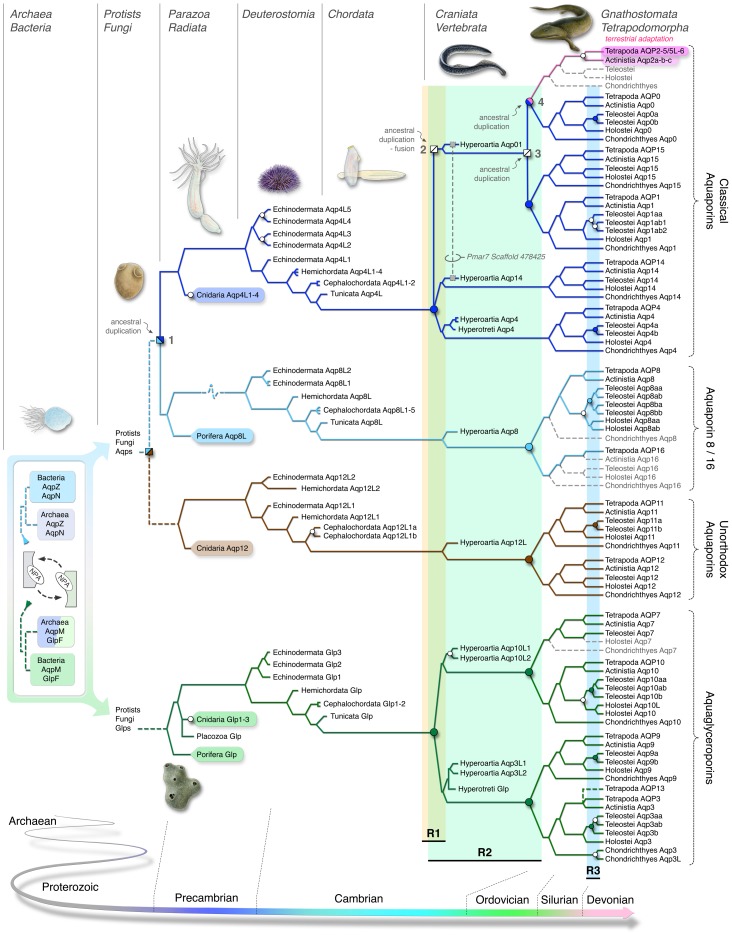
Evolutionary model and ancestry of the deuterostome aquaporin superfamily. Archaea and Bacteria are encapsulated in a putative “ring of life” scenario. Dotted lines indicate uncertain relationships. White nodes indicate tandem duplication, coloured nodes are associated with serial rounds (R1–R3) of whole genome duplication. The nomenclature and model are explained further in the text. The tetrapodomorph shown above node 4 is *Tiktaalik roseae*, which likely harboured the *aqp2-like* genes as shown for Actinistia and Dipnoi in Figure 2.

To understand the origin of the apomorphic aquaporin gene clusters in Sarcopterygii, we constructed a model that incorporates the putative duplication history of the superfamily (Figure 6). Due to the absence of phylogenetic convergence to a single common stem for the metazoan subfamilies, the model traces each grade back to the earliest forms. The primordial channel is thought to have arisen through intragenic duplication of a hemipore to form an integral membrane protein consisting of six TMDs, which when folded created a central pore partially restricted by the two opposing NPA domains [71]. This prototypical structure is now found in all domains of life and forms the basis of the analysed alignments. As in previous studies, the present data support an early split of the superfamily into two major branches: 1. aquaporins in which the central pore evolved a stringent selectivity for water transport, and 2. aquaglyceroporins facilitating the passage of both water and small uncharged solutes such as glycerol [55], [56], [58], [72], [73]. In contrast to previous reports, however, we find an unexpected diversity of integral membrane proteins in Archaea and Bacteria, with robust support for four major clades (see Text S1, Figure S5). Although we do not find more than one paralog in any given species of Archaea, some orders of Bacteria (Lactobacillales) encode multiple copies of GlpF in addition to AqpZ, while others (Bacillales) encode AqpN and AqpZ, and archaeal and bacterial genes are represented in each of the four clades. These novel observations are potentially consistent with the “ring of life” hypothesis involving horizontal gene transfer (HGT) and genome fusions prior to the evolution of Eukaryota [74], [75]. The subsequent endosymbiosis and operational flow of genes to the chromosomes of Eukaryota [76] may in part explain the broad diversity of aquaporins recently documented in single-celled organisms [56]. To date, however, the phylogenetic interrelations of aquaporins between single-celled and metazoan organisms remains mostly obscure, which, as noted in the present study, may be masked by the presence of endosymbiont genes. The current evidence indicates that Protists and Fungi encode both multiple copies of aquaglyeroporins, and a separate set of aquaporins more related to Aqp8 rather than Aqp4 or the unorthodox aquaporins.

Our previous analyses of the evolution of piscine aquaporins provided evidence for four major grades of integral membrane proteins in Vertebrata [36], [42], [43]. More recently this concept has been extended to plants [77], [78], however the origin of plant glycerol transporters, including NIPs and GIPs, may have occurred via HGT [55], [56], indicating that the gene flows are not coalescent. For basal metazoan organisms, we find putative evidence of HGT in Cnidaria, which lack a phylogenetically resolved branch for Aqp8. In this instance the functions of Aqp8 may have been co-opted by endosymbiotic zooanthellae or bacteria. Amongst Porifera the phylogenetic evidence supports the presence of an ancestral Aqp8-like channel in addition to an aquaglyceroporin, while the latter function may also have expanded via HGT or bacterial symbiosis. Thus, with the exception of an orthologous *aqp8* gene in Cnidaria, we successfully traced the four major grades of deuterostome aquaporin to the parazoan-eumetazoan divide, which is estimated to have occurred >1000 Ma [79].

It is well established that serial rounds of WGD played a major role in determining the genomic architecture of chordate animals [65], [80]–[83]. A surprising finding is thus the prevalence of aquaporin clusters encoded in nearly every lineage examined. We not only identify novel gene clusters in Radiata, Echinodermata and Cephalochordata, but find evidence of independent intrachromosomal duplications in Hyperoartia (*aqp01*, *-14*, *-10L1*, *-10L2*), Chondrichthyes (*aqp3*, *-3L*), Actinopterygii (*aqp1aa*, *-1ab1*, *-1ab2*, *-3aa*, *-3ab*, *-8aa*, *-8ab*, *-10aa*, *-10ab*) and Sarcopterygii (*aqp2a*, *-2b*, *-2c*, *-5*, *-5L*, *-6ub*, *-6vs*). Similar gene clusters have also been reported for Panarthropoda [84], [85]. This recurrent feature of aquaporin evolution indicates that the superfamily is not only prone to errors during DNA replication, but that positive selection pressure favours retention of the duplicates. For example, the recent analysis of the rainbow trout (*Oncorhynchus mykiss*) genome [86] revealed that approximately half of the protein-coding genes arising from an 88 - 103 Ma WGD in the salmonid lineage [87] are now fractionated, yet our anlysis of the aquaporin repertoire in tetraploid salmonids revealed that they retain twice the gene copy number compared to diploid Teleostei.

In an effort to separate the mechanism of gene expansion between tandem duplication and WGD, we combined broad taxon sampling with synteny. The model thus favours tandem duplication when the gene clusters are proximally linked in the DNA of a restricted subset of organisms, and WGD when the phylogenetic signal supports major branches separated at the level of Hyperoatia, Gnathostomata or Teleostei. The timing of WGD, however, remains an open topic, with recent studies suggesting that two rounds likely occurred before the divergence of ancestral lamprey and gnathostome lineages [88], [89]. If this is true then at least seven classes of aquaporin should have been lost from the genomes of the two species of lamprey studied. Such a scenario cannot be ruled out since genome reduction is a dominant mode of evolution [90], which in Vertebrata has been associated with multiple chromosomal fusions and rearrangements [91]–[93]. A further confounding feature is the discovery of genome remodelling in Hyperoartia, whereby ∼20% of germ line DNA is eliminated from many somatic cell lineages during embryonic development [94] Thus, although we identify seven classes of aquaporin in Hyperoartia, and show that at least seventeen classes exist in extant Gnathostomata, the timing of R2 remains an open question.

The application of the WGD theory is consistent with the evolution of at least three of the novel classes of vertebrate aquaporin reported here, including *aqp14*, *-15* and *-16*. The modular combination of WGD and tandem duplication also explains why there are eight *aqp8* genes in the tetraploid salmon, but only single orthologs in diploid Tetrapoda. The situation for the classical aquaporins is more complex. The model shown in Figure 6 predicts that *aqp4* and *aqp8* genes likely arose by duplication at node 1, and while the former *aqp4* grade of aquaporins expanded independently in Cnidaria, Echinodermata, and Protochordata, diversification into six subfamilies encompassing *aqp4*, *-14*, *-1*, *-15*, *-0* and *-2* did not occur until WGD began to shape the vertebrate genomes in the Cambrian. Assuming that R2 occurred after the divergence of ancestral lamprey and gnathostome lineages, we expected to find two classical aquaporins in the hyperoartian genomes that could explain the WGD origin of *aqp1*, *-15*, *-0* and *-2*. We initially found evidence that supported this scenario, but we subsequently experimentaly demonstrated that the *aqp1*-like and *aqp0*-like fragments are encoded by a single gene proximally linked to *aqp14*. Nevertheless the unorthodox exon structure coupled with the presence of a mobile element encoded between the two hemipores indicates that two ancestral genes arose at node 2, and subsequently fused in lampreys leaving the *aqp01* chimaera and a ghost of a duplication at node 3. Fusion genes are well documented in *Drosphila* [95] and are recognised as a major source of oncogenes in humans [96]. In lampreys we speculate that the generation of such gene chimaeras may also be a consequence of the programmed genome rearrangements during embryogenesis [97]. While studies of hagfishes (Hyperotreti) may shed light on the origin of the chimaeric gene, the novel observation that an *aqp0*-like water channel is expressed in the sea lamprey eye is consistant with the evolution of multifocal lenses in Hyperoartia after the lineage diverged from Hyperotreti [98].

It has previously been suggested that duplication of *aqp0* in Teleostei and subsequent expansion via tandem duplication could explain the origin of the amphibian *AQP2*, *-5* and *-a2* genes [24]. The underlying rationale for this suggestion was the location of *aqp0* between *faim2* and *racgap1* in the medaka (*Oryzias latipes*), green-spotted pufferfish (*Tetraodon nigroviridis*) and human genomes. While certainly a plausible scenario, it does not account for the theory of WGD and rediploidisation associated with chromosomal fusions and rearrangements [90]–[93]. Nevertheless, in favour of the tandem duplication theory is the ancient linkage of the three coelacanth *aqp2*-like genes with *faim2*, *racgap1* and *aqp0*, and the absence of either *aqp2*, *-5* or *-6* in the available genomes of Chondrichthyes and Actinopterygii. On the contrary, in favour of a WGD origin of the apomorphic genes in Sarcopterygii is the new evidence that Gnathostomata encode the novel *aqp15* subfamily in addition to the *aqp1* and *aqp0* subfamilies, while the phylogenetic analyses of syntenic nuclear receptors revealed that lineage-level loss of gene subfamilies is common during vertebrate evolution. Indeed an earlier study examining the phylogenetic relationships of the amphibian *CAR* genes proposed that this xenosensor arose through WGD, but was subsequently lost in the fish lineage [99]. In the present study we further show parallels between the lineage-level loss the *NR1D4* and *RORD* subfamilies and *AQP14* in Eutheria. The model thus posits that the basal sarcopterygian *aqp2*-like genes may equally well have arisen via WGD at node 4, but were subsequently lost in Actinopterygii. In any event the new data presented here reveal that the genomic apomorphy existed prior to the divergence of the actinistian and lissamphibian lineages >430 Ma [100], and may have arisen as early as the Cambrian. This timing predates the oldest Devonian tetrapodomorph fossil *Tiktaalik rosiae* [101], [102] and the earliest evidence of tetrapod-like trackways [103] by ∼35–135 million years.

The reduced rate of nucleotide substitution of the coleacanth *aqp2*-like transcripts is consistant with the significantly slower rate of protein evolution in this organism [104]. The present findings indicate that gene conversion may have been a contributing factor to such morphological retardation. By contrast we find that the *AQP2*, *-5* and particularly *-6* transcripts show an accelerated rate of divergence in Tetrapoda compared to the actinistian forms. The Bayesian and syntenic analyses show that the previously identified AQPa2 anuran-specific paralogs (AQP-h2 and -h3, [23], [24], [30], [34], [35]) are ancestral forms of *AQP6* encoded at the outer edges of the genomic retro-cluster. These observations suggest that the ancestral function and regulation of AQP6 was closely related to that of amphibian AQP2, whereby neurohypophysial control of vasotocin-like receptors facilitated the transepithelial uptake of water through the ventral skin of Amphibia and the recycling of water from the urinary bladder [25], [30]–[35]. The conservation of the amphibian AQP2, -5, -5L and -6 C-terminal ‘S256’ P1 residues that are recognised phosphorylation sites for AQP2-AVPR2-mediated trafficking [105] further suggests that the proteins have retained ancestral properties. However, this changed in the Sauropsida and Mammalia. In Squamata, the gene cluster is differentially split in Iguania and Serpentes, although both groups retain the novel *AQP5L* gene. The Archosauria appear to have lost *AQP6* leaving only *AQP2-5* linked to *GCG2* and the conserved flanking genes, a feature that may be related to the evolution of a uric acid habitus and loss of the urinary bladder [17], [18], [106]. In Mammalia only one *AQP6* is retained, which unlike *AQP14*, survived extinction associated with the rapid rate of nucleotide substitution, and neofunctionalised. Mammalian AQP6 is localized in the intracellular vesicles of the glomerular podocyte cell bodies and foot processes, and the intercalating cells in the outer and inner medullary collecting ducts [69]. It has diminished water permeability properties that are only activated at low pH, and it is thus considered a vacuolar-type water channel that contributes to acid-base balance, tubular endocytosis, glomerular filtration, and ion (NO_3_^−^, Cl^−^) transport [29], [107], [108]. The novel functions of mammalian AQP6 and loss in the avian lineage are consistent with the absence of water uptake via the skin or recycling through urinary bladders, and potentially reflects the selection pressure leading to more efficient AQP2-mediated antidiuresis in these clades.

The adaptive function of AQP5 appears to be associated with its glandular localization. In Anura it is expressed in mucus cells and small granular glands of the skin, contributing to the maintenance of moist skin, cutaneous gas exchange and thermoregulation [24], [28]. In Mammalia it is expressed in the lung and sweat glands where, in the latter instance, it also contributes to evaporative heat-loss associated with endothermy [29]. In Sauropsida, AQP5 is expressed in the venom gland of snakes [109], and the nasal salt-secreting glands of birds [22], a related organ to the lingual glands of salt-water crocodiles [19], [21], [110]. Although freshwater alligators lack the lingual salt glands, all Crocodylia shed tears through their lacrimal and tongue-like Harderian glands [106]. Both of these latter glands are specific to the Tetrapoda with the lacrimal appearing secondarily in the Amniota and eventually superceding the porphyin and lipid-secreting mammalian Harderian gland in adult Primates [111]. The expression of AQP5 in lacrimal glands is well documented [29], [112], and the early role of the two types of gland in terrestrial adaptation is associated with hydration of the cornea and the nictitating membrane [113].

The water-conserving role of AQP2 is now well documented in the renal collecting ducts of each of the three extant clades of Tetrapoda, but its evolutionary significance becomes manifest in humans with nephrogenic diabetes insipidus. This hereditary or acquired disease is caused by mutations in AQP2 or the AVPR2 receptor and results in the excretion of large volumes of dilute urine [114]–[116], a rapidly dehydrating syndrome that reflects the osmoregulatory modus operandi of freshwater fish. Indeed polyuria is the ancestral mechanism by which freshwater fish including Hyperoartia, Chondrichthyes and Actinopterygii, maintain their hyperosomotic condition in the face of substantial osmotic gradients (∼240–340: 1 mOsm, [14], [117]–[119]. We therefore suggest that it is the lack of equivalent homeostatic mechanisms involving the AVPR2-AQP2, -5 or -6 systems that has precluded actinopterygian fishes from making the permanent transition from water to land. By contrast, evidence showing that the most basal ichthyan Sarcopterygii (Actinistia and Dipnoi) encode Aqp2-like channels is consistent with observations that estivating lungfishes activate this channel via a similar system to the AVT-AVPR2-AQP2 axis of the mammalian kidney [44] and that this axis is mediated by the sarcopterygian-specific steroid aldosterone [120] to facilitate water conservation in the terrestrial environment.

Although the functional divergence of the apomorphic aquaporin genes took many millions of years before water conservation systems were sufficiently evolved in Tetrapoda to allow permanent habitation of the terrestial environment, it was nevertheless possible due to the fact that they existed in their genomes. In other words, the ability to adapt to novel environments is rooted in the genetic makeup of an organism. This can therefore be considered as genomic competence, a permissive condition that may be modulated through Darwinian selection. Unlike the ubiquitous transcription factors involved in limb and lung development, we propose that the selective evolution of water conserving aquaporins in the sarcopterygian lineage represents a permissive adaptation that facilitated tetrapod terrestrial adaptation.

## Supporting Information

Figure S1 **Phylogenetic interrelationships of deuterostome animals for which genomic complements of aquaporins were studied.** The number of taxa within each group are shown to the right. Square nodes indicate whole genome duplication. Circular dots on terminal branches indicate an assembled linkage map.
(PDF)

Figure S2 **Annotated Bayesian majority rule consensus tree of the basal deuterostome aquaporin superfamily.** The tree is mid-point rooted. Posterior probabilities resulting from analyses of the codon/amino acid alignments are shown at each node, with the scale bar indicating the rate of substitutions per site. Four major grades of aquaporin, Aqp4-like, Aqp8-like, Glp and Aqp12-like are respectively shaded in blue, cyan, green and orange with paralogues of a given subfamily highlighted and numerically labeled.
(PDF)

Figure S3 **Annotated Bayesian majority rule consensus tree of Parazoa-Radiata aquaporins.** The tree is rooted with *aqpM*. Posterior probabilities resulting from analyses of the codon/amino acid alignments are shown at each node, with the scale bar indicating the rate of substitutions per site. Four major grades of aquaporin Aqp4-like, Aqp8-like, Aqp12-like and Glp are respectively shaded in blue, cyan, orange and green with paralogues of a given subfamily highlighted and numerically labeled. Putative endosymbiont aquaporin and GlpF orthologs are respectively shaded in grey and magenta. Tandemly arranged paralogs are shown as numerically linked circles.
(PDF)

Figure S4 **Summarised Bayesian tree of the Parazoa-Cnidaria-Basal deuterostome aquaporins.** The tree is rooted with *aqpM*. Posterior probabilities resulting from analyses of the codon/amino acid alignments are shown at each node, with the scale bar indicating the rate of substitutions per site.
(PDF)

Figure S5 **Annotated Bayesian majority rule consensus tree of archaeal and bacterial aquaporins.** The tree is mid-point rooted. Posterior probabilities resulting from analyses of the codon/amino acid alignments are shown at each node, with the scale bar indicating the rate of substitutions per site. Four major grades of Bacterial and Archaean aquaporins are shaded in colour, with Archaean orthologs highlighted in pink to show the polyphyletic clustering of Archaean AqpZ orthologs.
(PDF)

Figure S6 **Annotated Bayesian majority rule consensus tree of deuetrostome aquaglyceroporins.** The tree is rooted with *Hydra vulgaris* Glp2. Posterior probabilities resulting from analyses of the codon alignments are shown at each node. Evolutionary older nodes associated with Basal Deuterostomia, Cyclostomata, Chondrichthyes, Holostei and Actinisia are respectively shaded in peach, yellow, grey, cyan and magenta. Teleost and tetrapod subclusters are shaded in green.
(PDF)

Figure S7 **Syntenic alignment of** ***AQP13*** **.** Genomic regions (Mb) are given in parentheses. The coding direction is indicated by the pointed end of the gene symbol.
(PDF)

Figure S8 **Annotated Bayesian majority rule consensus tree of deuterostome unorthodox aquaporin proteins.** The tree is rooted with starlet sea anemone Aqp12. Posterior probabilities resulting from analyses of the codon/amino acid alignments are shown at each node, with the scale bar indicating the rate of substitutions per site. Gnathostome Aqp12 and Aqp11 paralogs are respectively shaded cyan, and light orange. Evolutionary older nodes associated with Cyclostomata and basal Deuterostomia are respectively shaded in orange and grey. Nodes consistent with whole genome duplications (R2, R3) are labelled.
(PDF)

Figure S9 **Annotated Bayesian majority rule consensus tree of the deuterostome aquaporin 8 grade.** The tree is rooted with bacterial *aqpZ*. Posterior probabilities resulting from analyses of the codon/amino acid alignments are shown at each node, with the scale bar indicating the rate of substitutions per site. The teleost *aqp8aa* and *-8ab* tandem duplicates are shaded in green and the teleost *aqp8ba* and *-8bb* genomic duplicates are shaded in yellow. Evolutionary older nodes associated with Cyclostomata and basal Deuterostomia are respectively shaded in orange and grey, while tetrapod AQP16 orthologs are shaded in pink. Nodes consistent with whole genome duplications (R2, R3) are labelled. A schematic of the proposed evolution of actinopterygian *aqp8* tandem duplicates and genome duplicates is shown to the right.
(PDF)

Figure S10 **Syntenic alignment of** ***aqp8*** **genes in Actinopterygii.** Genomic regions (Mb) are given in parentheses with circular arrows indicating that the region is flipped in relation to orthologous regions. Coding direction is indicated by the pointed end of the gene symbol. Genes that are previously isolated and cloned [36], [121] are shown as blue symbols (**A**) Data for actinopterygian *aqp8aa* and *-8ab* paralogs. (**B**) Data for actinopterygian *aqp8ba* and *-8bb* paralogs. The zebrafish segmental duplication is boxed in green.
(PDF)

Figure S11 **Annotated Bayesian majority rule consensus tree of deuterostome classical aquaporins.** The tree is rooted with *Nematostella vectensis aqp4L1*. Posterior probabilities resulting from analyses of the codon alignments are shown at each node. Nodes consistent with whole genome duplications are labelled with black circles, and nodes consistent with tandem duplications are labeled with black diamonds. Evolutionary older nodes associated with Cyclostomata, Chondrichthyes, Holostei and Actinisia are respectively shaded in yellow, grey, cyan and magenta. Teleost and tetrapod subclusters are shaded in light blue for AQP0, -1, -15, -4 and -14, and pink for AQP2, -5-, 5-like (5L) and -6.
(PDF)

Figure S12 **Syntenic alignment of** ***AQP15*** **.** Genomic regions (Mb) are given in parentheses. The coding direction is indicated by the pointed end of the gene symbol.
(PDF)

Figure S13 **PCR products amplified from Chondrichthyes and Hyperoartia using primers for** ***aqp0*** **.** Representative gel image of RT-PCR analysis using primers flanking the NPA motifs of *aqp0* cDNA for the smaller-spotted catshark (*Scyliorhinus canicula*). Lanes 1-4 are the DNA ladder, and PCR products amplified from the eye mRNA of the smaller-spotted catshark, sea lamprey (*Petromyzon marinus*) and brook lamprey (*Lampetra planeri*), respectively.
(PDF)

Figure S14 **Chromosomal loci and synteny of chordate aquaporins.** (**A**) Linkage groups are drawn to scale within species showing approximate locations of the aquaporin superfamily in each organism. HOX-cluster-bearing chromosomes are coloured according to the key including teleost “a” and “b” duplicated clusters. For organisms without an assembled genome, the presence of a given paralog is annotated with a tick. (B) Syntenic arrangement of vertebrate *aqp0*, *-2*, *-5*, *-5L*, *-6* and *-14* genes. Genes are drawn in accordance with their contiguous coding in each genome, with dashed linker lines illustrating rearrangements between the lineages. Nuclear receptors analysed via Bayesian protocols are shown in red, with † indicating extinction of the gene. The *AQP2*, *-5*, *-5L* and *-6* gene clusters annotated with pink gene symbols are outlined in light grey to illustrate conservation in Mammalia, but split clusters in Sauropsida. Proximally linked *FAIM2* and *RACGAP1* are annoted in blue. Circular arrows indicate that the genomic region is flipped.
(PDF)

Figure S15 ***aqp0*** **linkage group maps to the human karyotype.** The linkage group of each organism is mapped using the karyotype view available in Genomicus. The human karyotype is scaled according to the gene copy number (GCN). Boxed numbers represent estimates of the lineage divergence times in millions of years before present (Ma) after Hedges and Kumar [100].
(PDF)

Figure S16 **Annotated Bayesian majority rule consensus tree of deuterostome retinoic acid receptors.** The tree is mid-point rooted. Posterior probabilities resulting from analyses of the codon/amino acid alignments are shown at each node, with the scale bar indicating the rate of substitutions per site. Gnathostome Rara, Rarb and Rarg paralogs are respectively shaded cyan, green and magenta. Evolutionary older nodes associated with Cyclostomata and basal Deuterostomia are respectively shaded in orange and grey. Nodes consistent with whole genome duplications (R1, R2, R3) are labelled with black circles. Receptors that are syntenic with aquaporins are indicated with coloured squares.
(PDF)

Figure S17 **Annotated Bayesian majority rule consensus tree of the deuterostome Nr1d family of nuclear receptors.** The tree is rooted with yellow-fever mosquito sevenup (*svp*). Gnathostome Nr1d1, Nr1d2 and Euteleostomi Nr1d4 paralogs are respectively shaded cyan, green and magenta. Evolutionary older nodes associated with Cyclostomata and basal Deuterostomia are respectively shaded in orange and grey. Labels and annotations are as for Supplementary Figure S16.
(PDF)

Figure S18 **Annotated Bayesian majority rule consensus tree of deuterostome constitutive androgen (CAR), pregnane-X (PXR) and vitamin D (VDR) receptors.** The tree is rooted with yellow-fever mosquito *svp*. Sarcopterygian Car, and gnathostome Pxr and Vdr paralogs are respectively shaded cyan, green and magenta. Evolutionary older nodes associated with Cyclostomata and basal Deuterostomia are respectively shaded in orange and grey. Labels and annotations are as for Supplementary Figure S16.
(PDF)

Figure S19 **Annotated Bayesian majority rule consensus tree of the deuterostome Nr4A family of nuclear receptors.** The tree is rooted with yellow-fever mosquito *svp*. Gnathostome Nr4a1, Nr4a2 and Nr4a3 paralogs are respectively shaded cyan, green and magenta. Evolutionary older nodes associated with Cyclostomata and basal Deuterostomia are respectively shaded in orange and grey. Labels and annotations are as for Supplementary Figure S16.
(PDF)

Figure S20 **Annotated Bayesian majority rule consensus tree of deuterostome retinoic-related orphan receptors (Ror).** The tetraparalogous topology of gnathostome Rors is characterised here for the first time, with Rord identified in Actinopteryii and all extant sarcopterygian lineages except Eutheria. The tree is rooted with yellow-fever mosquito *svp*. Gnathostome Rora, Rorc, Rorb and Rord paralogs are respectively shaded cyan, red, green and magenta. Evolutionary older nodes associated with Cyclostomata and basal Deuterostomia are respectively shaded in orange and grey. Labels and annotations are as for Supplementary Figure S16.
(PDF)

Figure S21 **Genomic arrangements of the king cobra** ***AQP2*** **,** ***-5*** **,** ***-5L*** **and** ***-6*** **genes.** Exons are labeled in accordance with the coding direction of each gene. Circular arrow indicates that the genomic region is flipped.
(PDF)

Table S1 **Primer sequences and PCR conditions for cloning of aquaporin cDNAs.**
(PDF)

Table S2 **List of aquaporin accession numbers used in the study.**
(PDF)

Table S3 **List of nuclear receptor accession numbers used in the study.**
(PDF)

Table S4 **Conservation of residues that putatively determine channel transport selectivities of aquaporins and aquaglyceroporins in Deuterostomia.**
(PDF)

Table S5 **Conservation of AQP2-S256 (P1), S261 (P2) in Sarcopterygii.**
(PDF)

Text S1 **Additional text concerning the unexpected diversity of prokaryotic and basal metazoan aquaporins, the broader repertoire of aquaglyceroporins and the ubiquity of unorthodox aquaporins in Vertebrata, and the observation of eight Aqp8s in tetraploid teleostei vs one and a novel subfamily in diploid Tetrapoda.**
(DOC)

## References

[1] Grosberg RK, Vermeij GJ, Wainwright PC. Biodiversity in water and on land. Curr Biol. 2012;22:R900–R903. 10.1016/j.cub.2012.09.050 23137680

[2] Carroll RL. The origin and early radiation of terrestrial vertebrates. J Paleontol. 2001;75:1202–1213.

[3] Shubin N, Tabin C, Carroll S. Fossils, genes and the evolution of animal limbs. Nature. 1997;388:639–648. 10.1038/41710 9262397

[4] Ruvinsky I, Gibson-Brown JJ. Genetic and developmental bases of serial homology in vertebrate limb evolution. Development. 2000;127:5233–5244. 11076746 10.1242/dev.127.24.5233

[5] Zhao XL, Sirbu IO, Mlc FA, Molotkova N, Molotkov A, et al Retinoic acid promotes limb induction through effects on body axis extension but is unnecessary for limb patterning. Curr Biol. 2009;19:1050–1057. 19464179 10.1016/j.cub.2009.04.059 PMC2701469

[6] Zhang J, Wagh P, Guay D, Sanchez-Pulido L, Padhi BK, et al Loss of fish actinotrichia proteins and the fin-to-limb transition. Nature. 2010;466:234–U106. 20574421 10.1038/nature09137

[7] Freitas R, Gomez-Marin C, Mark WJ, Casares F, Gómez-Skarmeta JL. Hoxd13 Contribution to the Evolution of Vertebrate Appendages. Dev Cell. 2012;23:1219–1229. 23237954 10.1016/j.devcel.2012.10.015

[8] Schneider I, Shubin NH. Making Limbs from Fins. Dev Cell. 2012;23:1121–1122. 23237946 10.1016/j.devcel.2012.11.011

[9] Morris C. The origin orf lungs, a chapter in evolution. American Naturalist. 1892;26:975–986.

[10] Daniels CB, Orgeig S, Sullivan LC, Ling N, Bennett MB, et al The origin and evolution of the surfactant system in fish: Insights into the evolution of lungs and swim bladders. Physiol Biochem Zool. 2004;77:732–749. 15547792 10.1086/422058

[11] Cass AN, Servetnick MD, McCune AR. Expression of a lung developmental cassette in the adult and developing zebrafish swimbladder. Evol Dev. 2013;15:119–132. 25098637 10.1111/ede.12022

[12] Graham JB. Air-Breathing Fishes. Evolution Diversity and Adaptation. New York: Academic Press. 1997;299 p.

[13] Bernard C. Leçons sur les propriétés physiologique et les alterations pathologique des liquids de l'organsime. France: Baillère. 1859.

[14] Schmidt-Nielsen K. Animal Physiology: Adaptation and Environment. Cambridge: Cambridge University Press. 1997.

[15] Lau KK, Yang Y, Cook GA, Wyatt RJ, Nishimura H. Control of aquaporin 2 expression in collecting ducts of quail kidneys. Gen Comp Endocr. 2009;160:288–294. 19135443 10.1016/j.ygcen.2008.12.007

[16] Nishimura H, Fan Z. Sodium and water transport and urine concentration in avian kidney. Symp Soc Exp Biol. 2002;54:129–151. 14992149

[17] Smith HW. From fish to philosopher. The story of our internal environment. USA: CIBA Pharmaceutical Products Ltd. 1953.

[18] Nishimura H. Urine concentration and avian aquaporin water channels. Pflug Arch Eur J Phy. 2008;456:755–768. 10.1007/s00424-008-0469-6 18278509

[19] Schmidt-Nielsen K, Fänge R. Salt glands in marine reptiles. Nature. 1958;182:783–785.

[20] Shuttleworth TJ, Hildebrandt JP. Vertebrate salt glands: Short- and long-term regulation of function. J Exp Zool. 1999;283:689–701. 10222591 10.1002/(sici)1097-010x(19990601)283:7<689::aid-jez7>3.0.co;2-t

[21] Cramp RL, Meyer EA, Sparks N, Franklin CE. Functional and morphological plasticity of crocodile (*Crocodylus porosus*) salt glands. J Exp Biol. 2008;211:1482–1489. 18424682 10.1242/jeb.015636

[22] Müller C, Sendler M, Hildebrandt JP. Downregulation of aquaporins 1 and 5 in nasal gland by osmotic stress in ducklings, *Anas platyrhynchos*: implications for the production of hypertonic fluid. J Exp Biol. 2006;209:4067–4076. 17023601 10.1242/jeb.02491

[23] Suzuki M, Ogushi THY, Tanaka S. Amphibian aquaporins and adaptation to terrestrial environments: A review. Comp Biochem Physiol A-Mol Integr Physiol. 2007;148:72–81. 17270476 10.1016/j.cbpa.2006.12.021

[24] Suzuki M, Tanaka S. Molecular and cellular regulation of water homeostasis in anuran amphibians by aquaporins. Comp Biochem Phys. 2009;153A:231–241. 10.1016/j.cbpa.2009.02.035 19268556

[25] Ogushi Y, Mochida H, Nakakura T, Suzuki M, Tanaka S. Immunocytochemical and phylogenetic analyses of an arginine vasotocin-dependent aquaporin, AQP-h2K, specifically expressed in the kidney of the tree frog, *Hyla japonica* . Endocrinology. 2007;148:5891–5901. 17872371 10.1210/en.2007-0613

[26] Pandey RN, Yaganti S, Coffey S, Frisbie J, Alnajjar K, et al Expression and immunolocalization of aquaporins HC-1,-2, and-3 in Cope's gray treefrog, Hyla chrysoscelis. Comp Biochem Physiol A-Mol Integr Physiol. 2010;157:86–94. 20416391 10.1016/j.cbpa.2010.04.007

[27] Gwee PC, Tay BH, Brenner S, Venkatesh B. Characterization of the neurohypophysial hormone gene loci in elephant shark and the Japanese lamprey: Origin of the vertebrate neurohypophysial hormone genes. 2008; BMC Evol Biol 9: ARTN 47. 10.1186/1471-2148-9-47 PMC2656470 19243634

[28] Kubota M, Hasegawa T, Nakakura T, Tanii H, Suzuki M, et al Molecular and cellular characterization of a new aquaporin, AQP-x5, specifically expressed in the small granular glands of *Xenopus* skin. J Exp Biol. 2006;209:3199–3208. 16888067 10.1242/jeb.02351

[29] Takata K, Matsuzaki T, Tajika Y. Aquaporins: water channel proteins of the cell membrane. Prog Histochem Cyto. 2004;39:1–83. 10.1016/j.proghi.2004.03.001 15242101

[30] Hasegawa T, Tanii H, Suzuki M, Tanaka S. Regulation of water absorption in the frog skins by two vasotocin-dependent water-channel aquaporins, AQP-h2 and AQP-h3. Endocrinology. 2003;144:4087–4096. 12933683 10.1210/en.2003-0418

[31] Hasegawa T, Suzuki M, Tanaka S. Immunocytochemical studies on translocation of phosphorylated aquaporin-h2 protein in granular cells of the frog urinary bladder before and after stimulation with vasotocin. Cell Tissue Res. 2005;322:407–415. 16047161 10.1007/s00441-005-0037-8

[32] Ogushi Y, Tsuzuki A, Sato M, Mochida H, Okada R, et al The water-absorption region of ventral skin of several semiterrestrial and aquatic anuran amphibians identified by aquaporins. Am J Physiol-Regul Integr Comp Physiol. 2010;299:R1150–R1162. 20811008 10.1152/ajpregu.00320.2010

[33] Ogushi Y, Kitagawa D, Hasegawa T, Suzuki M, Tanaka S. Correlation between aquaporin and water permeability in response to vasotocin, hydrin and beta-adrenergic effectors in the ventral pelvic skin of the tree frog Hyla japonica. J Exp Biol. 2010;213:288–294. 20038663 10.1242/jeb.036871

[34] Shibata Y, Takeuchi H-, Hasegawa T, Suzuki M, Tanaka S, et al Localization of Water Channels in the Skin of Two Species of Desert Toads, *Anaxyrus (Bufo) punctatus* and *Incilius (Bufo) alvarius* . Zool Sci. 2011;28:664–670. 21882955 10.2108/zsj.28.664

[35] Saitoh Y, Ogushi Y, Okada, Tanaka S, Suzuki MR, et al Novel vasotocin-regulated aquaporins expressed in the ventral skin of semiaquatic anuran amphibians: Evolution of cutaneous water-absorbing mechanisms. General Endocrinology. 2014;155:2166–2177. 10.1210/en.2013-1928 24654785

[36] Tingaud-Sequeira A, Calusinska M, Chauvigné F, Lozano J, Finn RN, et al The zebrafish genome encodes the largest vertebrate repertoire of functional aquaporins with dual parology and substrate specificities similar to tetrapods. BMC Evol Biol. 2010;10:38. 20149227 10.1186/1471-2148-10-38 PMC2829555

[37] Sweet G, Gandor C, Voegele R, Wittekindt N, Beuerle J, et al Glycerol facilitator of *Escherichia coli*: cloning of *glpF* and identification of the *glpF* product. J Bacteriol. 1990;172:424–430. 2152911 10.1128/jb.172.1.424-430.1990 PMC208448

[38] Preston GM, Carroll TP, Guggino WB, Agre P. Appearance of water channels in *Xenopus* oocytes expressing red cell CHIP28 protein. Science. 1992;256:385–387. 1373524 10.1126/science.256.5055.385

[39] Jahn TP, Moller ALB, Zeuthen T, Holm LM, Klaerke DA, et al Aquaporin homologues in plants and mammals transport ammonia. FEBS Lett. 2004;574:31–36. 15358535 10.1016/j.febslet.2004.08.004

[40] Wu B, Beitz E. Aquaporins with selectivity for unconventional permeants. Cell Mol Life Sci. 2007;64:2413–2421. 17571212 10.1007/s00018-007-7163-2 PMC11138416

[41] Hamdi M, Sanchez MA, Beene LC, Liu Q, Landfear SM, et al Arsenic transport by zebrafish aquaglyceroporins. BMC Mol Biol. 2009;10:104. 19939263 10.1186/1471-2199-10-104 PMC2788550

[42] Cerdà J, Finn RN. Piscine Aquaporins: An Overview of Recent Advances. J Exp Zool. 2010;313A:623–650. 10.1002/jez.634 20717996

[43] Finn RN, Cerdà J. Aquaporin evolution in fishes. Front Physiol. 2011;2:44. 21886623 10.3389/fphys.2011.00044 PMC3145251

[44] Konno N, Hyodo S, Yamaguchi Y, Matsuda K, Uchiyama M. Vasotocin/V2-Type Receptor/Aquaporin Axis Exists in African Lungfish Kidney but Is Functional Only in Terrestrial Condition. Endocrinology. 2010;151:1089–1096. 20147523 10.1210/en.2009-1070

[45] Cutler CP, Cramb G. Differential expression of absorptive intestinal and renal tissues of the cation-chloride-cotransporters in the European eel (*Anguilla anguilla*). Comp Biochem Physiol B-Biochem Mol Biol. 2008;149:63–73. 17950642 10.1016/j.cbpb.2007.08.007

[46] Notredame C, Higgins DG, Heringa J. T-Coffee: A novel method for fast and accurate multiple sequence alignment. J Mol Biol. 2000;302:205–217. 10964570 10.1006/jmbi.2000.4042

[47] Katoh K, Toh H. Recent developments in the MAFFT multiple sequence alignment program. Brief Bioinform. 2008;9:286–298. 18372315 10.1093/bib/bbn013

[48] Suyama M, Torrents D, Bork P. PAL2NAL: robust conversion of protein sequence alignments into the corresponding codon alignments. Nucleic Acids Res. 2006;34:W609–W612. 16845082 10.1093/nar/gkl315 PMC1538804

[49] Ronquist F, Huelsenbeck JP. MrBayes 3: Bayesian phylogenetic inference under mixed models. Bioinformatics. 2003;19:1572–1574. 12912839 10.1093/bioinformatics/btg180

[50] Swafford DL. PAUP*. Phylogenetic analysis using parsimony (* and other models). Version 4.0b10 for macintosh.2002; (www.sinauer.com/detail.php?id=8060). Sunderland, MASS: Sinauer Associates Inc.

[51] Finn RN, Kristoffersen BA. Vertebrate vitellogenin gene duplication in relation to the “3R hypothesis”: Correlation to the pelagic egg and the oceanic radiation of teleosts. PLoS ONE. 2007;2:e169. 17245445 10.1371/journal.pone.0000169 PMC1770952

[52] Zapater C, Chauvigné F, Fernandez-Gomez B, Finn RN, Cerdà J. Alternative splicing of the nuclear progestin receptor in a perciform teleost generates novel mechanisms of dominant-negative transcriptional regulation. Gen Comp Endocrinol. 2013;182:24–40. 23220040 10.1016/j.ygcen.2012.11.015

[53] Han MV, Zmasek CM. phyloXML: XML for evolutionary biology and comparative genomics. BMC Bioinformatics 2009;10. 10.1186/1471-2105-10-356 PMC2774328 19860910

[54] Hedges SB, Kumar S. The timetree of life. 2009; New York: Oxford University Press.

[55] Zardoya R. Phylogeny and evolution of the major intrinsic protein family. Biol Cell. 2005;97:397–414. 15850454 10.1042/BC20040134

[56] Abascal F, Irisarri I, Zardoya R. Diversity and evolution of membrane intrinsic proteins. Biochim Biophys Acta-Gen Subj. 2014;1840:1468–1481. 10.1016/j.bbagen.2013.12.001 24355433

[57] Zapater C, Chauvigné F, Norberg B, Finn RN, Cerdà J. Dual Neofunctionalization of a Rapidly Evolving Aquaporin-1 Paralog Resulted in Constrained and Relaxed Traits Controlling Channel Function during Meiosis Resumption in Teleosts. Mol Biol Evol. 2011;28:3151–3169. 21653921 10.1093/molbev/msr146

[58] King LS, Kozono D, Agre P. From structure to disease: The evolving tale of aquaporin biology. Nat Rev Mol Cell Bio. 2004;5:687–698. 15340377 10.1038/nrm1469

[59] Fu DX, Libson A, Miercke LJW, Weitzman C, Nollert P, et al Structure of a glycerol-conducting channel and the basis for its selectivity. Science. 2000;290:481–486. 11039922 10.1126/science.290.5491.481

[60] de Groot BL, Grubmuller H. The dynamics and energetics of water permeation and proton exclusion in aquaporins. Curr Opin Struc Biol. 2005;15:176–183. 10.1016/j.sbi.2005.02.003 15837176

[61] Froger A, Tallur B, Thomas D, Delamarche C. Prediction of functional residues in water channels and related proteins. Protein Sci. 1998;7:1458–1468. 9655351 10.1002/pro.5560070623 PMC2144022

[62] Amores A, Force A, Yan YL, Joly L, Amemiya C, et al Zebrafish *hox* clusters and vertebrate genome evolution. Science. 1998;282:1711–1714. 9831563 10.1126/science.282.5394.1711

[63] Jaillon O, Aury JM, Brunet F, Petit JL, Stange-Thomann N, et al Genome duplication in the teleost fish Tetraodon nigroviridis reveals the early vertebrate proto-karyotype. Nature. 2004;431:946–957. 15496914 10.1038/nature03025

[64] Amemiya CT, Powers TP, Prohaska SJ, Grimwood J, Schmutz J, et al Complete HOX cluster characterization of the coelacanth provides further evidence for slow evolution of its genome. Proc Natl Acad Sci USA. 2010;107:3622–3627. 20139301 10.1073/pnas.0914312107 PMC2840454

[65] Braasch I, Postlethwaite JH. Polyploidy in Fish and the Teleost Genome Duplication. In: Soltis PS, Soltis DEeditors. Polyploidy and Genome Evolution. Berlin: Springer-Verlag. 2012;pp. 341–383.

[66] Garcia HE, Laudet V. Nuclear receptors are markers of animal genome evolution. J Struct Funct Genomics. 2003;3:177–184. 12836696

[67] Ma TH, Yang BX, Umenishi F, Verkman AS. Closely spaced tandem arrangement of AQP2, AQP5, and AQP6 genes in a 27-kilobase segment at chromosome locus 12q13. Genomics. 1997;43:387–389. 9268644 10.1006/geno.1997.4836

[68] Sawyer SA. A computer package for the statistical detection of gene conversion. Distributed by the author Department of Mathematics, Washington University in St Louis Sawyer website. 1999. Available: http://www.math.wustl.edu/~sawyer.

[69] Yasui M, Kwon TH, Knepper MA, Nielsen S, Agre P. Aquaporin-6: An intracellular vesicle water channel protein in renal epithelia. Proc Natl Acad Sci USA. 1999;96:5808–5813. 10318966 10.1073/pnas.96.10.5808 PMC21942

[70] Beitz E, Liu K, Ikeda M, Guggino WB, Agre P, et al Determinants of AQP6 trafficking to intracellular sites versus the plasma membrane in transfected mammalian cells. Biol Cell. 2006;98:101–109. 15892693 10.1042/BC20050025

[71] Pao GM, Wu LF, Johnson KD, Hofte H, Crispeels MJ, et al Evolution of the mip family of integral membrane-transport proteins. Mol Microbiol. 1991;5:33–37. 2014003 10.1111/j.1365-2958.1991.tb01823.x

[72] Park JH, Saier MH. Phylogenetic characterization of the MIP family of transmembrane channel proteins. J Membr Biol. 1996;153:171–180. 8849412 10.1007/s002329900120

[73] Heymann JB, Engel A. Aquaporins: Phylogeny, structure, and physiology of water channels. News Physiol Sci. 1999;14:187–193. 11390849 10.1152/physiologyonline.1999.14.5.187

[74] Rivera MC, Lake JA. The ring of life provides evidence for a genome fusion origin of eukaryotes. Nature. 2004;431:152–155. 15356622 10.1038/nature02848

[75] Lake JA, Sinsheimer JS. The Deep Roots of the Rings of Life. Genome Biol Evol. 2013;5:2440–2448. 24281049 10.1093/gbe/evt194 PMC3879980

[76] Timmis JN, Ayliffe MA, Huang CY, Martin W. Endosymbiotic gene transfer: Organelle genomes forge eukaryotic chromosomes. Nat Rev Genet. 2004;5:123–U16. 14735123 10.1038/nrg1271

[77] Soto G, Alleva K, Amodeo G, Muschietti J, Ayub ND. New insight into the evolution of aquaporins from flowering plants and vertebrates: Orthologous identification and functional transfer is possible. Gene. 2012;503:165–176. 22561693 10.1016/j.gene.2012.04.021

[78] Perez DG, Juliana, Soto G, Alleva K, Jozefkowicz C, et al Prediction of Aquaporin Function by Integrating Evolutionary and Functional Analyses. J Membr Biol. 2014;247:107–125. 24292667 10.1007/s00232-013-9618-8

[79] Blair JE. Animals (Metazoa). In: Hedges SB, Kumar Seditors. The Timetree of Life. Oxford: Oxford University Press. 2009;pp.220–230.

[80] McLysaght A, Hokamp K, Wolfe KH. Extensive genomic duplication during early chordate evolution. Nature Genet. 2002;31:200–204. 12032567 10.1038/ng884

[81] Dehal P, Boore JL. Two rounds of whole genome duplication in the ancestral vertebrate. PLoS Biol. 2005;3:1700–1708. 10.1371/journal.pbio.0030314 PMC1197285 16128622

[82] Putnam NH, Butts T, Ferrier DEK, Furlong RF, Hellsten U, et al The amphioxus genome and the evolution of the chordate karyotype. Nature. 2008;453:1064–10U3. 18563158 10.1038/nature06967

[83] Amores A, Catchen J, Ferrara A, Fontenot Q, Postlethwait JH. Genome Evolution and Meiotic Maps by Massively Parallel DNA Sequencing: Spotted Gar, an Outgroup for the Teleost Genome Duplication. Genetics. 2011;188:799–U79. 21828280 10.1534/genetics.111.127324 PMC3176089

[84] Drake LL, Boudko DY, Marinotti O, Carpenter VK, Dawe AL, et al**.** The Aquaporin Gene Family of the Yellow Fever Mosquito, *Aedes aegypti*. PLoS One 5.2010. 10.1371/journal.pone.0015578 PMC3014591 21249121

[85] Grohme MA, Mali B, Wełnicz W, Michel S. The aquaporin channel repertoire of the tardigrade *Milnesium tardigradum* . Bioinformatics Biol Insights. 2013;7:153–165. 10.4137/BBI.S11497 PMC3666991 23761966

[86] Berthelot C, Brunet F, Chalopin D, Juanchich A, Bernard M, et al The rainbow trout genome provides novel insights into evolution after whole-genome duplication in vertebrates. Nat Commun. 2014;5:3657. 24755649 10.1038/ncomms4657 PMC4071752

[87] Macqueen DJ, Johnston IA. A well-constrained estimate for the timing of the salmonid whole genome duplication reveals major decoupling from species diversification. Proc Biol Sci. 2014;281:20132881. 24452024 10.1098/rspb.2013.2881 PMC3906940

[88] Kuraku S, Meyer A, Kuratani S. Timing of genome duplications relative to the origin of the vertebrates: Did Cyclostomes diverge before or after? Mol Biol Evol. 2009;26:47–59. 18842688 10.1093/molbev/msn222

[89] Smith JJ, Kuraku S, Holt C, Sauka-Spengler T, Jiang N, et al Sequencing of the sea lamprey (*Petromyzon marinus*) genome provides insights into vertebrate evolution. Nature Genet. 2013;45:415–421. 23435085 10.1038/ng.2568 PMC3709584

[90] Wolf YI, Koonin EV. Genome reduction as the dominant mode of evolution. Bioessays. 2013;35:829–837. 23801028 10.1002/bies.201300037 PMC3840695

[91] Kasahara M, Naruse K, Sasaki S, Nakatani Y, Qu W, et al The medaka draft genome and insights into vertebrate genome evolution. Nature. 2007;447:714–719. 17554307 10.1038/nature05846

[92] Muffato M, Crollius HR. Paleogenomics in vertebrates, or the recovery of lost genomes from the mist of time. Bioessays. 2008;30:122–134. 18200550 10.1002/bies.20707

[93] Nakatani Y, Takeda H, Kohara Y, Morishita S. Reconstruction of the vertebrate ancestral genome reveals dynamic genome reorganization in early vertebrates. Genome Res. 2007;17:1254–1265. 17652425 10.1101/gr.6316407 PMC1950894

[94] Smith JJ, Antonacci F, Eichler EE, Amemiya CT. Programmed loss of millions of base pairs from a vertebrate genome. Proc Natl Acad Sci USA. 2009;106:11212–11217. 19561299 10.1073/pnas.0902358106 PMC2708698

[95] Jones CD, Begun DJ. Parallel evolution of chimeric fusion genes. Proc Natl Acad Sci USA. 2005;102:11373–11378. 16076957 10.1073/pnas.0503528102 PMC1183565

[96] Maher CA, Kumar-Sinha C, Cao X, Kalyana-Sundaram S, Han B, et al Transcriptome sequencing to detect gene fusions in cancer. Nature. 2009;458:97–U9. 19136943 10.1038/nature07638 PMC2725402

[97] Smith JJ, Baker C, Eichler EE, Amemiya CT. Genetic Consequences of Programmed Genome Rearrangement. Curr Biol. 2012;22:1524–1529. 22818913 10.1016/j.cub.2012.06.028 PMC3427415

[98] Gustafsson OSE, Collin SP, Kroeger RHH. Early evolution of multifocal optics for well-focused colour vision in vertebrates. J Exp Biol. 2008;211:1559–1564. 18456882 10.1242/jeb.016048

[99] Mathaes M, Burk O, Qiu H, Nusshag C, Goedtel-Armbrust U, et al Evolutionary History and Functional Characterization of the Amphibian Xenosensor CAR. Mol Endocrinol. 2012;26:14–26. 22074953 10.1210/me.2011-1235 PMC5417167

[100] Hedges SB. Vertebrates (Vertebrata). In: Hedges SB, Kumar Seditors. The Timetree of Life. Oxford: Oxford University Press. 2009;pp.309–314.

[101] Daeschler EB, Shubin NH, Jenkins FA. A Devonian tetrapod-like fish and the evolution of the tetrapod body plan. Nature. 2006;440:757–763. 16598249 10.1038/nature04639

[102] Shubin NH, Daeschler EB, Jenkins FA. The pectoral fin of *Tiktaalik roseae* and the origin of the tetrapod limb. Nature. 2006;440:764–771. 16598250 10.1038/nature04637

[103] Niedzwiedzki G, Szrek P, Narkiewicz K, Narkiewicz M, Ahlberg PE. Tetrapod trackways from the early Middle Devonian period of Poland. Nature. 2010;463:43–48. 20054388 10.1038/nature08623

[104] Amemiya CT, Alfoeldi J, Lee AP, Fan S, Philippe H, et al The African coelacanth genome provides insights into tetrapod evolution. Nature. 2013;496:311–316. 23598338 10.1038/nature12027 PMC3633110

[105] van Balkom BWM, Savelkoul PJM, Markovich D, Hofman E, Nielsen S, et al The role of putative phosphorylation sites in the targeting and shuttling of the aquaporin-2 water channel. J Biol Chem. 2002;277:41473–41479. 12194985 10.1074/jbc.M207525200

[106] Grigg G, Gans C. Morphology & Physiology of the Crocodylia. editors. ‘Fauna of Australia, 2A: Amphibia and Reptilia’. Canberra: Australian Government Publishing Service. 1993;pp. 326–336.

[107] Yasui M, Hazama A, Kwon TH, Nielsen S, Guggino WB, et al Rapid gating and anion permeability of an intracellular aquaporin. Nature. 1999;402:184–187. 10647010 10.1038/46045

[108] Ikeda M, Beitz E, Kozono D, Guggino WB, Agre P, et al Characterization of aquaporin-6 as a nitrate channel in mammalian cells - Requirement of pore-lining residue threonine 63. J Biol Chem. 2002;277:39873–39879. 12177001 10.1074/jbc.M207008200

[109] Cardoso KC, Da Silva MJ, Costa GGL, Torres TT, Del B, et al A transcriptomic analysis of gene expression in the venom gland of the snake *Bothrops alternatus* (urutu). BMC Genomics. 2010;11:605. 20977763 10.1186/1471-2164-11-605 PMC3017861

[110] Taplin LE, Grigg GC. Salt glands in the tongue of the estuarine crocodile *Crocodylus porosus* . Science. 1981;212:1045–1047. 17779977 10.1126/science.212.4498.1045

[111] Rehorek SJ, Smith TD. The primate Harderian gland: Does it really exist? Ann Anat. 2006;188:319–327. 16856596 10.1016/j.aanat.2006.01.018

[112] Delporte C. Aquaporins in salivary glands and pancreas. Biochim Biophys Acta-Gen Subj. 2014;1840:1524–1532. 10.1016/j.bbagen.2013.08.007 23954206

[113] Payne AP. The Harderian gland - A Tercentennial review. J Anat. 1994;185:1–49. 7559104 PMC1166813

[114] Oksche A, Moller A, Dickson J, Rosendahl W, Rascher W, et al Two novel mutations in the aquaporin-2 and the vasopressin V2 receptor genes in patients with congenital nephrogenic diabetes insipidus. Hum Genet. 1996;98:587–589. 8882880 10.1007/s004390050264

[115] VargasPoussou R, Forestier L, Dautzenberg MD, Niaudet P, Dechaux M, et al Mutations in the vasopressin V2 receptor and aquaporin-2 genes in 12 families with congenital nephrogenic diabetes insipidus. J Am Soc Nephrol. 1997;8:1855–1862. 9402087 10.1681/ASN.V8121855

[116] Boone M, Deen PMT. Physiology and pathophysiology of the vasopressin-regulated renal water reabsorption. Pflug Arch Eur J Phy. 2008;456:1005–1024. 10.1007/s00424-008-0498-1 PMC2518081 18431594

[117] Pang PKT, Griffith RW, Atz JW. Osmoregulation in Elasmobranchs. Am Zool. 1977;17:365–377.

[118] Beamish FWH. Osmoregulation in Juvenile and Adult Lampreys. Can J Fish Aquat Sci. 1980;37:1739–1750.

[119] Evans DH. Osmotic and Ionic Regulation. In: Evans DHeditor. The Physiology of Fishes. Boca Raton: CRC Press. 1993;pp.315–341.

[120] Colombo L, Dalla Valle L, Fiore C, Armanini D, Belvedere P. Aldosterone and the conquest of land. J Endocrinol Invest. 2006;29:373–379. 16699307 10.1007/BF03344112

[121] Engelund MB, Chauvigné F, Christensen BM, Finn RN, Cerdà J, et al Differential expression and novel permeability properties of three aquaporin 8 paralogs from seawater-challenged Atlantic salmon smolts. J Exp Biol. 2013;216:3873–3885. 23868847 10.1242/jeb.087890

