# Supplementary figures and images for: The Lineage-Specific Evolution of Aquaporin Gene Clusters Facilitated Tetrapod Terrestrial Adaptation
Source: PLoS One. 2014 Nov 26;9(11):e113686. doi: 10.1371/journal.pone.0113686 (PMC4245216; doi:10.1371/journal.pone.0113686)

Species tree

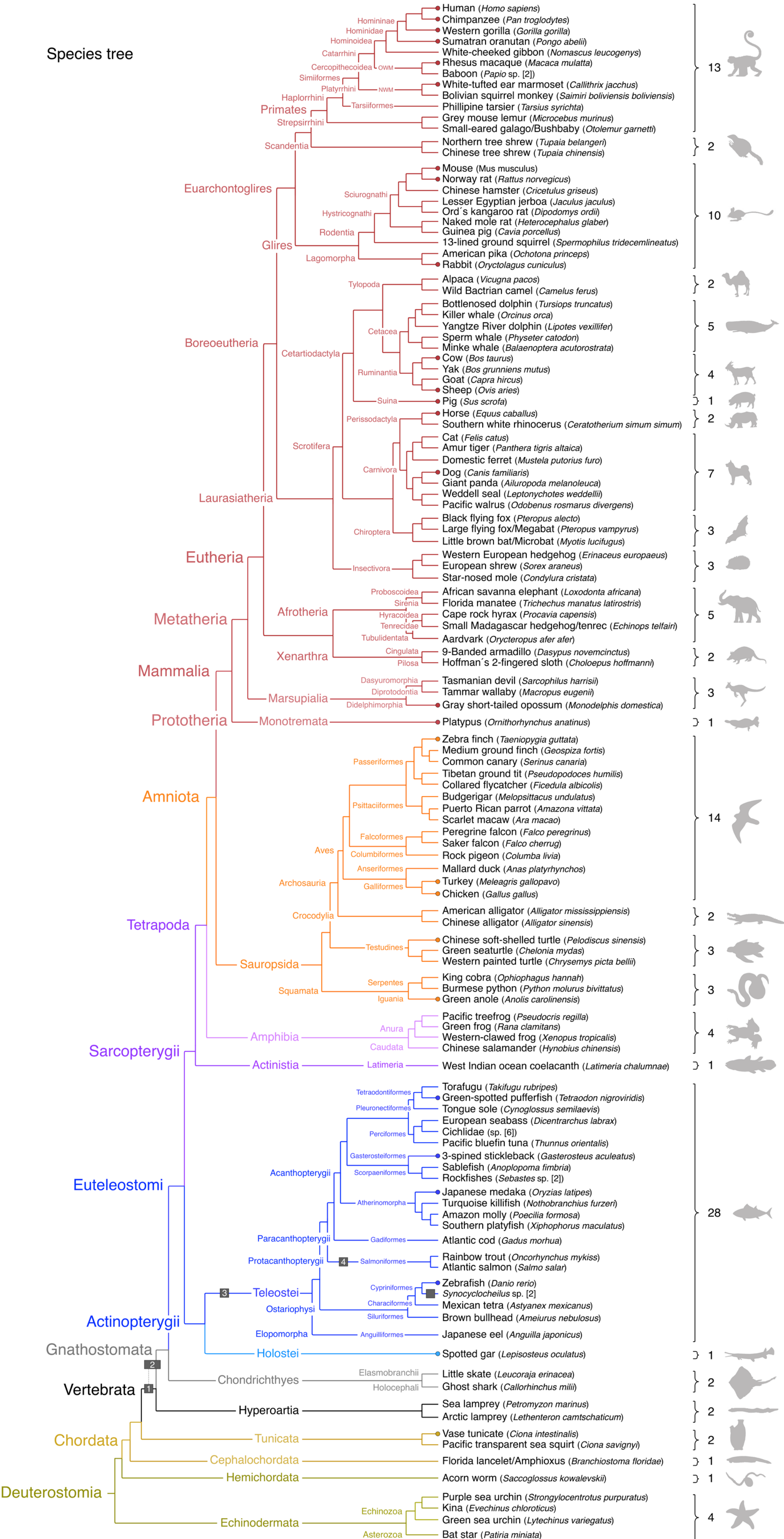

Supplement: Figure S1 — Phylogenetic interrelationships of deuterostome animals for which genomic complements of aquaporins were studied. The number of taxa within each group are shown to the right. Square nodes indicate whole genome duplication. Circular dots on terminal branches indicate an assembled linkage map. (PDF) [file pone.0113686.s001.pdf]

Deuterostomia aquaporins

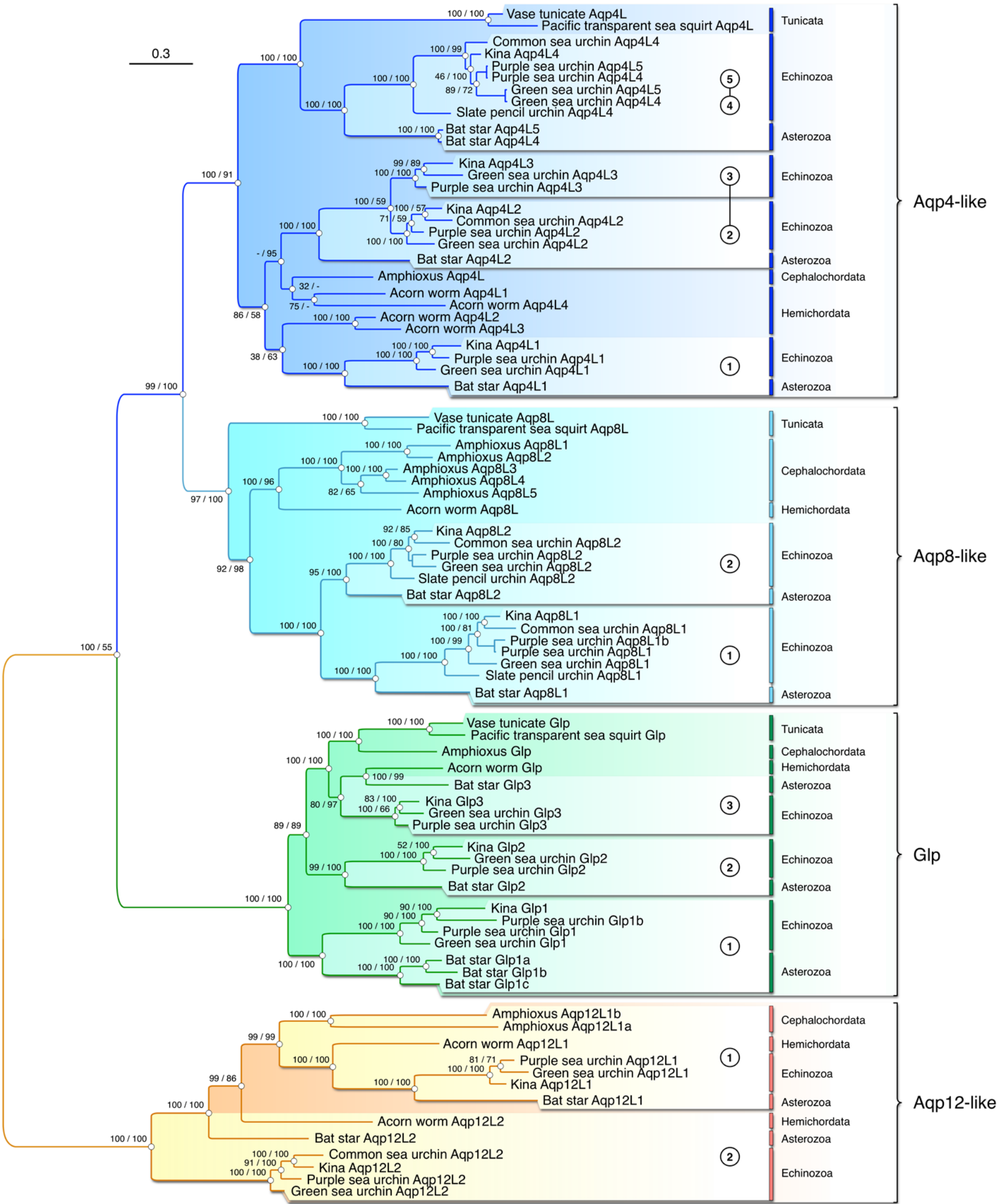

Supplement: Figure S2 — Annotated Bayesian majority rule consensus tree of the basal deuterostome aquaporin superfamily. The tree is mid-point rooted. Posterior probabilities resulting from analyses of the codon/amino acid alignments are shown at each node, with the scale bar indicating the rate of substitutions per site. Four major grades of aquaporin, Aqp4-like, Aqp8-like, Glp and Aqp12-like are respectively shaded in blue, cyan, green and orange with paralogues of a given subfamily highlighted and numerically labeled. (PDF) [file pone.0113686.s002.pdf]

Parazoa-Radiata  
aquaporins

0.5

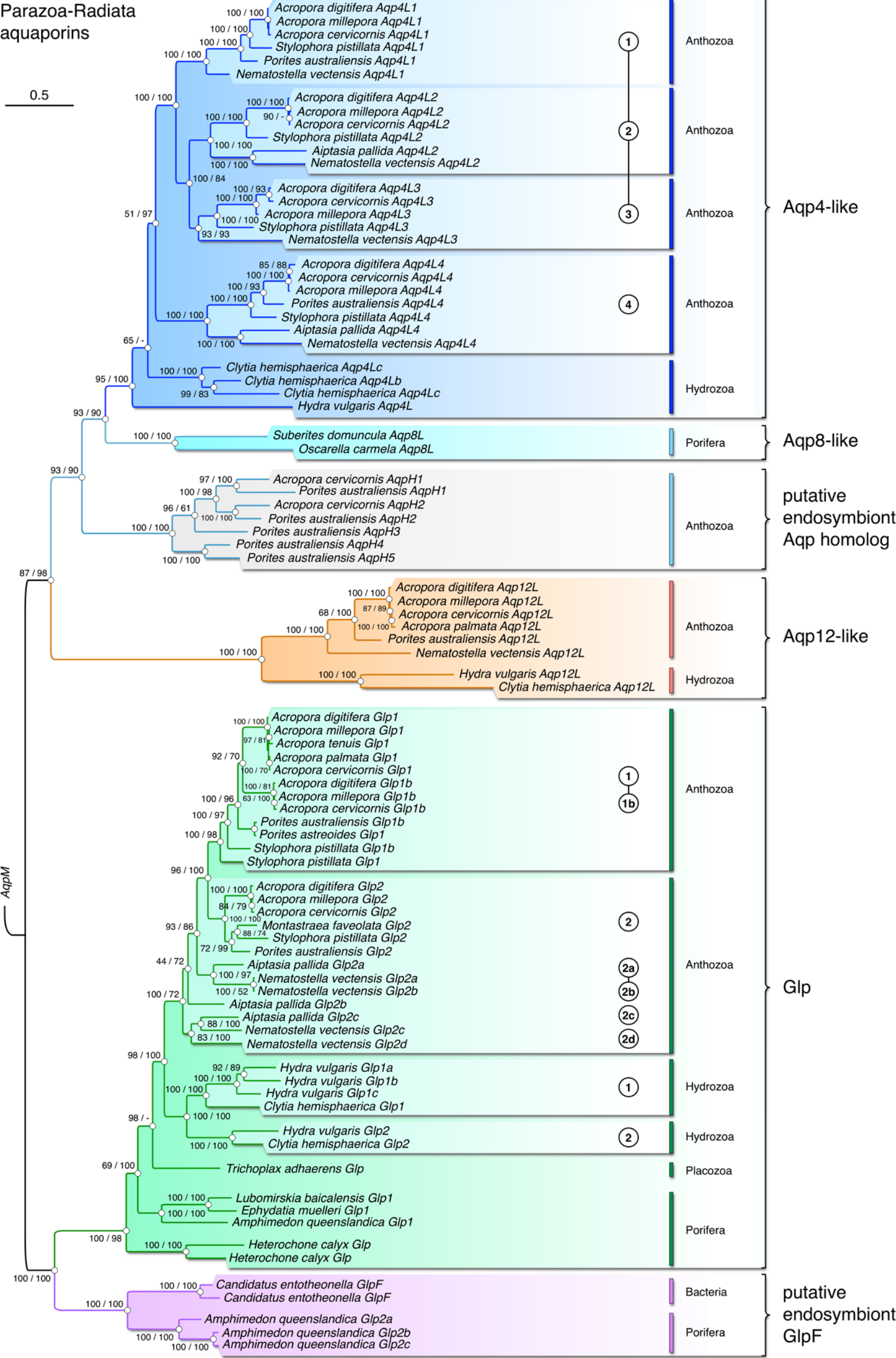

Supplement: Figure S3 — Annotated Bayesian majority rule consensus tree of Parazoa-Radiata aquaporins. The tree is rooted with aqpM. Posterior probabilities resulting from analyses of the codon/amino acid alignments are shown at each node, with the scale bar indicating the rate of substitutions per site. Four major grades of aquaporin Aqp4-like, Aqp8-like, Aqp12-like and Glp are respectively shaded in blue, cyan, orange and green with paralogues of a given subfamily highlighted and numerically labeled. Putative endosymbiont aquaporin and GlpF orthologs are respectively shaded in grey and magenta. Tandemly arranged paralogs are shown as numerically linked circles. (PDF) [file pone.0113686.s003.pdf]

# Parazoa-Radiata-Deuterostomia aquaporins

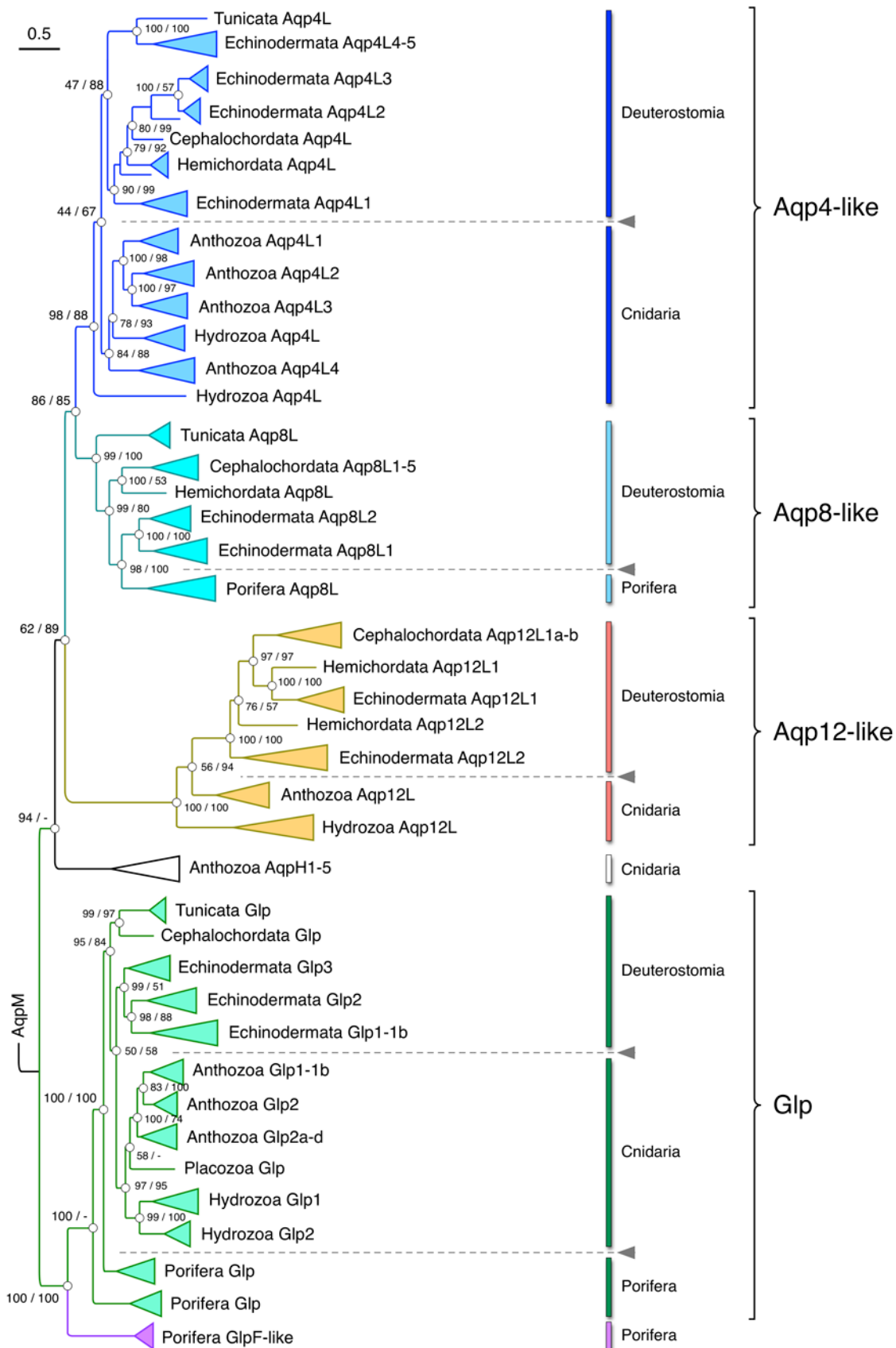

Supplement: Figure S4 — Summarised Bayesian tree of the Parazoa-Cnidaria-Basal deuterostome aquaporins. The tree is rooted with aqpM. Posterior probabilities resulting from analyses of the codon/amino acid alignments are shown at each node, with the scale bar indicating the rate of substitutions per site. (PDF) [file pone.0113686.s004.pdf]

Archaea-Bacteria aquaporins

0.25

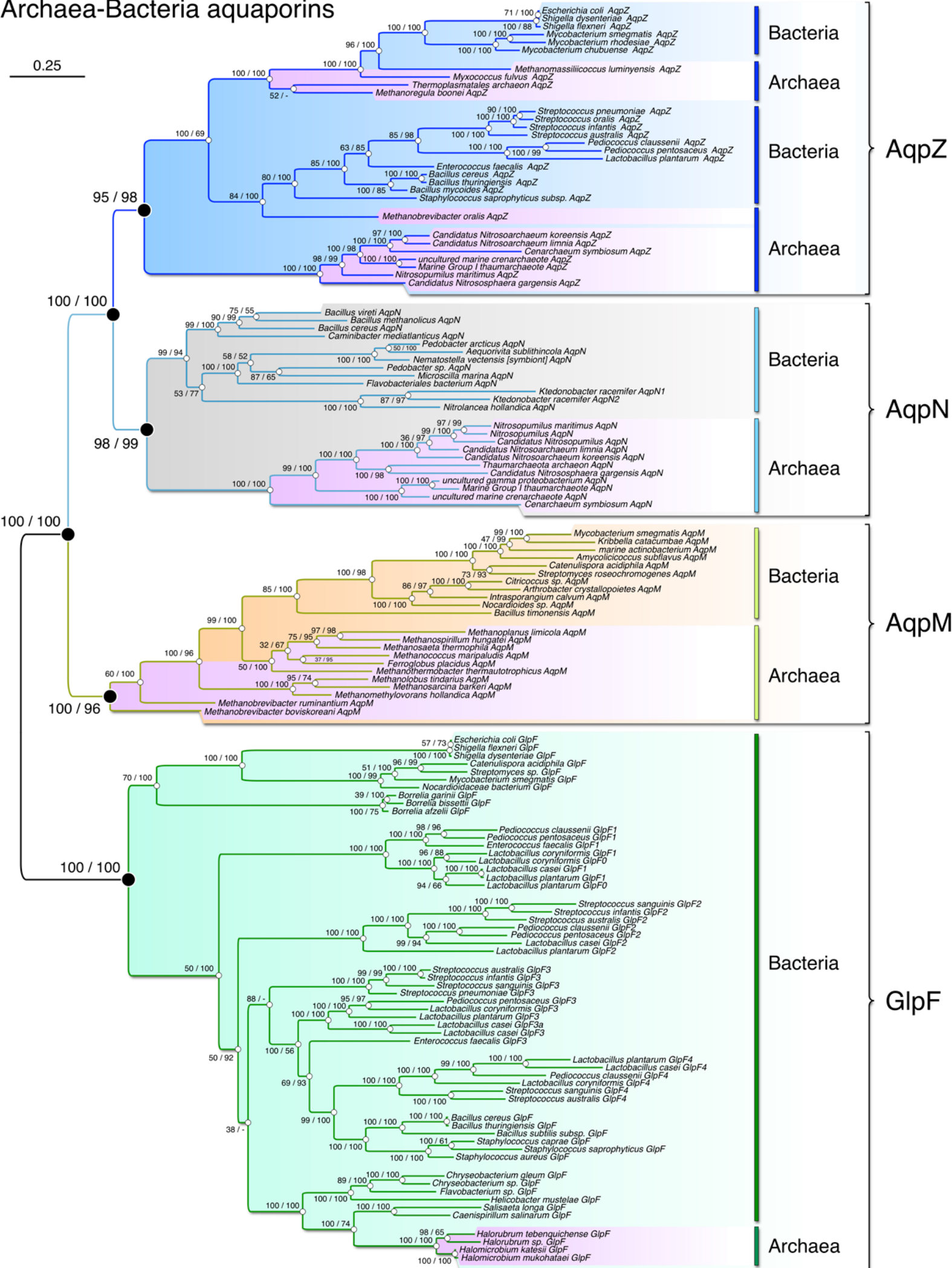

Supplement: Figure S5 — Annotated Bayesian majority rule consensus tree of archaeal and bacterial aquaporins. The tree is mid-point rooted. Posterior probabilities resulting from analyses of the codon/amino acid alignments are shown at each node, with the scale bar indicating the rate of substitutions per site. Four major grades of Bacterial and Archaean aquaporins are shaded in colour, with Archaean orthologs highlighted in pink to show the polyphyletic clustering of Archaean AqpZ orthologs. (PDF) [file pone.0113686.s005.pdf]

Glp

Aquaglyceroporin 3 / 9

Deuterostome  
Aqua-  
glyceroporins

Glp

10

9

7

13

13

Aquaglyceroporin 7 / 10

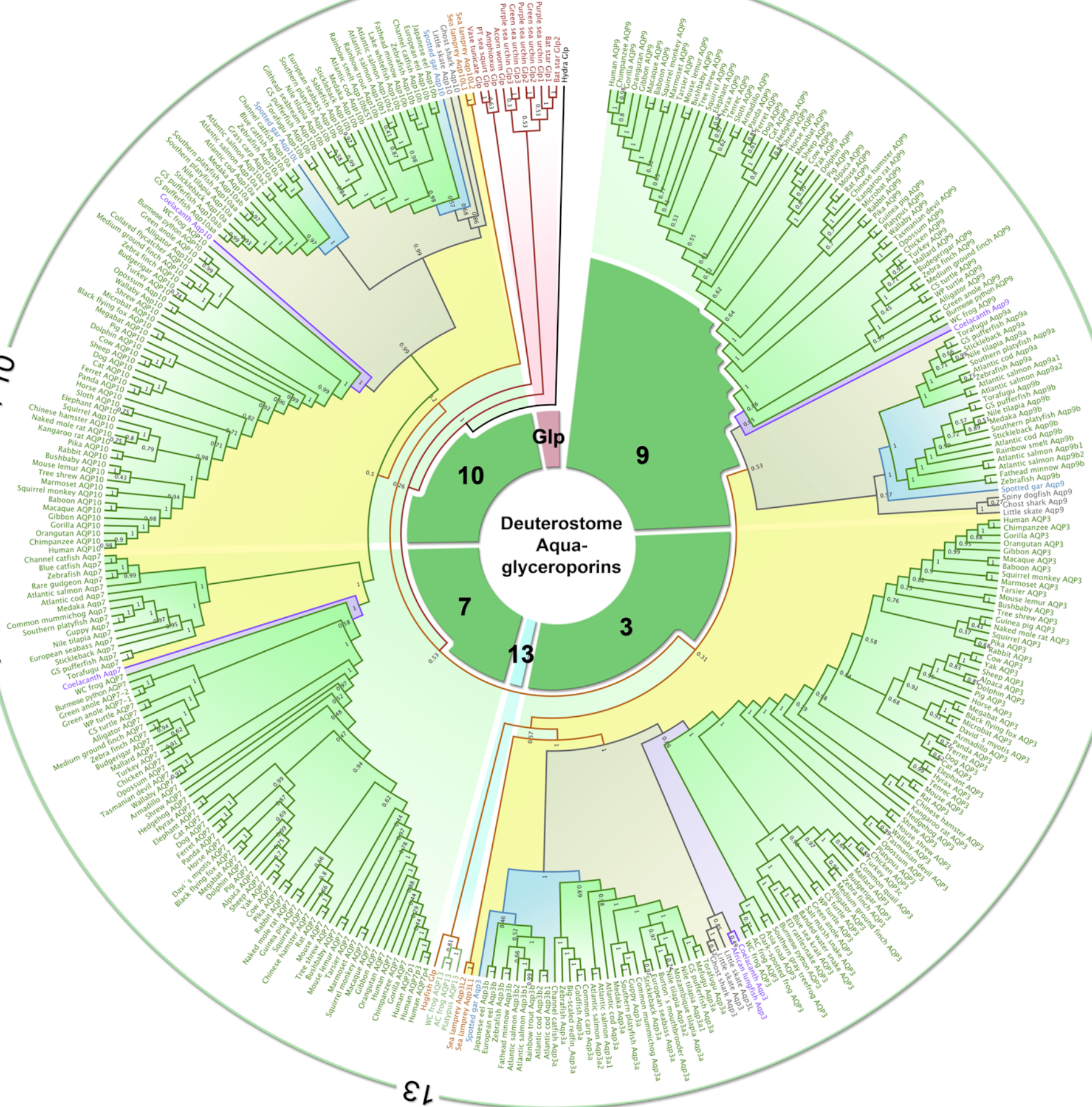

Supplement: Figure S6 — Annotated Bayesian majority rule consensus tree of deuetrostome aquaglyceroporins. The tree is rooted with Hydra vulgaris Glp2. Posterior probabilities resulting from analyses of the codon alignments are shown at each node. Evolutionary older nodes associated with Basal Deuterostomia, Cyclostomata, Chondrichthyes, Holostei and Actinisia are respectively shaded in peach, yellow, grey, cyan and magenta. Teleost and tetrapod subclusters are shaded in green. (PDF) [file pone.0113686.s006.pdf]

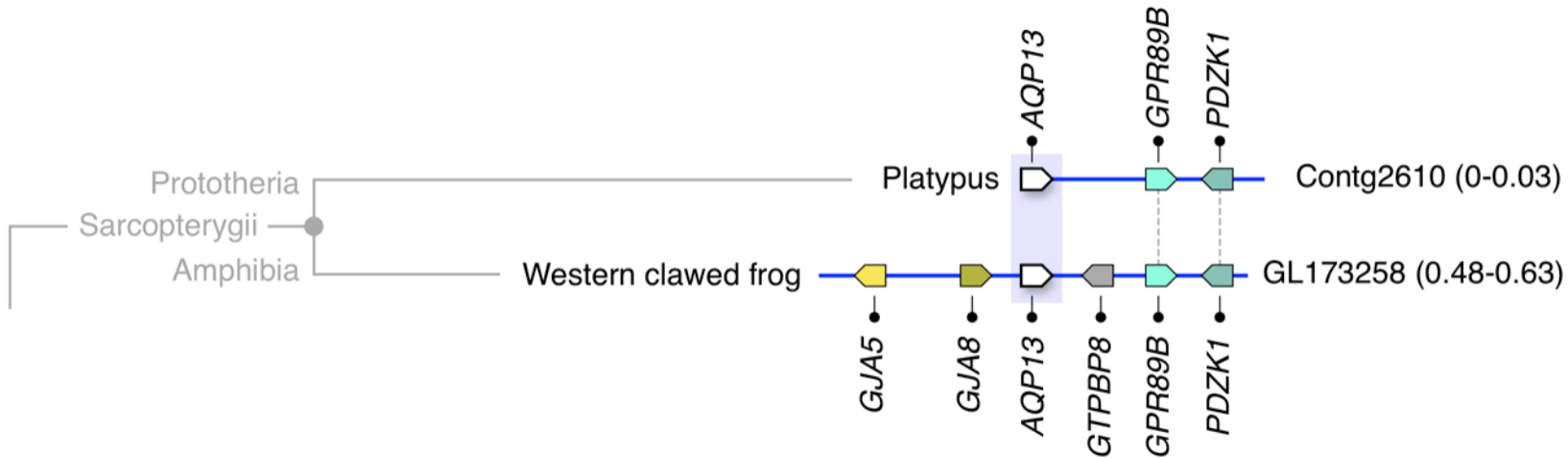

Supplement: Figure S7 — Syntenic alignment of AQP13 . Genomic regions (Mb) are given in parentheses. The coding direction is indicated by the pointed end of the gene symbol. (PDF) [file pone.0113686.s007.pdf]

Unorthodox aquaporins

0.35

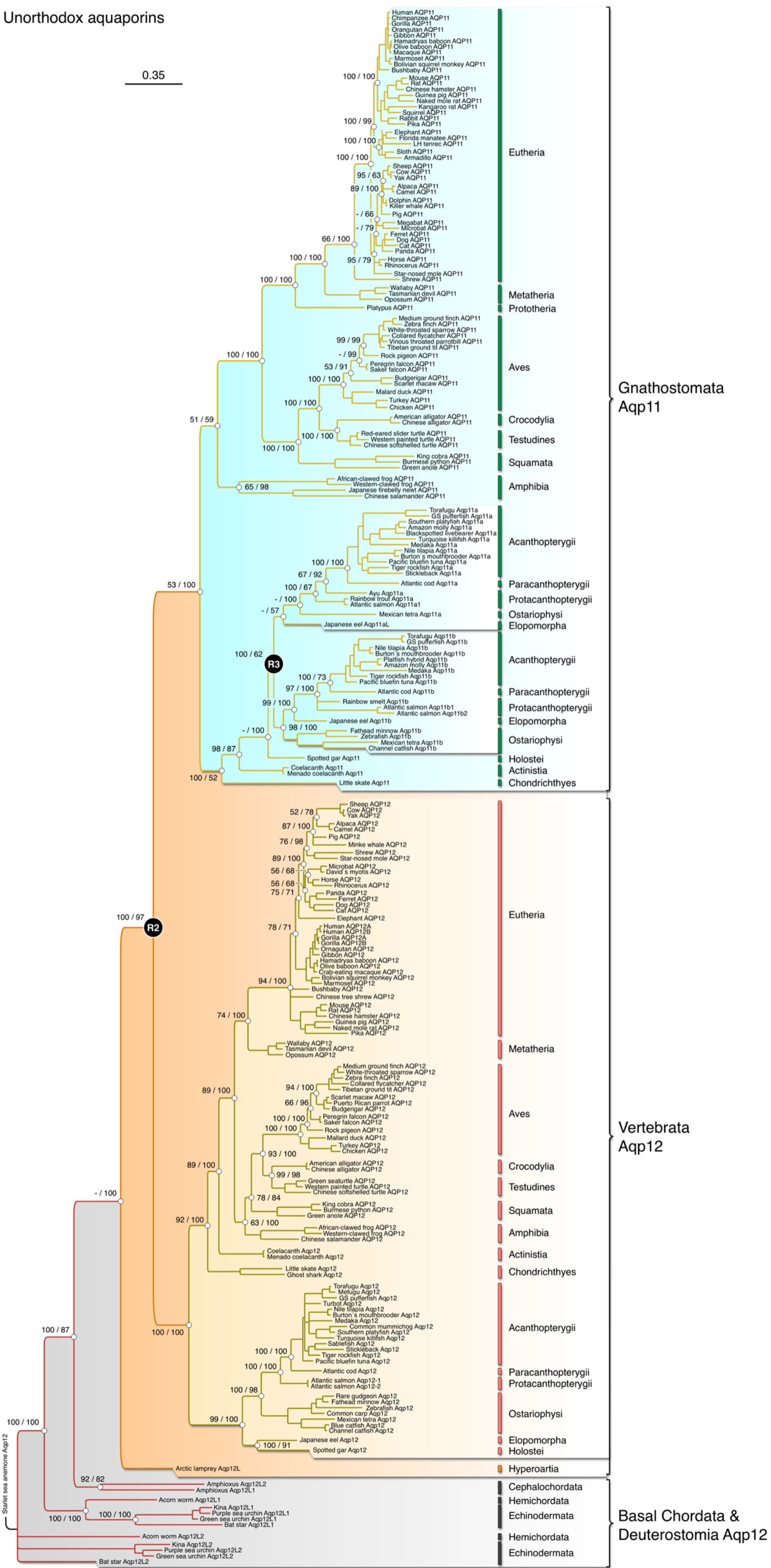

Supplement: Figure S8 — Annotated Bayesian majority rule consensus tree of deuterostome unorthodox aquaporin proteins. The tree is rooted with starlet sea anemone Aqp12. Posterior probabilities resulting from analyses of the codon/amino acid alignments are shown at each node, with the scale bar indicating the rate of substitutions per site. Gnathostome Aqp12 and Aqp11 paralogs are respectively shaded cyan, and light orange. Evolutionary older nodes associated with Cyclostomata and basal Deuterostomia are respectively shaded in orange and grey. Nodes consistent with whole genome duplications (R2, R3) are labelled. (PDF) [file pone.0113686.s008.pdf]

Deuterostome Aqp8 Phylogeny

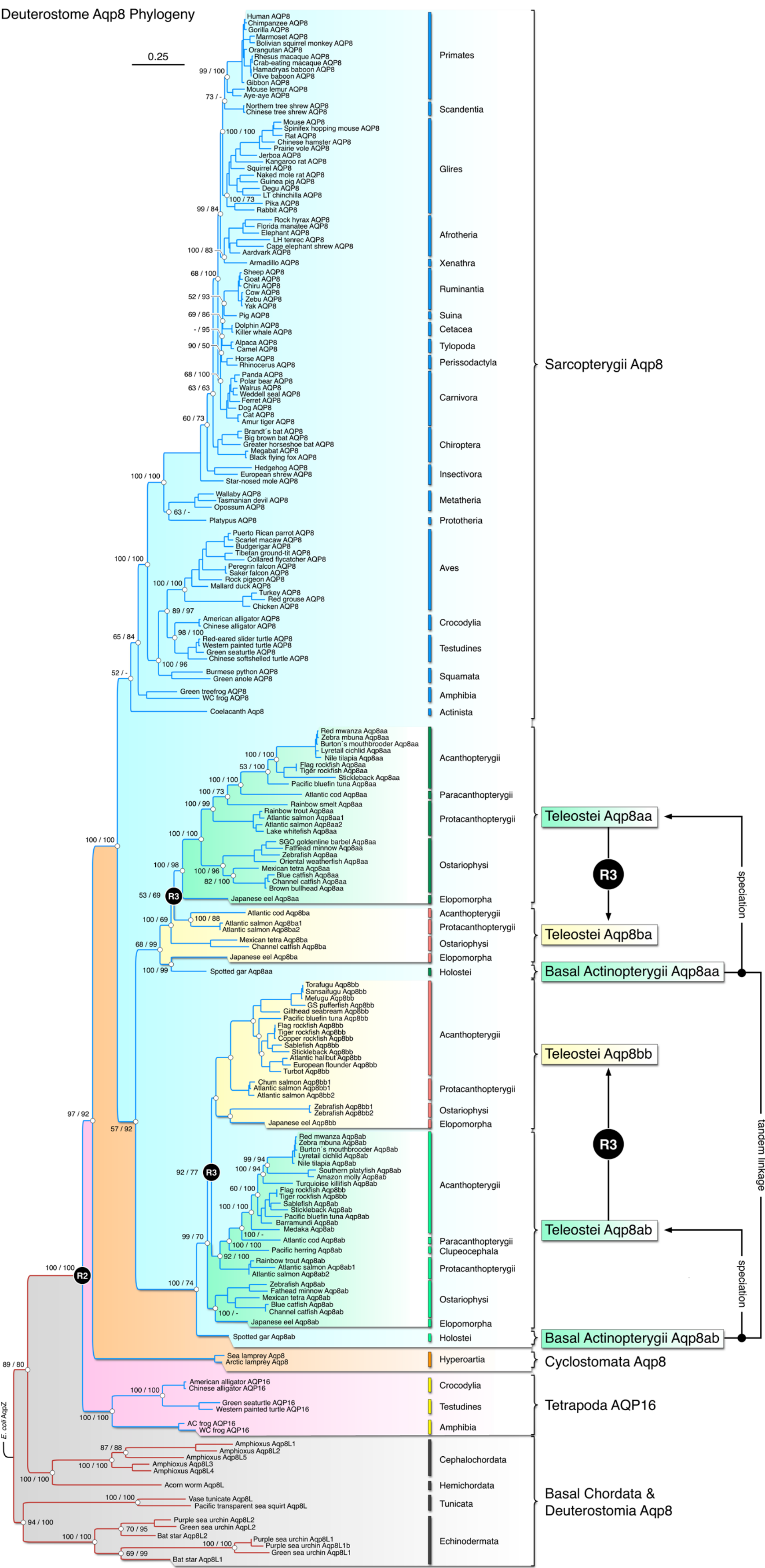

Supplement: Figure S9 — Annotated Bayesian majority rule consensus tree of the deuterostome aquaporin 8 grade. The tree is rooted with bacterial aqpZ. Posterior probabilities resulting from analyses of the codon/amino acid alignments are shown at each node, with the scale bar indicating the rate of substitutions per site. The teleost aqp8aa and -8ab tandem duplicates are shaded in green and the teleost aqp8ba and -8bb genomic duplicates are shaded in yellow. Evolutionary older nodes associated with Cyclostomata and basal Deuterostomia are respectively shaded in orange and grey, while tetrapod AQP16 orthologs are shaded in pink. Nodes consistent with whole genome duplications (R2, R3) are labelled. A schematic of the proposed evolution of actinopterygian aqp8 tandem duplicates and genome duplicates is shown to the right. (PDF) [file pone.0113686.s009.pdf]

(A)

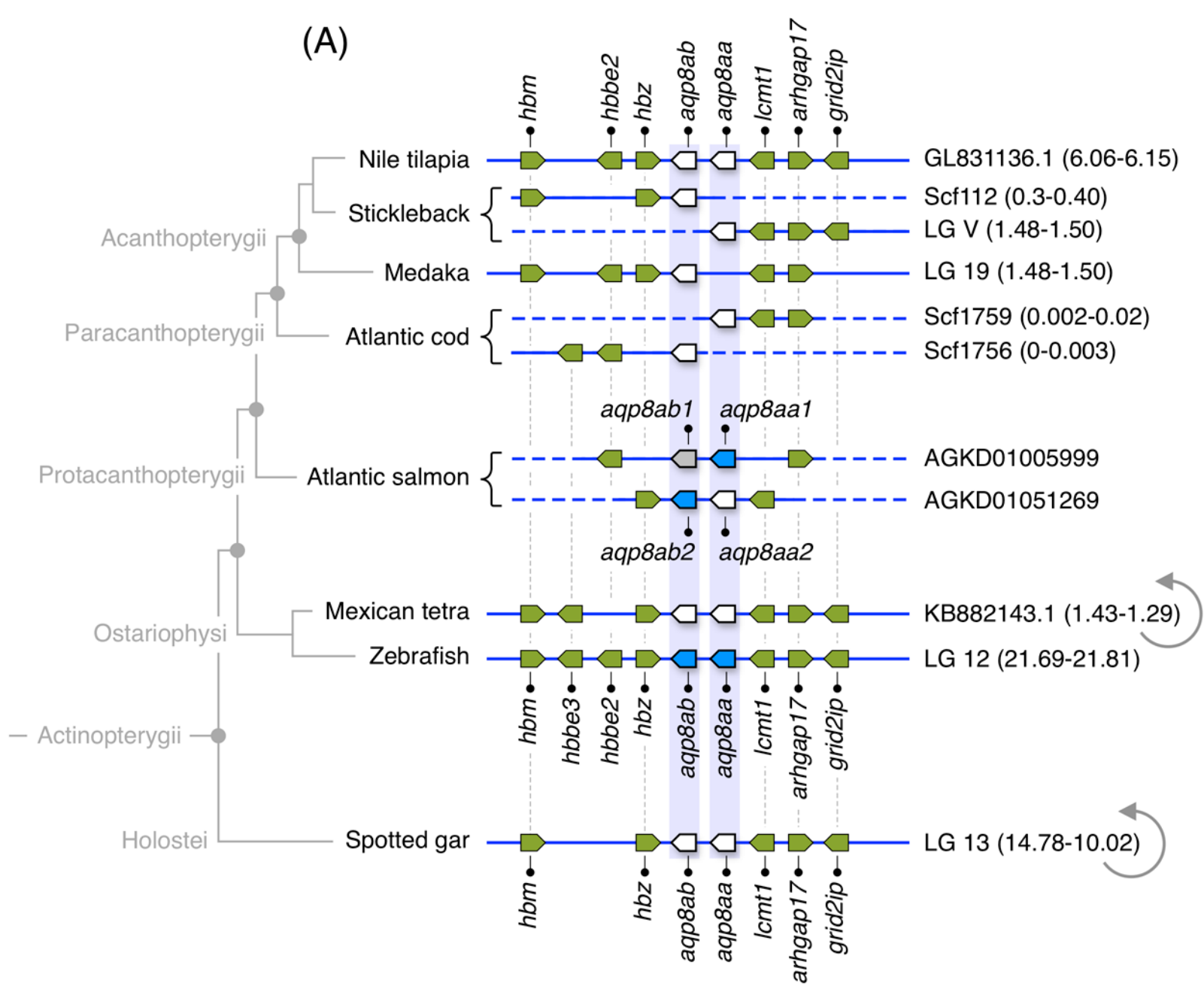

(B)

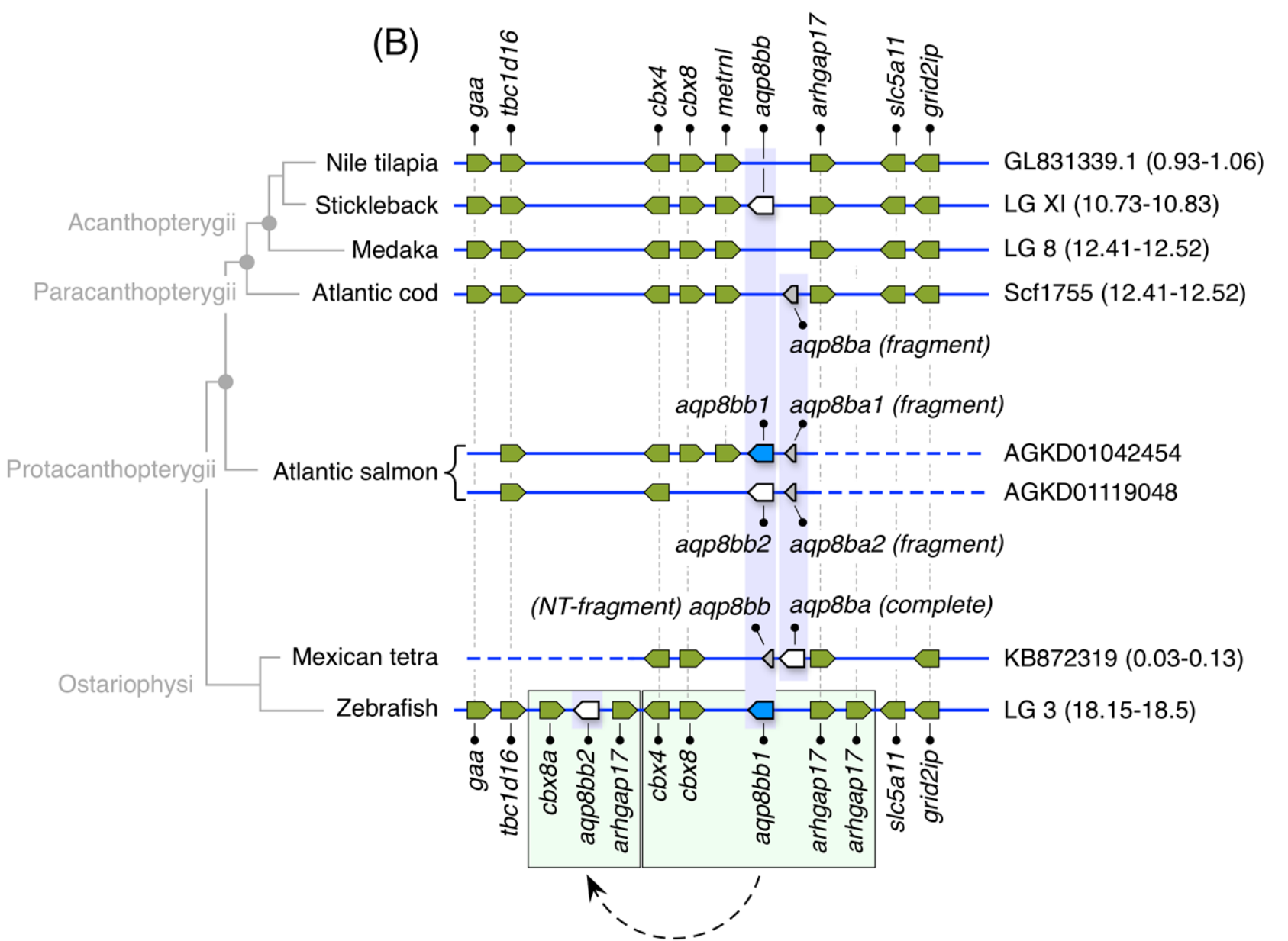

Supplement: Figure S10 — Syntenic alignment of aqp8 genes in Actinopterygii. Genomic regions (Mb) are given in parentheses with circular arrows indicating that the region is flipped in relation to orthologous regions. Coding direction is indicated by the pointed end of the gene symbol. Genes that are previously isolated and cloned [36], [121] are shown as blue symbols (A) Data for actinopterygian aqp8aa and -8ab paralogs. (B) Data for actinopterygian aqp8ba and -8bb paralogs. The zebrafish segmental duplication is boxed in green. (PDF) [file pone.0113686.s010.pdf]

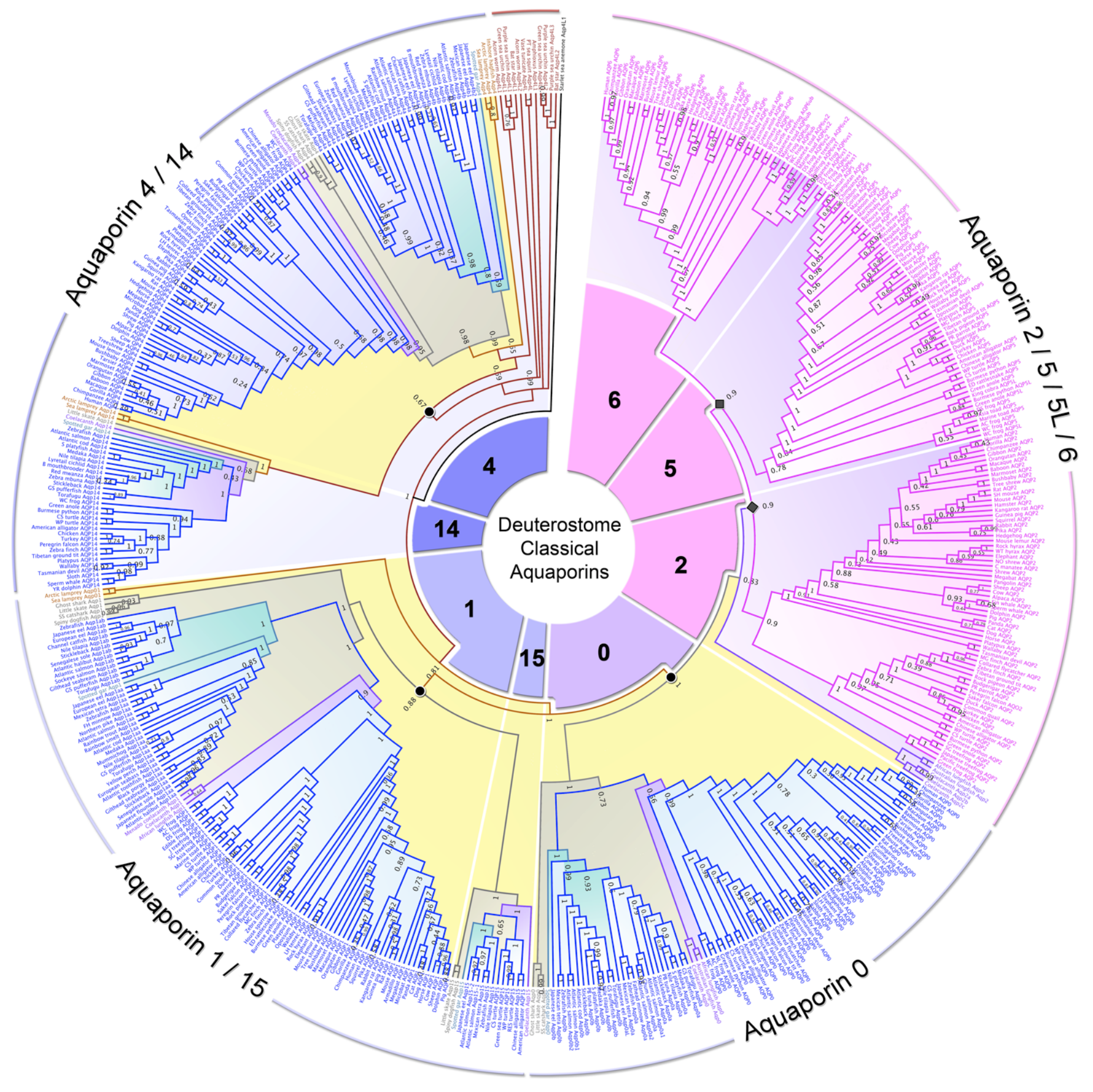

Supplement: Figure S11 — Annotated Bayesian majority rule consensus tree of deuterostome classical aquaporins. The tree is rooted with Nematostella vectensis aqp4L1. Posterior probabilities resulting from analyses of the codon alignments are shown at each node. Nodes consistent with whole genome duplications are labelled with black circles, and nodes consistent with tandem duplications are labeled with black diamonds. Evolutionary older nodes associated with Cyclostomata, Chondrichthyes, Holostei and Actinisia are respectively shaded in yellow, grey, cyan and magenta. Teleost and tetrapod subclusters are shaded in light blue for AQP0, -1, -15, -4 and -14, and pink for AQP2, -5-, 5-like (5L) and -6. (PDF) [file pone.0113686.s011.pdf]

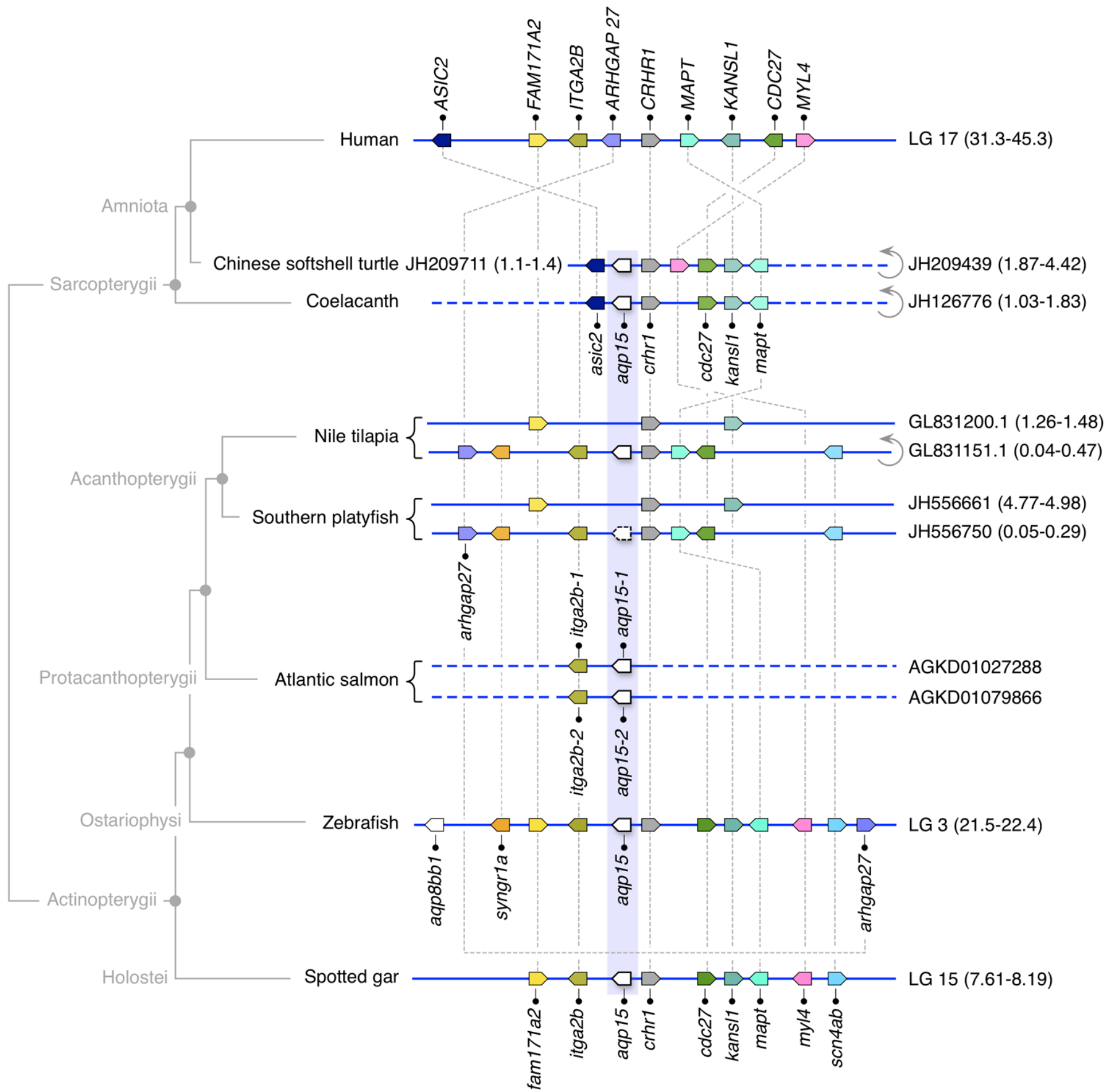

Supplement: Figure S12 — Syntenic alignment of AQP15 . Genomic regions (Mb) are given in parentheses. The coding direction is indicated by the pointed end of the gene symbol. (PDF) [file pone.0113686.s012.pdf]

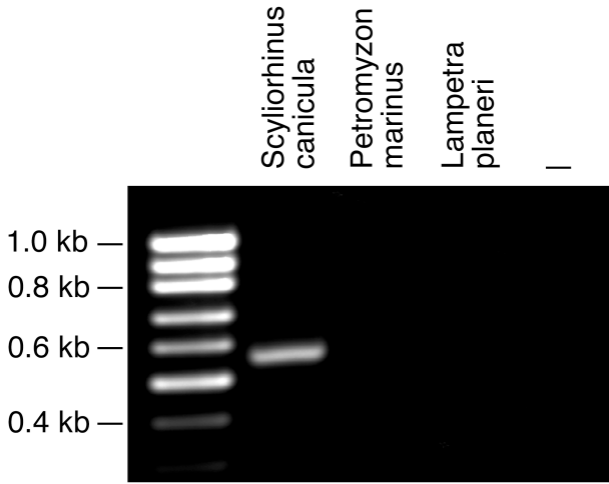

Supplement: Figure S13 — PCR products amplified from Chondrichthyes and Hyperoartia using primers for aqp0 . Representative gel image of RT-PCR analysis using primers flanking the NPA motifs of aqp0 cDNA for the smaller-spotted catshark (Scyliorhinus canicula). Lanes 1-4 are the DNA ladder, and PCR products amplified from the eye mRNA of the smaller-spotted catshark, sea lamprey (Petromyzon marinus) and brook lamprey (Lampetra planeri), respectively. (PDF) [file pone.0113686.s013.pdf]

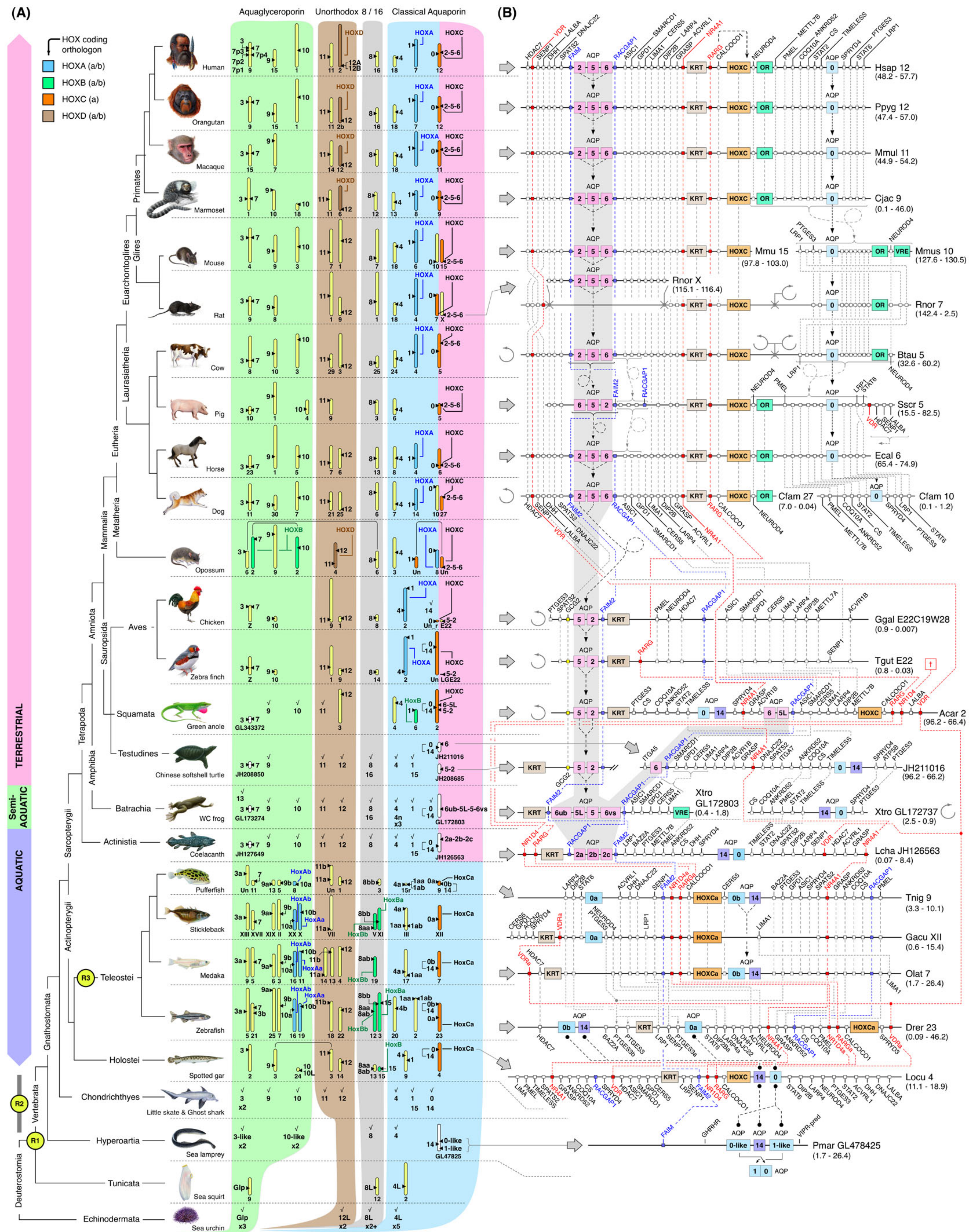

Supplement: Figure S14 — Chromosomal loci and synteny of chordate aquaporins. (A) Linkage groups are drawn to scale within species showing approximate locations of the aquaporin superfamily in each organism. HOX-cluster-bearing chromosomes are coloured according to the key including teleost “a” and “b” duplicated clusters. For organisms without an assembled genome, the presence of a given paralog is annotated with a tick. (B) Syntenic arrangement of vertebrate aqp0, -2, -5, -5L, -6 and -14 genes. Genes are drawn in accordance with their contiguous coding in each genome, with dashed linker lines illustrating rearrangements between the lineages. Nuclear receptors analysed via Bayesian protocols are shown in red, with † indicating extinction of the gene. The AQP2, -5, -5L and -6 gene clusters annotated with pink gene symbols are outlined in light grey to illustrate conservation in Mammalia, but split clusters in Sauropsida. Proximally linked FAIM2 and RACGAP1 are annoted in blue. Circular arrows indicate that the genomic region is flipped. (PDF) [file pone.0113686.s014.pdf]

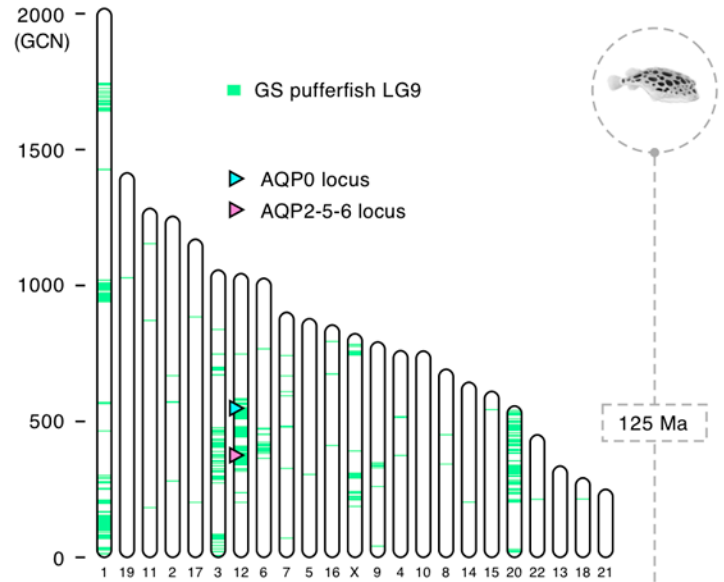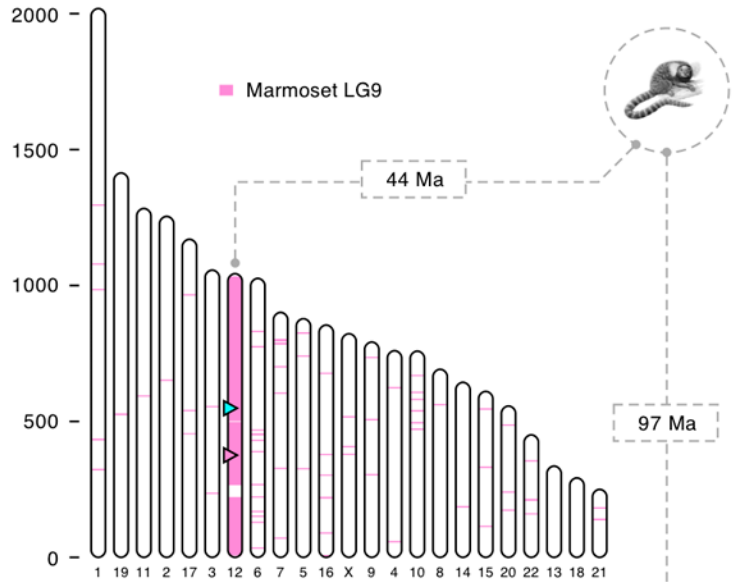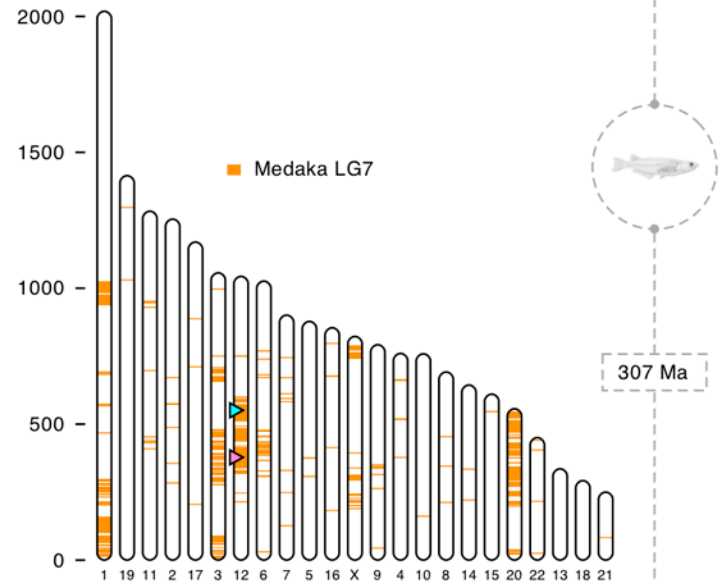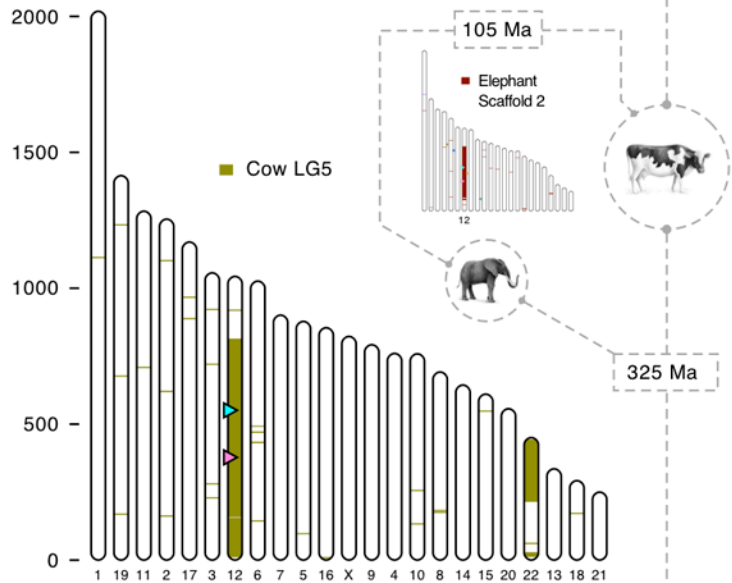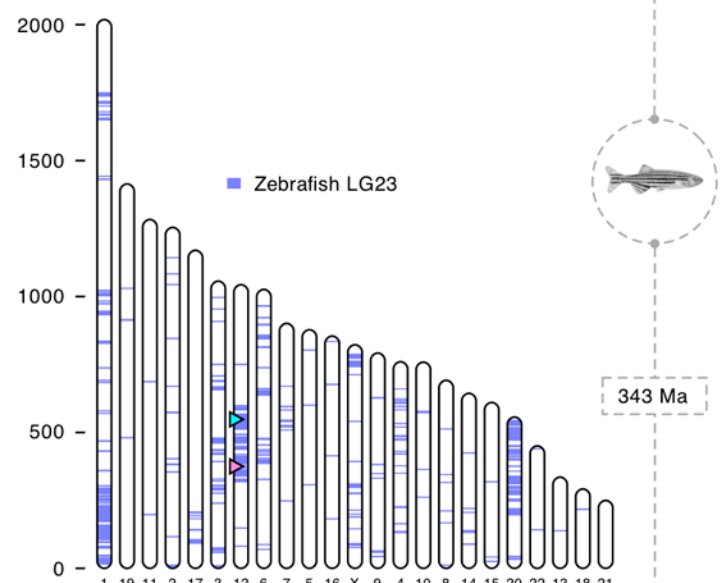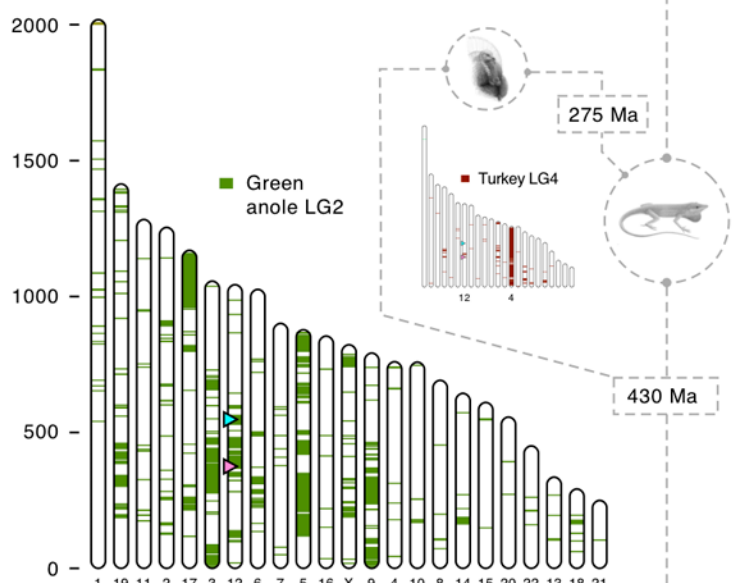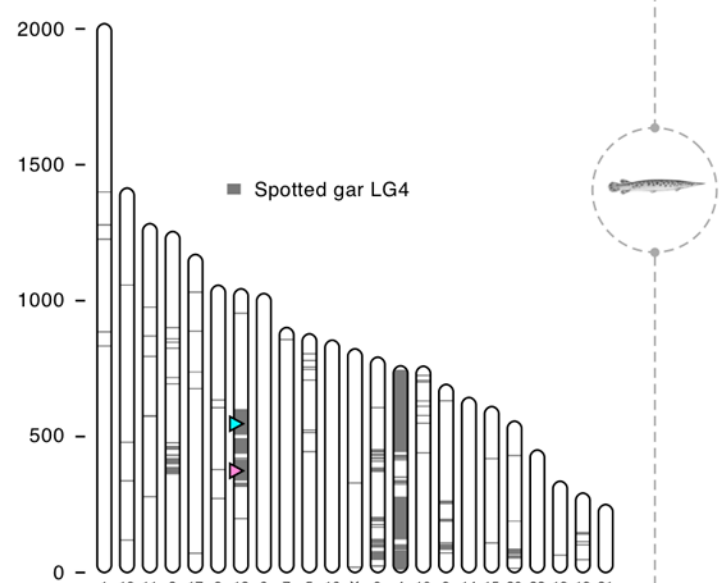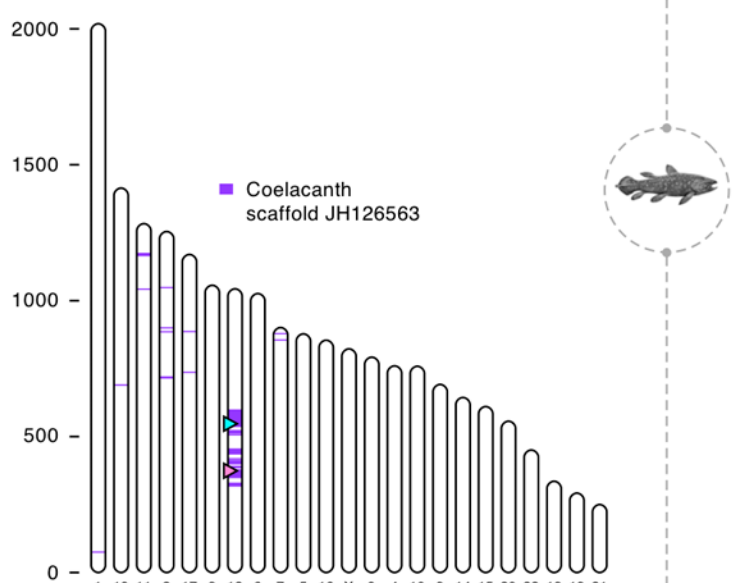

Supplement: Figure S15 — aqp0 linkage group maps to the human karyotype. The linkage group of each organism is mapped using the karyotype view available in Genomicus. The human karyotype is scaled according to the gene copy number (GCN). Boxed numbers represent estimates of the lineage divergence times in millions of years before present (Ma) after Hedges and Kumar [100]. (PDF) [file pone.0113686.s015.pdf]

Nr1d phylogeny

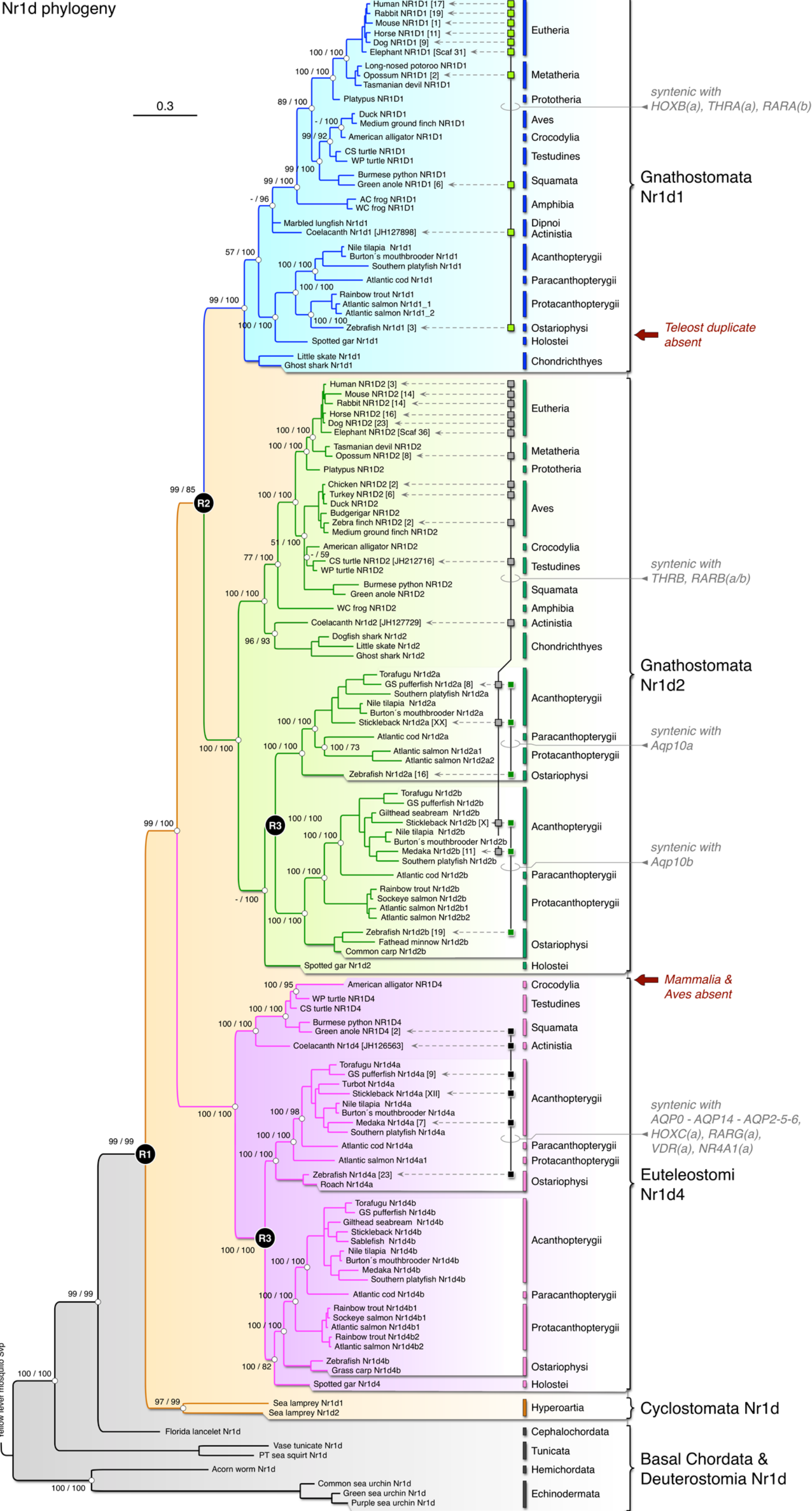

Supplement: Figure S17 — Annotated Bayesian majority rule consensus tree of the deuterostome Nr1d family of nuclear receptors. The tree is rooted with yellow-fever mosquito sevenup (svp). Gnathostome Nr1d1, Nr1d2 and Euteleostomi Nr1d4 paralogs are respectively shaded cyan, green and magenta. Evolutionary older nodes associated with Cyclostomata and basal Deuterostomia are respectively shaded in orange and grey. Labels and annotations are as for Supplementary Figure S16. (PDF) [file pone.0113686.s017.pdf]

## Yellow fever mosquito Svp

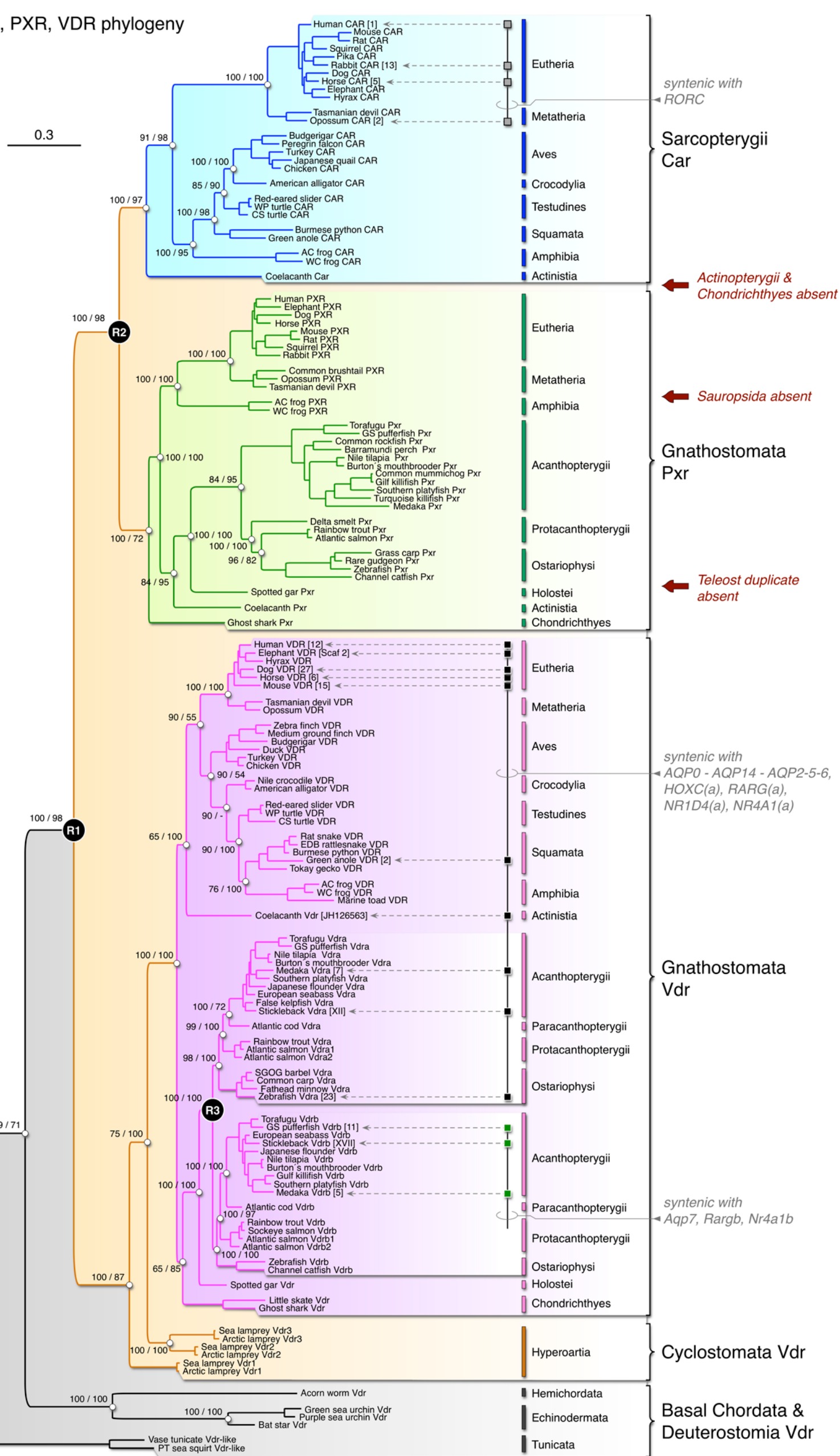

Supplement: Figure S18 — Annotated Bayesian majority rule consensus tree of deuterostome constitutive androgen (CAR), pregnane-X (PXR) and vitamin D (VDR) receptors. The tree is rooted with yellow-fever mosquito svp. Sarcopterygian Car, and gnathostome Pxr and Vdr paralogs are respectively shaded cyan, green and magenta. Evolutionary older nodes associated with Cyclostomata and basal Deuterostomia are respectively shaded in orange and grey. Labels and annotations are as for Supplementary Figure S16. (PDF) [file pone.0113686.s018.pdf]

# Nr4a phylogeny

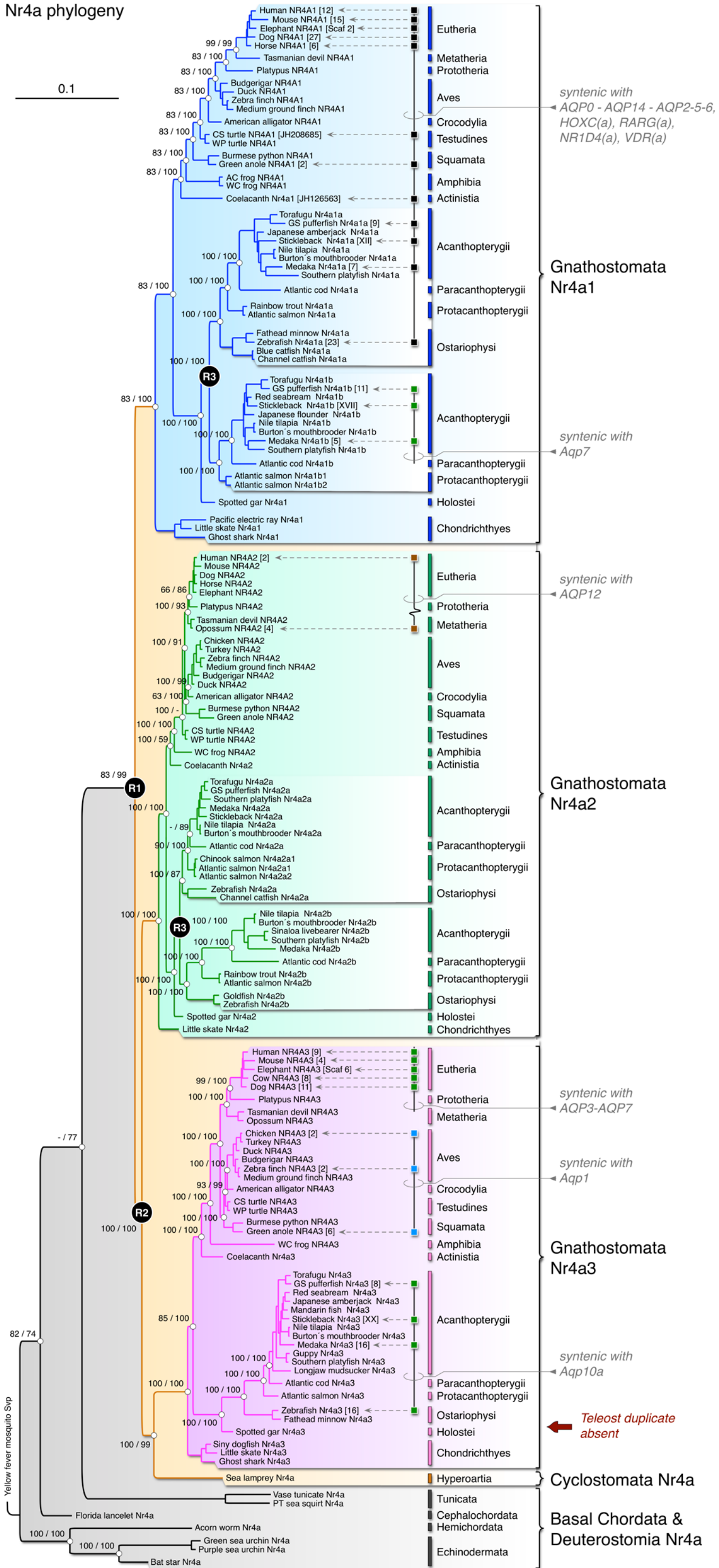

Supplement: Figure S19 — Annotated Bayesian majority rule consensus tree of the deuterostome Nr4A family of nuclear receptors. The tree is rooted with yellow-fever mosquito svp. Gnathostome Nr4a1, Nr4a2 and Nr4a3 paralogs are respectively shaded cyan, green and magenta. Evolutionary older nodes associated with Cyclostomata and basal Deuterostomia are respectively shaded in orange and grey. Labels and annotations are as for Supplementary Figure S16. (PDF) [file pone.0113686.s019.pdf]

— Yellow fever mosquito Svp

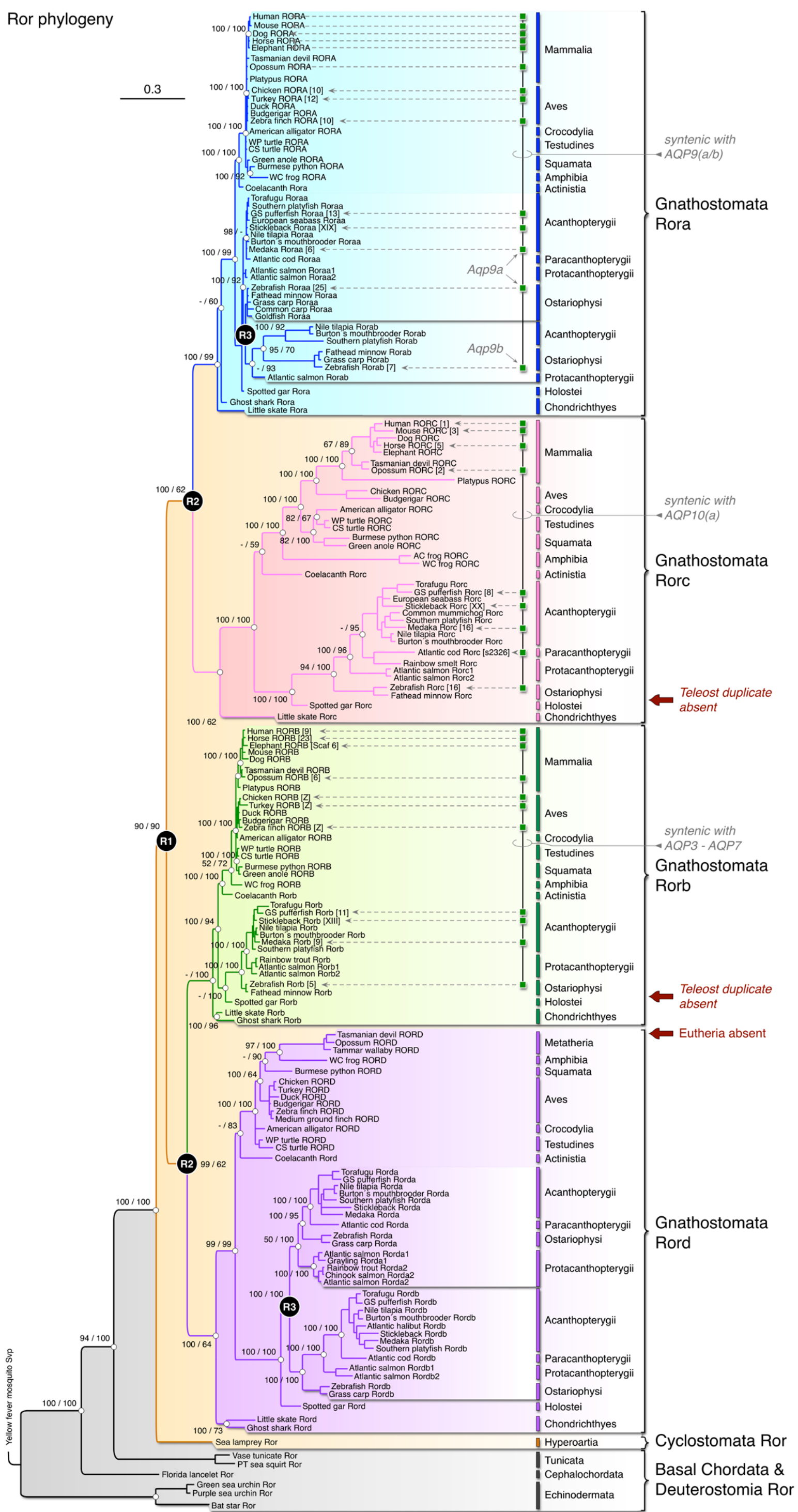

Supplement: Figure S20 — Annotated Bayesian majority rule consensus tree of deuterostome retinoic-related orphan receptors (Ror). The tetraparalogous topology of gnathostome Rors is characterised here for the first time, with Rord identified in Actinopteryii and all extant sarcopterygian lineages except Eutheria. The tree is rooted with yellow-fever mosquito svp. Gnathostome Rora, Rorc, Rorb and Rord paralogs are respectively shaded cyan, red, green and magenta. Evolutionary older nodes associated with Cyclostomata and basal Deuterostomia are respectively shaded in orange and grey. Labels and annotations are as for Supplementary Figure S16. (PDF) [file pone.0113686.s020.pdf]

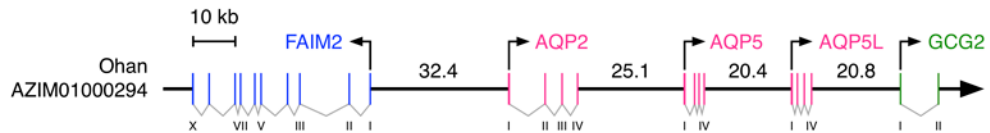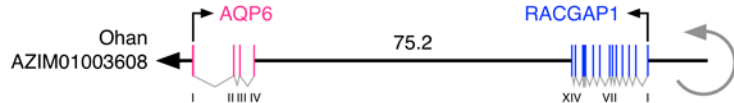

Supplement: Figure S21 — Genomic arrangements of the king cobra AQP2 , -5 , -5L and -6 genes. Exons are labeled in accordance with the coding direction of each gene. Circular arrow indicates that the genomic region is flipped. (PDF) [file pone.0113686.s021.pdf]
