# Supplementary material for: The Lineage-Specific Evolution of Aquaporin Gene Clusters Facilitated Tetrapod Terrestrial Adaptation
Source: PLoS One. 2014 Nov 26;9(11):e113686. doi: 10.1371/journal.pone.0113686 (PMC4245216; doi:10.1371/journal.pone.0113686)
Supplement: Figure S16 — Annotated Bayesian majority rule consensus tree of deuterostome retinoic acid receptors. The tree is mid-point rooted. Posterior probabilities resulting from analyses of the codon/amino acid alignments are shown at each node, with the scale bar indicating the rate of substitutions per site. Gnathostome Rara, Rarb and Rarg paralogs are respectively shaded cyan, green and magenta. Evolutionary older nodes associated with Cyclostomata and basal Deuterostomia are respectively shaded in orange and grey. Nodes consistent with whole genome duplications (R1, R2, R3) are labelled with black circles. Receptors that are syntenic with aquaporins are indicated with coloured squares. (PDF) [file pone.0113686.s016.pdf]

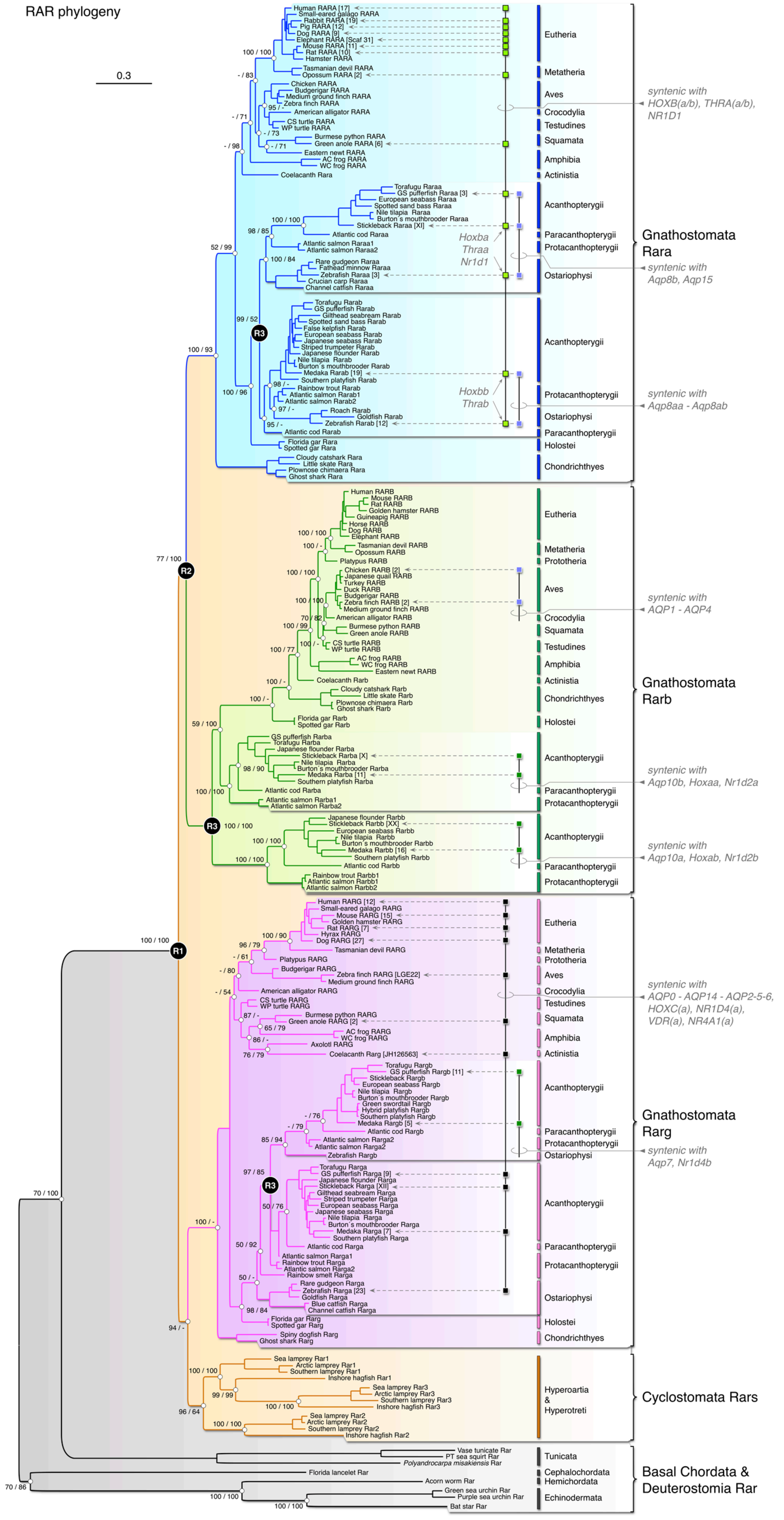

RAR phylogeny
0.3
Human RARA [17]
Small-eared galago RARA
Rabbit RARA [19]
Pig RARA [12]
Dog RARA [9]
Elephant RARA [Scaf 31]
Mouse RARA [11]
Rat RARA [10]
Hamster RARA
Tasmanian devil RARA
Opossum RARA [2]
Chicken RARA
Budgerigar RARA
Medium ground finch RARA
Zebra finch RARA
American alligator RARA
CS turtle RARA
WP turtle RARA
Burmese python RARA
Green anole RARA [6]
Eastern newt RARA
AC frog RARA
WC frog RARA
Coelacanth Rara
Torafugu Raraa
GS pufferfish Raraa [3]
European seabass Raraa
Spotted sand bass Raraa
Nile tilapia Raraa
Burton´s mouthbrooder Raraa
Stickleback Raraa [XI]
Atlantic cod Raraa
Atlantic salmon Raraa1
Atlantic salmon Raraa2
Rare gudgeon Raraa
Fathead minnow Raraa
Zebrafish Raraa [3]
Crucian carp Raraa
Channel catfish Raraa
Hoxba
Thraa
Nr1d1
Torafugu Rarab
GS pufferfish Rarab
Gilthead seabream Rarab
Spotted sand bass Rarab
False kelpfish Rarab
European seabass Rarab
Japanese seabass Rarab
Striped trumpeter Rarab
Japanese flounder Rarab
Nile tilapia Rarab
Burton´s mouthbrooder Rarab
Medaka Rarab [19]
Southern platyfish Rarab
Rainbow trout Rarab
Atlantic salmon Rarab1
Atlantic salmon Rarab2
Roach Rarab
Goldfish Rarab
Zebrafish Rarab [12]
Atlantic cod Rarab
Hoxbb
Thrab
Florida gar Rara
Spotted gar Rara
Cloudy catshark Rara
Little skate Rara
Plownose chimaera Rara
Ghost shark Rara
Eutheria
Metatheria
Aves
Crocodylia
Testudines
Squamata
Amphibia
Actinistia
Acanthopterygii
Paracanthopterygii
Protacanthopterygii
Ostariophysi
Holostei
Chondrichthyes
syntenic with HOXB(a/b), THRA(a/b), NR1D1
Gnathostomata Rara
syntenic with Aqp8b, Aqp15
syntenic with Aqp8aa - Aqp8ab
R3
R2
Human RARB
Mouse RARB
Rat RARB
Golden hamster RARB
Guineapig RARB
Horse RARB
Dog RARB
Elephant RARB
Tasmanian devil RARB
Opossum RARB
Platypus RARB
Chicken RARB [2]
Japanese quail RARB
Turkey RARB
Duck RARB
Budgerigar RARB
Zebra finch RARB [2]
Medium ground finch RARB
American alligator RARB
Burmese python RARB
Green anole RARB
CS turtle RARB
WP turtle RARB
AC frog RARB
WC frog RARB
Eastern newt RARB
Coelacanth Rarb
Cloudy catshark Rarb
Little skate Rarb
Plownose chimaera Rarb
Ghost shark Rarb
Florida gar Rarb
Spotted gar Rarb
GS pufferfish Rarba
Torafugu Rarba
Japanese flounder Rarba
Stickleback Rarba [X]
Nile tilapia Rarba
Burton´s mouthbrooder Rarba
Medaka Rarba [11]
Southern platyfish Rarba
Atlantic cod Rarba
Atlantic salmon Rarba1
Atlantic salmon Rarba2
Japanese flounder Rarbb
Stickleback Rarbb [XX]
European seabass Rarbb
Nile tilapia Rarbb
Burton´s mouthbrooder Rarbb
Medaka Rarbb [16]
Southern platyfish Rarbb
Atlantic cod Rarbb
Rainbow trout Rarbb1
Atlantic salmon Rarbb1
Atlantic salmon Rarbb2
Eutheria
Metatheria
Prototheria
Aves
Crocodylia
Squamata
Testudines
Amphibia
Actinistia
Chondrichthyes
Holostei
Acanthopterygii
Paracanthopterygii
Protacanthopterygii
syntenic with AQP1 - AQP4
Gnathostomata Rarb
syntenic with Aqp10b, Hoxaa, Nr1d2a
syntenic with Aqp10a, Hoxab, Nr1d2b
Human RARG [12]
Small-eared galago RARG
Mouse RARG [15]
Golden hamster RARG
Rat RARG [7]
Hyrax RARG
Dog RARG [27]
Tasmanian devil RARG
Platypus RARG
Budgerigar RARG
Zebra finch RARG [LGE22]
Medium ground finch RARG
American alligator RARG
CS turtle RARG
WP turtle RARG
Burmese python RARG
Green anole RARG [2]
AC frog RARG
WC frog RARG
Axolotl RARG
Coelacanth Rarg [JH126563]
Torafugu Rargb
GS pufferfish Rargb [11]
Stickleback Rargb
European seabass Rargb
Nile tilapia Rargb
Burton´s mouthbrooder Rargb
Green swordtail Rargb
Hybrid platyfish Rargb
Southern platyfish Rargb
Medaka Rargb [5]
Atlantic cod Rargb
Atlantic salmon Rarga2
Atlantic salmon Rarga2
Zebrafish Rargb
Torafugu Rarga
GS pufferfish Rarga [9]
Japanese flounder Rarga
Stickleback Rarga [XII]
Gilthead seabream Rarga
Striped trumpeter Rarga
European seabass Rarga
Japanese seabass Rarga
Nile tilapia Rarga
Burton´s mouthbrooder Rarga
Medaka Rarga [7]
Southern platyfish Rarga
Atlantic cod Rarga
Atlantic salmon Rarga1
Rainbow trout Rarga
Atlantic salmon Rarga2
Rainbow smelt Rarga
Rare gudgeon Rarga
Zebrafish Rarga [23]
Goldfish Rarga
Blue catfish Rarga
Channel catfish Rarga
Florida gar Rarg
Spotted gar Rarg
Spiny dogfish Rarg
Ghost shark Rarg
Eutheria
Metatheria
Prototheria
Aves
Crocodylia
Testudines
Squamata
Amphibia
Actinistia
Acanthopterygii
Paracanthopterygii
Protacanthopterygii
Ostariophysi
Acanthopterygii
Paracanthopterygii
Protacanthopterygii
Ostariophysi
Holostei
Chondrichthyes
syntenic with AQP0 - AQP14 - AQP2-5-6, HOXC(a), NR1D4(a), VDR(a), NR4A1(a)
Gnathostomata Rarg
syntenic with Aqp7, Nr1d4b
R1
Sea lamprey Rar1
Arctic lamprey Rar1
Southern lamprey Rar1
Inshore hagfish Rar1
Sea lamprey Rar3
Arctic lamprey Rar3
Southern lamprey Rar3
Inshore hagfish Rar3
Sea lamprey Rar2
Arctic lamprey Rar2
Southern lamprey Rar2
Inshore hagfish Rar2
Hyperoartia & Hyperotreti
Cyclostomata Rars
Vase tunicate Rar
PT sea squirt Rar
Polyandrocarpa misakiensis Rar
Florida lancelet Rar
Acorn worm Rar
Green sea urchin Rar
Purple sea urchin Rar
Bat star Rar
Tunicata
Cephalochordata
Hemichordata
Echinodermata
Basal Chordata & Deuterostomia Rar
