# Supplementary material for: The Lineage-Specific Evolution of Aquaporin Gene Clusters Facilitated Tetrapod Terrestrial Adaptation
Source: PLoS One. 2014 Nov 26;9(11):e113686. doi: 10.1371/journal.pone.0113686 (PMC4245216; doi:10.1371/journal.pone.0113686)
Supplement: Table S1 — Primer sequences and PCR conditions for cloning of aquaporin cDNAs. (PDF) [file pone.0113686.s022.pdf]

**Table S1:** Primer sequences and PCR conditions for cloning of aquaporin cDNAs

| Species | Gene | Tissue | Primers[1] | PCR conditions | Amplicon (bp) |
|---|---|---|---|---|---|
| *Scyliorhinus canicula* | *aqp0* | eye | Aqp0-d1F=GTGGGAGYTSMRNTCNATGWHBTTYTGG/<br>Aqp0-d1R=GCMGGRGCRAAVGAYCKNGCDGGGTTCA | 94º 1 min, annealing 30 s, 72º 1 min<br>Annealing: 10 cycles at 75º to 55º, change 2º each cycle; 20 cycles at 55º<br>Final extension 72º 7 min | ~580 |
| | *aqp1* | rectal gland | Aqp1-d1F=GAGYKTNGGNCACATMAGYGGNGCHCA/<br>Aqp1-d1R=AGSADRWARTCRTANAYRAKVGCNGC | 94º 1 min, annealing 30 s, 72º 1 min<br>Annealing: 11 cycles at 72º to 50º, change 2º each cycle; 20 cycles at 50º<br>Final extension 72º 7 min | ~501 |
| | *aqp4* | kidney | P1-1F=TAGYGGDKGNCACHTNAAYC/<br>P2-2R=GNCCNACCCARHANACCCART | 94º 1 min, annealing 30 s, 72º 1 min<br>Annealing: 10 cycles at 70º to 50º, change 2º each cycle; 25 cycles at 50º<br>Final extension 72º 7 min | ~440 |
| *Squalus acanthias* | *aqp15* | kidney | Elas1eF= TTYTGGMGIGCIYTIACIGCIGARTTYYT<br>Elas1eR= ARIARIGGICCIACCCARAADATCCA | Cutler et al. (2005)<br>Meischke et al. (2007) | 602 |
| | | | 5'RACE based on PCR product above using:<br>Squal1e-5-R2= GAAGCCCAGAGCCACCTGCAG<br>Squal1e-5-R1= GAGTTGACCACCGCTCACTCG | Following manufacturer`s instructions | ~200 |
| | | | 3'RACE based on PCR product above using:<br>Squal1e-3-F2= GCCCTTGGAGCATCCGTGACC<br>Squal1e-3-F1= GGACATTTGGTGGCAATTGGA | Following manufacturer`s instructions | ~660 |
| *Petromyzon marinus* | *aqp3L1* | eye | P1F=GGNGSNCAYNTNAAYCCNGC/<br>P2R=GGDSCVARNSWBCKNGCNGG | 30 cycles: 94º 1 min, annealing 55º 1 min, 72º 2 min<br>Final extension 72º 7 min | ~350 |
| | *aqp3L2* | eye | P1F=GGNGSNCAYNTNAAYCCNGC/<br>P2R=GGDSCVARNSWBCKNGCNGG | 30 cycles: 94º 1 min, annealing 55º 1 min, 72º 2 min<br>Final extension 72º 7 min | ~350 |
| | *aqp01* | eye | Based on APJL01031882_A_Lamp_Aqp1_DNA genomic sequence (exon 3):<br>LC-01-Ex4F1=CGTGCTAACGAGGAATTTCA/<br>LC-01-Ex5R1=CTTGATGTCGTCTGGGCTCT | 30 cycles: 94º 1 min, annealing 60º 1 min, 72º 2 min | 205 |
| | | | 5'RACE based on PCR product above using:<br>E200-RT=AGCAGGAAGTCGTAGAAG<br>E200-R1=GAACACCCAGTGGTCTTTGAA | Following manufacturer`s instructions | - |
| *Myxine glutinosa* | *glp* | esophagus | Elas9F= TTYGGITGYGGITCIRYIGCICARWC<br>AQP3acR= GGRTTIADIGGRTAICCRCARTTIARICCCAT | Cutler (2006) | 518 |
| | | | 5'RACE based on PCR product above using:<br>Hag9-5-R2= CAGATGAGCTCCAGAGACTCC<br>Hag9-5-R1= GCTGAGCAACGCAGTAGACC | Following manufacturer`s instructions | ~620 |
| | | | 3'RACE based on PCR product above using:<br>Hag9-3-F2= GGTCTACTGCGTTGCTCAGC<br>Hag9-3-F1= GTGCATACTTGCCTTGCTCGAC | Following manufacturer`s instructions | ~380 |

[1]F = forward; R = reverse; RT = reverse primer for cDNA synthesis.

Cutler CP (2006) Cloning of an aquaporin 9 gene orthologue from the hagfish (*Myxine glutinosa*). Bulletin Mount Desert Island Biological Laboratory 45: 42-43.

Cutler CP, Lara Meischke L, Cramb G (2005) Evolutionary and comparative analysis of aquaporin water channel genes in fish. Bulletin Mount Desert Island Biological Laboratory 44: 55

Meischke L, Cramb G, Cutler CP (2007) Cloning and expression of aquaporin water channels in the euryhaline bull shark, *Carcharhinus leucas*. Comparative Biochemistry and Physiology 146: S93
