# Supplementary material for: The Lineage-Specific Evolution of Aquaporin Gene Clusters Facilitated Tetrapod Terrestrial Adaptation
Source: PLoS One. 2014 Nov 26;9(11):e113686. doi: 10.1371/journal.pone.0113686 (PMC4245216; doi:10.1371/journal.pone.0113686)
Supplement: Table S2 — List of aquaporin accession numbers used in the study. (PDF) [file pone.0113686.s023.pdf]

Table S2: List of aquaporin accession numbers used in the study

Accession #	ortholog	Animal	Species	Rank/Grade	Order	Family
AQP0						
ENSP00000257979	AQP0	Human	*Homo sapiens*	Euarchontoglires	Primates	Hominidae
ENSPTRP00000008676	AQP0	Chimpanzee	*Pan troglodytes*	Euarchontoglires	Primates	Hominidae
ENSGGOP00000016164	AQP0	Western lowland gorilla	*Gorilla gorilla gorilla*	Euarchontoglires	Primates	Hominidae
ENSPPYP00000005312	AQP0	Sumatran orangutan	*Pongo abelii*	Euarchontoglires	Primates	Hominidae
ENSNLEP00000021223	AQP0	Northern white-cheeked gibbon	*Nomascus leucogenys*	Euarchontoglires	Primates	Hylobatidae
ENSMMUP00000013540	AQP0	Rhesus macaque	*Macaca mulatta*	Euarchontoglires	Primates	Cercopithecidae
XP_005571292	AQP0	Crab-eating macaque	*Macaca fascicularis*	Euarchontoglires	Primates	Cercopithecidae
ENSP00000257979	AQP0	Hamadryas baboon	*Papio hamadryas*	Euarchontoglires	Primates	Cercopithecidae
XP_003906656	AQP0	Olive baboon	*Papio anubis*	Euarchontoglires	Primates	Cercopithecidae
ENSCJAP00000020259	AQP0	White-tufted-ear marmoset	*Callithrix jacchus*	Euarchontoglires	Primates	Cebidae
XP_003939378	AQP0	Bolivian squirrel monkey	*Saimiri boliviensis boliviensis*	Euarchontoglires	Primates	Cebidae
ENSTSYP00000007346	AQP0	Philippine tarsier	*Tarsius syrichta*	Euarchontoglires	Primates	Tarsiidae
ENSMICP00000009241	AQP0	Gray mouse lemur	*Microcebus murinus*	Euarchontoglires	Primates	Cheirogaleidae
XP_003790578/ENSOGAP00000011482	AQP0	Small-eared galago/Bushbaby	*Otolemur garnettii*	Euarchontoglires	Primates	Galagidae
ENSTBEP00000006948	AQP0	Northern tree shrew	*Tupaia belangeri*	Euarchontoglires	Scandentia	Tupaiidae
ALAR01195931	AQP0	Chinese tree shrew	*Tupaia chinensis*	Euarchontoglires	Scandentia	Tupaiidae
ENSMUSP00000026455	AQP0	Mouse	*Mus musculus*	Euarchontoglires	Rodentia	Muridae
ENSRNOP00000004223	AQP0	Norway rat	*Rattus norvegicus*	Euarchontoglires	Rodentia	Muridae
XP_003506228	AQP0	Chinese hamster	*Cricetulus griseus*	Euarchontoglires	Rodentia	Cricetidae
XP_005371258	AQP0	Prairie vole	*Microtus ochrogaster*	Euarchontoglires	Rodentia	Cricetidae
AKZC01011359/AKZC01011358/AKZC01011357	AQP0	Lesser Egyptian jerboa	*Jaculus jaculus*	Euarchontoglires	Rodentia	Dipodidae
ENSDORP00000005973	AQP0	Ord's kangaroo rat	*Dipodomys ordii*	Euarchontoglires	Rodentia	Heteromyidae
XP_004647118	AQP0	Degu	*Octodon degus*	Euarchontoglires	Rodentia	Octodontidae
ENSCPOP00000002132	AQP0	Domestic guinea pig	*Cavia porcellus*	Euarchontoglires	Rodentia	Caviidae
EHB06533	AQP0	Naked mole-rat	*Heterocephalus glaber*	Euarchontoglires	Rodentia	Bathyergidae
AGTP01085348/ENSSTOP00000002048	AQP0	Thirteen-lined ground squirrel	*Spermophilus tridecemlineatus*	Euarchontoglires	Rodentia	Sciuridae
XP_005397371	AQP0	Long-tailed chinchilla	*Chinchilla lanigera*	Euarchontoglires	Rodentia	Chinchillidae
ALIT01070065/ENSOPRP00000001485	AQP0	American pika	*Ochotona princeps*	Euarchontoglires	Lagomorpha	Ochotonidae
NP_001093431	AQP0	Rabbit	*Oryctolagus cuniculus*	Euarchontoglires	Lagomorpha	Leporidae
AMDU01169065/ENSEEUP00000006846	AQP0	Western European hedgehog	*Erinaceus europaeus*	Laurasiatheria	Insectivora	Erinaceinae
AALT02013458/AALT02013459	AQP0	European shrew	*Sorex araneus*	Laurasiatheria	Insectivora	Soricidae
AJFV01069141	AQP0	Star-nosed mole	*Condylura cristata*	Laurasiatheria	Insectivora	Talpidae
ENSPVAP00000007185/ABRP01010038	AQP0	Large flying fox/Megabat	*Pteropus vampyrus*	Laurasiatheria	Chiroptera	Pteropodidae
ELK15285	AQP0	Black flying fox	*Pteropus alecto*	Laurasiatheria	Chiroptera	Pteropodidae
AWHC01164199	AQP0	Straw-colored fruit bat	*Eidolon helvum*	Laurasiatheria	Chiroptera	Pteropodidae
ENSMLUP00000006243	AQP0	Little brown bat/Microbat	*Myotis lucifugus*	Laurasiatheria	Chiroptera	Vespertilionidae
ALWT01314484	AQP0	David´s myotis	*Myotis davidii*	Laurasiatheria	Chiroptera	Vespertilionidae
XP_005876816	AQP0	Brandt's bat	*Myotis brandtii*	Laurasiatheria	Chiroptera	Vespertilionidae
AWHB01448805/AWHB01346598	AQP0	Indian false vampire	*Megaderma lyra*	Laurasiatheria	Chiroptera	Megadermatidae
ENSP00000257979	AQP0	Sheep	*Ovis aries*	Laurasiatheria	Ruminantia	Bovidae
AJPT01056746 /XP_005680399	AQP0	Goat	*Capra hircus*	Laurasiatheria	Ruminantia	Bovidae
XP_005960818/ AGTT01249570	AQP0	Chiru	*Pantholops hodgsonii*	Laurasiatheria	Ruminantia	Bovidae
ENSBTAP00000013360/ AAFC03087741	AQP0	Cow	*Bos taurus*	Laurasiatheria	Ruminantia	Bovidae
ELR50774/ AGSK01134320	AQP0	Yak	*Bos mutus*	Laurasiatheria	Ruminantia	Bovidae
AGFL01047914	AQP0	Zebu	*Bos indicus*	Laurasiatheria	Ruminantia	Bovidae
ENSTTRP00000006787	AQP0	Bottlenosed dolphin	*Tursiops truncatus*	Laurasiatheria	Cetacea	Delphinidae
ANOL02036785	AQP0	Killer whale	*Orcinus orca*	Laurasiatheria	Cetacea	Delphinidae
AUPI01131496	AQP0	Yangtze River dolphin	*Lipotes vexillifer*	Laurasiatheria	Cetacea	Lipotidae
AWZP01107178	AQP0	Sperm whale	*Physeter catodon*	Laurasiatheria	Cetacea	Physeteridae
ATDI01166774	AQP0	Minke whale	*Balaenoptera acutorostrata*	Laurasiatheria	Cetacea	Balaenopteridae
ENSSSCP00000000426	AQP0	Pig	*Sus scrofa*	Laurasiatheria	Suina	Suidae
ABRR02151252	AQP0	Alpaca	*Vicugna pacos*	Laurasiatheria	Tylopoda	Camelidae
AGVR01019229	AQP0	Wild Bactrian camel	*Camelus ferus*	Laurasiatheria	Tylopoda	Camelidae
ENSECAP00000006778	AQP0	Horse	*Equus caballus*	Laurasiatheria	Perissodactyla	Equidae
AKZM01023099	AQP0	Southern white rhinocerus	*Ceratotherium simum simum*	Laurasiatheria	Perissodactyla	Rhinocerotidae
ENSAMEP00000004222	AQP0	Giant panda	*Ailuropoda melanoleuca*	Laurasiatheria	Carnivora	Ursidae
GAJD01020567	AQP0	Polar bear	*Ursus maritimus*	Laurasiatheria	Carnivora	Ursidae
ENSCAFP00000000190	AQP0	Dog	*Canis lupus familiaris*	Laurasiatheria	Carnivora	Canidae
ENSMPUP00000001444/AGTQ0102688	AQP0	Domestic ferret	*Mustela putorius furo*	Laurasiatheria	Carnivora	Mustelidae
ENSFCAP00000011157	AQP0	Domestic cat	*Felis catus*	Laurasiatheria	Carnivora	Felidae
ATCQ01051901	AQP0	Amur tiger	*Panthera tigris altaica*	Laurasiatheria	Carnivora	Felidae
APMU01097947	AQP0	Weddell seal	*Leptonychotes weddellii*	Laurasiatheria	Carnivora	Phocidae
ANOP01018828	AQP0	Pacific walrus	*Odobenus rosmarus divergens*	Laurasiatheria	Carnivora	Odobenidae
ENSLAFP00000019404	AQP0	African savanna elephant	*Loxodonta africana*	Afrotheria	Proboscidea	Elephantidae
AHIN01072581	AQP0	Florida manatee	*Trichechus manatus latirostris*	Afrotheria	Sirenia	Trichechidae
ABRQ01696750	AQP0	Cape rock hyrax	*Procavia capensis*	Afrotheria	Hyracoidea	Procaviidae
ENSETEP00000009755	AQP0	Lesser hedgehog tenrec	*Echinops telfairi*	Afrotheria	Afrosoricida	Tenrecidae
AMDV01086667	AQP0	Cape golden mole	*Chrysochloris asiatica*	Afrotheria	Afrosoricida	Chrysochloridae

AMGZ01214343	AQP0	Cape elephant shrew	*Elephantulus edwardii*	Afrotheria	Macroscelidea	Macroscelididae
ALYB01301190	AQP0	Ardvaark	*Orycteropus afer afer*	Afrotheria	Tubulidentata	Orycteropodidae
AAGV03143792	AQP0	Nine-banded armadillo	*Dasypus novemcinctus*	Xenarthra	Cingulata	Dasypodidae
ENSCHOP00000011237	AQP0	Hoffmann's two-fingered sloth	*Choloepus hoffmanni*	Xenarthra	Pilosa	Megalonychidae
ENSMEUP00000011464	AQP0	Tammar wallaby	*Macropus eugenii*	Metatheria	Diprotodontia	Macropodidae
ENSSHAP00000002808	AQP0	Tasmanian devil	*Sarcophilus harrisii*	Metatheria	Dasyuromorphia	Dasyuridae
ENSMODP00000026443	AQP0	Gray short-tailed opossum	*Monodelphis domestica*	Metatheria	Didelphimorphia	Didelphidae
ENSOANP00000029592/ENSOANP00000019822	AQP0	Platypus	*Ornithorhynchus anatinus*	Prototheria	Monotremata	Ornithorhynchidae
AOCU01154550/AOCU01203708	AQP0	Puerto Rican parrot	*Amazona vittata*	Aves	Psittaciformes	Psittacidae
CAVT010223535/CAVT010189548	AQP0	Common canary	*Serinus canaria*	Aves	Passeriformes	Fringillidae
AKZB01048032/AKZB01002023	AQP0	Medium ground finch	*Geospiza fortis*	Aves	Passeriformes	Fringillidae
ENSTGUP00000014885/XP_002200311	AQP0	Zebra finch	*Taeniopygia guttata*	Aves	Passeriformes	Estrildidae
ANZD01003873/XP_005532018	AQP0	Tibetan ground-tit	*Pseudopodoces humilis*	Aves	Passeriformes	Paridae
AGTO01028949/AGTO01011829/ ENSFALP00000015205	AQP0	Collared flycatcher	*Ficedula albicollis*	Aves	Passeriformes	Muscicapidae
AKMT01045875	AQP0	Peregrin falcon	*Falco peregrinus*	Aves	Falconiformes	Falconidae
AKMU01048961	AQP0	Saker falcon	*Falco cherrug*	Aves	Falconiformes	Falconidae
AKCR01027958	AQP0	Rock pigeon	*Columba livia*	Aves	Columbiformes	Columbidae
NP_989597	AQP0	Chicken	*Gallus gallus*	Aves	Galliformes	Phasianidae
AKHW01052381	AQP0	American alligator	*Alligator mississippiensis*	Archosauria	Crocodylia	Alligatoridae
AVPB01160521/AVPB01160520	AQP0	Chinese alligator	*Alligator sinensis*	Archosauria	Crocodylia	Alligatoridae
AGCU01140836/ENSPSIP00000003487	AQP0	Chinese softshell turtle	*Pelodiscus sinensis*	Sauropsida	Testudines	Trionychidae
AJIM01220994	AQP0	Green seaturtle	*Chelonia mydas*	Sauropsida	Testudines	Cheloniidae
JW429802	AQP0	Red-eared slider turtle	*Trachemys scripta elegans*	Sauropsida	Testudines	Emydidae
AHGY01503377	AQP0	Western painted turtle	*Chrysemys picta bellii*	Sauropsida	Testudines	Emydidae
AZIM01000024	AQP0	King cobra	*Ophiophagus hannah*	Lepidosauria	Squamata	Elapidae
AEQU02069336	AQP0	Burmese python	*Python molurus bivittatus*	Lepidosauria	Squamata	Pythonidae
ENSACAP00000016272	AQP0	Green anole	*Anolis carolinensis*	Lepidosauria	Squamata	Iguanidae
Q06019/CAA40291	AQP0	Northern leopard frog	*Rana pipiens*	Amphibia	Anura	Ranidae
JN0557	AQP0	African clawed frog	*Xenopus laevis*	Amphibia	Anura	Pipidae
ENSXETP00000047158/AAMC02007443	AQP0	Western clawed frog	*Xenopus (Silurana) tropicalis*	Amphibia	Anura	Pipidae
GAQK01077627	AQP0	Chinese salamander	*Hynobius chinensis*	Amphibia	Caudata	Hynobiidae
AB513618	Aqp0	West African lungfish	*Protopterus annectens*	Dipnoi	Lepidosireniformes	Protopteridae
BAH98062	Aqp0	Australian lungfish	*Neoceratodus forsteri*	Dipnoi	Ceratodontiformes	Ceratodontidae
AFYH01001823/ENSLACP00000021649	Aqp0	Coelacanth	*Latimeria chalumnae*	Actinistia	Coelacanthiformes	Coelacanthidae
ENSTRUP00000040095	Aqp0a	Torafugu	*Takifugu rubripes*	Acanthopterygii	Tetraodontiformes	Tetraodontidae
AOOT01089397	Aqp0a	Sansaifugu	*Takifugu flavidus*	Acanthopterygii	Tetraodontiformes	Tetraodontidae
ENSTNIP00000018156	Aqp0a	Green-spotted pufferfish	*Tetraodon nigroviridis*	Acanthopterygii	Tetraodontiformes	Tetraodontidae
AGRG01027418/AGRG01027419	Aqp0a	Tongue sole	*Cynoglossus semilaevis*	Acanthopterygii	Pleuronectiformes	Cynoglossidae
AGT57405	Aqp0a	Gilthead seabream	*Sparus aurata*	Acanthopterygii	Perciformes	Sparidae
AGTA02027801	Aqp0a	Zebra mbuna	*Maylandia zebra*	Acanthopterygii	Perciformes	Cichlidae
ABPL01063606	Aqp0a	Golden mbuna	*Melanochromis auratus*	Acanthopterygii	Perciformes	Cichlidae
AFNX01017056	Aqp0a	Red mwanza	*Pundamilia nyererei*	Acanthopterygii	Perciformes	Cichlidae
AFNY01015535	Aqp0a	Lyretail cichlid	*Neolamprologus brichardi*	Acanthopterygii	Perciformes	Cichlidae
AFNZ01002315	Aqp0a	Burton's mouthbrooder	*Haplochromis burtoni*	Acanthopterygii	Perciformes	Cichlidae
ENSONIP00000015031	Aqp0a	Nile tilapia	*Oreochromis niloticus*	Acanthopterygii	Perciformes	Cichlidae
JL566631/JL556852	Aqp0a	Blue Tanganyika featherfin	*Ophthalmotilapia ventralis*	Acanthopterygii	Perciformes	Cichlidae
BADN01059801	Aqp0a	Pacific bluefin tuna	*Thunnus orientalis*	Acanthopterygii	Perciformes	Scombridae
AWGY01060387	Aqp0a	Sablefish	*Anoplopoma fimbria*	Acanthopterygii	Scorpaeniformes	Anoplopomatidae
AUPQ01060261	Aqp0a	Flag rockfish	*Sebastes rubrivinctus*	Acanthopterygii	Scorpaeniformes	Sebastidae
AUPR01049683	Aqp0a	Tiger rockfish	*Sebastes rubrivinctus*	Acanthopterygii	Scorpaeniformes	Sebastidae
ENSGACP00000011234	Aqp0a	Three-spined stickleback	*Gasterosteus aculeatus*	Acanthopterygii	Gasterosteiformes	Gasterosteidae
ENSORLP00000025045	Aqp0a	Japanese medaka	*Oryzias latipes*	Acanthopterygii	Beloniformes	Adrianichthyidae
GAIB01003927	Aqp0a	Turquoise killifish	*Nothobranchius furzeri*	Acanthopterygii	Cyprinodontiformes	Nothobranchiidae
AAF04146	Aqp0a	Common mummichog	*Fundulus heteroclitus*	Acanthopterygii	Cyprinodontiformes	Fundulidae
AYCK01006676	Aqp0a	Amazon molly	*Poecilia formosa*	Acanthopterygii	Cyprinodontiformes	Poeciliidae
ENSXMAP00000010636	Aqp0a	Southern platyfish	*Xiphophorus maculatus*	Acanthopterygii	Cyprinodontiformes	Poeciliidae
ENSGMOP00000008858	Aqp0a	Atlantic cod	*Gadus morhua*	Paracanthopterygii	Gadiformes	Gadidae
AFQ36921	Aqp0a	Chum salmon	*Oncorhynchus keta*	Protacanthopterygii	Salmoniformes	Salmonidae
CCAF010074982	Aqp0a1	Rainbow trout	*Oncorhynchus mykiss*	Protacanthopterygii	Salmoniformes	Salmonidae
AGKD01164501	Aqp0a1	Atlantic salmon	*Salmo salar*	Protacanthopterygii	Salmoniformes	Salmonidae
CCAF010023966	Aqp0a2	Rainbow trout	*Oncorhynchus mykiss*	Protacanthopterygii	Salmoniformes	Salmonidae
AGKD01193927	Aqp0a2	Atlantic salmon	*Salmo salar*	Protacanthopterygii	Salmoniformes	Salmonidae
DT359524	Aqp0a	Fathead minnow	*Pimephales promelas*	Ostariophysi	Cypriniformes	Cyprinidae
GAHL01069030/GAHL01057104	Aqp0a	Blind goldenline barbel	*Sinocyclocheilus anophthalmus*	Ostariophysi	Cypriniformes	Cyprinidae
FJ666326/ENSDARP00000054237	Aqp0a	Zebrafish	*Danio rerio*	Ostariophysi	Cypriniformes	Cyprinidae
APWO01111469	Aqp0a	Mexican tetra	*Astyanax mexicanus*	Ostariophysi	Characiformes	Characidae
JT420530	Aqp0a	Channel catfish	*Ictalurus punctatus*	Ostariophysi	Siluriformes	Ictaluridae
GAGX01323253	Aqp0a	Brown bullhead	*Ameiurus nebulosus*	Ostariophysi	Siluriformes	Ictaluridae
AVPY01076505/AVPY01076506	Aqp0a	Japanese eel	*Anguilla japonica*	Elopomorpha	Angilliformes	Anguillidae
ENSTRUP00000009786	Aqp0b	Torafugu	*Takifugu rubripes*	Acanthopterygii	Tetraodontiformes	Tetraodontidae
AOOT01015865	Aqp0b	Sansaifugu	*Takifugu flavidus*	Acanthopterygii	Tetraodontiformes	Tetraodontidae
ENSTNIP00000015570	Aqp0b	Green-spotted pufferfish	*Tetraodon nigroviridis*	Acanthopterygii	Tetraodontiformes	Tetraodontidae
AGRG01001238/AGRG01001239	Aqp0b	Tongue sole	*Cynoglossus semilaevis*	Acanthopterygii	Pleuronectiformes	Cynoglossidae

AGTA02010417	Aqp0b	Zebra mbuna	*Maylandia zebra*	Acanthopterygii	Perciformes	Cichlidae
AFNX01016365	Aqp0b	Red mwanza	*Pundamilia nyererei*	Acanthopterygii	Perciformes	Cichlidae
AFNY01042433	Aqp0b	Lyretail cichlid	*Neolamprologus brichardi*	Acanthopterygii	Perciformes	Cichlidae
AFNZ01014050	Aqp0b	Burton's mouthbrooder	*Haplochromis burtoni*	Acanthopterygii	Perciformes	Cichlidae
ENSONIP00000021524	Aqp0b	Nile tilapia	*Oreochromis niloticus*	Acanthopterygii	Perciformes	Cichlidae
ABPN01017241	Aqp0b	Tigerfish	*Rhamphochromis esox*	Acanthopterygii	Perciformes	Cichlidae
JL556709	Aqp0b	Blue Tanganyika featherfin	*Ophthalmotilapia ventralis*	Acanthopterygii	Perciformes	Cichlidae
BADN01085846	Aqp0b	Pacific bluefin tuna	*Thunnus orientalis*	Acanthopterygii	Perciformes	Scombridae
AWGY01185229	Aqp0b	Sablefish	*Anoplopoma fimbria*	Acanthopterygii	Scorpaeniformes	Anoplopomatidae
AUPQ01098549	Aqp0b	Flag rockfish	*Sebastes rubrivinctus*	Acanthopterygii	Scorpaeniformes	Sebastidae
ENSGACP00000020083	Aqp0b	Three-spined stickleback	*Gasterosteus aculeatus*	Acanthopterygii	Gasterosteiformes	Gasterosteidae
ENSORLP00000015228	Aqp0b	Medaka	*Oryzias latipes*	Acanthopterygii	Beloniformes	Adrianichthyidae
GAIB01100693	Aqp0b	Turquoise killifish	*Nothobranchius furzeri*	Acanthopterygii	Cyprinodontiformes	Nothobranchiidae
ES379168/ES375646	Aqp0b	Guppy	*Poecilia reticulata*	Acanthopterygii	Cyprinodontiformes	Poeciliidae
AYCK01011345	Aqp0b	Amazon molly	*Poecilia formosa*	Acanthopterygii	Cyprinodontiformes	Poeciliidae
ENSXMAP00000017496	Aqp0b	Southern platyfish	*Xiphophorus maculatus*	Acanthopterygii	Cyprinodontiformes	Poeciliidae
ENSGMOP00000001121	Aqp0b	Atlantic cod	*Gadus morhua*	Paracanthopterygii	Gadiformes	Gadidae
CCAF010085063	Aqp0b1	Rainbow trout	*Oncorhynchus mykiss*	Protacanthopterygii	Salmoniformes	Salmonidae
AGKD01047775	Aqp0b1	Atlantic salmon	*Salmo salar*	Protacanthopterygii	Salmoniformes	Salmonidae
CCAF010053309	Aqp0b2	Rainbow trout	*Oncorhynchus mykiss*	Protacanthopterygii	Salmoniformes	Salmonidae
AGKD01117189	Aqp0b2	Atlantic salmon	*Salmo salar*	Protacanthopterygii	Salmoniformes	Salmonidae
GAHL01044644	Aqp0b	Blind goldenline barbel	*Sinocyclocheilus anophthalmus*	Ostariophysi	Cypriniformes	Cyprinidae
FJ655389/ENSDARP00000005361	Aqp0b	Zebrafish	*Danio rerio*	Ostariophysi	Cypriniformes	Cyprinidae
APWO01019346/FO255265	Aqp0b	Mexican tetra	*Astyanax mexicanus*	Ostariophysi	Characiformes	Characidae
GAGX01013963	Aqp0b	Brown bullhead	*Ameiurus nebulosus*	Ostariophysi	Siluriformes	Ictaluridae
AVPY01025853	Aqp0b	Japanese eel	*Anguilla japonica*	Elopomorpha	Angilliformes	Anguillidae
AHAT01018943/AHAT01018944	Aqp0	Spotted gar	*Lepisosteus oculatus*	Holostei	Semionotiformes	Lepisosteidae
KJ784515	Aqp0	Smaller spotted catshark	*Scyliorhinus canicula*	Chondrichthyes	Carcharhiniformes	Scyliorhinidae
AESE011108195/AESE012538798/AESE010060442	Aqp0	Little skate	*Leucoraja erinacea*	Chondrichthyes	Rajiformes	Rajidae
AAVX02044963/ AAVX02037708	Aqp0	Ghost shark	*Callorhinchus milii*	Chondrichthyes	Chimaeriformes	Callorhinchidae
KJ784520/AEFG01022836/ENSPMAP00000004928 / ENSPMAP00000004946	Aqp01	Sea lamprey	*Petromyzon marinus*	Hyperoartia	Petromyzontiformes	Petromyzontidae
APJL01031881/APJL01031882	Aqp01	Arctic lamprey	*Lethenteron camtschaticum*	Hyperoartia	Petromyzontiformes	Petromyzontidae

AQP1

ENSP00000311165	AQP1	Human	*Homo sapiens*	Euarchontoglires	Primates	Hominidae
ENSPTRP00000032543	AQP1	Chimpanzee	*Pan troglodytes*	Euarchontoglires	Primates	Hominidae
ENSGGOP00000009650	AQP1	Western lowland gorilla	*Gorilla gorilla gorilla*	Euarchontoglires	Primates	Hominidae
ENSPPYP00000019805	AQP1	Sumatran orangutan	*Pongo abelii*	Euarchontoglires	Primates	Hominidae
ENSNLEP00000018458	AQP1	Northern white-cheeked gibbon	*Nomascus leucogenys*	Euarchontoglires	Primates	Hylobatidae
ENSMMUP00000015441	AQP1	Rhesus macaque	*Macaca mulatta*	Euarchontoglires	Primates	Cercopithecidae
NP_001270465	AQP1	Crab-eating macaque	*Macaca fascicularis*	Euarchontoglires	Primates	Cercopithecidae
AHZZ01012709	AQP1	Olive baboon	*Papio anubis*	Euarchontoglires	Primates	Cercopithecidae
ENSCJAP00000043678	AQP1	White-tufted-ear marmoset	*Callithrix jacchus*	Euarchontoglires	Primates	Cebidae
XP_003935232	AQP1	Bolivian squirrel monkey	*Saimiri boliviensis boliviensis*	Euarchontoglires	Primates	Cebidae
ENSMICP00000011114	AQP1	Gray mouse lemur	*Microcebus murinus*	Euarchontoglires	Primates	Cheirogaleidae
XP_003788602/ENSOGAP00000001701	AQP1	Small-eared galago/Bushbaby	*Otolemur garnettii*	Euarchontoglires	Primates	Galagidae
ENSTBEP00000003384	AQP1	Northern tree shrew	*Tupaia belangeri*	Euarchontoglires	Scandentia	Tupaiidae
ELW67938	AQP1	Chinese tree shrew	*Tupaia chinensis*	Euarchontoglires	Scandentia	Tupaiidae
ENSMUSP00000004774	AQP1	Mouse	*Mus musculus*	Euarchontoglires	Rodentia	Muridae
ENSRNOP00000015692	AQP1	Norway rat	*Rattus norvegicus*	Euarchontoglires	Rodentia	Muridae
AAW47637	AQP1	Spinifex hopping mouse	*Notomys alexis*	Euarchontoglires	Rodentia	Muridae
XP_003503971	AQP1	Chinese hamster	*Cricetulus griseus*	Euarchontoglires	Rodentia	Cricetidae
XP_005360862	AQP1	Prairie vole	*Microtus ochrogaster*	Euarchontoglires	Rodentia	Cricetidae
AKZC01053220	AQP1	Lesser Egyptian jerboa	*Jaculus jaculus*	Euarchontoglires	Rodentia	Dipodidae
ENSDORP00000006023	AQP1	Ord's kangaroo rat	*Dipodomys ordii*	Euarchontoglires	Rodentia	Heteromyidae
XP_004626583	AQP1	Degu	*Octodon degus*	Euarchontoglires	Rodentia	Octodontidae
ENSCPOP00000010240	AQP1	Domestic guinea pig	*Cavia porcellus*	Euarchontoglires	Rodentia	Caviidae
EHB16106	AQP1	Naked mole-rat	*Heterocephalus glaber*	Euarchontoglires	Rodentia	Bathyergidae
ENSSTOP00000013889	AQP1	Thirteen-lined ground squirrel	*Ictidomys tridecemlineatus*	Euarchontoglires	Rodentia	Sciuridae
XP_005377565	AQP1	Long-tailed chinchilla	*Chinchilla lanigera*	Euarchontoglires	Rodentia	Chinchillidae
ENSOPRP00000005980	AQP1	American pika	*Ochotona princeps*	Laurasiatheria	Lagomorpha	Ochotonidae
ENSOCUP00000018388	AQP1	Rabbit	*Oryctolagus cuniculus*	Laurasiatheria	Lagomorpha	Leporidae
AANN01240335	AQP1	Western European hedgehog	*Erinaceus europaeus*	Laurasiatheria	Insectivora	Erinaceinae
AALT02063098	AQP1	European shrew	*Sorex araneus*	Laurasiatheria	Insectivora	Soricidae
AJFV01015306	AQP1	Star-nosed mole	*Condylura cristata*	Laurasiatheria	Insectivora	Talpidae
ENSPVAP00000009966	AQP1	Large flying fox/Megabat	*Pteropus vampyrus*	Laurasiatheria	Chiroptera	Pteropodidae
ELK12636	AQP1	Black flying fox	*Pteropus alecto*	Laurasiatheria	Chiroptera	Pteropodidae
JAA46116	AQP1	Common vampire bat	*Desmodus rotundus*	Laurasiatheria	Chiroptera	Phyllostomidae
ENSMLUP00000016202 /XP_006088797	AQP1	Little brown bat/Microbat	*Myotis lucifugus*	Laurasiatheria	Chiroptera	Vespertilionidae
XP_005863006	AQP1	Brandt's bat	*Myotis brandtii*	Laurasiatheria	Chiroptera	Vespertilionidae
ALEH01007947/ALEH01007945	AQP1	Big brown bat	*Eptesicus fuscus*	Laurasiatheria	Chiroptera	Vespertilionidae
AWHA01016190/AWHA01225982	AQP1	Greater horseshoe bat	*Rhinolophus ferrumequinum*	Laurasiatheria	Chiroptera	Rhinolophinae
NP_001009194	AQP1	Sheep	*Ovis aries*	Laurasiatheria	Ruminantia	Bovidae
AJPT01046005/AJPT01046004 /XP_005679299	AQP1	Goat	*Capra hircus*	Laurasiatheria	Ruminantia	Bovidae

XP_005981267	AQP1	Chiru	*Pantholops hodgsonii*	Laurasiatheria	Ruminantia	Bovidae
ENSBTAP00000000993	AQP1	Cow	*Bos taurus*	Laurasiatheria	Ruminantia	Bovidae
ELR61157	AQP1	Yak	*Bos mutus*	Laurasiatheria	Ruminantia	Bovidae
AGFL01038270/AGFL01038269	AQP1	Zebu	*Bos indicus*	Laurasiatheria	Ruminantia	Bovidae
ENSTTRP00000000321	AQP1	Bottlenosed dolphin	*Tursiops truncatus*	Laurasiatheria	Cetacea	Delphinidae
AGV55459	AQP1	Indo-pacific humpbacked dolphin	*Sousa chinensis*	Laurasiatheria	Cetacea	Delphinidae
AUPI01120507/AUPI01120508/AUPI01120509	AQP1	Yangtze River dolphin	*Lipotes vexillifer*	Laurasiatheria	Cetacea	Delphinidae
AGV55460	AQP1	Risso's dolphin	*Grampus griseus*	Laurasiatheria	Cetacea	Delphinidae
ANOL02024324	AQP1	Killer whale	*Orcinus orca*	Laurasiatheria	Cetacea	Delphinidae
AGV55465	AQP1	Beluga whale	*Delphinapterus leucas*	Laurasiatheria	Cetacea	Monodontidae
AWZP01108580	AQP1	Sperm whale	*Physeter catodon*	Laurasiatheria	Cetacea	Physeteridae
AGV55464	AQP1	Dwarf sperm whale	*Kogia sima*	Laurasiatheria	Cetacea	Physeteridae
AGV55463	AQP1	Blainville's beaked whale	*Mesoplodon densirostris*	Laurasiatheria	Cetacea	Ziphiidae
ATDI01064192/ATDI01064190/AGV55462	AQP1	Minke whale	*Balaenoptera acutorostrata*	Laurasiatheria	Cetacea	Balaenopteridae
AGV55461	AQP1	Omura's baleen whale	*Balaenoptera omurai*	Laurasiatheria	Cetacea	Balaenopteridae
NM_214454	AQP1	Pig	*Sus scrofa*	Laurasiatheria	Suina	Suidae
ABRR02039783	AQP1	Alpaca	*Vicugna pacos*	Laurasiatheria	Tylopoda	Camelidae
AGVR01037331	AQP1	Wild Bactrian camel	*Camelus ferus*	Laurasiatheria	Tylopoda	Camelidae
GAEB01028929	AQP1	Arabian camel	*Camelus dromedarius*	Laurasiatheria	Tylopoda	Camelidae
ENSECAP00000007138	AQP1	Horse	*Equus caballus*	Laurasiatheria	Perissodactyla	Equidae
AKZM01001957	AQP1	Southern white rhinocerus	*Ceratotherium simum simum*	Laurasiatheria	Perissodactyla	Rhinocerotidae
ENSAMEP00000007112	AQP1	Giant panda	*Ailuropoda melanoleuca*	Laurasiatheria	Carnivora	Ursidae
GAJD01001381	AQP1	Polar bear	*Ursus maritimus*	Laurasiatheria	Carnivora	Ursidae
ENSCAFP00000004614	AQP1	Dog	*Canis lupus familiaris*	Laurasiatheria	Carnivora	Canidae
ENSMPUP00000005125	AQP1	Domestic ferret	*Mustela putorius furo*	Laurasiatheria	Carnivora	Mustelidae
ENSFCAP00000002341	AQP1	Domestic cat	*Felis catus*	Laurasiatheria	Carnivora	Felidae
ATCQ01104586/ATCQ01104585	AQP1	Amur tiger	*Panthera tigris altaica*	Laurasiatheria	Carnivora	Felidae
APMU01133821	AQP1	Weddell seal	*Leptonychotes weddellii*	Laurasiatheria	Carnivora	Phocidae
ANOP01050556	AQP1	Pacific walrus	*Odobenus rosmarus divergens*	Laurasiatheria	Carnivora	Odobenidae
ENSLAFP00000008524	AQP1	African savanna elephant	*Loxodonta africana*	Afrotheria	Proboscidea	Elephantidae
AHIN01109068/AHIN01109067	AQP1	Florida manatee	*Trichechus manatus latirostris*	Afrotheria	Sirenia	Trichechidae
ENSPCAP00000014912	AQP1	Cape rock hyrax	*Procavia capensis*	Afrotheria	Hyracoidea	Procaviidae
ENSETEP00000003377	AQP1	Lesser hedgehog tenrec	*Echinops telfairi*	Afrotheria	Afrosoricida	Tenrecidae
AMDV01022493	AQP1	Cape golden mole	*Chrysochloris asiatica*	Afrotheria	Afrosoricida	Chrysochloridae
AMGZ01265485	AQP1	Cape elephant shrew	*Elephantulus edwardii*	Afrotheria	Macroscelidea	Macroscelididae
ALYB01018035	AQP1	Ardvaark	*Orycteropus afer afer*	Afrotheria	Tubulidentata	Orycteropodidae
AAGV03054280/AAGV03054281/ ENSDNOP00000008167	AQP1	Nine-banded armadillo	*Dasypus novemcinctus*	Xenarthra	Cingulata	Dasypodidae
ENSMEUP00000007054	AQP1	Tammar wallaby	*Macropus eugenii*	Metatheria	Diprotodontia	Macropodidae
AFEY01321846	AQP1	Tasmanian devil	*Sarcophilus harrisii*	Metatheria	Dasyuromorphia	Dasyuridae
ENSMODP00000026101	AQP1	Gray short-tailed opossum	*Monodelphis domestica*	Metatheria	Didelphimorphia	Didelphidae
GENSCAN00000084522/ENSOANP00000020359	AQP1	Platypus	*Ornithorhynchus anatinus*	Prototheria	Monotremata	Ornithorhynchidae
AGAI01061621	AQP1	Budgerigar	*Melopsittacus undulatus*	Aves	Psittaciformes	Psittacidae
AOCU01097369/AOCU01095546/AOCU01018583	AQP1	Puerto Rican parrot	*Amazona vittata*	Aves	Psittaciformes	Psittacidae
AMXX01105025/AMXX01130217/AOUJ01455056/ AOUJ01404714	AQP1	Scarlet macaw	*Ara macao*	Aves	Psittaciformes	Psittacidae
AAV65290	AQP1	House sparrow	*Passer domesticus*	Aves	Passeriformes	Passeridae
JV166424	AQP1	Duck-eyed junco	*Junco hyemalis*	Aves	Passeriformes	Fringillidae
CAVT010009167/CAVT010009166	AQP1	Common canary	*Serinus canaria*	Aves	Passeriformes	Fringillidae
AKZB01029466/AKZB01029467	AQP1	Medium ground finch	*Geospiza fortis*	Aves	Passeriformes	Fringillidae
ENSTGUP00000000351	AQP1	Zebra finch	*Taeniopygia guttata*	Aves	Passeriformes	Estrildidae
ANZD01023602	AQP1	Tibetan ground-tit	*Pseudopodoces humilis*	Aves	Passeriformes	Paridae
AGTO01021544	AQP1	Collared flycatcher	*Ficedula albicollis*	Aves	Passeriformes	Muscicapidae
AKMT01025673/AKMT01025674	AQP1	Peregrin falcon	*Falco peregrinus*	Aves	Falconiformes	Falconidae
AKMU01075516	AQP1	Saker falcon	*Falco cherrug*	Aves	Falconiformes	Falconidae
EMC82019	AQP1	Rock pigeon	*Columba livia*	Aves	Columbiformes	Columbidae
ENSAPLP00000011568	AQP1	Mallard	*Anas platyrhynchos*	Aves	Anseriformes	Anatidae
AAU07832	AQP1	Common quail	*Coturnix coturnix*	Aves	Galliformes	Phasianidae
ENSMGAP00000001252	AQP1	Turkey	*Meleagris gallopavo*	Aves	Galliformes	Phasianidae
ENSGALP00000008350	AQP1	Chicken	*Gallus gallus*	Aves	Galliformes	Phasianidae
AKHW01023235/AKHW01023236	AQP1	American alligator	*Alligator mississippiensis*	Archosauria	Crocodylia	Alligatoridae
AVPB01075988/AVPB01075985	AQP1	Chinese alligator	*Alligator sinensis*	Archosauria	Crocodylia	Alligatoridae
AGCU01068649/ENSPSIP00000016282	AQP1	Chinese softshell turtle	*Pelodiscus sinensis*	Sauropsida	Testudines	Trionychidae
AJIM01232803/EMP29429	AQP1	Green seaturtle	*Chelonia mydas*	Sauropsida	Testudines	Cheloniidae
JW308388	AQP1	Red-eared slider turtle	*Trachemys scripta elegans*	Sauropsida	Testudines	Emydidae
AHGY01020053/AHGY01020054	AQP1	Western painted turtle	*Chrysemys picta bellii*	Sauropsida	Testudines	Emydidae
ETE59872/AZIM01042903	AQP1	King cobra	*Ophiophagus hannah*	Lepidosauria	Squamata	Elapidae
AFJ49442	AQP1	Eastern diamondback rattlesnake	*Crotalus adamanteus*	Lepidosauria	Squamata	Viperidae
GAAZ01000199	AQP1	Timber rattlesnake	*Crotalus horridus*	Lepidosauria	Squamata	Viperidae
AEQU02121829/AEQU02121834/AEQU02121836	AQP1	Burmese python	*Python molurus bivittatus*	Lepidosauria	Squamata	Pythonidae
ENSACAP00000005702	AQP1	Green anole	*Anolis carolinensis*	Lepidosauria	Squamata	Iguanidae
AAC69693	AQP1	Marine toad	*Rhinella marina*	Amphibia	Anura	Hylidae
ABC98208	AQP1	Southern gray treefrog	*Hyla chrysoscelis*	Amphibia	Anura	Hylidae
BAC07470	AQP1	Japanese treefrog	*Hyla japonica*	Amphibia	Anura	Hylidae

GAEI01039371/GAEI01032191	AQP1	Pacific treefrog	*Pseudacris regilla*	Amphibia	Anura	Hylidae
P50501	AQP1	Edible frog	*Pelophylax esculentus*	Amphibia	Anura	Ranidae
ACK75671	AQP1	Dark-spotted frog	*Pelophylax nigromaculatus*	Amphibia	Anura	Ranidae
ACM51137	AQP1	Asiatic toad	*Bufo gargarizans*	Amphibia	Anura	Bufonidae
JP286557	AQP1	Carvalho's Surinam toad	*Pipa carvalhoi*	Amphibia	Anura	Pipidae
NP_001085391	AQP1	African clawed frog	*Xenopus laevis*	Amphibia	Anura	Pipidae
NP_001005829/AAMC02024113	AQP1	Western clawed frog	*Xenopus (Silurana) tropicalis*	Amphibia	Anura	Pipidae
GAQK01031218	AQP1	Chinese salamander	*Hynobius chinensis*	Amphibia	Caudata	Hynobiidae
BAI48049	Aqp1	West African lungfish	*Protopterus annectens*	Dipnoi	Lepidosireniformes	Protopteridae
AFYH01166611/AFYH01284055/ ENSLACP00000008076	Aqp1	Coelacanth	*Latimeria chalumnae*	Actinistia	Coelacanthiformes	Coelacanthidae
GAPS01057965	Aqp1	Menado coelacanth	*Latimeria menadoensis*	Actinistia	Coelacanthiformes	Coelacanthidae
ENSTRUP00000034715	Aqp1aa	Torafugu	*Takifugu rubripes*	Acanthopterygii	Tetraodontiformes	Tetraodontidae
AOOT01001870/AOOT01001869	Aqp1aa	Sansaifugu	*Takifugu flavidus*	Acanthopterygii	Tetraodontiformes	Tetraodontidae
ADG86337	Aqp1aa	Mefugu	*Takifugu obscurus*	Acanthopterygii	Tetraodontiformes	Tetraodontidae
ENSTNIP00000011311	Aqp1aa	Green-spotted pufferfish	*Tetraodon nigroviridis*	Acanthopterygii	Tetraodontiformes	Tetraodontidae
HQ185294	Aqp1aa	Atlantic halibut	*Hippoglossus hippoglossus*	Acanthopterygii	Pleuronectiformes	Pleuronectidae
FY382720/FE042295	Aqp1aa	Japanese flounder	*Paralichthys olivaceus*	Acanthopterygii	Pleuronectiformes	Paralichthyidae
GAAQ01006950	Aqp1aa	Dover sole	*Solea solea*	Acanthopterygii	Pleuronectiformes	Soleidae
ABK20157/AGRG01004606	Aqp1aa	Senegalese sole	*Solea senegalensis*	Acanthopterygii	Pleuronectiformes	Soleidae
ADG21868	Aqp1aa	Tongue sole	*Cynoglossus semilaevis*	Acanthopterygii	Pleuronectiformes	Cynoglossidae
AAV34610	Aqp1aa	Gilthead seabream	*Sparus aurata*	Acanthopterygii	Perciformes	Sparidae
AEG78286	Aqp1aa	Silver seabream	*Rhabdosargus sarba*	Acanthopterygii	Perciformes	Sparidae
AEU08496	Aqp1aa	White seabream	*Diplodus sargus*	Acanthopterygii	Perciformes	Sparidae
ABO38816	Aqp1aa	Black porgy	*Acanthopagrus schlegelii*	Acanthopterygii	Perciformes	Sparidae
ADO60022	Aqp1aa	Fire clownfish	*Amphiprion melanopus*	Acanthopterygii	Perciformes	Pomacentridae
GW669182	Aqp1aa	Mi-iuy croaker	*Miichthys miiuy*	Acanthopterygii	Perciformes	Sciaenidae
AAV34607	Aqp1aa	Black seabass	*Centropristis striata*	Acanthopterygii	Perciformes	Serranidae
GO570422	Aqp1aa	Yellow perch	*Perca flavescens*	Acanthopterygii	Perciformes	Percidae
ABI95464	Aqp1aa	European seabass	*Dicentrarchus labrax*	Acanthopterygii	Perciformes	Moronidae
AGTA02006474	Aqp1aa	Zebra mbuna	*Maylandia zebra*	Acanthopterygii	Perciformes	Cichlidae
AFNX01030543	Aqp1aa	Red mwanza	*Pundamilia nyererei*	Acanthopterygii	Perciformes	Cichlidae
AFNY01028576	Aqp1aa	Lyretail cichlid	*Neolamprologus brichardi*	Acanthopterygii	Perciformes	Cichlidae
AFNZ01000497	Aqp1aa	Burton's mouthbrooder	*Haplochromis burtoni*	Acanthopterygii	Perciformes	Cichlidae
ENSONIP00000011870/AERX01000980	Aqp1aa	Nile tilapia	*Oreochromis niloticus*	Acanthopterygii	Perciformes	Cichlidae
GAJK01038543	Aqp1aa	Black-faced blenny	*Tripterygion delaisi*	Acanthopterygii	Perciformes	Tripterygiidae
AGF30363	Aqp1aa	Climbing perch	*Anabas testudineus*	Acanthopterygii	Perciformes	Anabantidae
FE196516	Aqp1aa	Antarctic toothfish	*Dissostichus mawsoni*	Acanthopterygii	Perciformes	Nototheniidae
BADN01032054	Aqp1aa	Pacific bluefin tuna	*Thunnus orientalis*	Acanthopterygii	Perciformes	Scombridae
GAJJ01012677/GAJJ01012676/AWGY01116372/ AWGY01076552	Aqp1aa	Sablefish	*Anoplopoma fimbria*	Acanthopterygii	Scorpaeniformes	Anoplopomatidae
EW976757	Aqp1aa	Grass rockfish	*Sebastes rastrelliger*	Acanthopterygii	Scorpaeniformes	Sebastidae
AUPQ01097238	Aqp1aa	Flag rockfish	*Sebastes rubrivinctus*	Acanthopterygii	Scorpaeniformes	Sebastidae
AUPR01043427	Aqp1aa	Tiger rockfish	*Sebastes nigrocinctus*	Acanthopterygii	Scorpaeniformes	Sebastidae
ENSGACP00000022980	Aqp1aa	Three-spined stickleback	*Gasterosteus aculeatus*	Acanthopterygii	Gasterosteiformes	Gasterosteidae
ENSORLP00000022052	Aqp1aa	Japanese medaka	*Oryzias latipes*	Acanthopterygii	Beloniformes	Adrianichthyidae
ACI49538	Aqp1aa	Common mummichog	*Fundulus heteroclitus*	Acanthopterygii	Cyprinodontiformes	Fundulidae
GAIB01037056	Aqp1aa	Turquoise killifish	*Nothobranchius furzeri*	Acanthopterygii	Cyprinodontiformes	Nothobranchiidae
AYCK01003119	Aqp1aa	Amazon molly	*Poecilia formosa*	Acanthopterygii	Cyprinodontiformes	Poeciliidae
ENSXMAP00000005883	Aqp1aa	Southern platyfish	*Xiphophorus maculatus*	Acanthopterygii	Cyprinodontiformes	Poeciliidae
ENSGMOP00000012296	Aqp1aa	Atlantic cod	*Gadus morhua*	Paracanthopterygii	Gadiformes	Gadidae
ACO09149	Aqp1aa	Rainbow smelt	*Osmerus mordax*	Protacanthopterygii	Osmeriformes	Osmeridae
CA363480/CA378544/CCAF010002588	Aqp1aa1	Rainbow trout	*Oncorhynchus mykiss*	Protacanthopterygii	Salmoniformes	Salmonidae
EV376188	Aqp1aa	Sockeye salmon	*Oncorhynchus nerka*	Protacanthopterygii	Salmoniformes	Salmonidae
AGKD01005533/ACI66426	Aqp1aa1	Atlantic salmon	*Salmo salar*	Protacanthopterygii	Salmoniformes	Salmonidae
CCAF010038803	Aqp1aa2	Rainbow trout	*Oncorhynchus mykiss*	Protacanthopterygii	Salmoniformes	Salmonidae
AGKD01009244/ACI67627	Aqp1aa2	Atlantic salmon	*Salmo salar*	Protacanthopterygii	Salmoniformes	Salmonidae
ACO13816	Aqp1aa	Northern pike	*Esox lucius*	Neognathi	Esociformes	Esocidae
DT351671	Aqp1aa	Fathead minnow	*Pimephales promelas*	Ostariophysi	Cypriniformes	Cyprinidae
GAHO01032101	Aqp1aa	Small gill opening goldenline barbel	*Sinocyclocheilus angustiporus*	Ostariophysi	Cypriniformes	Cyprinidae
GAHL01124027/GAHL01094792	Aqp1aa	Blind goldenline barbel	*Sinocyclocheilus anophthalmus*	Ostariophysi	Cypriniformes	Cyprinidae
AY626937/ENSDARP00000036702	Aqp1aa	Zebrafish	*Danio rerio*	Ostariophysi	Cypriniformes	Cyprinidae
APWO01062007/APWO01062008	Aqp1aa	Mexican tetra	*Astyanax mexicanus*	Ostariophysi	Characiformes	Characidae
GAGX01004978	Aqp1aa	Brown bullhead	*Ameiurus nebulosus*	Ostariophysi	Siluriformes	Ictaluridae
JT484108/FD300713	Aqp1aa	Channel catfish	*Ictalurus punctatus*	Ostariophysi	Siluriformes	Ictaluridae
CAD92027	Aqp1aa	European eel	*Anguilla anguilla*	Elopomorpha	Anguilliformes	Anguillidae
BAC82110	Aqp1aa1	Japanese eel	*Anguilla japonica*	Elopomorpha	Anguilliformes	Anguillidae
BAC82109/ AVPY01347513	Aqp1aa2	Japanese eel	*Anguilla japonica*	Elopomorpha	Anguilliformes	Anguillidae
ENSTRUP00000034674	Aqp1ab	Torafugu	*Takifugu rubripes*	Acanthopterygii	Tetraodontiformes	Tetraodontidae
AOOT01001869	Aqp1ab	Sansaifugu	*Takifugu flavidus*	Acanthopterygii	Tetraodontiformes	Tetraodontidae
ENSTNIP00000001573	Aqp1ab	Green-spotted pufferfish	*Tetraodon nigroviridis*	Acanthopterygii	Tetraodontiformes	Tetraodontidae
HQ185295	Aqp1ab	Atlantic halibut	*Hippoglossus hippoglossus*	Acanthopterygii	Pleuronectiformes	Pleuronectidae
AAV34612	Aqp1ab	Senegalese sole	*Solea senegalensis*	Acanthopterygii	Pleuronectiformes	Soleidae
AGRG01004607	Aqp1ab1	Tongue sole	*Cynoglossus semilaevis*	Acanthopterygii	Pleuronectiformes	Cynoglossidae

AGRG01019927	Aqp1ab2	Tongue sole	*Cynoglossus semilaevis*	Acanthopterygii	Pleuronectiformes	Cynoglossidae
EF011740	Aqp1ab	Gilthead seabream	*Sparus aurata*	Acanthopterygii	Perciformes	Sparidae
AGTA02006474	Aqp1ab1	Zebra mbuna	*Maylandia zebra*	Acanthopterygii	Perciformes	Cichlidae
AFNX01030543	Aqp1ab1	Red mwanza	*Pundamilia nyererei*	Acanthopterygii	Perciformes	Cichlidae
AFNY01028577	Aqp1ab1	Lyretail cichlid	*Neolamprologus brichardi*	Acanthopterygii	Perciformes	Cichlidae
AFNZ01000497	Aqp1ab1	Burton's mouthbrooder	*Haplochromis burtoni*	Acanthopterygii	Perciformes	Cichlidae
ABPJ01031686	Aqp1ab1	Mchenga	*Mchenga conophoros*	Acanthopterygii	Perciformes	Cichlidae
XP_003438132/AERX01000980	Aqp1ab1	Nile tilapia	*Oreochromis niloticus*	Acanthopterygii	Perciformes	Cichlidae
AGTA02006474	Aqp1ab2	Zebra mbuna	*Maylandia zebra*	Acanthopterygii	Perciformes	Cichlidae
AFNX01030543	Aqp1ab2	Red mwanza	*Pundamilia nyererei*	Acanthopterygii	Perciformes	Cichlidae
AFNY01028577	Aqp1ab2	Lyretail cichlid	*Neolamprologus brichardi*	Acanthopterygii	Perciformes	Cichlidae
AFNZ01000497	Aqp1ab2	Burton's mouthbrooder	*Haplochromis burtoni*	Acanthopterygii	Perciformes	Cichlidae
AERX01000980	Aqp1ab2	Nile tilapia	*Oreochromis niloticus*	Acanthopterygii	Perciformes	Cichlidae
EG630605	Aqp1ab	Northern bluefin tuna	*Thunnus thynnus*	Acanthopterygii	Perciformes	Scombridae
BADN01032052	Aqp1ab1	Pacific bluefin tuna	*Thunnus orientalis*	Acanthopterygii	Perciformes	Scombridae
BADN01032052	Aqp1ab2p	Pacific bluefin tuna	*Thunnus orientalis*	Acanthopterygii	Perciformes	Scombridae
GAJJ01004370/AWGY01043971	Aqp1ab	Sablefish	*Anoplopoma fimbria*	Acanthopterygii	Scorpaeniformes	Anoplopomatidae
AUPQ01075151	Aqp1ab1	Flag rockfish	*Sebastes rubrivinctus*	Acanthopterygii	Scorpaeniformes	Sebastidae
AUPQ01075151	Aqp1ab2p	Flag rockfish	*Sebastes rubrivinctus*	Acanthopterygii	Scorpaeniformes	Sebastidae
AUPR01144650	Aqp1ab1	Tiger rockfish	*Sebastes nigrocinctus*	Acanthopterygii	Scorpaeniformes	Sebastidae
AUPR01144650	Aqp1ab2p	Tiger rockfish	*Sebastes nigrocinctus*	Acanthopterygii	Scorpaeniformes	Sebastidae
ENSGACP00000022968	Aqp1ab	Three-spined stickleback	*Gasterosteus aculeatus*	Acanthopterygii	Gasterosteiformes	Gasterosteidae
EV383061	Aqp1ab	Sockeye salmon	*Oncorhynchus nerka*	Protacanthopterygii	Salmoniformes	Salmonidae
AGKD01338342	Aqp1ab1	Atlantic salmon	*Salmo salar*	Protacanthopterygii	Salmoniformes	Salmonidae
CCAF010038802	Aqp1ab2	Rainbow trout	*Oncorhynchus mykiss*	Protacanthopterygii	Salmoniformes	Salmonidae
ACI33306/NP_001133472/AGKD01009244	Aqp1ab2	Atlantic salmon	*Salmo salar*	Protacanthopterygii	Salmoniformes	Salmonidae
DT351889	Aqp1ab	Fathead minnow	*Pimephales promelas*	Ostariophysi	Cypriniformes	Cyprinidae
EU327345/ENSDARP00000110131	Aqp1ab	Zebrafish	*Danio rerio*	Ostariophysi	Cypriniformes	Cyprinidae
APWO01101097	Aqp1ab	Mexican tetra	*Astyanax mexicanus*	Ostariophysi	Characiformes	Characidae
GAGX01121276/GAGX01205052/GAGX01141981	Aqp1ab	Brown bullhead	*Ameiurus nebulosus*	Ostariophysi	Siluriformes	Ictaluridae
CK418363	Aqp1ab	Channel catfish	*Ictalurus punctatus*	Ostariophysi	Siluriformes	Ictaluridae
HM051492	Aqp1ab	Stinging catfish	*Heteropneustes fossilis*	Ostariophysi	Siluriformes	Heteropneustidae
ABM26906/CAD92028	Aqp1ab	European eel	*Anguilla anguilla*	Elopomorpha	Anguilliformes	Anguillidae
BAK53383 /AVPY01099885/AVPY01099884	Aqp1ab	Japanese eel	*Anguilla japonica*	Elopomorpha	Anguilliformes	Anguillidae
AHAT01010321	Aqp1	Spotted gar	*Lepisosteus oculatus*	Holostei	Semionotiformes	Lepisosteidae
KJ784516	Aqp1	Smaller spotted catshark	*Scyliorhinus canicula*	Chondrichthyes	Carcharhiniformes	Scyliorhinidae
AESE011188333/AESE012729986/ AESE011094485/AESE010570963	Aqp1	Little skate	*Leucoraja erinacea*	Chondrichthyes	Rajiformes	Rajidae
ES324936	Aqp1	Spiny dogfish	*Squalus acanthias*	Chondrichthyes	Squaliformes	Squalidae
AAVX02036742/ AAVX02055351	Aqp1	Ghost shark	*Callorhinchus milii*	Chondrichthyes	Chimaeriformes	Callorhinchidae
**AQP2**						
ENSP00000199280	AQP2	Human	*Homo sapiens*	Euarchontoglires	Primates	Hominidae
ENSPTRP00000008402	AQP2	Chimpanzee	*Pan troglodytes*	Euarchontoglires	Primates	Hominidae
ENSGGOP00000005773	AQP2	Western lowland gorilla	*Gorilla gorilla gorilla*	Euarchontoglires	Primates	Hominidae
ENSPPYP00000005138	AQP2	Sumatran orangutan	*Pongo abelii*	Euarchontoglires	Primates	Hominidae
ENSNLEP00000021639	AQP2	Northern white-cheeked gibbon	*Nomascus leucogenys*	Euarchontoglires	Primates	Hylobatidae
ENSMMUP00000030182	AQP2	Rhesus macaque	*Macaca mulatta*	Euarchontoglires	Primates	Cercopithecidae
EHH66266	AQP2	Crab-eating macaque	*Macaca fascicularis*	Euarchontoglires	Primates	Cercopithecidae
ENSP00000199280	AQP2	Hamadryas baboon	*Papio hamadryas*	Euarchontoglires	Primates	Cercopithecidae
ALAR01214979/ALAR01214978	AQP2	Chinese tree shrew	*Tupaia chinensis*	Euarchontoglires	Scandentia	Tupaiidae
ENSCJAP00000039175	AQP2	White-tufted-ear marmoset	*Callithrix jacchus*	Euarchontoglires	Primates	Cebidae
XP_003939194	AQP2	Bolivian squirrel monkey	*Saimiri boliviensis boliviensis*	Euarchontoglires	Primates	Cebidae
ENSMICP00000005498	AQP2	Gray mouse lemur	*Microcebus murinus*	Euarchontoglires	Primates	Cheirogaleidae
XP_003793670/ENSOGAP00000004157	AQP2	Small-eared galago/Bushbaby	*Otolemur garnettii*	Euarchontoglires	Primates	Galagidae
ENSTBEP00000010708	AQP2	Northern tree shrew	*Tupaia belangeri*	Euarchontoglires	Scandentia	Tupaiidae
ENSMUSP00000023752	AQP2	Mouse	*Mus musculus*	Euarchontoglires	Rodentia	Muridae
ENSRNOP00000000324	AQP2	Norway rat	*Rattus norvegicus*	Euarchontoglires	Rodentia	Muridae
AAN11309	AQP2	Spinifex hopping mouse	*Notomys alexis*	Euarchontoglires	Rodentia	Muridae
XP_003507883	AQP2	Chinese hamster	*Cricetulus griseus*	Euarchontoglires	Rodentia	Cricetidae
XP_005353969	AQP2	Prairie vole	*Microtus ochrogaster*	Euarchontoglires	Rodentia	Cricetidae
AKZC01010080/AKZC01010081	AQP2	Lesser Egyptian jerboa	*Jaculus jaculus*	Euarchontoglires	Rodentia	Dipodidae
ENSDORP00000013587	AQP2	Ord's kangaroo rat	*Dipodomys ordii*	Euarchontoglires	Rodentia	Heteromyidae
XP_004637853	AQP2	Degu	*Octodon degus*	Euarchontoglires	Rodentia	Octodontidae
ENSCPOP00000019628	AQP2	Domestic guinea pig	*Cavia porcellus*	Euarchontoglires	Rodentia	Caviidae
EHB00520	AQP2	Naked mole-rat	*Heterocephalus glaber*	Euarchontoglires	Rodentia	Bathyergidae
ENSSTOP00000010097	AQP2	Thirteen-lined ground squirrel	*Ictidomys tridecemlineatus*	Euarchontoglires	Rodentia	Sciuridae
XM_005411856	AQP2	Long-tailed chinchilla	*Chinchilla lanigera*	Euarchontoglires	Rodentia	Chinchillidae
ENSOPRP00000007457	AQP2	American pika	*Ochotona princeps*	Euarchontoglires	Lagomorpha	Ochotonidae
ENSOCUP00000004002	AQP2	Rabbit	*Oryctolagus cuniculus*	Euarchontoglires	Lagomorpha	Leporidae
ENSEEUP00000002145	AQP2	Western European hedgehog	*Erinaceus europaeus*	Laurasiatheria	Insectivora	Erinaceinae
ENSSART00000007564	AQP2	European shrew	*Sorex araneus*	Laurasiatheria	Insectivora	Soricidae
AJFV01069458	AQP2	Star-nosed mole	*Condylura cristata*	Laurasiatheria	Insectivora	Talpidae
ENSPVAP00000006320	AQP2	Large flying fox/Megabat	*Pteropus vampyrus*	Laurasiatheria	Chiroptera	Pteropodidae
ELK08377	AQP2	Black flying fox	*Pteropus alecto*	Laurasiatheria	Chiroptera	Pteropodidae

XM_006091581	AQP2	Little brown bat/Microbat	*Myotis lucifugus*	Laurasiatheria	Chiroptera	Vespertilionidae
XP_005877371	AQP2	Brandt's bat	*Myotis brandtii*	Laurasiatheria	Chiroptera	Vespertilionidae
O62735/ XP_004006420	AQP2	Sheep	*Ovis aries*	Laurasiatheria	Ruminantia	Bovidae
AJPT01054268/AJPT01054267	AQP2	Goat	*Capra hircus*	Laurasiatheria	Ruminantia	Bovidae
XP_005981023	AQP2	Chiru	*Pantholops hodgsonii*	Laurasiatheria	Ruminantia	Bovidae
ENSBTAP00000011024	AQP2	Cow	*Bos taurus*	Laurasiatheria	Ruminantia	Bovidae
ELR55938	AQP2	Yak	*Bos mutus*	Laurasiatheria	Ruminantia	Bovidae
ENSTTRP00000000945	AQP2	Bottlenosed dolphin	*Tursiops truncatus*	Laurasiatheria	Cetacea	Delphinidae
AGV55471	AQP2	Indo-pacific humpbacked dolphin	*Sousa chinensis*	Laurasiatheria	Cetacea	Delphinidae
AUPI01045706/AGV55468	AQP2	Yangtze River dolphin	*Lipotes vexillifer*	Laurasiatheria	Cetacea	Delphinidae
ANOL02036890	AQP2	Killer whale	*Orcinus orca*	Laurasiatheria	Cetacea	Delphinidae
AGV55469	AQP2	Indo-Pacific finless porpoise	*Neophocaena phocaenoides*	Laurasiatheria	Cetacea	Phocoenidae
AWZP01022054/AWZP01022053/AJ251101	AQP2	Sperm whale	*Physeter catodon*	Laurasiatheria	Cetacea	Physeteridae
AGV55472	AQP2	Beluga whale	*Delphinapterus leucas*	Laurasiatheria	Cetacea	Monodontidae
AGV55467	AQP2	Blainville's beaked whale	*Mesoplodon densirostris*	Laurasiatheria	Cetacea	Ziphiidae
ATDI01166360/AGV55470	AQP2	Minke whale	*Balaenoptera acutorostrata*	Laurasiatheria	Cetacea	Balaenopteridae
AGV55466	AQP2	Omura's baleen whale	*Balaenoptera omurai*	Laurasiatheria	Cetacea	Balaenopteridae
CAC10187	AQP2	Fin whale	*Balaenoptera physalus*	Laurasiatheria	Cetacea	Balaenopteridae
AJ251102	AQP2	Caribbean manatee	*Trichechus manatus*	Laurasiatheria	Sirenia	Trichechidae
ENSSSCP00000000222	AQP2	Pig	*Sus scrofa*	Laurasiatheria	Suina	Suidae
ENSVPAP00000006539	AQP2	Alpaca	*Vicugna pacos*	Laurasiatheria	Tylopoda	Camelidae
AGVR01013768	AQP2	Wild Bactrian camel	*Camelus ferus*	Laurasiatheria	Tylopoda	Camelidae
GADU01035806	AQP2	Arabian camel	*Camelus dromedarius*	Laurasiatheria	Tylopoda	Camelidae
ENSECAP00000021228	AQP2	Horse	*Equus caballus*	Laurasiatheria	Perissodactyla	Equidae
AKZM01022930	AQP2	Southern white rhinocerus	*Ceratotherium simum simum*	Laurasiatheria	Perissodactyla	Rhinocerotidae
ENSAMEP00000001129	AQP2	Giant panda	*Ailuropoda melanoleuca*	Laurasiatheria	Carnivora	Ursidae
ENSCAFP00000012304	AQP2	Dog	*Canis lupus familiaris*	Laurasiatheria	Carnivora	Canidae
ENSMPUP00000014416	AQP2	Domestic ferret	*Mustela putorius furo*	Laurasiatheria	Carnivora	Mustelidae
ENSFCAP00000008771	AQP2	Domestic cat	*Felis catus*	Laurasiatheria	Carnivora	Felidae
ATCQ01001277	AQP2	Amur tiger	*Panthera tigris altaica*	Laurasiatheria	Carnivora	Felidae
APMU01027972	AQP2	Weddell seal	*Leptonychotes weddellii*	Laurasiatheria	Carnivora	Phocidae
ANOP01016854	AQP2	Pacific walrus	*Odobenus rosmarus divergens*	Laurasiatheria	Carnivora	Odobenidae
CAA71660	AQP2	Pangolin	*Manis sp.*	Laurasiatheria	Pholidota	Manidae
ENSLAFP00000017930	AQP2	African savanna elephant	*Loxodonta africana*	Afrotheria	Proboscidea	Elephantidae
ENSPCAP00000006378	AQP2	Cape rock hyrax	*Procavia capensis*	Afrotheria	Hyracoidea	Procaviidae
AHIN01131586	AQP2	Florida manatee	*Trichechus manatus latirostris*	Afrotheria	Sirenia	Trichechidae
AJ251102	AQP2	West Indian manatee	*Trichechus manatus*	Afrotheria	Sirenia	Trichechidae
CAA75902	AQP2	Dugong	*Dugong dugon*	Afrotheria	Sirenia	Dugongidae
ENSPCAP00000006378	AQP2	Cape rock hyrax	*Procavia capensis*	Afrotheria	Hyracoidea	Procaviidae
CAC16373	AQP2	Western tree hyrax	*Dendrohyrax dorsalis*	Afrotheria	Hyracoidea	Procaviidae
CAC16395	AQP2	Nimba otter shrew	*Micropotamogale lamottei*	Afrotheria	Afrosoricida	Tenrecidae
ALYB01072797	AQP2	Ardvaark	*Orycteropus afer afer*	Afrotheria	Tubulidentata	Orycteropodidae
DY596766	AQP2	Common brushtail	*Trichosurus vulpecula*	Metatheria	Diprotodontia	Phalangeridae
ENSMEUP00000004277	AQP2	Tammar wallaby	*Macropus eugenii*	Metatheria	Diprotodontia	Macropodidae
ENSSHAP00000003457	AQP2	Tasmanian devil	*Sarcophilus harrisii*	Metatheria	Dasyuromorphia	Dasyuridae
ENSOANP00000000683	AQP2	Platypus	*Ornithorhynchus anatinus*	Prototheria	Monotremata	Ornithorhynchidae
AGAI01046028	AQP2	Budgerigar	*Melopsittacus undulatus*	Aves	Psittaciformes	Psittacidae
AOCU01029765	AQP2	Puerto Rican parrot	*Amazona vittata*	Aves	Psittaciformes	Psittacidae
AMXX01179152/AOUJ01046482/AOUJ01046483	AQP2	Scarlet macaw	*Ara macao*	Aves	Psittaciformes	Psittacidae
CAVT010044030	AQP2	Common canary	*Serinus canaria*	Aves	Passeriformes	Fringillidae
AKZB01039358	AQP2	Medium ground finch	*Geospiza fortis*	Aves	Passeriformes	Fringillidae
ENSTGUP00000004286	AQP2	Zebra finch	*Taeniopygia guttata*	Aves	Passeriformes	Estrildidae
ANZD01003998	AQP2	Tibetan ground-tit	*Pseudopodoces humilis*	Aves	Passeriformes	Paridae
AGTO01022026/	AQP2	Collared flycatcher	*Ficedula albicollis*	Aves	Passeriformes	Muscicapidae
AKMT01046110/AKMT01046109	AQP2	Peregrin falcon	*Falco peregrinus*	Aves	Falconiformes	Falconidae
AKMU01023145	AQP2	Saker falcon	*Falco cherrug*	Aves	Falconiformes	Falconidae
AKCR01079490	AQP2	Rock pigeon	*Columba livia*	Aves	Columbiformes	Columbidae
ENSAPLP00000015369	AQP2	Mallard	*Anas platyrhynchos*	Aves	Anseriformes	Anatidae
AAR06953	AQP2	Common quail	*Coturnix coturnix*	Aves	Galliformes	Phasianidae
ENSMGAP00000011651	AQP2	Turkey	*Meleagris gallopavo*	Aves	Galliformes	Phasianidae
ENSGALP00000016674	AQP2	Chicken	*Gallus gallus*	Aves	Galliformes	Phasianidae
AKHW01094568	AQP2	American alligator	*Alligator mississippiensis*	Archosauria	Crocodylia	Alligatoridae
AVPB01022407	AQP2	Chinese alligator	*Alligator sinensis*	Archosauria	Crocodylia	Alligatoridae
AGCU01084247/ENSPSIP00000006745	AQP2	Chinese softshell turtle	*Pelodiscus sinensis*	Sauropsida	Testudines	Trionychidae
AJIM01256814/AJIM01256813	AQP2	Green seaturtle	*Chelonia mydas*	Sauropsida	Testudines	Cheloniidae
AHGY01100855/AHGY01100857	AQP2	Western painted turtle	*Chrysemys picta bellii*	Sauropsida	Testudines	Emydidae
AZIM01000294/ETE71913	AQP2	King cobra	*Ophiophagus hannah*	Lepidosauria	Squamata	Elapidae
AEQU02005686/AEQU02005685	AQP2	Burmese python	*Python molurus bivittatus*	Lepidosauria	Squamata	Pythonidae
ENSACAP00000008275	AQP2	Green anole	*Anolis carolinensis*	Lepidosauria	Squamata	Iguanidae
ABC98209	AQP2	Southern gray treefrog	*Hyla chrysoscelis*	Amphibia	Anura	Hylidae
BAF80993	AQP2	Japanese treefrog	*Hyla japonica*	Amphibia	Anura	Hylidae
GAEI01001789	AQP2	Pacific treefrog	*Pseudacris regilla*	Amphibia	Anura	Hylidae
GAEG01002994	AQP2	Green frog	*Rana clamitans*	Amphibia	Anura	Ranidae

AB474277	Aqp2	West African lungfish	*Protopterus annectens*	Dipnoi	Lepidosireniformes	Protopteridae
BAH98063	Aqp2	Australian lungfish	*Neoceratodus forsteri*	Dipnoi	Ceratodontiformes	Ceratodontidae
AFYH01001362/ENSLACP00000020482	Aqp2a	Coelacanth	*Latimeria chalumnae*	Actinistia	Coelacanthiformes	Coelacanthidae
AFYH01001362/GENSCAN00000096392	Aqp2b	Coelacanth	*Latimeria chalumnae*	Actinistia	Coelacanthiformes	Coelacanthidae
AFYH01001365/AFYH01001367/ ENSLACP00000020505	Aqp2c	Coelacanth	*Latimeria chalumnae*	Actinistia	Coelacanthiformes	Coelacanthidae
**AQP3**						
ENSP00000297991	AQP3	Human	*Homo sapiens*	Euarchontoglires	Primates	Hominidae
ENSPTRP00000035672	AQP3	Chimpanzee	*Pan troglodytes*	Euarchontoglires	Primates	Hominidae
ENSGGOP00000003917	AQP3	Western lowland gorilla	*Gorilla gorilla gorilla*	Euarchontoglires	Primates	Hominidae
ENSPPYP00000021438	AQP3	Sumatran orangutan	*Pongo abelii*	Euarchontoglires	Primates	Hominidae
ENSNLEP00000006154	AQP3	Northern white-cheeked gibbon	*Nomascus leucogenys*	Euarchontoglires	Primates	Hylobatidae
ENSMMUP00000004418	AQP3	Rhesus macaque	*Macaca mulatta*	Euarchontoglires	Primates	Cercopithecidae
ENSP00000297991	AQP3	Hamadryas baboon	*Papio hamadryas*	Euarchontoglires	Primates	Cercopithecidae
AHZZ01031032	AQP3	Olive baboon	*Papio anubis*	Euarchontoglires	Primates	Cercopithecidae
ENSCJAP00000015149	AQP3	White-tufted-ear marmoset	*Callithrix jacchus*	Euarchontoglires	Primates	Cebidae
XP_003939792	AQP3	Bolivian squirrel monkey	*Saimiri boliviensis boliviensis*	Euarchontoglires	Primates	Cebidae
ENSTSYP00000010301	AQP3	Philippine tarsier	*Tarsius syrichta*	Euarchontoglires	Primates	Tarsiidae
ENSMICP00000011053	AQP3	Gray mouse lemur	*Microcebus murinus*	Euarchontoglires	Primates	Cheirogaleidae
XP_003800331/ENSOGAP00000012860	AQP3	Small-eared galago/Bushbaby	*Otolemur garnettii*	Euarchontoglires	Primates	Galagidae
ENSTBEP00000002696	AQP3	Northern tree shrew	*Tupaia belangeri*	Euarchontoglires	Scandentia	Tupaiidae
ELW70260	AQP3	Chinese tree shrew	*Tupaia chinensis*	Euarchontoglires	Scandentia	Tupaiidae
ENSMUST00000055327	AQP3	Mouse	*Mus musculus*	Euarchontoglires	Rodentia	Muridae
ENSRNOP00000013803	AQP3	Norway rat	*Rattus norvegicus*	Euarchontoglires	Rodentia	Muridae
XP_003512012	AQP3	Chinese hamster	*Cricetulus griseus*	Euarchontoglires	Rodentia	Cricetidae
ENSDORP00000005634	AQP3	Ord's kangaroo rat	*Dipodomys ordii*	Euarchontoglires	Rodentia	Heteromyidae
ENSCPOP00000011041	AQP3	Domestic guinea pig	*Cavia porcellus*	Euarchontoglires	Rodentia	Caviidae
EHB02657	AQP3	Naked mole-rat	*Heterocephalus glaber*	Euarchontoglires	Rodentia	Bathyergidae
ENSSTOP00000014181	AQP3	Thirteen-lined ground squirrel	*Ictidomys tridecemlineatus*	Euarchontoglires	Rodentia	Sciuridae
ENSOPRP00000015348	AQP3	American pika	*Ochotona princeps*	Euarchontoglires	Lagomorpha	Ochotonidae
ENSOCUP00000015333	AQP3	Rabbit	*Oryctolagus cuniculus*	Euarchontoglires	Lagomorpha	Leporidae
ENSEEUP00000000364	AQP3	Western European hedgehog	*Erinaceus europaeus*	Laurasiatheria	Insectivora	Erinaceinae
ENSSARP00000001379	AQP3	European shrew	*Sorex araneus*	Laurasiatheria	Insectivora	Soricidae
BAF49644	AQP3	House shrew	*Suncus murinus*	Laurasiatheria	Insectivora	Soricidae
ENSPVAP00000010827	AQP3	Large flying fox/Megabat	*Pteropus vampyrus*	Laurasiatheria	Chiroptera	Pteropodidae
ELK09077	AQP3	Black flying fox	*Pteropus alecto*	Laurasiatheria	Chiroptera	Pteropodidae
ENSMLUP00000011127	AQP3	Little brown bat/Microbat	*Myotis lucifugus*	Laurasiatheria	Chiroptera	Vespertilionidae
ELK34287	AQP3	David´s myotis	*Myotis davidii*	Laurasiatheria	Chiroptera	Vespertilionidae
XP_004005296/ENSP00000297991/ ACIV010833122	AQP3	Sheep	*Ovis aries*	Laurasiatheria	Ruminantia	Bovidae
ENSBTAP00000011196	AQP3	Cow	*Bos taurus*	Laurasiatheria	Ruminantia	Bovidae
ELR51091	AQP3	Yak	*Bos mutus*	Laurasiatheria	Ruminantia	Bovidae
ENSTTRP00000010032	AQP3	Bottlenosed dolphin	*Tursiops truncatus*	Laurasiatheria	Cetacea	Delphinidae
ANOL02039846/XP_004275133	AQP3	Killer whale	*Orcinus orca*	Laurasiatheria	Cetacea	Delphinidae
ENSSSCP00000011724	AQP3	Pig	*Sus scrofa*	Laurasiatheria	Suina	Suidae
ENSVPAP00000005209	AQP3	Alpaca	*Vicugna pacos*	Laurasiatheria	Tylopoda	Camelidae
ENSECAP00000018712	AQP3	Horse	*Equus caballus*	Laurasiatheria	Perissodactyla	Equidae
ENSAMEP00000007833	AQP3	Giant panda	*Ailuropoda melanoleuca*	Laurasiatheria	Carnivora	Ursidae
ENSCAFP00000002716	AQP3	Dog	*Canis lupus familiaris*	Laurasiatheria	Carnivora	Canidae
ENSCAFP00000002716	AQP3	Domestic ferret	*Mustela putorius furo*	Laurasiatheria	Carnivora	Mustelidae
ENSFCAP00000002505	AQP3	Domestic cat	*Felis catus*	Laurasiatheria	Carnivora	Felidae
ENSPCAP00000015510	AQP3	Cape rock hyrax	*Procavia capensis*	Afrotheria	Hyracoidea	Procaviidae
ENSLAFP00000018135	AQP3	African savanna elephant	*Loxodonta africana*	Afrotheria	Proboscidea	Elephantidae
ENSETEP00000014787	AQP3	Lesser hedgehog tenrec	*Echinops telfairi*	Afrotheria	Afrosoricida	Tenrecidae
AHIN01023994	AQP3	Florida manatee	*Trichechus manatus latirostris*	Afrotheria	Sirenia	Trichechidae
ALYB01234330	AQP3	Ardvaark	*Orycteropus afer afer*	Afrotheria	Tubulidentata	Orycteropodidae
ENSDNOP00000017578	AQP3	Nine-banded armadillo	*Dasypus novemcinctus*	Xenarthra	Cingulata	Dasypodidae
ABVD01594697	AQP3	Hoffmann's two-fingered sloth	*Choloepus hoffmanni*	Xenarthra	Pilosa	Megalonychidae
ENSMEUP00000013288	AQP3	Tammar wallaby	*Macropus eugenii*	Metatheria	Diprotodontia	Macropodidae
ENSSHAP00000003616	AQP3	Tasmanian devil	*Sarcophilus harrisii*	Metatheria	Dasyuromorphia	Dasyuridae
ENSMODP00000004690	AQP3	Gray short-tailed opossum	*Monodelphis domestica*	Metatheria	Didelphimorphia	Didelphidae
ENSOANP00000023098	AQP3	Platypus	*Ornithorhynchus anatinus*	Prototheria	Monotremata	Ornithorhynchidae
AGAI01048038/AGAI01048039	AQP3	Budgerigar	*Melopsittacus undulatus*	Aves	Psittaciformes	Psittacidae
AMXX01081206/AMXX01126069/AOUJ01111547/ AOUJ01246249	AQP3	Scarlet macaw	*Ara macao*	Aves	Psittaciformes	Psittacidae
CAVT010029651/CAVT010029652	AQP3	Common canary	*Serinus canaria*	Aves	Passeriformes	Fringillidae
AKZB01095700/AKZB01095701	AQP3	Medium ground finch	*Geospiza fortis*	Aves	Passeriformes	Fringillidae
ENSTGUP00000001721	AQP3	Zebra finch	*Taeniopygia guttata*	Aves	Passeriformes	Estrildidae
AGTO01001363	AQP3	Collared flycatcher	*Ficedula albicollis*	Aves	Passeriformes	Muscicapidae
ENSAPLP00000004013	AQP3	Mallard	*Anas platyrhynchos*	Aves	Anseriformes	Anatidae
ACF19804	AQP3	Common quail	*Coturnix coturnix*	Aves	Galliformes	Phasianidae
ENSMGAP00000001305	AQP3	Turkey	*Meleagris gallopavo*	Aves	Galliformes	Phasianidae
ENSGALP00000003859	AQP3	Chicken	*Gallus gallus*	Aves	Galliformes	Phasianidae
AKHW01039336/AKHW01039339	AQP3	American alligator	*Alligator mississippiensis*	Archosauria	Crocodylia	Alligatoridae

AVPB01026904	AQP3	Chinese alligator	*Alligator sinensis*	Archosauria	Crocodylia	Alligatoridae
ENSPSIP00000017085	AQP3	Chinese softshell turtle	*Pelodiscus sinensis*	Sauropsida	Testudines	Trionychidae
EMP32512	AQP3	Green seaturtle	*Chelonia mydas*	Sauropsida	Testudines	Cheloniidae
GANJ01003270	AQP3	Reeves's turtle	*Mauremys reevesii*	Sauropsida	Testudines	Geoemydidae
AHGY01159137/AHGY01159138	AQP3	Western painted turtle	*Chrysemys picta bellii*	Sauropsida	Testudines	Emydidae
AEH96275	AQP3	Salt marsh snake	*Nerodia clarkii clarkii*	Lepidosauria	Squamata	Colubridae
AEH96276	AQP3	Banded water snake	*Nerodia fasciata*	Lepidosauria	Squamata	Colubridae
ETE57537/AZIM01031376/AZIM01008241	AQP3	King cobra	*Ophiophagus hannah*	Lepidosauria	Squamata	Elapidae
JAB54667	AQP3	Eastern coral snake	*Micrurus fulvius*	Lepidosauria	Squamata	Elapidae
AEH96274	AQP3	Broad-banded blue sea krait	*Laticauda semifasciata*	Lepidosauria	Squamata	Elapidae
AFJ49443	AQP3	Eastern diamondback rattlesnake	*Crotalus adamanteus*	Lepidosauria	Squamata	Viperidae
GAAZ01000200	AQP3	Timber rattlesnake	*Crotalus horridus*	Lepidosauria	Squamata	Viperidae
AEQU02198269/AEQU02198273/AEQU02198274	AQP3	Burmese python	*Python molurus bivittattus*	Lepidosauria	Squamata	Pythonidae
ENSACAP00000012483	AQP3	Green anole	*Anolis carolinensis*	Lepidosauria	Squamata	Iguanidae
ABC98210	AQP3	Southern gray treefrog	*Hyla chrysoscelis*	Amphibia	Anura	Hylidae
ACM18196	AQP3	Dark-spotted frog	*Pelophylax nigromaculatus*	Amphibia	Anura	Ranidae
ACM51136	AQP3	Asiatic toad	*Bufo gargarizans*	Amphibia	Anura	Bufonidae
NP_001081876	AQP3	African clawed frog	*Xenopus laevis*	Amphibia	Anura	Pipidae
AAI58299/ENSXETP00000004583	AQP3	Western clawed frog	*Xenopus (Silurana) tropicalis*	Amphibia	Anura	Pipidae
GAQK01000665	AQP3	Chinese salamander	*Hynobius chinensis*	Amphibia	Caudata	Hynobiidae
AFYH01154020/AFYH01154029/AFYH01154028/ AFYH01154027/AFYH01154026	Aqp3	Coelacanth	*Latimeria chalumnae*	Actinistia	Coelacanthiformes	Coelacanthidae
ENSTRUP00000012922	Aqp3a	Torafugu	*Takifugu rubripes*	Acanthopterygii	Tetraodontiformes	Tetraodontidae
AOOT01001915/AOOT01001916/AOOT01001917	Aqp3a	Sansaifugu	*Takifugu flavidus*	Acanthopterygii	Tetraodontiformes	Tetraodontidae
ADG86338	Aqp3a	Mefugu	*Takifugu obscurus*	Acanthopterygii	Tetraodontiformes	Tetraodontidae
ENSTNIP00000006428	Aqp3a	Green-spotted pufferfish	*Tetraodon nigroviridis*	Acanthopterygii	Tetraodontiformes	Tetraodontidae
GAAQ01004266	Aqp3a	Dover sole	*Solea solea*	Acanthopterygii	Pleuronectiformes	Soleidae
AGRG01019534/AGRG01019534	Aqp3a	Tongue sole	*Cynoglossus semilaevis*	Acanthopterygii	Pleuronectiformes	Cynoglossidae
DQ333306	Aqp3a	Gold-lined seabream	*Rhabdosargus sarba*	Acanthopterygii	Perciformes	Sparidae
AGTA02008491/AGTA02008490	Aqp3aa	Zebra mbuna	*Maylandia zebra*	Acanthopterygii	Perciformes	Cichlidae
AFNX01003871/AFNX01003872	Aqp3aa	Red mwanza	*Pundamilia nyererei*	Acanthopterygii	Perciformes	Cichlidae
AFNY01011364	Aqp3aa	Lyretail cichlid	*Neolamprologus brichardi*	Acanthopterygii	Perciformes	Cichlidae
AFNZ01010497/AFNZ01010496	Aqp3aa	Burton's mouthbrooder	*Haplochromis burtoni*	Acanthopterygii	Perciformes	Cichlidae
ENSONIP00000020098	Aqp3aa	Nile tilapia	*Oreochromis niloticus*	Acanthopterygii	Perciformes	Cichlidae
AGTA02008478/AGTA02008480	Aqp3ab	Zebra mbuna	*Maylandia zebra*	Acanthopterygii	Perciformes	Cichlidae
AFNX01003878	Aqp3ab	Red mwanza	*Pundamilia nyererei*	Acanthopterygii	Perciformes	Cichlidae
AFNY01011353/AFNY01011355	Aqp3ab	Lyretail cichlid	*Neolamprologus brichardi*	Acanthopterygii	Perciformes	Cichlidae
DY630346/AFNZ01010486/AFNZ01010487	Aqp3ab	Burton's mouthbrooder	*Haplochromis burtoni*	Acanthopterygii	Perciformes	Cichlidae
ENSONIP00000020110	Aqp3ab	Nile tilapia	*Oreochromis niloticus*	Acanthopterygii	Perciformes	Cichlidae
BAD20708	Aqp3ab	Mozamique tilapia	*Oreochromis mossambicus*	Acanthopterygii	Perciformes	Cichlidae
BJ686705	Aqp3ab	Victoria thick-lips mouthbrooder	*Haplochromis chilotes*	Acanthopterygii	Perciformes	Cichlidae
BJ690402	Aqp3ab	Redtail sheller	*Haplochromis sp.*	Acanthopterygii	Perciformes	Cichlidae
BADN01029189	Aqp3a	Pacific bluefin tuna	*Thunnus orientalis*	Acanthopterygii	Perciformes	Scombridae
AWGY01142399	Aqp3aa	Sablefish	*Anoplopoma fimbria*	Acanthopterygii	Scorpaeniformes	Anoplopomatidae
AWGY01126917	Aqp3ab	Sablefish	*Anoplopoma fimbria*	Acanthopterygii	Scorpaeniformes	Anoplopomatidae
AUPQ01054323	Aqp3a	Flag rockfish	*Sebastes rubrivinctus*	Acanthopterygii	Scorpaeniformes	Sebastidae
AUPR01136281/AUPR01181854	Aqp3a	Tiger rockfish	*Sebastes nigrocinctus*	Acanthopterygii	Scorpaeniformes	Sebastidae
ENSGACP00000013694	Aqp3a	Three-spined stickleback	*Gasterosteus aculeatus*	Acanthopterygii	Gasterosteiformes	Gasterosteidae
ENSORLP00000012759	Aqp3a	Japanese medaka	*Oryzias latipes*	Acanthopterygii	Beloniformes	Adrianichthyidae
GAIB01128398/JZ265611	Aqp3a	Turquoise killifish	*Nothobranchius furzeri*	Acanthopterygii	Cyprinodontiformes	Nothobranchiidae
ACI49539	Aqp3a	Common mummichog	*Fundulus heteroclitus*	Acanthopterygii	Cyprinodontiformes	Fundulidae
ES375494	Aqp3a	Guppy	*Poecilia reticulata*	Acanthopterygii	Cyprinodontiformes	Poeciliidae
AYCK01000894	Aqp3a	Amazon molly	*Poecilia formosa*	Acanthopterygii	Cyprinodontiformes	Poeciliidae
ENSXMAP00000019454	Aqp3a	Southern platyfish	*Xiphophorus maculatus*	Acanthopterygii	Cyprinodontiformes	Poeciliidae
FF416557/ENSGMOP00000000666	Aqp3a	Atlantic cod	*Gadus morhua*	Paracanthopterygii	Gadiformes	Gadidae
CCAF010038670	Aqp3a1	Rainbow trout	*Oncorhynchus mykiss*	Protacanthopterygii	Salmoniformes	Salmonidae
AGKD01094322/AGKD01056897	Aqp3a1	Atlantic salmon	*Salmo salar*	Protacanthopterygii	Salmoniformes	Salmonidae
CCAF010040466	Aqp3a2	Rainbow trout	*Oncorhynchus mykiss*	Protacanthopterygii	Salmoniformes	Salmonidae
AGKD01068849/AGKD01149541	Aqp3a2	Atlantic salmon	*Salmo salar*	Protacanthopterygii	Salmoniformes	Salmonidae
EX885773	Aqp3a	Common carp	*Cyprinus carpio*	Ostariophysi	Cypriniformes	Cyprinidae
AM928312	Aqp3a	Goldfish	*Carassius auratus*	Ostariophysi	Cypriniformes	Cyprinidae
BAB83082	Aqp3a	Big-scaled redfin	*Tribolodon hakonensis*	Ostariophysi	Cypriniformes	Cyprinidae
DT246747	Aqp3a	Fathead minnow	*Pimephales promelas*	Ostariophysi	Cypriniformes	Cyprinidae
GAHO01101838/GAHO01080011/GAHO01065997/ GAHO01045963/GAHO01051616/ GAHO01060873	Aqp3a	Small gill opening goldenline barbel	*Sinocyclocheilus angustiporus*	Ostariophysi	Cypriniformes	Cyprinidae
GAHL01086926/GAHL01011211	Aqp3a	Blind goldenline barbel	*Sinocyclocheilus anophthalmus*	Ostariophysi	Cypriniformes	Cyprinidae
EU341833/ENSDARP00000018463	Aqp3a	Zebrafish	*Danio rerio*	Ostariophysi	Cypriniformes	Cyprinidae
GAAD01002505	Aqp3a	Oriental weatherfish	*Misgurnus anguillicaudatus*	Ostariophysi	Cypriniformes	Cobitidae
APWO01112913	Aqp3a	Mexican tetra	*Astyanax mexicanus*	Ostariophysi	Characiformes	Characidae
CV987951	Aqp3a	Channel catfish	*Ictalurus punctatus*	Ostariophysi	Siluriformes	Ictaluridae
GAGX01002847	Aqp3a	Brown bullhead	*Ameiurus nebulosus*	Ostariophysi	Siluriformes	Ictaluridae
AVPY01014543/AVPY01014545/AVPY01014546	Aqp3a	Japanese eel	*Anguilla japonica*	Elopomorpha	Anguilliformes	Anguillidae
BADN01004527/BADN01004528	Aqp3b	Pacific bluefin tuna	*Thunnus orientalis*	Acanthopterygii	Perciformes	Scombridae
AWGY01142399	Aqp3b	Sablefish	*Anoplopoma fimbria*	Acanthopterygii	Scorpaeniformes	Anoplopomatidae

AUPQ01059403/AUPQ01059404/AUPQ01102314/ AUPQ01104729	Aqp3b	Flag rockfish	*Sebastes rubrivinctus*	Acanthopterygii	Scorpaeniformes	Sebastidae
AUPR01165934/AUPR01010211/AUPR01176565/ AUPR01194884	Aqp3b	Tiger rockfish	*Sebastes nigrocinctus*	Acanthopterygii	Scorpaeniformes	Sebastidae
FF411765/CAEA01538197/ENSGMOP00000008635	Aqp3b	Atlantic cod	*Gadus morhua*	Paracanthopterygii	Gadiformes	Gadidae
CAEA01538157/ ENSGMOP00000017976	Aqp3b	Atlantic cod	*Gadus morhua*	Paracanthopterygii	Gadiformes	Gadidae
CU071888/CCAF010016865	Aqp3b1	Rainbow trout	*Oncorhynchus mykiss*	Protacanthopterygii	Salmoniformes	Salmonidae
AGKD01002624	Aqp3b1	Atlantic salmon	*Salmo salar*	Protacanthopterygii	Salmoniformes	Salmonidae
CCAF010164985	Aqp3b2	Rainbow trout	*Oncorhynchus mykiss*	Protacanthopterygii	Salmoniformes	Salmonidae
AGKD01215587	Aqp3b2	Atlantic salmon	*Salmo salar*	Protacanthopterygii	Salmoniformes	Salmonidae
DT136147	Aqp3b	Fathead minnow	*Pimephales promelas*	Ostariophysi	Cypriniformes	Cyprinidae
GAHO01051838/GAHO01061212	Aqp3b	Small gill opening goldenline barbel	*Sinocyclocheilus angustiporus*	Ostariophysi	Cypriniformes	Cyprinidae
GAHL01027245/GAHL01037208	Aqp3b	Blind goldenline barbel	*Sinocyclocheilus anophthalmus*	Ostariophysi	Cypriniformes	Cyprinidae
EU341832/ENSDARP00000092015	Aqp3b	Zebrafish	*Danio rerio*	Ostariophysi	Cypriniformes	Cyprinidae
APWO01038715	Aqp3b	Mexican tetra	*Astyanax mexicanus*	Ostariophysi	Characiformes	Characidae
CAC85286	Aqp3b	European eel	*Anguilla anguilla*	Elopomorpha	Anguilliformes	Anguillidae
BAH89253/ GAGT01000112/AVPY01155731	Aqp3b	Japanese eel	*Anguilla japonica*	Elopomorpha	Anguilliformes	Anguillidae
AHAT01012409/ENSLOCP00000006040	Aqp3	Spotted gar	*Lepisosteus oculatus*	Holostei	Semionotiformes	Lepisosteidae
AESE012529808/AESE010180002/ AESE010640639/AESE010645223/AESE010899949	Aqp3	Little skate	*Leucoraja erinacea*	Chondrichthyes	Rajiformes	Rajidae
AESE010521158/AESE010679026/AESE010221296 /AESE011697121	Aqp3L	Little skate	*Leucoraja erinacea*	Chondrichthyes	Rajiformes	Rajidae
AAVX02016279	Aqp3	Ghost shark	*Callorhinchus milii*	Chondrichthyes	Chimaeriformes	Callorhinchidae
AAVX02016279	Aqp3L	Ghost shark	*Callorhinchus milii*	Chondrichthyes	Chimaeriformes	Callorhinchidae
KJ784518/AEFG01033607/ ENSPMAP00000008130	Aqp3L1	Sea lamprey	*Petromyzon marinus*	Hyperoartia	Petromyzontiformes	Petromyzontidae
APJL01047447/APJL01047446/APJL01047445	Aqp3L1	Arctic lamprey	*Lethenteron camtschaticum*	Hyperoartia	Petromyzontiformes	Petromyzontidae
KJ784520/AEFG01042179	Aqp3L2	Sea lamprey	*Petromyzon marinus*	Hyperoartia	Petromyzontiformes	Petromyzontidae
APJL01034901/APJL01034902	Aqp3L2	Arctic lamprey	*Lethenteron camtschaticum*	Hyperoartia	Petromyzontiformes	Petromyzontidae
**AQP4**						
ENSP00000372654	AQP4	Human	*Homo sapiens*	Euarchontoglires	Primates	Hominidae
ENSPTRP00000016898	AQP4	Chimpanzee	*Pan troglodytes*	Euarchontoglires	Primates	Hominidae
ENSGGOP00000010219	AQP4	Western lowland gorilla	*Gorilla gorilla gorilla*	Euarchontoglires	Primates	Hominidae
ENSPPYP00000010193	AQP4	Sumatran orangutan	*Pongo abelii*	Euarchontoglires	Primates	Hominidae
ENSNLEP00000007954	AQP4	Northern white-cheeked gibbon	*Nomascus leucogenys*	Euarchontoglires	Primates	Hylobatidae
ENSMMUP00000019590	AQP4	Rhesus macaque	*Macaca mulatta*	Euarchontoglires	Primates	Cercopithecidae
XP_005587045	AQP4	Crab-eating macaque	*Macaca fascicularis*	Euarchontoglires	Primates	Cercopithecidae
ENSP00000372654	AQP4	Hamadryas baboon	*Papio hamadryas*	Euarchontoglires	Primates	Cercopithecidae
XP_003914306/AHZZ01033905	AQP4	Olive baboon	*Papio anubis*	Euarchontoglires	Primates	Cercopithecidae
ENSCJAP00000023226	AQP4	White-tufted-ear marmoset	*Callithrix jacchus*	Euarchontoglires	Primates	Cebidae
XP_003924812	AQP4	Bolivian squirrel monkey	*Saimiri boliviensis boliviensis*	Euarchontoglires	Primates	Cebidae
ENSTSYP00000001315	AQP4	Philippine tarsier	*Tarsius syrichta*	Euarchontoglires	Primates	Tarsiidae
ENSMICP00000002129	AQP4	Gray mouse lemur	*Microcebus murinus*	Euarchontoglires	Primates	Cheirogaleidae
XP_003784818/ENSOGAP00000003480	AQP4	Small-eared galago/Bushbaby	*Otolemur garnettii*	Euarchontoglires	Primates	Galagidae
ENSTBEP00000006848	AQP4	Northern tree shrew	*Tupaia belangeri*	Euarchontoglires	Scandentia	Tupaiidae
ELW61644	AQP4	Chinese tree shrew	*Tupaia chinensis*	Euarchontoglires	Scandentia	Tupaiidae
ENSMUSP00000078088	AQP4	Mouse	*Mus musculus*	Euarchontoglires	Rodentia	Muridae
ENSRNOP00000021962	AQP4	Norway rat	*Rattus norvegicus*	Euarchontoglires	Rodentia	Muridae
XP_003502272	AQP4	Chinese hamster	*Cricetulus griseus*	Euarchontoglires	Rodentia	Cricetidae
XP_005355847	AQP4	Prairie vole	*Microtus ochrogaster*	Euarchontoglires	Rodentia	Cricetidae
AKZC01083494/AKZC01083493	AQP4	Lesser Egyptian jerboa	*Jaculus jaculus*	Euarchontoglires	Rodentia	Dipodidae
ENSDORP00000007647	AQP4	Ord's kangaroo rat	*Dipodomys ordii*	Euarchontoglires	Rodentia	Heteromyidae
AAK66823	AQP4	Merriam's kangaroo rat	*Dipodomys merriami*	Euarchontoglires	Rodentia	Heteromyidae
XP_004623603	AQP4	Degu		Euarchontoglires	Rodentia	Octodontidae
ENSCPOP00000004012	AQP4	Domestic guinea pig	*Cavia porcellus*	Euarchontoglires	Rodentia	Caviidae
EHB09536	AQP4	Naked mole-rat	*Heterocephalus glaber*	Euarchontoglires	Rodentia	Bathyergidae
ENSSTOP00000011934	AQP4	Thirteen-lined ground squirrel	*Ictidomys tridecemlineatus*	Euarchontoglires	Rodentia	Sciuridae
XP_005415161	AQP4	Long-tailed chinchilla	*Chinchilla lanigera*	Euarchontoglires	Rodentia	Chinchillidae
ENSOPRP00000008537	AQP4	American pika	*Ochotona princeps*	Euarchontoglires	Lagomorpha	Ochotonidae
ENSOCUP00000007625	AQP4	Rabbit	*Oryctolagus cuniculus*	Euarchontoglires	Lagomorpha	Leporidae
AMDU01061559	AQP4	Western European hedgehog	*Erinaceus europaeus*	Laurasiatheria	Insectivora	Erinaceinae
ENSSARP00000010861	AQP4	European shrew	*Sorex araneus*	Laurasiatheria	Insectivora	Soricidae
AJFV01043685	AQP4	Star-nosed mole	*Condylura cristata*	Laurasiatheria	Insectivora	Talpidae
ENSPVAP00000006319	AQP4	Large flying fox/Megabat	*Pteropus vampyrus*	Laurasiatheria	Chiroptera	Pteropodidae
ELK05687/ALWS01088995	AQP4	Black flying fox	*Pteropus alecto*	Laurasiatheria	Chiroptera	Pteropodidae
AWHC01004955	AQP4	Straw-colored fruit bat	*Eidolon helvum*	Laurasiatheria	Chiroptera	Pteropodidae
ENSMLUP00000010006	AQP4	Little brown bat/Microbat	*Myotis lucifugus*	Laurasiatheria	Chiroptera	Vespertilionidae
ELK29349	AQP4	David´s myotis	*Myotis davidii*	Laurasiatheria	Chiroptera	Vespertilionidae
XP_005857129	AQP4	Brandt's bat	*Myotis brandtii*	Laurasiatheria	Chiroptera	Vespertilionidae
AWHA01245409	AQP4	Greater horseshoe bat	*Rhinolophus ferrumequinum*	Laurasiatheria	Chiroptera	Rhinolophinae
AWGZ01135479	AQP4	Parnell's mustached bat	*Pteronotus parnellii*	Laurasiatheria	Chiroptera	Mormoopidae
AWHB01024273/AWHB01024274/AWHB01024275	AQP4	Indian false vampire	*Megaderma lyra*	Laurasiatheria	Chiroptera	Megadermatidae
NP_001009279	AQP4	Sheep	*Ovis aries*	Laurasiatheria	Ruminantia	Bovidae
AJPT01207634	AQP4	Goat	*Capra hircus*	Laurasiatheria	Ruminantia	Bovidae
XP_005982667/XP_005982668	AQP4	Chiru	*Pantholops hodgsonii*	Laurasiatheria	Ruminantia	Bovidae

ENSBTAP00000025341	AQP4	Cow	*Bos taurus*	Laurasiatheria	Ruminantia	Bovidae
ELR49016	AQP4	Yak	*Bos mutus*	Laurasiatheria	Ruminantia	Bovidae
ENSTTRP00000011193	AQP4	Bottlenosed dolphin	*Tursiops truncatus*	Laurasiatheria	Cetacea	Delphinidae
AGV55483	AQP4	Long-beaked common dolphin	*Delphinus capensis*	Laurasiatheria	Cetacea	Delphinidae
AGV55482	AQP4	Indo-pacific humpbacked dolphin	*Sousa chinensis*	Laurasiatheria	Cetacea	Delphinidae
AGV55484	AQP4	Risso's dolphin	*Grampus griseus*	Laurasiatheria	Cetacea	Delphinidae
ANOL02035809/XP_004273776	AQP4	Killer whale	*Orcinus orca*	Laurasiatheria	Cetacea	Delphinidae
AUPI01064204/AGV55488	AQP4	Yangtze River dolphin	*Lipotes vexillifer*	Laurasiatheria	Cetacea	Lipotidae
AGV55486	AQP4	Yangtze finless porpoise	*Neophocaena asiaeorientalis*	Laurasiatheria	Cetacea	Phocoenidae
AGV55487	AQP4	Indo-Pacific finless porpoise	*Neophocaena phocaenoides*	Laurasiatheria	Cetacea	Phocoenidae
AGV55485	AQP4	Beluga whale	*Delphinapterus leucas*	Laurasiatheria	Cetacea	Monodontidae
AWZP01016437	AQP4	Sperm whale	*Physeter catodon*	Laurasiatheria	Cetacea	Physeteridae
AGV55490	AQP4	Dwarf sperm whale	*Kogia sima*	Laurasiatheria	Cetacea	Physeteridae
AGV55489	AQP4	Blainville's beaked whale	*Mesoplodon densirostris*	Laurasiatheria	Cetacea	Ziphiidae
ATDI01150330/AGV55491	AQP4	Minke whale	*Balaenoptera acutorostrata*	Laurasiatheria	Cetacea	Balaenopteridae
ENSSSCP00000004028	AQP4	Pig	*Sus scrofa*	Laurasiatheria	Suina	Suidae
ENSVPAP00000002491	AQP4	Alpaca	*Vicugna pacos*	Laurasiatheria	Tylopoda	Camelidae
AGVR01035798	AQP4	Wild Bactrian camel	*Camelus ferus*	Laurasiatheria	Tylopoda	Camelidae
ENSECAP00000003378	AQP4	Horse	*Equus caballus*	Laurasiatheria	Perissodactyla	Equidae
AKZM01006356	AQP4	Southern white rhinocerus	*Ceratotherium simum simum*	Laurasiatheria	Perissodactyla	Rhinocerotidae
ENSAMEP00000014983	AQP4	Giant panda	*Ailuropoda melanoleuca*	Laurasiatheria	Carnivora	Ursidae
GAJD01028718	AQP4	Polar bear	*Ursus maritimus*	Laurasiatheria	Carnivora	Ursidae
ENSCAFP00000026782	AQP4	Dog	*Canis lupus familiaris*	Laurasiatheria	Carnivora	Canidae
ENSMPUP00000006534	AQP4	Domestic ferret	*Mustela putorius furo*	Laurasiatheria	Carnivora	Mustelidae
XP_003995137	AQP4	Domestic cat	*Felis catus*	Laurasiatheria	Carnivora	Felidae
ATCQ01019693/ATCQ01019692	AQP4	Amur tiger	*Panthera tigris altaica*	Laurasiatheria	Carnivora	Felidae
APMU01121065	AQP4	Weddell seal	*Leptonychotes weddellii*	Laurasiatheria	Carnivora	Phocidae
ANOP01042223	AQP4	Pacific walrus	*Odobenus rosmarus divergens*	Laurasiatheria	Carnivora	Odobenidae
ENSLAFP00000005168	AQP4	African savanna elephant	*Loxodonta africana*	Afrotheria	Proboscidea	Elephantidae
AHIN01127370	AQP4	Florida manatee	*Trichechus manatus latirostris*	Afrotheria	Sirenia	Trichechidae
ENSPCAP00000003928	AQP4	Cape rock hyrax	*Procavia capensis*	Afrotheria	Hyracoidea	Procaviidae
ENSETEP00000005713/AAIY02093242	AQP4	Lesser hedgehog tenrec	*Echinops telfairi*	Afrotheria	Afrosoricida	Tenrecidae
ALYB01054355	AQP4	Ardvaark	*Orycteropus afer afer*	Afrotheria	Tubulidentata	Orycteropodidae
ENSDNOP00000010511	AQP4	Nine-banded armadillo	*Dasypus novemcinctus*	Xenarthra	Cingulata	Dasypodidae
ENSMEUP0000001264	AQP4	Tammar wallaby	*Macropus eugenii*	Metatheria	Diprotodontia	Macropodidae
ENSSHAP00000017636	AQP4	Tasmanian devil	*Sarcophilus harrisii*	Metatheria	Dasyuromorphia	Dasyuridae
ENSMODP00000026878	AQP4	Gray short-tailed opossum	*Monodelphis domestica*	Metatheria	Didelphimorphia	Didelphidae
ENSOANP00000014435	AQP4	Platypus	*Ornithorhynchus anatinus*	Prototheria	Monotremata	Ornithorhynchidae
AGAI01067595	AQP4	Budgerigar	*Melopsittacus undulatus*	Aves	Psittaciformes	Psittacidae
AOCU01015563/AOCU01012416	AQP4	Puerto Rican parrot	*Amazona vittata*	Aves	Psittaciformes	Psittacidae
AMXX01100436/AOUJ01146850/AOUJ01146849	AQP4	Scarlet macaw	*Ara macao*	Aves	Psittaciformes	Psittacidae
JV182330	AQP4	Duck-eyed junco	*Junco hyemalis*	Aves	Passeriformes	Fringillidae
CAVT010001060	AQP4	Common canary	*Serinus canaria*	Aves	Passeriformes	Fringillidae
AKZB01037819	AQP4	Medium ground finch	*Geospiza fortis*	Aves	Passeriformes	Fringillidae
XP_005479315	AQP4	White-throated sparrow	*Zonotrichia albicollis*	Aves	Passeriformes	Fringillidae
HX772987	AQP4	Bengalese finch	*Lonchura striata domestica*	Aves	Passeriformes	Estrildidae
ENSTGUP00000010943	AQP4	Zebra finch	*Taeniopygia guttata*	Aves	Passeriformes	Estrildidae
ANZD01025686	AQP4	Tibetan ground-tit	*Pseudopodoces humilis*	Aves	Passeriformes	Paridae
JR866965	AQP4	Vinous-throated parrotbill	*Paradoxornis webbianus*	Aves	Passeriformes	Muscicapidae
AGTO01010368	AQP4	Collared flycatcher	*Ficedula albicollis*	Aves	Passeriformes	Muscicapidae
AKMT01060575	AQP4	Peregrin falcon	*Falco peregrinus*	Aves	Falconiformes	Falconidae
AKMU01012343	AQP4	Saker falcon	*Falco cherrug*	Aves	Falconiformes	Falconidae
EMC81065	AQP4	Rock pigeon	*Columba livia*	Aves	Columbiformes	Columbidae
ENSAPLP00000003539	AQP4	Mallard	*Anas platyrhynchos*	Aves	Anseriformes	Anatidae
AAL73511	AQP4	Common quail	*Coturnix coturnix*	Aves	Galliformes	Phasianidae
ENSMGAP00000010781	AQP4	Turkey	*Meleagris gallopavo*	Aves	Galliformes	Phasianidae
ENSGALP00000024367	AQP4	Chicken	*Gallus gallus*	Aves	Galliformes	Phasianidae
AKHW01033483	AQP4	American alligator	*Alligator mississippiensis*	Archosauria	Crocodylia	Alligatoridae
AVPB01164972	AQP4	Chinese alligator	*Alligator sinensis*	Archosauria	Crocodylia	Alligatoridae
AGCU01113997/ENSPSIP00000002695	AQP4	Chinese softshell turtle	*Pelodiscus sinensis*	Sauropsida	Testudines	Trionychidae
APJP01627304/APJP01719894	AQP4	Spiny softshell turtle	*Apalone spinifera*	Sauropsida	Testudines	Trionychidae
AJIM01255269	AQP4	Green seaturtle	*Chelonia mydas*	Sauropsida	Testudines	Cheloniidae
AHGY01172519	AQP4	Western painted turtle	*Chrysemys picta bellii*	Sauropsida	Testudines	Emydidae
ETE65176	AQP4	King cobra	*Ophiophagus hannah*	Lepidosauria	Squamata	Elapidae
AEQU02020963/AEQU02020962/AEQU02020961	AQP4	Burmese python	*Python molurus bivittatus*	Lepidosauria	Squamata	Pythonidae
AAWZ02009571/ENSACAP00000002099	AQP4	Green anole	*Anolis carolinensis*	Lepidosauria	Squamata	Iguanidae
NP_001124421	AQP4	African clawed frog	*Xenopus laevis*	Amphibia	Anura	Pipidae
AAMC02021699/ENSXETP00000061647	AQP4	Western clawed frog	*Xenopus (Silurana) tropicalis*	Amphibia	Anura	Pipidae
GAQK01110609/GAQK01053720	AQP4	Chinese salamander	*Hynobius chinensis*	Amphibia	Caudata	Hynobiidae
NP_001135583/ENSXETP00000022885/AAMC02021400	AQP4n1	Western clawed frog	*Xenopus (Silurana) tropicalis*	Amphibia	Anura	Pipidae
GAQK01086512/GAQK01053719	AQP4n1	Chinese salamander	*Hynobius chinensis*	Amphibia	Caudata	Hynobiidae
CN057663	AQP4n1	Eastern tiger salamander	*Ambystoma tigrinum tigrinum*	Amphibia	Caudata	Ambystomatidae
AAMC02021615	AQP4n2	Western clawed frog	*Xenopus (Silurana) tropicalis*	Amphibia	Anura	Pipidae

AFYH01162813/AFYH01162814/AFYH01162815	Aqp4	Coelacanth	*Latimeria chalumnae*	Actinistia	Coelacanthiformes	Coelacanthidae
GAPS01045174	Aqp4	Menado coelacanth	*Latimeria menadoensis*	Actinistia	Coelacanthiformes	Coelacanthidae
ENSTRUP00000021667	Aqp4a	Torafugu	*Takifugu rubripes*	Acanthopterygii	Tetraodontiformes	Tetraodontidae
AOOT01024085/AOOT01024086/AOOT01024087/ AOOT01024088/AOOT01024089	Aqp4a	Sansaifugu	*Takifugu flavidus*	Acanthopterygii	Tetraodontiformes	Tetraodontidae
BAL44699	Aqp4a	Mefugu	*Takifugu obscurus*	Acanthopterygii	Tetraodontiformes	Tetraodontidae
ENSTNIP00000018275	Aqp4a	Green-spotted pufferfish	*Tetraodon nigroviridis*	Acanthopterygii	Tetraodontiformes	Tetraodontidae
GAIB01123840	Aqp4a	Turbot	*Scophthalmus maximus*	Acanthopterygii	Pleuronectiformes	Scophthalmidae
AGRG01030937	Aqp4a	Tongue sole	*Cynoglossus semilaevis*	Acanthopterygii	Pleuronectiformes	Cynoglossidae
FM156410	Aqp4a	Gilthead seabream	*Sparus aurata*	Acanthopterygii	Perciformes	Sparidae
FM027211/FM008510	Aqp4a	European seabass	*Dicentrarchus labrax*	Acanthopterygii	Perciformes	Moronidae
AGTA02006339	Aqp4a	Zebra mbuna	*Maylandia zebra*	Acanthopterygii	Perciformes	Cichlidae
AFNX01002653	Aqp4a	Red mwanza	*Pundamilia nyererei*	Acanthopterygii	Perciformes	Cichlidae
AFNY01017713	Aqp4a	Lyretail cichlid	*Neolamprologus brichardi*	Acanthopterygii	Perciformes	Cichlidae
AFNZ01000609	Aqp4a	Burton's mouthbrooder	*Haplochromis burtoni*	Acanthopterygii	Perciformes	Cichlidae
ENSONIP00000012221	Aqp4a	Nile tilapia	*Oreochromis niloticus*	Acanthopterygii	Perciformes	Cichlidae
CBH76623	Aqp4a	Mozamique tilapia	*Oreochromis mossambicus*	Acanthopterygii	Perciformes	Cichlidae
BADN01019226/BADN01019225	Aqp4a	Pacific bluefin tuna	*Thunnus orientalis*	Acanthopterygii	Perciformes	Scombridae
GAJJ01007846/AWGY01141004	Aqp4a	Sablefish	*Anoplopoma fimbria*	Acanthopterygii	Scorpaeniformes	Anoplopomatidae
AUPQ01003929	Aqp4a	Flag rockfish	*Sebastes rubrivinctus*	Acanthopterygii	Scorpaeniformes	Sebastidae
AUPR01036549	Aqp4a	Tiger rockfish	*Sebastes nigrocinctus*	Acanthopterygii	Scorpaeniformes	Sebastidae
ENSGACP00000017439	Aqp4a	Three-spined stickleback	*Gasterosteus aculeatus*	Acanthopterygii	Gasterosteiformes	Gasterosteidae
ENSORLP00000021288	Aqp4a	Japanese medaka	*Oryzias latipes*	Acanthopterygii	Beloniformes	Adrianichthyidae
GAIB01200326	Aqp4a	Turquoise killifish	*Nothobranchius furzeri*	Acanthopterygii	Cyprinodontiformes	Nothobranchiidae
AYCK01007792	Aqp4a	Amazon molly	*Poecilia formosa*	Acanthopterygii	Cyprinodontiformes	Poeciliidae
AGAJ01002422/ENSXMAP00000016690	Aqp4a	Southern platyfish	*Xiphophorus maculatus*	Acanthopterygii	Cyprinodontiformes	Poeciliidae
ENSGMOP00000003113	Aqp4a	Atlantic cod	*Gadus morhua*	Paracanthopterygii	Gadiformes	Gadidae
BX885214/CCAF010050670	Aqp4a1	Rainbow trout	*Oncorhynchus mykiss*	Protacanthopterygii	Salmoniformes	Salmonidae
AGKD01106121	Aqp4a1	Atlantic salmon	*Salmo salar*	Protacanthopterygii	Salmoniformes	Salmonidae
CCAF010007960	Aqp4a2	Rainbow trout	*Oncorhynchus mykiss*	Protacanthopterygii	Salmoniformes	Salmonidae
AGKD01009294	Aqp4a2	Atlantic salmon	*Salmo salar*	Protacanthopterygii	Salmoniformes	Salmonidae
APWO01102777/APWO01102776/APWO01102775	Aqp4a	Mexican tetra	*Astyanax mexicanus*	Ostariophysi	Characiformes	Characidae
JT417843	Aqp4a	Channel catfish	*Ictalurus punctatus*	Ostariophysi	Siluriformes	Ictaluridae
GAGX01016516/GAGX01123310/GAGX01041313	Aqp4a	Brown bullhead	*Ameiurus nebulosus*	Ostariophysi	Siluriformes	Ictaluridae
AVPY01087012/AVPY01087011/AVPY01087010	Aqp4a1	Japanese eel	*Anguilla japonica*	Elopomorpha	Anguilliformes	Anguillidae
AVPY01209060/AVPY01568671/AVPY01209065	Aqp4a2	Japanese eel	*Anguilla japonica*	Elopomorpha	Anguilliformes	Anguillidae
AGTA02032263	Aqp4b	Zebra mbuna	*Maylandia zebra*	Acanthopterygii	Perciformes	Cichlidae
AFNX01020326	Aqp4b	Red mwanza	*Pundamilia nyererei*	Acanthopterygii	Perciformes	Cichlidae
AFNY01027610	Aqp4b	Lyretail cichlid	*Neolamprologus brichardi*	Acanthopterygii	Perciformes	Cichlidae
AFNZ01011199	Aqp4b	Burton's mouthbrooder	*Haplochromis burtoni*	Acanthopterygii	Perciformes	Cichlidae
ENSONIP00000009148	Aqp4b	Nile tilapia	*Oreochromis niloticus*	Acanthopterygii	Perciformes	Cichlidae
BADN01048260/BADN01048261	Aqp4b	Pacific bluefin tuna	*Thunnus orientalis*	Acanthopterygii	Perciformes	Scombridae
AWGY01079741/AWGY01034995	Aqp4b	Sablefish	*Anoplopoma fimbria*	Acanthopterygii	Scorpaeniformes	Anoplopomatidae
AUPQ01058153/AUPQ01055846	Aqp4b	Flag rockfish	*Sebastes rubrivinctus*	Acanthopterygii	Scorpaeniformes	Sebastidae
AUPR01075041/AUPR01118149	Aqp4b	Tiger rockfish	*Sebastes nigrocinctus*	Acanthopterygii	Scorpaeniformes	Sebastidae
CCAF010009104	Aqp4b1	Rainbow trout	*Oncorhynchus mykiss*	Protacanthopterygii	Salmoniformes	Salmonidae
AGKD01074371	Aqp4b1	Atlantic salmon	*Salmo salar*	Protacanthopterygii	Salmoniformes	Salmonidae
CCAF010095299	Aqp4b2	Rainbow trout	*Oncorhynchus mykiss*	Protacanthopterygii	Salmoniformes	Salmonidae
AGKD01045191	Aqp4b2	Atlantic salmon	*Salmo salar*	Protacanthopterygii	Salmoniformes	Salmonidae
GAHO01058079/GAHO01008893	Aqp4b	Small gill opening goldenline barbel	*Sinocyclocheilus angustiporus*	Ostariophysi	Cypriniformes	Cyprinidae
GAHL01119850/GAHL01044412	Aqp4b	Blind goldenline barbel	*Sinocyclocheilus anophthalmus*	Ostariophysi	Cypriniformes	Cyprinidae
FJ666327/ENSDARP00000021578	Aqp4b	Zebrafish	*Danio rerio*	Ostariophysi	Cypriniformes	Cyprinidae
APWO01019403	Aqp4b	Mexican tetra	*Astyanax mexicanus*	Ostariophysi	Characiformes	Characidae
GAGX01016516/GAGX01123310	Aqp4b	Brown bullhead	*Ameiurus nebulosus*	Ostariophysi	Siluriformes	Ictaluridae
AVPY01122738/AVPY01122737	Aqp4b1	Japanese eel	*Anguilla japonica*	Elopomorpha	Anguilliformes	Anguillidae
AVPY01196169	Aqp4b2	Japanese eel	*Anguilla japonica*	Elopomorpha	Anguilliformes	Anguillidae
AHAT01033601	Aqp4	Spotted gar	*Lepisosteus oculatus*	Holostei	Semionotiformes	Lepisosteidae
KJ784517	Aqp4	Smaller spotted catshark	*Scyliorhinus canicula*	Chondrichthyes	Carcharhiniformes	Scyliorhinidae
AEJ08190	Aqp4	Spiny dogfish	*Squalus acanthias*	Chondrichthyes	Squaliformes	Squalidae
AESE011524036/AESE010005714/AESE012049129	Aqp4	Little skate	*Leucoraja erinacea*	Chondrichthyes	Rajiformes	Rajidae
AAVX02013228	Aqp4	Ghost shark	*Callorhinchus milii*	Chondrichthyes	Chimaeriformes	Callorhinchidae
ENSPMAP00000008913	Aqp4	Sea lamprey	*Petromyzon marinus*	Hyperoartia	Petromyzontiformes	Petromyzontidae
APJL01016226/APJL01016225	Aqp4	Arctic lamprey	*Lethenteron camtschaticum*	Hyperoartia	Petromyzontiformes	Petromyzontidae
APJL01097212/APJL01119505	Aqp4h	Arctic lamprey	*Lethenteron camtschaticum*	Hyperoartia	Petromyzontiformes	Petromyzontidae
BAE93686	Aqp4	Inshore hagfish	*Eptatretus burgeri*	Hyperotreti	Myxiniformes	Myxinidae
ENSCINP00000007510	Aqp4L	Vase tunicate	*Ciona intestinalis*	Tunicata	Enterogona	Cionidae
AACT01066003	Aqp4L	Pacific transparent sea squirt	*Ciona savignyi*	Tunicata	Enterogona	Cionidae
XP_002612400/ABEP02002539/FE561739/ XP_002213012/XP_002215983	Aqp4L1	Florida lancelet	*Branchiostoma floridae*	Cephalochordata	Amphioxiformes	Branchiostomidae
XP_002592465	Aqp4L2	Florida lancelet	*Branchiostoma floridae*	Cephalochordata	Amphioxiformes	Branchiostomidae
ACQM01032646/ACQM01032648/XP_002735112	Aqp4L1	Acorn worm	*Saccoglossus kowalevskii*	Hemichordata	Enteropneusta	Harrimaniidae
XP_002737956/ACQM01066396	Aqp4L2	Acorn worm	*Saccoglossus kowalevskii*	Hemichordata	Enteropneusta	Harrimaniidae
XP_002733505/ACQM01011882/ACQM01041644	Aqp4L3	Acorn worm	*Saccoglossus kowalevskii*	Hemichordata	Enteropneusta	Harrimaniidae
NP_001158487/ACQM01049774/ACQM01049776	Aqp4L4	Acorn worm	*Saccoglossus kowalevskii*	Hemichordata	Enteropneusta	Harrimaniidae

XP_002736465/ACQM01049776/ACQM01049777/ ACQM01049779	Aqp4L5	Acorn worm	*Saccoglossus kowalevskii*	Hemichordata	Enteropneusta	Harrimaniidae
XP_001185961/AAGJ04084831/AAGJ04084830/ SPU_012222-tr	Aqp4L1	Purple sea urchin	*Strongylocentrotus purpuratus*	Echinodermata	Echinozoa	Strongylocentrotida
GAPB01022147	Aqp4L1	Kina	*Evechinus chloroticus*	Echinodermata	Echinozoa	Echinometridae
AGCV01326836/AGCV01326841	Aqp4L1	Green sea urchin	*Lytechinus variegatus*	Echinodermata	Echinozoa	Toxopneustidae
AKZP01034236	Aqp4L1	Bat star	*Patiria miniata*	Echinodermata	Asterozoa	Asterinidae
AM546738/AM564397/AM546840	Aqp4L2	Common sea urchin	*Paracentrotus lividus*	Echinodermata	Echinozoa	Echinidae
XP_799266/JT104793/AAGJ04146714/ SPU_021388-tr	Aqp4L2	Purple sea urchin	*Strongylocentrotus purpuratus*	Echinodermata	Echinozoa	Strongylocentrotida
GAPB01002239	Aqp4L2	Kina	*Evechinus chloroticus*	Echinodermata	Echinozoa	Echinometridae
AGCV01123237	Aqp4L2	Green sea urchin	*Lytechinus variegatus*	Echinodermata	Echinozoa	Toxopneustidae
XP_003727808/JT111244/AAGJ04146718/ SPU_021387-tr	Aqp4L3	Purple sea urchin	*Strongylocentrotus purpuratus*	Echinodermata	Echinozoa	Strongylocentrotida
GAPB01026719	Aqp4L3	Kina	*Evechinus chloroticus*	Echinodermata	Echinozoa	Echinometridae
AGCV01123236/AGCV01326608/AGCV01123241/ JI430437	Aqp4L3	Green sea urchin	*Lytechinus variegatus*	Echinodermata	Echinozoa	Toxopneustidae
AKZP01139048/AKZP01127451	Aqp4L2	Bat star	*Patiria miniata*	Echinodermata	Asterozoa	Asterinidae
AM210830/AM555046	Aqp4L4	Common sea urchin	*Paracentrotus lividus*	Echinodermata	Echinozoa	Echinidae
JT120368/XP_001190612/AAGJ04168952/ AAGJ04168954/SPU_008301-tr	Aqp4L4	Purple sea urchin	*Strongylocentrotus purpuratus*	Echinodermata	Echinozoa	Strongylocentrotida
GAPB01048960	Aqp4L4	Kina	*Evechinus chloroticus*	Echinodermata	Echinozoa	Echinometridae
AGCV01410707/AGCV01020101	Aqp4L4	Green sea urchin	*Lytechinus variegatus*	Echinodermata	Echinozoa	Toxopneustidae
JI332114	Aqp4L4	Slate pencil urchin	*Eucidaris tribuloides*	Echinodermata	Echinozoa	Cidaridae
AKZP01081306/AKZP01035467	Aqp4L4	Bat star	*Patiria miniata*	Echinodermata	Asterozoa	Asterinidae
AAGJ04168954/SPU_007511-tr	Aqp4L5	Purple sea urchin	*Strongylocentrotus purpuratus*	Echinodermata	Echinozoa	Strongylocentrotida
AGCV01037590	Aqp4L5	Green sea urchin	*Lytechinus variegatus*	Echinodermata	Echinozoa	Toxopneustidae
AKZP01081328/AKZP01081329	Aqp4L5	Bat star	*Patiria miniata*	Echinodermata	Asterozoa	Asterinidae

Radiata AQP4L orthologs

BACK01018438	Aqp4L1	Stony coral	*Acropora digitifera*	Cnidaria	Scleractinia	Acroporidae
JT015911	Aqp4L1	Stony coral	*Acropora millepora*	Cnidaria	Scleractinia	Acroporidae
GASU01031648/GASU01030213	Aqp4L1	Stony coral	*Acropora cervicornis*	Cnidaria	Scleractinia	Acroporidae
GARY01002118	Aqp4L1	Stony coral	*Stylophora pistillata*	Cnidaria	Scleractinia	Pocilloporidae
FX451850	Aqp4L1	Stony coral	*Porites australiensis*	Cnidaria	Scleractinia	Poritidae
v1g185753/XP_001633187	Aqp4L1	Starlet sea anemone	*Nematostella vectensis*	Cnidaria	Actiniaria	Edwardsiidae
BACK01018439	Aqp4L2	Stony coral	*Acropora digitifera*	Cnidaria	Scleractinia	Acroporidae
JT000077	Aqp4L2	Stony coral	*Acropora millepora*	Cnidaria	Scleractinia	Acroporidae
GASU01069771	Aqp4L2	Stony coral	*Acropora cervicornis*	Cnidaria	Scleractinia	Acroporidae
GARY01039993	Aqp4L2	Stony coral	*Stylophora pistillata*	Cnidaria	Scleractinia	Pocilloporidae
JV113280	Aqp4L2	Symbiotic anemone	*Aiptasia pallida*	Cnidaria	Actiniaria	Aiptasiidae
v1g206913/XP_001633227	Aqp4L2	Starlet sea anemone	*Nematostella vectensis*	Cnidaria	Actiniaria	Edwardsiidae
BACK01018439	Aqp4L3	Stony coral	*Acropora digitifera*	Cnidaria	Scleractinia	Acroporidae
JT013618	Aqp4L3	Stony coral	*Acropora millepora*	Cnidaria	Scleractinia	Acroporidae
GASU01060721	Aqp4L3	Stony coral	*Acropora cervicornis*	Cnidaria	Scleractinia	Acroporidae
GARY01017733	Aqp4L3	Stony coral	*Stylophora pistillata*	Cnidaria	Scleractinia	Pocilloporidae
v1g24968/XP_001633212	Aqp4L3	Starlet sea anemone	*Nematostella vectensis*	Cnidaria	Actiniaria	Edwardsiidae
BACK01049583/BACK01046109	Aqp4L4	Stony coral	*Acropora digitifera*	Cnidaria	Scleractinia	Acroporidae
JR987402	Aqp4L4	Stony coral	*Acropora millepora*	Cnidaria	Scleractinia	Acroporidae
GASU01086600	Aqp4L4	Stony coral	*Acropora cervicornis*	Cnidaria	Scleractinia	Acroporidae
GARY01004248	Aqp4L4	Stony coral	*Stylophora pistillata*	Cnidaria	Scleractinia	Pocilloporidae
FX456176	Aqp4L4	Stony coral	*Porites australiensis*	Cnidaria	Scleractinia	Poritidae
JV084153	Aqp4L4	Symbiotic anemone	*Aiptasia pallida*	Cnidaria	Actiniaria	Aiptasiidae
v1g140371/XP_001622649	Aqp4L4	Starlet sea anemone	*Nematostella vectensis*	Cnidaria	Actiniaria	Edwardsiidae
XP_002157029	Aqp4L	Freshwater anemone	*Hydra vulgaris*	Cnidaria	Hydroida	Hydridae
FP931643	Aqp4La	Thecate hydroid	*Clytia hemisphaerica*	Cnidaria	Hydroida	Campanulariidae
FP965297	Aqp4Lb	Thecate hydroid	*Clytia hemisphaerica*	Cnidaria	Hydroida	Campanulariidae
CU430818	Aqp4Lc	Thecate hydroid	*Clytia hemisphaerica*	Cnidaria	Hydroida	Campanulariidae

AQP5

ENSP00000293599	AQP5	Human	*Homo sapiens*	Euarchontoglires	Primates	Hominidae
ENSPTRP00000008403	AQP5	Chimpanzee	*Pan troglodytes*	Euarchontoglires	Primates	Hominidae
ENSGGOP00000005775	AQP5	Western lowland gorilla	*Gorilla gorilla gorilla*	Euarchontoglires	Primates	Hominidae
ENSPPYP00000005139	AQP5	Sumatran orangutan	*Pongo abelii*	Euarchontoglires	Primates	Hominidae
ENSNLEP00000021638	AQP5	Northern white-cheeked gibbon	*Nomascus leucogenys*	Euarchontoglires	Primates	Hylobatidae
XP_001110608	AQP5	Rhesus macaque	*Macaca mulatta*	Euarchontoglires	Primates	Cercopithecidae
XP_005570852	AQP5	Crab-eating macaque	*Macaca fascicularis*	Euarchontoglires	Primates	Cercopithecidae
AHZZ01018917	AQP5	Hamadryas baboon	*Papio hamadryas*	Euarchontoglires	Primates	Cercopithecidae
XP_003906402	AQP5	Olive baboon	*Papio anubis*	Euarchontoglires	Primates	Cercopithecidae
ENSCJAP00000039181	AQP5	White-tufted-ear marmoset	*Callithrix jacchus*	Euarchontoglires	Primates	Cebidae
XP_003939195	AQP5	Bolivian squirrel monkey	*Saimiri boliviensis boliviensis*	Euarchontoglires	Primates	Cebidae
ENSMICP00000014213	AQP5	Gray mouse lemur	*Microcebus murinus*	Euarchontoglires	Primates	Cheirogaleidae
XP_003793671/ENSOGAP00000014952	AQP5	Small-eared galago/Bushbaby	*Otolemur garnettii*	Euarchontoglires	Primates	Galagidae
ENSTBEP00000010797	AQP5	Northern tree shrew	*Tupaia belangeri*	Euarchontoglires	Scandentia	Tupaiidae
ALAR01214976	AQP5	Chinese tree shrew	*Tupaia chinensis*	Euarchontoglires	Scandentia	Tupaiidae
ENSMUSP00000048739	AQP5	Mouse	*Mus musculus*	Euarchontoglires	Rodentia	Muridae
ENSRNOP00000024102	AQP5	Norway rat	*Rattus norvegicus*	Euarchontoglires	Rodentia	Muridae

EGV93598	AQP5	Chinese hamster	*Cricetulus griseus*	Euarchontoglires	Rodentia	Cricetidae
XP_005353968	AQP5	Prairie vole	*Microtus ochrogaster*	Euarchontoglires	Rodentia	Cricetidae
AKZC01010084	AQP5	Lesser Egyptian jerboa	*Jaculus jaculus*	Euarchontoglires	Rodentia	Dipodidae
ENSDORP00000013586	AQP5	Ord's kangaroo rat	*Dipodomys ordii*	Euarchontoglires	Rodentia	Heteromyidae
ENSCPOP00000016163	AQP5	Domestic guinea pig	*Cavia porcellus*	Euarchontoglires	Rodentia	Caviidae
EHB00519	AQP5	Naked mole-rat	*Heterocephalus glaber*	Euarchontoglires	Rodentia	Bathyergidae
ENSSTOP00000010100	AQP5	Thirteen-lined ground squirrel	*Ictidomys tridecemlineatus*	Euarchontoglires	Rodentia	Sciuridae
XP_005411914	AQP5	Long-tailed chinchilla	*Chinchilla lanigera*	Euarchontoglires	Rodentia	Chinchillidae
ALIT01111584/ENSOPRP00000007466	AQP5	American pika	*Ochotona princeps*	Euarchontoglires	Lagomorpha	Ochotonidae
AAGW02058904/ENSOCUP00000003998/ XP_002711143	AQP5	Rabbit	*Oryctolagus cuniculus*	Euarchontoglires	Lagomorpha	Leporidae
ENSEEUP00000005123	AQP5	Western European hedgehog	*Erinaceus europaeus*	Laurasiatheria	Insectivora	Erinaceinae
BAF96781	AQP5	House shrew	*Suncus murinus*	Laurasiatheria	Insectivora	Soricidae
AALT02012851	AQP5	European shrew	*Sorex araneus*	Laurasiatheria	Insectivora	Soricidae
AJFV01069455/AJFV01069456	AQP5	Star-nosed mole	*Condylura cristata*	Laurasiatheria	Insectivora	Talpidae
ENSPVAP00000006321	AQP5	Large flying fox/Megabat	*Pteropus vampyrus*	Laurasiatheria	Chiroptera	Pteropodidae
ALWS01070686/ELK08376	AQP5	Black flying fox	*Pteropus alecto*	Laurasiatheria	Chiroptera	Pteropodidae
JAA51177	AQP5	Common vampire bat	*Desmodus rotundus*	Laurasiatheria	Chiroptera	Phyllostomidae
ENSMLUP00000014522	AQP5	Little brown bat/Microbat	*Myotis lucifugus*	Laurasiatheria	Chiroptera	Vespertilionidae
XP_005877372	AQP5	Brandt's bat	*Myotis brandtii*	Laurasiatheria	Chiroptera	Vespertilionidae
ENSBTAP00000038149	AQP5	Sheep	*Ovis aries*	Laurasiatheria	Ruminantia	Bovidae
AJPT01054264/ XP_005680167	AQP5	Goat	*Capra hircus*	Laurasiatheria	Ruminantia	Bovidae
XP_005981102	AQP5	Chiru	*Pantholops hodgsonii*	Laurasiatheria	Ruminantia	Bovidae
ENSBTAP00000038149	AQP5	Cow	*Bos taurus*	Laurasiatheria	Ruminantia	Bovidae
ELR55939	AQP5	Yak	*Bos mutus*	Laurasiatheria	Ruminantia	Bovidae
ENSTTRP00000000946	AQP5	Bottlenosed dolphin	*Tursiops truncatus*	Laurasiatheria	Cetacea	Delphinidae
ANOL02036890/XP_004274398	AQP5	Killer whale	*Orcinus orca*	Laurasiatheria	Cetacea	Delphinidae
AUPI01045706	AQP5	Yangtze River dolphin	*Lipotes vexillifer*	Laurasiatheria	Cetacea	Lipotidae
AWZP01022053	AQP5	Sperm whale	*Physeter catodon*	Laurasiatheria	Cetacea	Physeteridae
ATDI01166362	AQP5	Minke whale	*Balaenoptera acutorostrata*	Laurasiatheria	Cetacea	Balaenopteridae
ENSSSCP00000000223	AQP5	Pig	*Sus scrofa*	Laurasiatheria	Suina	Suidae
ABRR02045818	AQP5	Alpaca	*Vicugna pacos*	Laurasiatheria	Tylopoda	Camelidae
AGVR01013769	AQP5	Wild Bactrian camel	*Camelus ferus*	Laurasiatheria	Tylopoda	Camelidae
ENSECAP00000006464	AQP5	Horse	*Equus caballus*	Laurasiatheria	Perissodactyla	Equidae
AKZM01022930	AQP5	Southern white rhinocerus	*Ceratotherium simum simum*	Laurasiatheria	Perissodactyla	Rhinocerotidae
Ame_R010633	AQP5	Giant panda	*Ailuropoda melanoleuca*	Laurasiatheria	Carnivora	Ursidae
ENSCAFP00000012302	AQP5	Dog	*Canis lupus familiaris*	Laurasiatheria	Carnivora	Canidae
ENSMPUP00000014412	AQP5	Domestic ferret	*Mustela putorius furo*	Laurasiatheria	Carnivora	Mustelidae
XP_003988760	AQP5	Domestic cat	*Felis catus*	Laurasiatheria	Carnivora	Felidae
ATCQ01001279	AQP5	Amur tiger	*Panthera tigris altaica*	Laurasiatheria	Carnivora	Felidae
APMU01027970/APMU01027971	AQP5	Weddell seal	*Leptonychotes weddellii*	Laurasiatheria	Carnivora	Phocidae
ANOP01016854	AQP5	Pacific walrus	*Odobenus rosmarus divergens*	Laurasiatheria	Carnivora	Odobenidae
GENSCAN00000015195	AQP5	African savanna elephant	*Loxodonta africana*	Afrotheria	Proboscidea	Elephantidae
AHIN01131586	AQP5	Florida manatee	*Trichechus manatus latirostris*	Afrotheria	Sirenia	Trichechidae
ENSPCAP00000006341	AQP5	Cape rock hyrax	*Procavia capensis*	Afrotheria	Hyracoidea	Procaviidae
ALYB01072796	AQP5	Ardvaark	*Orycteropus afer afer*	Afrotheria	Tubulidentata	Orycteropodidae
AAGV03242312	AQP5	Nine-banded armadillo	*Dasypus novemcinctus*	Xenarthra	Cingulata	Dasypodidae
ABVD01587023	AQP5	Hoffmann's two-fingered sloth	*Choloepus hoffmanni*	Xenarthra	Pilosa	Megalonychidae
ENSMEUP00000004288	AQP5	Tammar wallaby	*Macropus eugenii*	Metatheria	Diprotodontia	Macropodidae
ENSSHAP00000004311	AQP5	Tasmanian devil	*Sarcophilus harrisii*	Metatheria	Dasyuromorphia	Dasyuridae
ENSMODP00000000292	AQP5	Gray short-tailed opossum	*Monodelphis domestica*	Metatheria	Didelphimorphia	Didelphidae
GENSCAN00000062534	AQP5	Platypus	*Ornithorhynchus anatinus*	Prototheria	Monotremata	Ornithorhynchidae
AGAI01046028	AQP5	Budgerigar	*Melopsittacus undulatus*	Aves	Psittaciformes	Psittacidae
AOCU01270242/AOCU01056902	AQP5	Puerto Rican parrot	*Amazona vittata*	Aves	Psittaciformes	Psittacidae
AMXX01067670/AOUJ01012027	AQP5	Scarlet macaw	*Ara macao*	Aves	Psittaciformes	Psittacidae
JV171493	AQP5	Duck-eyed junco	*Junco hyemalis*	Aves	Passeriformes	Fringillidae
AKZB01039356	AQP5	Medium ground finch	*Geospiza fortis*	Aves	Passeriformes	Fringillidae
GENSCAN00000026660	AQP5	Zebra finch	*Taeniopygia guttata*	Aves	Passeriformes	Estrildidae
ANZD01003998	AQP5	Tibetan ground-tit	*Pseudopodoces humilis*	Aves	Passeriformes	Paridae
AGTO01022026	AQP5	Collared flycatcher	*Ficedula albicollis*	Aves	Passeriformes	Muscicapidae
AKMT01046107/AKMT01046108	AQP5	Peregrin falcon	*Falco peregrinus*	Aves	Falconiformes	Falconidae
AKMU01023141	AQP5	Saker falcon	*Falco cherrug*	Aves	Falconiformes	Falconidae
EMC76750	AQP5	Rock pigeon	*Columba livia*	Aves	Columbiformes	Columbidae
ENSAPLP00000015360	AQP5	Mallard	*Anas platyrhynchos*	Aves	Anseriformes	Anatidae
ENSMGAP00000011096	AQP5	Turkey	*Meleagris gallopavo*	Aves	Galliformes	Phasianidae
CAH25504	AQP5	Chicken	*Gallus gallus*	Aves	Galliformes	Phasianidae
AKHW01094570	AQP5	American alligator	*Alligator mississippiensis*	Archosauria	Crocodylia	Alligatoridae
AVPB01022407	AQP5	Chinese alligator	*Alligator sinensis*	Archosauria	Crocodylia	Alligatoridae
AGCU01084247/ENSPSIP00000006298	AQP5	Chinese softshell turtle	*Pelodiscus sinensis*	Sauropsida	Testudines	Trionychidae
APJP01292073	AQP5	Spiny softshell turtle	*Apalone spinifera*	Sauropsida	Testudines	Trionychidae
AJIM01256813/AJIM01069436	AQP5	Green seaturtle	*Chelonia mydas*	Sauropsida	Testudines	Cheloniidae
AHGY01100855	AQP5	Western painted turtle	*Chrysemys picta bellii*	Sauropsida	Testudines	Emydidae
AZIM01000294/ETE71913	AQP5	King cobra	*Ophiophagus hannah*	Lepidosauria	Squamata	Elapidae
JAB54666	AQP5	Eastern coral snake	*Micrurus fulvius*	Lepidosauria	Squamata	Elapidae

GW577974	AQP5	Crossed pit viper	*Bothrops alternatus*	Lepidosauria	Squamata	Viperidae
AFJ49444	AQP5	Eastern diamondback rattlesnake	*Crotalus adamanteus*	Lepidosauria	Squamata	Viperidae
GAAZ01000201	AQP5	Timber rattlesnake	*Crotalus horridus*	Lepidosauria	Squamata	Viperidae
AEQU02005683	AQP5	Burmese python	*Python molurus bivittatus*	Lepidosauria	Squamata	Pythonidae
ENSACAP00000008294/ENSACAP00000018777	AQP5	Green anole	*Anolis carolinensis*	Lepidosauria	Squamata	Iguanidae
AZIM01000294/ETE71917	AQP5L	King cobra	*Ophiophagus hannah*	Lepidosauria	Squamata	Elapidae
AEQU02005676/AEQU02005675	AQP5L	Burmese python	*Python molurus bivittatus*	Lepidosauria	Squamata	Pythonidae
ENSACAP00000016688	AQP5L	Green anole	*Anolis carolinensis*	Lepidosauria	Squamata	Iguanidae
AAC69696	AQP5	Marine toad	*Rhinella marina*	Amphibia	Anura	Hylidae
GAEI01000167	AQP5	Pacific treefrog	*Pseudacris regilla*	Amphibia	Anura	Hylidae
ACM69369	AQP5	Dark-spotted frog	*Pelophylax nigromaculatus*	Amphibia	Anura	Ranidae
ACM51135	AQP5	Asiatic toad	*Bufo gargarizans*	Amphibia	Anura	Bufonidae
AAI70067/AAI70069/BAF02790/ NP_001079331/ NM_001085862	AQP5	African clawed frog	*Xenopus laevis*	Amphibia	Anura	Pipidae
ENSXETP00000043976/AAMC02007649	AQP5	Western clawed frog	*Xenopus (Silurana) tropicalis*	Amphibia	Anura	Pipidae
ENSXETP00000043991/AAMC02007648	AQP5L	Western clawed frog	*Xenopus (Silurana) tropicalis*	Amphibia	Anura	Pipidae
**AQP6**						
ENSP00000320247	AQP6	Human	*Homo sapiens*	Euarchontoglires	Primates	Hominidae
ENSPTRP00000008404	AQP6	Chimpanzee	*Pan troglodytes*	Euarchontoglires	Primates	Hominidae
ENSGGOP00000005779	AQP6	Western lowland gorilla	*Gorilla gorilla gorilla*	Euarchontoglires	Primates	Hominidae
ENSPPYP00000005140	AQP6	Sumatran orangutan	*Pongo abelii*	Euarchontoglires	Primates	Hominidae
ENSNLEP00000021637	AQP6	Northern white-cheeked gibbon	*Nomascus leucogenys*	Euarchontoglires	Primates	Hylobatidae
ENSMMUP00000028555	AQP6	Rhesus macaque	*Macaca mulatta*	Euarchontoglires	Primates	Cercopithecidae
EHH66267	AQP6	Crab-eating macaque	*Macaca fascicularis*	Euarchontoglires	Primates	Cercopithecidae
ENSP00000320247	AQP6	Hamadryas baboon	*Papio hamadryas*	Euarchontoglires	Primates	Cercopithecidae
XP_003906403	AQP6	Olive baboon	*Papio anubis*	Euarchontoglires	Primates	Cercopithecidae
ENSCJAP00000039187	AQP6	White-tufted-ear marmoset	*Callithrix jacchus*	Euarchontoglires	Primates	Cebidae
XP_003939196	AQP6	Bolivian squirrel monkey	*Saimiri boliviensis boliviensis*	Euarchontoglires	Primates	Cebidae
ENSMICP00000014217	AQP6	Gray mouse lemur	*Microcebus murinus*	Euarchontoglires	Primates	Cheirogaleidae
XP_003793672/ENSOGAP00000004158	AQP6	Small-eared galago/Bushbaby	*Otolemur garnettii*	Euarchontoglires	Primates	Galagidae
ENSTBEP00000010947	AQP6	Northern tree shrew	*Tupaia belangeri*	Euarchontoglires	Scandentia	Tupaiidae
ALAR01214973	AQP6	Chinese tree shrew	*Tupaia chinensis*	Euarchontoglires	Scandentia	Tupaiidae
ENSMUSP00000023754	AQP6	Mouse	*Mus musculus*	Euarchontoglires	Rodentia	Muridae
ENSRNOP00000000323	AQP6	Norway rat	*Rattus norvegicus*	Euarchontoglires	Rodentia	Muridae
XP_003507890	AQP6	Chinese hamster	*Cricetulus griseus*	Euarchontoglires	Rodentia	Cricetidae
XP_005353967	AQP6	Prairie vole	*Microtus ochrogaster*	Euarchontoglires	Rodentia	Cricetidae
AKZC01010086/AKZC01010087	AQP6	Lesser Egyptian jerboa	*Jaculus jaculus*	Euarchontoglires	Rodentia	Dipodidae
ENSDORP00000013584	AQP6	Ord's kangaroo rat	*Dipodomys ordii*	Euarchontoglires	Rodentia	Heteromyidae
XP_004637855	AQP6	Degu	*Octodon degus*	Euarchontoglires	Rodentia	Octodontidae
ENSCPOP00000003951	AQP6	Domestic guinea pig	*Cavia porcellus*	Euarchontoglires	Rodentia	Caviidae
EHB00518	AQP6	Naked mole-rat	*Heterocephalus glaber*	Euarchontoglires	Rodentia	Bathyergidae
XP_005411915	AQP6	Long-tailed chinchilla	*Chinchilla lanigera*	Euarchontoglires	Rodentia	Chinchillidae
ALIT01111584/ENSOPRP00000007476	AQP6	American pika	*Ochotona princeps*	Euarchontoglires	Lagomorpha	Ochotonidae
AAGW02058904/ENSOCUP00000022532	AQP6	Rabbit	*Oryctolagus cuniculus*	Euarchontoglires	Lagomorpha	Leporidae
ENSEEUP00000005178	AQP6	Western European hedgehog	*Erinaceus europaeus*	Laurasiatheria	Insectivora	Erinaceinae
AALT02012851	AQP6	European shrew	*Sorex araneus*	Laurasiatheria	Insectivora	Soricidae
AJFV01069454	AQP6	Star-nosed mole	*Condylura cristata*	Laurasiatheria	Insectivora	Talpidae
ENSPVAP00000006322	AQP6	Large flying fox/Megabat	*Pteropus vampyrus*	Laurasiatheria	Chiroptera	Pteropodidae
ALWS01070685	AQP6	Black flying fox	*Pteropus alecto*	Laurasiatheria	Chiroptera	Pteropodidae
ENSMLUP00000019499	AQP6	Little brown bat/Microbat	*Myotis lucifugus*	Laurasiatheria	Chiroptera	Vespertilionidae
ELK36320	AQP6	David´s myotis	*Myotis davidii*	Laurasiatheria	Chiroptera	Vespertilionidae
XP_005877370	AQP6	Brandt's bat	*Myotis brandtii*	Laurasiatheria	Chiroptera	Vespertilionidae
ENSBTAP00000016984	AQP6	Sheep	*Ovis aries*	Laurasiatheria	Ruminantia	Bovidae
AJPT01054264 /XP_005680038	AQP6	Goat	*Capra hircus*	Laurasiatheria	Ruminantia	Bovidae
XP_005981022	AQP6	Chiru	*Pantholops hodgsonii*	Laurasiatheria	Ruminantia	Bovidae
XP_606158/ENSBTAP00000016984	AQP6	Cow	*Bos taurus*	Laurasiatheria	Ruminantia	Bovidae
ELR55940	AQP6	Yak	*Bos mutus*	Laurasiatheria	Ruminantia	Bovidae
ENSTTRP00000000947	AQP6	Bottlenosed dolphin	*Tursiops truncatus*	Laurasiatheria	Cetacea	Delphinidae
ANOL02036890	AQP6	Killer whale	*Orcinus orca*	Laurasiatheria	Cetacea	Delphinidae
AUPI01045706	AQP6	Yangtze River dolphin	*Lipotes vexillifer*	Laurasiatheria	Cetacea	Lipotidae
AWZP01022053	AQP6	Sperm whale	*Physeter catodon*	Laurasiatheria	Cetacea	Physeteridae
ATDI01166362	AQP6	Minke whale	*Balaenoptera acutorostrata*	Laurasiatheria	Cetacea	Balaenopteridae
NP_001121939/ENSSSCP00000000224	AQP6	Pig	*Sus scrofa*	Laurasiatheria	Suina	Suidae
ABRR02045818	AQP6	Alpaca	*Vicugna pacos*	Laurasiatheria	Tylopoda	Camelidae
AGVR01013769	AQP6	Wild Bactrian camel	*Camelus ferus*	Laurasiatheria	Tylopoda	Camelidae
ENSECAP00000007730	AQP6	Horse	*Equus caballus*	Laurasiatheria	Perissodactyla	Equidae
AKZM01022930	AQP6	Southern white rhinocerus	*Ceratotherium simum simum*	Laurasiatheria	Perissodactyla	Rhinocerotidae
ENSAMEP00000001137	AQP6	Giant panda	*Ailuropoda melanoleuca*	Laurasiatheria	Carnivora	Ursidae
ENSCAFP00000012296	AQP6	Dog	*Canis lupus familiaris*	Laurasiatheria	Carnivora	Canidae
ENSMPUP00000014416	AQP6	Domestic ferret	*Mustela putorius furo*	Laurasiatheria	Carnivora	Mustelidae
ENSFCAP00000008772	AQP6	Domestic cat	*Felis catus*	Laurasiatheria	Carnivora	Felidae
ATCQ01001279	AQP6	Amur tiger	*Panthera tigris altaica*	Laurasiatheria	Carnivora	Felidae
APMU01027971	AQP6	Weddell seal	*Leptonychotes weddellii*	Laurasiatheria	Carnivora	Phocidae

ANOP01016854	AQP6	Pacific walrus	*Odobenus rosmarus divergens*	Laurasiatheria	Carnivora	Odobenidae
ENSLAFP00000009645	AQP6	African savanna elephant	*Loxodonta africana*	Afrotheria	Proboscidea	Elephantidae
AHIN01131587	AQP6	Florida manatee	*Trichechus manatus latirostris*	Afrotheria	Sirenia	Trichechidae
ENSPCAP00000006314	AQP6	Cape rock hyrax	*Procavia capensis*	Afrotheria	Hyracoidea	Procaviidae
ALYB01072796	AQP6	Ardvaark	*Orycteropus afer afer*	Afrotheria	Tubulidentata	Orycteropodidae
ALYB01072796/ENSDNOP00000013616	AQP6	Nine-banded armadillo	*Dasypus novemcinctus*	Xenarthra	Cingulata	Dasypodidae
ENSCHOP00000011009	AQP6	Hoffmann's two-fingered sloth	*Choloepus hoffmanni*	Xenarthra	Pilosa	Megalonychidae
ENSMEUP00000009880	AQP6	Tammar wallaby	*Macropus eugenii*	Metatheria	Diprotodontia	Macropodidae
ENSSHAP00000004904	AQP6	Tasmanian devil	*Sarcophilus harrisii*	Metatheria	Dasyuromorphia	Dasyuridae
XP_001510602	AQP6	Platypus	*Ornithorhynchus anatinus*	Prototheria	Monotremata	Ornithorhynchidae
AGCU01140275/ENSPSIP00000012907	AQP6	Chinese softshell turtle	*Pelodiscus sinensis*	Sauropsida	Testudines	Trionychidae
APJP01765402/APJP01493537/APJP01257492	AQP6	Spiny softshell turtle	*Apalone spinifera*	Sauropsida	Testudines	Trionychidae
AJIM01219375	AQP6	Green seaturtle	*Chelonia mydas*	Sauropsida	Testudines	Cheloniidae
AHGY01401458	AQP6	Western painted turtle	*Chrysemys picta bellii*	Sauropsida	Testudines	Emydidae
AZIM01003608/ETE61862	AQP6	King cobra	*Ophiophagus hannah*	Lepidosauria	Squamata	Elapidae
AEQU02005686/AEQU02005685	AQP6	Burmese python	*Python molurus bivittatus*	Lepidosauria	Squamata	Pythonidae
ENSACAP00000016674	AQP6	Green anole	*Anolis carolinensis*	Lepidosauria	Squamata	Iguanidae
BAC82379	AQP6ub	Japanese treefrog	*Hyla japonica*	Amphibia	Anura	Hylidae
AAC69694	AQP6ub	Marine toad	*Rhinella marina*	Amphibia	Anura	Hylidae
NP_001137369	AQP6ub	African clawed frog	*Xenopus laevis*	Amphibia	Anura	Pipidae
ENSXETP00000043995/AAMC02007648/ XP_002935778	AQP6ub	Western clawed frog	*Xenopus (Silurana) tropicalis*	Amphibia	Anura	Pipidae
AAC69695	AQP6vs2	Marine toad	*Rhinella marina*	Amphibia	Anura	Hylidae
BAC07471	AQP6vs2	Japanese treefrog	*Hyla japonica*	Amphibia	Anura	Hylidae
BAI49429	AQP6vs2	Japanese toad	*Bufo japonicus*	Amphibia	Anura	Bufonidae
BAO42828/ACM89453	AQP6vs2	Dark-spotted frog	*Pelophylax nigromaculatus*	Amphibia	Anura	Ranidae
BAO42827	AQP6vs2	Japanese frog	*Rana japonica*	Amphibia	Anura	Ranidae
BAO42829	AQP6vs2	Bullfrog	*Rana catesbeiana*	Amphibia	Anura	Ranidae
BAI49428	AQP6vs1	Dark-spotted frog	*Pelophylax nigromaculatus*	Amphibia	Anura	Ranidae
BAI49427	AQP6vs1	Japanese frog	*Rana japonica*	Amphibia	Anura	Ranidae
BAI49426	AQP6vs1	Bullfrog	*Rana catesbeiana*	Amphibia	Anura	Ranidae
BAI49430/NP_001163923	AQP6vs1	African clawed frog	*Xenopus laevis*	Amphibia	Anura	Pipidae
ENSXETP00000029351/AAMC02007651/ XP_002935790	AQP6vs1	Western clawed frog	*Xenopus (Silurana) tropicalis*	Amphibia	Anura	Pipidae
**AQP7**						
ENSP00000316510/ENST00000322309	AQP7p4	Human	*Homo sapiens*	Euarchontoglires	Primates	Hominidae
ENSP00000329634/ENST00000428759	AQP7p3	Human	*Homo sapiens*	Euarchontoglires	Primates	Hominidae
ENST00000453967	AQP7p2	Human	*Homo sapiens*	Euarchontoglires	Primates	Hominidae
ENSP00000335588	AQP7p1	Human	*Homo sapiens*	Euarchontoglires	Primates	Hominidae
ENSP00000456868	AQP7	Human	*Homo sapiens*	Euarchontoglires	Primates	Hominidae
ENSP00000368821	AQP7	Human	*Homo sapiens*	Euarchontoglires	Primates	Hominidae
ENSPTRP00000035670	AQP7	Chimpanzee	*Pan troglodytes*	Euarchontoglires	Primates	Hominidae
ENSGGOP00000004884	AQP7	Western lowland gorilla	*Gorilla gorilla gorilla*	Euarchontoglires	Primates	Hominidae
ENSPPYP00000021439	AQP7	Sumatran orangutan	*Pongo abelii*	Euarchontoglires	Primates	Hominidae
ENSNLEP00000006164	AQP7	Northern white-cheeked gibbon	*Nomascus leucogenys*	Euarchontoglires	Primates	Hylobatidae
ENSMMUP00000039362	AQP7	Rhesus macaque	*Macaca mulatta*	Euarchontoglires	Primates	Cercopithecidae
ENSMMUP00000039362	AQP7	Crab-eating macaque	*Macaca fascicularis*	Euarchontoglires	Primates	Cercopithecidae
GENSCAN00000041650	AQP7	Hamadryas baboon	*Papio hamadryas*	Euarchontoglires	Primates	Cercopithecidae
AHZZ01080412	AQP7	Olive baboon	*Papio anubis*	Euarchontoglires	Primates	Cercopithecidae
ENSCJAP00000015117	AQP7	White-tufted-ear marmoset	*Callithrix jacchus*	Euarchontoglires	Primates	Cebidae
XP_003939791	AQP7	Bolivian squirrel monkey	*Saimiri boliviensis boliviensis*	Euarchontoglires	Primates	Cebidae
ENSTSYP00000000772	AQP7	Philippine tarsier	*Tarsius syrichta*	Euarchontoglires	Primates	Tarsiidae
ENSMICP00000011819	AQP7	Gray mouse lemur	*Microcebus murinus*	Euarchontoglires	Primates	Cheirogaleidae
ENSOGAP00000000692	AQP7	Small-eared galago/Bushbaby	*Otolemur garnettii*	Euarchontoglires	Primates	Galagidae
ENSTBEP00000005344	AQP7	Northern tree shrew	*Tupaia belangeri*	Euarchontoglires	Scandentia	Tupaiidae
ELW70259	AQP7	Chinese tree shrew	*Tupaia chinensis*	Euarchontoglires	Scandentia	Tupaiidae
ENSMUSP00000030136	AQP7	Mouse	*Mus musculus*	Euarchontoglires	Rodentia	Muridae
ENSRNOP00000012974	AQP7	Norway rat	*Rattus norvegicus*	Euarchontoglires	Rodentia	Muridae
XP_003512015	AQP7	Chinese hamster	*Cricetulus griseus*	Euarchontoglires	Rodentia	Cricetidae
ABRO01459270	AQP7	Ord's kangaroo rat	*Dipodomys ordii*	Euarchontoglires	Rodentia	Heteromyidae
ENSCPOP00000011038	AQP7	Domestic guinea pig	*Cavia porcellus*	Euarchontoglires	Rodentia	Caviidae
EHB02656	AQP7	Naked mole-rat	*Heterocephalus glaber*	Euarchontoglires	Rodentia	Bathyergidae
ENSSTOP00000006294	AQP7	Thirteen-lined ground squirrel	*Ictidomys tridecemlineatus*	Euarchontoglires	Rodentia	Sciuridae
ENSOPRP00000015343	AQP7	American pika	*Ochotona princeps*	Euarchontoglires	Lagomorpha	Ochotonidae
ENSOCUP00000015329	AQP7	Rabbit	*Oryctolagus cuniculus*	Euarchontoglires	Lagomorpha	Leporidae
ENSEEUP00000002340	AQP7	Western European hedgehog	*Erinaceus europaeus*	Laurasiatheria	Insectivora	Erinaceinae
ENSSARP00000008852	AQP7	Common shrew	*Sorex araneus*	Laurasiatheria	Insectivora	Soricidae
ENSPVAP00000010825	AQP7	Large flying fox/Megabat	*Pteropus vampyrus*	Laurasiatheria	Chiroptera	Pteropodidae
ELK09078	AQP7	Black flying fox	*Pteropus alecto*	Laurasiatheria	Chiroptera	Pteropodidae
ELK34288	AQP7	David´s myotis	*Myotis davidii*	Laurasiatheria	Chiroptera	Vespertilionidae
ENSECAP00000011814	AQP7	Sheep	*Ovis aries*	Laurasiatheria	Ruminantia	Bovidae
ENSBTAP00000026782	AQP7	Cow	*Bos taurus*	Laurasiatheria	Ruminantia	Bovidae
ELR51090	AQP7	Yak	*Bos mutus*	Laurasiatheria	Ruminantia	Bovidae
ENSTTRP00000010030	AQP7	Bottlenosed dolphin	*Tursiops truncatus*	Laurasiatheria	Cetacea	Delphinidae

ANOL02039845/XP_004275132	AQP7	Killer whale	*Orcinus orca*	Laurasiatheria	Cetacea	Delphinidae
ENSSSCP00000011725	AQP7	Pig	*Sus scrofa*	Laurasiatheria	Suina	Suidae
ENSVPAP00000001206	AQP7	Alpaca	*Vicugna pacos*	Laurasiatheria	Tylopoda	Camelidae
ENSECAP00000011814	AQP7	Horse	*Equus caballus*	Laurasiatheria	Perissodactyla	Equidae
ENSAMEP00000007857	AQP7	Giant panda	*Ailuropoda melanoleuca*	Laurasiatheria	Carnivora	Ursidae
ENSCAFP00000002708	AQP7	Dog	*Canis lupus familiaris*	Laurasiatheria	Carnivora	Canidae
ENSCAFP00000002706	AQP7	Domestic ferret	*Mustela putorius furo*	Laurasiatheria	Carnivora	Mustelidae
ENSFCAP00000002506	AQP7	Domestic cat	*Felis catus*	Laurasiatheria	Carnivora	Felidae
ENSPCAP00000006546	AQP7	Cape rock hyrax	*Procavia capensis*	Afrotheria	Hyracoidea	Procaviidae
ENSLAFP00000023877	AQP7	African savanna elephant	*Loxodonta africana*	Afrotheria	Proboscidea	Elephantidae
AAIY02212605	AQP7	Lesser hedgehog tenrec	*Echinops telfairi*	Afrotheria	Afrosoricida	Tenrecidae
AHIN01023999	AQP7	Florida manatee	*Trichechus manatus latirostris*	Afrotheria	Sirenia	Trichechidae
ALYB01234333	AQP7	Ardvaark	*Orycteropus afer afer*	Afrotheria	Tubulidentata	Orycteropodidae
ENSDNOP00000010742	AQP7	Nine-banded armadillo	*Dasypus novemcinctus*	Xenarthra	Cingulata	Dasypodidae
ABVD01677639	AQP7	Hoffmann's two-fingered sloth	*Choloepus hoffmanni*	Xenarthra	Pilosa	Megalonychidae
ENSMEUP00000001313	AQP7	Tammar wallaby	*Macropus eugenii*	Metatheria	Diprotodontia	Macropodidae
ENSSHAP00000013186	AQP7	Tasmanian devil	*Sarcophilus harrisii*	Metatheria	Dasyuromorphia	Dasyuridae
ENSMODP00000001460	AQP7	Gray short-tailed opossum	*Monodelphis domestica*	Metatheria	Didelphimorphia	Didelphidae
AAPN01426604	AQP7	Platypus	*Ornithorhynchus anatinus*	Prototheria	Monotremata	Ornithorhynchidae
TGUHOMP00000001711	AQP7	Budgerigar	*Melopsittacus undulatus*	Aves	Psittaciformes	Psittacidae
AMXX01077757/AMXX01105423/AOUJ01157177/AOUJ01157173/AOCU01033654/AOUJ01039524	AQP7	Scarlet macaw	*Ara macao*	Aves	Psittaciformes	Psittacidae
CAVT010029656	AQP7	Common canary	*Serinus canaria*	Aves	Passeriformes	Fringillidae
AKZB01095704	AQP7	Medium ground finch	*Geospiza fortis*	Aves	Passeriformes	Fringillidae
ENSTGUP00000001711	AQP7	Zebra finch	*Taeniopygia guttata*	Aves	Passeriformes	Estrildidae
AGTO01001363	AQP7	Collared flycatcher	*Ficedula albicollis*	Aves	Passeriformes	Muscicapidae
ENSAPLP00000004144	AQP7	Mallard	*Anas platyrhynchos*	Aves	Anseriformes	Anatidae
ENSMGAP00000001223	AQP7	Turkey	*Meleagris gallopavo*	Aves	Galliformes	Phasianidae
ENSGALP00000003857	AQP7	Chicken	*Gallus gallus*	Aves	Galliformes	Phasianidae
AKHW01054970	AQP7	American alligator	*Alligator mississippiensis*	Archosauria	Crocodylia	Alligatoridae
AVPB01053037	AQP7	Chinese alligator	*Alligator sinensis*	Archosauria	Crocodylia	Alligatoridae
ENSPSIP00000017416	AQP7	Chinese softshell turtle	*Pelodiscus sinensis*	Sauropsida	Testudines	Trionychidae
EMP32514	AQP7	Green seaturtle	*Chelonia mydas*	Sauropsida	Testudines	Cheloniidae
AHGY01159091/AHGY01159090/AHGY01159089	AQP7	Western painted turtle	*Chrysemys picta bellii*	Sauropsida	Testudines	Emydidae
AZIM01008092/AZIM01023195/AZIM01004676	AQP7	King cobra	*Ophiophagus hannah*	Lepidosauria	Squamata	Elapidae
AEQU02200793/AEQU02200796/AEQU02069794/AEQU02069792/AEQU02069791	AQP7	Burmese python	*Python molurus bivittatus*	Lepidosauria	Squamata	Pythonidae
ENSACAP00000012359	AQP7-1	Green anole	*Anolis carolinensis*	Lepidosauria	Squamata	Iguanidae
ENSACAP00000009259	AQP7-2	Green anole	*Anolis carolinensis*	Lepidosauria	Squamata	Iguanidae
NP_001015726/ENSXETP00000063272/AAMC02000002/AAMC02000174/AAMC02000175	AQP7	Western clawed frog	*Xenopus (Silurana) tropicalis*	Amphibia	Anura	Pipidae
GAQK01140589/GAQK01041595	AQP7	Chinese salamander	*Hynobius chinensis*	Amphibia	Caudata	Hynobiidae
AFYH01154017/AFYH01154016/AFYH01154015/AFYH01154013/AFYH01154012	Aqp7	Coelacanth	*Latimeria chalumnae*	Actinistia	Coelacanthiformes	Coelacanthidae
GAPS01007864	Aqp7	Menado coelacanth	*Latimeria menadoensis*	Actinistia	Coelacanthiformes	Coelacanthidae
ENSTRUP00000039522	Aqp7	Torafugu	*Takifugu rubripes*	Acanthopterygii	Tetraodontiformes	Tetraodontidae
AOOT01048843	Aqp7	Sansaifugu	*Takifugu flavidus*	Acanthopterygii	Tetraodontiformes	Tetraodontidae
BAL44696	Aqp7	Mefugu	*Takifugu obscurus*	Acanthopterygii	Tetraodontiformes	Tetraodontidae
ENSTNIP00000013592	Aqp7	Green-spotted pufferfish	*Tetraodon nigroviridis*	Acanthopterygii	Tetraodontiformes	Tetraodontidae
GAAQ01015072/GAAQ01003666	Aqp7	Dover sole	*Solea solea*	Acanthopterygii	Pleuronectiformes	Soleidae
AGRG01023824	Aqp7	Tongue sole	*Cynoglossus semilaevis*	Acanthopterygii	Pleuronectiformes	Cynoglossidae
CBN81126	Aqp7	European seabass	*Dicentrarchus labrax*	Acanthopterygii	Perciformes	Moronidae
AGTA02009238	Aqp7	Zebra mbuna	*Maylandia zebra*	Acanthopterygii	Perciformes	Cichlidae
AFNX01015815	Aqp7	Red mwanza	*Pundamilia nyererei*	Acanthopterygii	Perciformes	Cichlidae
AFNY01011849	Aqp7	Lyretail cichlid	*Neolamprologus brichardi*	Acanthopterygii	Perciformes	Cichlidae
AFNZ01006222	Aqp7	Burton's mouthbrooder	*Haplochromis burtoni*	Acanthopterygii	Perciformes	Cichlidae
ENSONIP00000024506	Aqp7	Nile tilapia	*Oreochromis niloticus*	Acanthopterygii	Perciformes	Cichlidae
BADN01039026	Aqp7	Pacific bluefin tuna	*Thunnus orientalis*	Acanthopterygii	Perciformes	Scombridae
GAAQ01003666	Aqp7	Dover sole	*Solea solea*	Acanthopterygii	Pleuronectiformes	Soleidae
GAJJ01041829/GAJJ01041828/GAJJ01041827/AWGY01175109	Aqp7	Sablefish	*Anoplopoma fimbria*	Acanthopterygii	Scorpaeniformes	Anoplopomatidae
AUPQ01000186	Aqp7	Flag rockfish	*Sebastes rubrivinctus*	Acanthopterygii	Scorpaeniformes	Sebastidae
AUPR01045372/AUPR01045371	Aqp7	Tiger rockfish	*Sebastes nigrocinctus*	Acanthopterygii	Scorpaeniformes	Sebastidae
ENSGACP00000011600	Aqp7	Three-spined stickleback	*Gasterosteus aculeatus*	Acanthopterygii	Gasterosteiformes	Gasterosteidae
ENSORLP00000011130	Aqp7	Japanese medaka	*Oryzias latipes*	Acanthopterygii	Beloniformes	Adrianichthyidae
GAIB01101513	Aqp7	Turquoise killifish	*Nothobranchius furzeri*	Acanthopterygii	Cyprinodontiformes	Nothobranchiidae
DN951591	Aqp7	Common mummichog	*Fundulus heteroclitus*	Acanthopterygii	Cyprinodontiformes	Fundulidae
ES380999	Aqp7	Guppy	*Poecilia reticulata*	Acanthopterygii	Cyprinodontiformes	Poeciliidae
AYCK01005653	Aqp7	Amazon molly	*Poecilia formosa*	Acanthopterygii	Cyprinodontiformes	Poeciliidae
ENSXMAP00000018206	Aqp7	Southern platyfish	*Xiphophorus maculatus*	Acanthopterygii	Cyprinodontiformes	Poeciliidae
ENSGMOP00000009366	Aqp7	Atlantic cod	*Gadus morhua*	Paracanthopterygii	Gadiformes	Gadidae
JP753060/JP744328	Aqp7	Ayu	*Plecoglossus altivelis*	Protacanthopterygii	Osmeriformes	Osmeridae
EZ893726/CCAF010014398	Aqp7	Rainbow trout	*Oncorhynchus mykiss*	Protacanthopterygii	Salmoniformes	Salmonidae
AGKD01021376/AGKD01033103/AGKD01065234	Aqp7	Atlantic salmon	*Salmo salar*	Protacanthopterygii	Salmoniformes	Salmonidae
EE396397	Aqp7	Rare gudgeon	*Gobiocypris rarus*	Ostariophysi	Cypriniformes	Cyprinidae
FJ655385/ENSDARP00000037835	Aqp7	Zebrafish	*Danio rerio*	Ostariophysi	Cypriniformes	Cyprinidae

APWO01079311	Aqp7	Mexican tetra	*Astyanax mexicanus*	Ostariophysi	Characiformes	Characidae
ADO28207	Aqp7	Blue catfish	*Ictalurus furcatus*	Ostariophysi	Siluriformes	Ictaluridae
NP_001188011	Aqp7	Channel catfish	*Ictalurus punctatus*	Ostariophysi	Siluriformes	Ictaluridae
AVPY01118075/AVPY01485360/AVPY01278193/ AVPY01542626	Aqp7	Japanese eel	*Anguilla japonica*	Elopomorpha	Anguilliformes	Anguillidae
**AQP8**						
ENSP00000219660	AQP8	Human	*Homo sapiens*	Euarchontoglires	Primates	Hominidae
ENSPTRP00000013506	AQP8	Chimpanzee	*Pan troglodytes*	Euarchontoglires	Primates	Hominidae
ENSGGOP00000010766	AQP8	Western lowland gorilla	*Gorilla gorilla gorilla*	Euarchontoglires	Primates	Hominidae
ENSPPYP00000008147	AQP8	Sumatran orangutan	*Pongo abelii*	Euarchontoglires	Primates	Hominidae
ENSNLEP00000014269	AQP8	Northern white-cheeked gibbon	*Nomascus leucogenys*	Euarchontoglires	Primates	Hylobatidae
ENSMMUP00000013727	AQP8	Rhesus macaque	*Macaca mulatta*	Euarchontoglires	Primates	Cercopithecidae
XP_005591557	AQP8	Crab-eating macaque	*Macaca fascicularis*	Euarchontoglires	Primates	Cercopithecidae
ENSP00000219660	AQP8	Hamadryas baboon	*Papio hamadryas*	Euarchontoglires	Primates	Cercopithecidae
XP_003916747	AQP8	Olive baboon	*Papio anubis*	Euarchontoglires	Primates	Cercopithecidae
ENSCJAP00000038590	AQP8	White-tufted-ear marmoset	*Callithrix jacchus*	Euarchontoglires	Primates	Cebidae
XP_003930264	AQP8	Bolivian squirrel monkey	*Saimiri boliviensis boliviensis*	Euarchontoglires	Primates	Cebidae
ENSTSYP00000008941	AQP8	Philippine tarsier	*Tarsius syrichta*	Euarchontoglires	Primates	Tarsiidae
ENSMICP00000014811	AQP8	Gray mouse lemur	*Microcebus murinus*	Euarchontoglires	Primates	Cheirogaleidae
XP_003795792/XP_003795895/ ENSOGAP00000008315	AQP8	Small-eared galago/Bushbaby	*Otolemur garnettii*	Euarchontoglires	Primates	Galagidae
AGTM013009303/AGTM011747475/AGTM01184942 5/AGTM011887131/AGTM011696191	AQP8	Aye-aye	*Daubentonia madagascariensis*	Euarchontoglires	Primates	Daubentoniidae
AAPY01541514/AAPY01324025/AAPY01324030/ AAPY01720122	AQP8	Northern tree shrew	*Tupaia belangeri*	Euarchontoglires	Scandentia	Tupaiidae
ELW66270/ALAR01051960	AQP8	Chinese tree shrew	*Tupaia chinensis*	Euarchontoglires	Scandentia	Tupaiidae
ENSMUSP00000033023	AQP8	Mouse	*Mus musculus*	Euarchontoglires	Rodentia	Muridae
ENSRNOP00000019939	AQP8	Norway rat	*Rattus norvegicus*	Euarchontoglires	Rodentia	Muridae
Q5I4F8	AQP8	Spinifex hopping mouse	*Notomys alexis*	Euarchontoglires	Rodentia	Muridae
EGW10991	AQP8	Chinese hamster	*Cricetulus griseus*	Euarchontoglires	Rodentia	Cricetidae
XP_005351241	AQP8	Prairie vole	*Microtus ochrogaster*	Euarchontoglires	Rodentia	Cricetidae
AKZC01266636	AQP8	Lesser Egyptian jerboa	*Jaculus jaculus*	Euarchontoglires	Rodentia	Dipodidae
ENSDORP00000001917	AQP8	Ord's kangaroo rat	*Dipodomys ordii*	Euarchontoglires	Rodentia	Heteromyidae
XP_004622956	AQP8	Degu	*Octodon degus*	Euarchontoglires	Rodentia	Octodontidae
AAKN02007697/XP_003478257/ ENSCPOP00000004692	AQP8	Domestic guinea pig	*Cavia porcellus*	Euarchontoglires	Rodentia	Caviidae
AFSB01095888/EHB05804	AQP8	Naked mole-rat	*Heterocephalus glaber*	Euarchontoglires	Rodentia	Bathyergidae
ENSSTOP00000000373	AQP8	Thirteen-lined ground squirrel	*Ictidomys tridecemlineatus*	Euarchontoglires	Rodentia	Sciuridae
XP_005405186	AQP8	Long-tailed chinchilla	*Chinchilla lanigera*	Euarchontoglires	Rodentia	Chinchillidae
ALIT01053989	AQP8	American pika	*Ochotona princeps*	Euarchontoglires	Lagomorpha	Ochotonidae
ENSOCUP00000001602	AQP8	Rabbit	*Oryctolagus cuniculus*	Euarchontoglires	Lagomorpha	Leporidae
ENSEEUP00000008056	AQP8	Western European hedgehog	*Erinaceus europaeus*	Laurasiatheria	Insectivora	Erinaceinae
AALT02037545/ENSSART00000003839	AQP8	Common shrew	*Sorex araneus*	Laurasiatheria	Insectivora	Soricidae
AJFV01066659	AQP8	Star-nosed mole	*Condylura cristata*	Laurasiatheria	Insectivora	Talpidae
ENSPVAP00000007671	AQP8	Large flying fox/Megabat	*Pteropus vampyrus*	Laurasiatheria	Chiroptera	Pteropodidae
ALWS01045775/ELK12083	AQP8	Black flying fox	*Pteropus alecto*	Laurasiatheria	Chiroptera	Pteropodidae
AWHC01220492	AQP8	Straw-colored fruit bat	*Eidolon helvum*	Laurasiatheria	Chiroptera	Pteropodidae
XP_005862098	AQP8	Brandt's bat	*Myotis brandtii*	Laurasiatheria	Chiroptera	Vespertilionidae
ALEH01100443	AQP8	Big brown bat	*Eptesicus fuscus*	Laurasiatheria	Chiroptera	Vespertilionidae
AWHA01101990	AQP8	Greater horseshoe bat	*Rhinolophus ferrumequinum*	Laurasiatheria	Chiroptera	Rhinolophinae
XP_004020900	AQP8	Sheep	*Ovis aries*	Laurasiatheria	Ruminantia	Bovidae
AJPT01212597/AJPT01212598/AJPT01212599	AQP8	Goat	*Capra hircus*	Laurasiatheria	Ruminantia	Bovidae
XP_005975349/XP_005975350	AQP8	Chiru	*Pantholops hodgsonii*	Laurasiatheria	Ruminantia	Bovidae
NP_001193536/ENSBTAP00000026884	AQP8	Cow	*Bos taurus*	Laurasiatheria	Ruminantia	Bovidae
AGSK01021748/ELR61004	AQP8	Yak	*Bos mutus*	Laurasiatheria	Ruminantia	Bovidae
AGFL01188112	AQP8	Zebu	*Bos indicus*	Laurasiatheria	Ruminantia	Bovidae
ENSTTRP00000014466	AQP8	Bottlenosed dolphin	*Tursiops truncatus*	Laurasiatheria	Cetacea	Delphinidae
XP_004268643	AQP8	Killer whale	*Orcinus orca*	Laurasiatheria	Cetacea	Delphinidae
ENSSSCP00000008353	AQP8	Pig	*Sus scrofa*	Laurasiatheria	Suina	Suidae
ABRR02036842	AQP8	Alpaca	*Vicugna pacos*	Laurasiatheria	Tylopoda	Camelidae
AGVR01068084	AQP8	Wild Bactrian camel	*Camelus ferus*	Laurasiatheria	Tylopoda	Camelidae
AAWR02036274/ENSECAP00000007437	AQP8	Horse	*Equus caballus*	Laurasiatheria	Perissodactyla	Equidae
AKZM01042839	AQP8	Southern white rhinocerus	*Ceratotherium simum simum*	Laurasiatheria	Perissodactyla	Rhinocerotidae
ACTA01052011/ENSAMEP00000007344	AQP8	Giant panda	*Ailuropoda melanoleuca*	Laurasiatheria	Carnivora	Ursidae
GAJD01006926_1_pBear_AQP8	AQP8	Polar bear	*Ursus maritimus*	Laurasiatheria	Carnivora	Ursidae
ENSCAFP00000025764	AQP8	Dog	*Canis lupus familiaris*	Laurasiatheria	Carnivora	Canidae
AEYP01106350/ENSMPUP00000011317	AQP8	Domestic ferret	*Mustela putorius furo*	Laurasiatheria	Carnivora	Mustelidae
ENSFCAP00000014163	AQP8	Domestic cat	*Felis catus*	Laurasiatheria	Carnivora	Felidae
ATCQ01065188	AQP8	Amur tiger	*Panthera tigris altaica*	Laurasiatheria	Carnivora	Felidae
APMU01152163/APMU01072733	AQP8	Weddell seal	*Leptonychotes weddellii*	Laurasiatheria	Carnivora	Phocidae
ANOP01061805	AQP8	Pacific walrus	*Odobenus rosmarus divergens*	Laurasiatheria	Carnivora	Odobenidae
ENSLAFP00000001748	AQP8	African savanna elephant	*Loxodonta africana*	Afrotheria	Proboscidea	Elephantidae
AHIN01033319/XP_004373520	AQP8	Florida manatee	*Trichechus manatus latirostris*	Afrotheria	Sirenia	Trichechidae
ENSPCAP00000013157	AQP8	Cape rock hyrax	*Procavia capensis*	Afrotheria	Hyracoidea	Procaviidae
ENSETEP00000010987	AQP8	Lesser hedgehog tenrec	*Echinops telfairi*	Afrotheria	Afrosoricida	Tenrecidae

AMGZ01202933	AQP8	Cape elephant shrew	*Elephantulus edwardii*	Afrotheria	Macroscelidea	Macroscelididae
ALYB01288452	AQP8	Ardvaark	*Orycteropus afer afer*	Afrotheria	Tubulidentata	Orycteropodidae
AAGV03222964/AAGV03222963/ ENSDNOP00000012347	AQP8	Nine-banded armadillo	*Dasypus novemcinctus*	Xenarthra	Cingulata	Dasypodidae
ENSMEUP00000008589	AQP8	Tammar wallaby	*Macropus eugenii*	Metatheria	Diprotodontia	Macropodidae
ENSSHAP00000004658	AQP8	Tasmanian devil	*Sarcophilus harrisii*	Metatheria	Dasyuromorphia	Dasyuridae
ENSMODP00000020379	AQP8	Gray short-tailed opossum	*Monodelphis domestica*	Metatheria	Didelphimorphia	Didelphidae
ENSOANP00000002345	AQP8	Platypus	*Ornithorhynchus anatinus*	Prototheria	Monotremata	Ornithorhynchidae
AGAI01045362/AGAI01045363	AQP8	Budgerigar	*Melopsittacus undulatus*	Aves	Psittaciformes	Psittacidae
AOCU01047458	AQP8	Puerto Rican parrot	*Amazona vittata*	Aves	Psittaciformes	Psittacidae
AMXX01078163/AOUJ01150819	AQP8	Scarlet macaw	*Ara macao*	Aves	Psittaciformes	Psittacidae
CAVT010020799/CAVT010020798/ CAVT010020797	AQP8	Common canary	*Serinus canaria*	Aves	Passeriformes	Fringillidae
AKZB01020340/AKZB01020342	AQP8	Medium ground finch	*Geospiza fortis*	Aves	Passeriformes	Fringillidae
ENSTGUP00000006407	AQP8	Zebra finch	*Taeniopygia guttata*	Aves	Passeriformes	Estrildidae
ANZD01016667	AQP8	Tibetan ground-tit	*Pseudopodoces humilis*	Aves	Passeriformes	Paridae
AGTO01020840	AQP8	Collared flycatcher	*Ficedula albicollis*	Aves	Passeriformes	Muscicapidae
AKMT01081813	AQP8	Peregrin falcon	*Falco peregrinus*	Aves	Falconiformes	Falconidae
AKMU01036286	AQP8	Saker falcon	*Falco cherrug*	Aves	Falconiformes	Falconidae
AKCR01035325	AQP8	Rock pigeon	*Columba livia*	Aves	Columbiformes	Columbidae
ENSAPLP00000003161	AQP8	Mallard	*Anas platyrhynchos*	Aves	Anseriformes	Anatidae
GW701433	AQP8	Red grouse	*Lagopus lagopus scotica*	Aves	Galliformes	Phasianidae
ENSMGAP00000006410	AQP8	Turkey	*Meleagris gallopavo*	Aves	Galliformes	Phasianidae
ENSGALP00000009615	AQP8	Chicken	*Gallus gallus*	Aves	Galliformes	Phasianidae
AKHW01055511	AQP8	American alligator	*Alligator mississippiensis*	Archosauria	Crocodylia	Alligatoridae
AVPB01033562	AQP8	Chinese alligator	*Alligator sinensis*	Archosauria	Crocodylia	Alligatoridae
ENSPSIP00000005434/AGCU01157136/ AGCU01157135	AQP8	Chinese softshell turtle	*Pelodiscus sinensis*	Sauropsida	Testudines	Trionychidae
APJP01614078	AQP8	Spiny softshell turtle	*Apalone spinifera*	Sauropsida	Testudines	Trionychidae
AJIM01178016	AQP8	Green seaturtle	*Chelonia mydas*	Sauropsida	Testudines	Cheloniidae
JW367444	AQP8	Red-eared slider turtle	*Trachemys scripta elegans*	Sauropsida	Testudines	Emydidae
AHGY01056199/AHGY01056195/AHGY01056194/ JH584542	AQP8	Western painted turtle	*Chrysemys picta bellii*	Sauropsida	Testudines	Emydidae
ETE69496/AZIM01000751	AQP8	King cobra	*Ophiophagus hannah*	Lepidosauria	Squamata	Elapidae
AEQU02159372/AEQU02159371	AQP8	Burmese python	*Python molurus bivittatus*	Lepidosauria	Squamata	Pythonidae
GADO01011857	AQP8	Green anole	*Anolis carolinensis*	Lepidosauria	Squamata	Iguanidae
GAEG01013304	AQP8	Green frog	*Rana clamitans*	Amphibia	Anura	Ranidae
ENSXETP00000064129/NP_001107728	AQP8	Western clawed frog	*Xenopus (Silurana) tropicalis*	Amphibia	Anura	Pipidae
GAQK01005474	AQP8	Chinese salamander	*Hynobius chinensis*	Amphibia	Caudata	Hynobiidae
AFYH01059075/AFYH01059076/BAHO01108925/ ENSLACP00000014505	Aqp8	Coelacanth	*Latimeria chalumnae*	Actinistia	Coelacanthiformes	Coelacanthidae
AGRG01023563	Aqp8aa	Tongue sole	*Cynoglossus semilaevis*	Acanthopterygii	Pleuronectiformes	Cynoglossidae
ENSONIP00000024898	Aqp8aa	Nile tilapia	*Oreochromis niloticus*	Acanthopterygii	Perciformes	Cichlidae
AFNZ01030705	Aqp8aa	Burton's mouthbrooder	*Haplochromis burtoni*	Acanthopterygii	Perciformes	Cichlidae
AGTA02034040	Aqp8aa	Zebra mbuna	*Maylandia zebra*	Acanthopterygii	Perciformes	Cichlidae
AFNX01002912	Aqp8aa	Red mwanza	*Pundamilia nyererei*	Acanthopterygii	Perciformes	Cichlidae
AFNY01058702/AFNY01058703	Aqp8aa	Lyretail cichlid	*Neolamprologus brichardi*	Acanthopterygii	Perciformes	Cichlidae
BADN01115757	Aqp8aa	Pacific bluefin tuna	*Thunnus orientalis*	Acanthopterygii	Perciformes	Scombridae
AWGY01112802/AWGY01112803	Aqp8aa	Sablefish	*Anoplopoma fimbria*	Acanthopterygii	Scorpaeniformes	Anoplopomatidae
AUPQ01060689	Aqp8aa	Flag rockfish	*Sebastes rubrivinctus*	Acanthopterygii	Scorpaeniformes	Sebastidae
AUPR01111278	Aqp8aa	Tiger rockfish	*Sebastes nigrocinctus*	Acanthopterygii	Scorpaeniformes	Sebastidae
AANH01011088/ENSGACP00000012041	Aqp8aa	Three-spined stickleback	*Gasterosteus aculeatus*	Acanthopterygii	Gasterosteiformes	Gasterosteidae
CAEA01026730/ENSGMOP00000001261	Aqp8aa	Atlantic cod	*Gadus morhua*	Paracanthopterygii	Gadiformes	Gadidae
EL539051	Aqp8aa	Rainbow smelt	*Osmerus mordax*	Protacanthopterygii	Osmeriformes	Osmeridae
CU071487/CCAF010046511/CCAF010046510	Aqp8aa1	Rainbow trout	*Oncorhynchus mykiss*	Protacanthopterygii	Salmoniformes	Salmonidae
AGKD01005999/KC626878	Aqp8aa1	Atlantic salmon	*Salmo salar*	Protacanthopterygii	Salmoniformes	Salmonidae
CCAF010129175	Aqp8aa2	Rainbow trout	*Oncorhynchus mykiss*	Protacanthopterygii	Salmoniformes	Salmonidae
AGKD01093852/AGKD01051269/DW573347	Aqp8aa2	Atlantic salmon	*Salmo salar*	Protacanthopterygii	Salmoniformes	Salmonidae
EV367413	Aqp8aa	Lake whitefish	*Coregonus clupeaformis*	Protacanthopterygii	Salmoniformes	Salmonidae
DT104145	Aqp8aa	Fathead minnow	*Pimephales promelas*	Ostariophysi	Cypriniformes	Cyprinidae
GAHO01046818/GAHO01072248/GAHO01153843	Aqp8aa	Small gill opening goldenline barbel	*Sinocyclocheilus angustiporus*	Ostariophysi	Cypriniformes	Cyprinidae
FJ655386/ENSDARP00000066381	Aqp8aa	Zebrafish	*Danio rerio*	Ostariophysi	Cypriniformes	Cyprinidae
GAAD01003877/BJ834836/BJ823625	Aqp8aa	Oriental weatherfish	*Misgurnus anguillicaudatus*	Ostariophysi	Cypriniformes	Cobitidae
APWO01092527/APWO01092528/FO215588	Aqp8aa	Mexican tetra	*Astyanax mexicanus*	Ostariophysi	Characiformes	Characidae
CK402738	Aqp8aa	Blue catfish	*Ictalurus furcatus*	Ostariophysi	Siluriformes	Ictaluridae
GH669824	Aqp8aa	Channel catfish	*Ictalurus punctatus*	Ostariophysi	Siluriformes	Ictaluridae
GAGX01027716/GAGX01214961/GAGX01300098/ GAGX01299389	Aqp8aa	Brown bullhead	*Ameiurus nebulosus*	Ostariophysi	Siluriformes	Ictaluridae
BAH89254/AVPY01052199/AVPY01052198	Aqp8aa	Japanese eel	*Anguilla japonica*	Elopomorpha	Anguilliformes	Anguillidae
AVPY01204069/AVPY01204068	Aqp8-like	Japanese eel	*Anguilla japonica*	Elopomorpha	Anguilliformes	Anguillidae
ENSONIP00000024897	Aqp8ab	Nile tilapia	*Oreochromis niloticus*	Acanthopterygii	Perciformes	Cichlidae
AFNZ01030707	Aqp8ab	Burton's mouthbrooder	*Haplochromis burtoni*	Acanthopterygii	Perciformes	Cichlidae
AGTA02034040	Aqp8ab	Zebra mbuna	*Maylandia zebra*	Acanthopterygii	Perciformes	Cichlidae
AFNX01002912	Aqp8ab	Red mwanza	*Pundamilia nyererei*	Acanthopterygii	Perciformes	Cichlidae
AFNY01058702	Aqp8ab	Lyretail cichlid	*Neolamprologus brichardi*	Acanthopterygii	Perciformes	Cichlidae
JG739786	Aqp8ab	Barramundi perch	*Lates calcarifer*	Acanthopterygii	Perciformes	Latidae

BADN01122461/BADN01122460	Aqp 8ab	Pacific bluefin tuna	*Thunnus orientalis*	Acanthopterygii	Perciformes	Scombridae
ACQ57933/ AWGY01152486	Aqp 8ab	Sablefish	*Anoplopoma fimbria*	Acanthopterygii	Scorpaeniformes	Anoplopomatidae
AUPQ01106288	Aqp 8ab	Flag rockfish	*Sebastes rubrivinctus*	Acanthopterygii	Scorpaeniformes	Sebastidae
AUPR01063288	Aqp 8ab	Tiger rockfish	*Sebastes nigrocinctus*	Acanthopterygii	Scorpaeniformes	Sebastidae
AANH01012285/ENSGACP00000019141	Aqp 8ab	Three-spined stickleback	*Gasterosteus aculeatus*	Acanthopterygii	Gasterosteiformes	Gasterosteidae
ENSORLP00000003812	Aqp 8ab	Japanese medaka	*Oryzias latipes*	Acanthopterygii	Beloniformes	Adrianichthyidae
GAIB01093161/JZ223633	Aqp 8ab	Turquoise killifish	*Nothobranchius furzeri*	Acanthopterygii	Cyprinodontiformes	Nothobranchiidae
AYCK01026450	Aqp 8ab	Amazon molly	*Poecilia formosa*	Acanthopterygii	Cyprinodontiformes	Poeciliidae
AGAJ01040674/ENSXMAP00000017455	Aqp 8ab	Southern platyfish	*Xiphophorus maculatus*	Acanthopterygii	Cyprinodontiformes	Poeciliidae
CAEA01026729/CAEA01201958/EX741282	Aqp 8ab	Atlantic cod	*Gadus morhua*	Paracanthopterygii	Gadiformes	Gadidae
CU071568/CCAF010046510	Aqp 8ab1	Rainbow trout	*Oncorhynchus mykiss*	Protacanthopterygii	Salmoniformes	Salmonidae
AGKD01005999/DW532465	Aqp 8ab1	Atlantic salmon	*Salmo salar*	Protacanthopterygii	Salmoniformes	Salmonidae
CCAF010129175	Aqp 8ab2	Rainbow trout	*Oncorhynchus mykiss*	Protacanthopterygii	Salmoniformes	Salmonidae
AGKD01051269/KC626879	Aqp 8ab2	Atlantic salmon	*Salmo salar*	Protacanthopterygii	Salmoniformes	Salmonidae
DT342244	Aqp 8ab	Fathead minnow	*Pimephales promelas*	Ostariophysi	Cypriniformes	Cyprinidae
EU341834/ENSDARP00000096730	Aqp 8ab	Zebrafish	*Danio rerio*	Ostariophysi	Cypriniformes	Cyprinidae
APWO01092528/FO263691	Aqp 8ab	Mexican tetra	*Astyanax mexicanus*	Ostariophysi	Characiformes	Characidae
HP447158/ADO28238	Aqp 8ab	Blue catfish	*Ictalurus furcatus*	Ostariophysi	Siluriformes	Ictaluridae
JT194091/CV988323	Aqp 8ab	Channel catfish	*Ictalurus punctatus*	Ostariophysi	Siluriformes	Ictaluridae
GAGX01045334	Aqp 8ab	Brown bullhead	*Ameiurus nebulosus*	Ostariophysi	Siluriformes	Ictaluridae
JK480416	Aqp 8ab	Pacific herring	*Clupea pallasii*	Clupeomorpha	Clupeiformes	Clupeidae
AVPY01052198/ AVPY01052197	Aqp 8ab	Japanese eel	*Anguilla japonica*	Elopomorpha	Anguilliformes	Anguillidae
CAEA01278840/CAEA01532611/ ENSGMOP00000012111	Aqp 8ba	Atlantic cod	*Gadus morhua*	Paracanthopterygii	Gadiformes	Gadidae
AGKD01042454	Aqp 8ba1	Atlantic salmon	*Salmo salar*	Protacanthopterygii	Salmoniformes	Salmonidae
AGKD01119048/AGKD01190352	Aqp 8ba2	Atlantic salmon	*Salmo salar*	Protacanthopterygii	Salmoniformes	Salmonidae
APWO01049839/ APWO01049837	Aqp 8ba	Mexican tetra	*Astyanax mexicanus*	Ostariophysi	Characiformes	Characidae
JT413011	Aqp 8ba	Channel catfish	*Ictalurus punctatus*	Ostariophysi	Siluriformes	Ictaluridae
GAGX01179772	Aqp 8ba	Brown bullhead	*Ameiurus nebulosus*	Ostariophysi	Siluriformes	Ictaluridae
AVPY01197226/ AVPY01197230	Aqp 8ba	Japanese eel	*Anguilla japonica*	Elopomorpha	Anguilliformes	Anguillidae
CAAB02006724/ENSTRUP00000007329	Aqp 8bb	Torafugu	*Takifugu rubripes*	Acanthopterygii	Tetraodontiformes	Tetraodontidae
AOOT01031522	Aqp 8bb	Sansaifugu	*Takifugu flavidus*	Acanthopterygii	Tetraodontiformes	Tetraodontidae
BAL44700	Aqp 8bb	Mefugu	*Takifugu obscurus*	Acanthopterygii	Tetraodontiformes	Tetraodontidae
CAG11438/ENSTNIP00000021161	Aqp 8bb	Green-spotted pufferfish	*Tetraodon nigroviridis*	Acanthopterygii	Tetraodontiformes	Tetraodontidae
EB038315	Aqp 8bb	Atlantic halibut	*Hippoglossus hippoglossus*	Acanthopterygii	Pleuronectiformes	Pleuronectidae
DV567193	Aqp 8bb	European flounder	*Platichthys flesus*	Acanthopterygii	Pleuronectiformes	Pleuronectidae
JU402645/FE950965	Aqp 8bb	Turbot	*Scophthalmus maximus*	Acanthopterygii	Pleuronectiformes	Scophthalmidae
GAAQ01009944	Aqp 8bb	Dover sole	*Solea solea*	Acanthopterygii	Pleuronectiformes	Soleidae
AGRG01016566	Aqp 8bb	Tongue sole	*Cynoglossus semilaevis*	Acanthopterygii	Pleuronectiformes	Cynoglossidae
HS988011/DQ889225/ABK20159	Aqp 8bb	Gilthead seabream	*Sparus aurata*	Acanthopterygii	Perciformes	Sparidae
CABK01022538	Aqp 8bb	European seabass	*Dicentrarchus labrax*	Acanthopterygii	Perciformes	Moronidae
BADN01013948	Aqp 8bb	Pacific bluefin tuna	*Thunnus orientalis*	Acanthopterygii	Perciformes	Scombridae
GAJJ01001641/GO620173/ AWGY01064472	Aqp 8bb	Sablefish	*Anoplopoma fimbria*	Acanthopterygii	Scorpaeniformes	Anoplopomatidae
GE808463	Aqp 8bb	Copper rockfish	*Sebastes caurinus*	Acanthopterygii	Scorpaeniformes	Sebastidae
AUPQ01004045	Aqp 8bb	Flag rockfish	*Sebastes rubrivinctus*	Acanthopterygii	Scorpaeniformes	Sebastidae
AUPR01079840	Aqp 8bb	Tiger rockfish	*Sebastes nigrocinctus*	Acanthopterygii	Scorpaeniformes	Sebastidae
AANH01004389/ENSGACP00000015550	Aqp 8bb	Three-spined stickleback	*Gasterosteus aculeatus*	Acanthopterygii	Gasterosteiformes	Gasterosteidae
AFQ36924	Aqp 8bb	Sockeye salmon	*Oncorhynchus nerka*	Protacanthopterygii	Salmoniformes	Salmonidae
AFQ36926	Aqp 8bb1	Chum salmon	*Oncorhynchus keta*	Protacanthopterygii	Salmoniformes	Salmonidae
AGKD01119048/AGKD01156665/KC626880/ ACN11279	Aqp 8bb1	Atlantic salmon	*Salmo salar*	Protacanthopterygii	Salmoniformes	Salmonidae
CCAF010129175	Aqp 8bb2	Rainbow trout	*Oncorhynchus mykiss*	Protacanthopterygii	Salmoniformes	Salmonidae
AGKD01042454	Aqp 8bb2	Atlantic salmon	*Salmo salar*	Protacanthopterygii	Salmoniformes	Salmonidae
FJ695516/ENSDARP00000005510	Aqp 8bb-1	Zebrafish	*Danio rerio*	Ostariophysi	Cypriniformes	Cyprinidae
ENSDARP00000109513	Aqp 8bb-2	Zebrafish	*Danio rerio*	Ostariophysi	Cypriniformes	Cyprinidae
AVPY01383552/ AVPY01383554/ AVPY01383555	Aqp 8bb	Japanese eel	*Anguilla japonica*	Elopomorpha	Anguilliformes	Anguillidae
AHAT01013515	Aqp 8aa	Spotted gar	*Lepisosteus oculatus*	Holostei	Semionotiformes	Lepisosteidae
AHAT01013516	Aqp 8ab	Spotted gar	*Lepisosteus oculatus*	Holostei	Semionotiformes	Lepisosteidae
AEFG01050375/ENSPMAP00000006709	Aqp 8	Sea lamprey	*Petromyzon marinus*	Hyperoartia	Petromyzontiformes	Petromyzontidae
APJL01042678/APJL01112078	Aqp 8	Arctic lamprey	*Lethenteron camtschaticum*	Hyperoartia	Petromyzontiformes	Petromyzontidae
XP_002131566/ENSCINP00000011466	Aqp 8	Vase tunicate	*Ciona intestinalis*	Tunicata	Enterogona	Cionidae
ENSCSAVP00000018099	Aqp 8	Pacific transparent sea squirt	*Ciona savignyi*	Tunicata	Enterogona	Cionidae
XP_002599198	Aqp 8L1	Florida lancelet	*Branchiostoma floridae*	Cephalochordata	Amphioxiformes	Branchiostomidae
XP_002599199	Aqp 8L2	Florida lancelet	*Branchiostoma floridae*	Cephalochordata	Amphioxiformes	Branchiostomidae
XP_002599196	Aqp 8L3	Florida lancelet	*Branchiostoma floridae*	Cephalochordata	Amphioxiformes	Branchiostomidae
XP_002599197	Aqp 8L4	Florida lancelet	*Branchiostoma floridae*	Cephalochordata	Amphioxiformes	Branchiostomidae
XP_002599200_Amx_Aqp8L5	Aqp 8L5	Florida lancelet	*Branchiostoma floridae*	Cephalochordata	Amphioxiformes	Branchiostomidae
FF484301/ACQM01132929	Aqp 8L	Acorn worm	*Saccoglossus kowalevskii*	Hemichordata	Enteropneusta	Harrimaniidae
AM580308/AM595475	Aqp 8L1	Common sea urchin	*Paracentrotus lividus*	Echinodermata	Echinozoa	Echinidae
AAGJ04074400/XP_792735/XP_001197182/ XP_003730891	Aqp 8L1b	Purple sea urchin	*Strongylocentrotus purpuratus*	Echinodermata	Echinozoa	Strongylocentrotida
AAGJ04122156/XP_001198238/SPU_027252-tr	Aqp 8L1	Purple sea urchin	*Strongylocentrotus purpuratus*	Echinodermata	Echinozoa	Strongylocentrotida
GAPB01060243	Aqp 8L1	Kina	*Evechinus chloroticus*	Echinodermata	Echinozoa	Echinometridae
AGCV01110912	Aqp 8L1	Green sea urchin	*Lytechinus variegatus*	Echinodermata	Echinozoa	Toxopneustidae
JI310018	Aqp 8L1	Slate pencil urchin	*Eucidaris tribuloides*	Echinodermata	Echinozoa	Cidaridae

AKZP01049432/AKZP01081921	Aqp8L1	Bat star	*Patiria miniata*	Echinodermata	Asterozoa	Asterinidae
AM563189	Aqp8L2	Common sea urchin	*Paracentrotus lividus*	Echinodermata	Echinozoa	Echinidae
AAGJ04086390/XP_794577/SPU_012095-tr	Aqp8L2	Purple sea urchin	*Strongylocentrotus purpuratus*	Echinodermata	Echinozoa	Strongylocentrotida
GAPB01011060	Aqp8L2	Kina	*Evechinus chloroticus*	Echinodermata	Echinozoa	Echinometridae
AGCV01266172	Aqp8L2	Green sea urchin	*Lytechinus variegatus*	Echinodermata	Echinozoa	Toxopneustidae
JI302630	Aqp8L2	Slate pencil urchin	*Eucidaris tribuloides*	Echinodermata	Echinozoa	Cidaridae
AKZP01072746/AKZP01109668	Aqp8L2	Bat star	*Patiria miniata*	Echinodermata	Asterozoa	Asterinidae
**Protostomia Aqp8L orthologs**						
AEP14560	Aqp8L(6)	Water bear	*Milnesium tardigradum*	Tardigrada	Apochela	Milnesiidae
EKC40660	Aqp8L1	Pacific oyster	*Crassostrea gigas*	Mollusca	Ostreoida	Ostreidae
EKC40659	Aqp8L2	Pacific oyster	*Crassostrea gigas*	Mollusca	Ostreoida	Ostreidae
EKC40657	Aqp8L3	Pacific oyster	*Crassostrea gigas*	Mollusca	Ostreoida	Ostreidae
EKC40658	Aqp8L4	Pacific oyster	*Crassostrea gigas*	Mollusca	Ostreoida	Ostreidae
LotgiP156476	Aqp8L1	Owl limpet	*Lottia gigantea*	Mollusca		Lottiidae
LotgiP196256	Aqp8L2	Owl limpet	*Lottia gigantea*	Mollusca		Lottiidae
FX193841	Aqp8L	Great pond snail	*Lymnaea stagnalis*	Mollusca		Lymnaeidae
XP_005094214	Aqp8L1	California sea hare	*Aplysia californica*	Mollusca		Aplysiidae
XP_005098818	Aqp8L2	California sea hare	*Aplysia californica*	Mollusca		Aplysiidae
XP_005109261	Aqp8L3	California sea hare	*Aplysia californica*	Mollusca		Aplysiidae
C32C4.2	Aqp8L(6)	Free-living roundworm	*Caenorhabditis elegans*	Nematoda	Rhabditida	Rhabditidae
F40F9.9	Aqp8L(4)	Free-living roundworm	*Caenorhabditis elegans*	Nematoda	Rhabditida	Rhabditidae
PPA19447	Aqp8L(6)	Diplogastrid nematode	*Pristionchus pacificus*	Nematoda	Diplogasterida	Neodiplogasteridae
CapteP142373	Aqp8L	Segmented worm	*Capitella teleta*	Annelida	Capitellida	Capitellidae
CapteP166556	Aqp8L	Segmented worm	*Capitella teleta*	Annelida	Capitellida	Capitellidae
CapteP219373	Aqp8L	Segmented worm	*Capitella teleta*	Annelida	Capitellida	Capitellidae
**Porifera Aqp8L orthologs**						
EC374967	Aqp8L	Slime sponge	*Oscarella carmela*	Porifera	Homosclerophorida	Plakinidae
CBY89223	Aqp8L	Siliceous sponge	*Suberites domuncula*	Porifera	Hadromerida	Suberitidae
**Metazoa-Plant homologs**						
GAFI01010612	PIPL	Southern pine beetle	*Dendroctonus frontalis*	Hexapoda	Coleoptera	Curculionidae
JR485718	PIPL	Red palm weevil	*Rhynchophorus ferrugineus*	Hexapoda	Coleoptera	Curculionidae
JG687602	PIPL	Desert locust	*Schistocerca gregaria*	Hexapoda	Orthoptera	Acrididae
GADG01008520	PIPL	Sea scallop	*Placopecten magellanicus*	Mollusca	Pectinoida	Pectinidae
JU024224	PIPL	Portugese oyster	*Crassostrea angulata*	Mollusca	Ostreoida	Ostreidae
EY298090	PIPL	Freshwater leech	*Helobdella robusta*	Annelida	Rhynchobdellida	Glossiphoniidae
FP978959	PIPL	Thecate hydroid	*Clytia hemisphaerica*	Cnidaria	Hydroida	Campanulariidae
AEQ29857	PIP2	Plum-leaf crabapple	*Malus prunifolia*	Streptophyta	Rosales	Rosaceae
AEY75243	PIP	Tea crabapple	*Malus hupehensis*	Streptophyta	Rosales	Rosaceae
GALR01005288	PIP	Asian pear	*Pyrus pyrifolia*	Streptophyta	Rosales	Rosaceae
AAG23179	PIP	Cabbage	*Brassica oleracea*	Streptophyta	Brassicales	Brassicaceae
EZ193234	PIP	Sweet wormwood	*Artemisia annua*	Streptophyta	Asterales	Asteraceae
**Radiata homologs**						
GASU01090694	Aqp H1	Stony coral	*Acropora cervicornis*	Cnidaria	Scleractinia	Acroporidae
FX442020	Aqp H1	Stony coral	*Porites australiensis*	Cnidaria	Scleractinia	Poritidae
GASU01090694	Aqp H2	Stony coral	*Acropora cervicornis*	Cnidaria	Scleractinia	Acroporidae
FX442020	Aqp H2	Stony coral	*Porites australiensis*	Cnidaria	Scleractinia	Poritidae
FX492755	Aqp H3	Stony coral	*Porites australiensis*	Cnidaria	Scleractinia	Poritidae
FX447496	Aqp H4	Stony coral	*Porites australiensis*	Cnidaria	Scleractinia	Poritidae
FX492100	Aqp H5	Stony coral	*Porites australiensis*	Cnidaria	Scleractinia	Poritidae
EDO28361/v1g148074/XP_001620461	Aqp N	Starlet sea anemone	*Nematostella vectensis*	Cnidaria	Actiniaria	Edwardsiidae
**Alveolata homologs**						
GAKY01183306	Aqp H1	Endosymbiotic dinoflaggelate	*Symbiodinium sp. A1*	Dinophyceae	Suessiales	Symbiodiniaceae
GAFO01008469	Aqp H1	Endosymbiotic dinoflaggelate	*Symbiodinium sp. clade C*	Dinophyceae	Suessiales	Symbiodiniaceae
GAKY01183306	Aqp H2	Endosymbiotic dinoflaggelate	*Symbiodinium sp. A1*	Dinophyceae	Suessiales	Symbiodiniaceae
GAFO01008469	Aqp H2	Endosymbiotic dinoflaggelate	*Symbiodinium sp. clade C*	Dinophyceae	Suessiales	Symbiodiniaceae
GAFO01008469	Aqp H3	Endosymbiotic dinoflaggelate	*Symbiodinium sp. A1*	Dinophyceae	Suessiales	Symbiodiniaceae
GAKY01168000	Aqp H5	Endosymbiotic dinoflaggelate	*Symbiodinium sp. A1*	Dinophyceae	Suessiales	Symbiodiniaceae
GAFP01017341	Aqp H5	Endosymbiotic dinoflaggelate	*Symbiodinium sp. clade C*	Dinophyceae	Suessiales	Symbiodiniaceae
BASF01000746	Aqp H5	Endosymbiotic dinoflaggelate	*Symbiodinium minutum Mf*	Dinophyceae	Suessiales	Symbiodiniaceae
**Protists-Fungi AQP**						
DDB0191271/XP_641629/BAA85158	AqpA	Cellular slime mold	*Dictyostelium discoideum*	Amoebozoa	Dictyosteliida	
AJWI01000352	AqpA	Cellular slime mold	*Dictyostelium intermedium*	Amoebozoa	Dictyosteliida	
XP_003283881	AqpA	Cellular slime mold	*Dictyostelium purpureum*	Amoebozoa	Dictyosteliida	
EGG21443	AqpA	Cellular slime mold	*Dictyostelium fasciculatum*	Amoebozoa	Dictyosteliida	
ADBJ01000050	AqpA	Cellular slime mold	*Polysphondylium pallidum*	Amoebozoa	Dictyosteliida	
DDB0214915	WacA	Cellular slime mold	*Dictyostelium discoideum*	Amoebozoa	Dictyosteliida	
AJWI01007140	WacA	Cellular slime mold	*Dictyostelium intermedium*	Amoebozoa	Dictyosteliida	
ADID01000158	WacA	Cellular slime mold	*Dictyostelium purpureum*	Amoebozoa	Dictyosteliida	
AJWJ01000122	WacA	Cellular slime mold	*Polysphondylium violaceum*	Amoebozoa	Dictyosteliida	
ADBJ01000047	WacA	Cellular slime mold	*Polysphondylium pallidum*	Amoebozoa	Dictyosteliida	

DDB0205768	AqpP	Cellular slime mold	*Dictyostelium discoideum*	Amoebozoa	Dictyosteliida	
AJWI01001707	AqpP	Cellular slime mold	*Dictyostelium intermedium*	Amoebozoa	Dictyosteliida	
ADID01000883	AqpP	Cellular slime mold	*Dictyostelium purpureum*	Amoebozoa	Dictyosteliida	
ADHC01000009	AqpP	Cellular slime mold	*Dictyostelium fasciculatum*	Amoebozoa	Dictyosteliida	
EFA81707	AqpP	Cellular slime mold	*Polysphondylium pallidum*	Amoebozoa	Dictyosteliida	
BAF75061	Aqp	Amoeba	*Amoeba proteus*	Amoebozoa	Tubulinida	Amoebidae
XP_004339464	Aqp	Amoeba	*Acanthamoeba castellanii*	Amoebozoa	Centramoebida	Acanthamoebidae
XP_001527487	Aqy1	Fungus	*Lodderomyces elongisporus*	Ascomycota	Saccharomycetales	Debaryomycetaceae
CCG20420	Aqy1	Fungus	*Candida orthopsilosis*	Ascomycota	Saccharomycetales	
EGW34382	Aqy1	Fungus	*Spathaspora passalidarum*	Ascomycota	Saccharomycetales	Debaryomycetaceae
XP_001383665	Aqy1	Pichia stipitis	*Scheffersomyces stipitis*	Ascomycota	Saccharomycetales	Debaryomycetaceae
YPR192W	Aqy1	Baker´s yeast	*Saccharomyces cerevisiae*	Ascomycota	Saccharomycetales	Saccharomycetacea
**AQP9**						
ENSP00000219919	AQP9	Human	*Homo sapiens*	Euarchontoglires	Primates	Hominidae
ENSPTRP00000012166	AQP9	Chimpanzee	*Pan troglodytes*	Euarchontoglires	Primates	Hominidae
ENSGGOP00000011853	AQP9	Western lowland gorilla	*Gorilla gorilla gorilla*	Euarchontoglires	Primates	Hominidae
ENSPPYP00000007384	AQP9	Sumatran orangutan	*Pongo abelii*	Euarchontoglires	Primates	Hominidae
ENSNLEP00000013765	AQP9	Northern white-cheeked gibbon	*Nomascus leucogenys*	Euarchontoglires	Primates	Hylobatidae
ENSMMUP00000014712	AQP9	Rhesus macaque	*Macaca mulatta*	Euarchontoglires	Primates	Cercopithecidae
ENSP00000219919	AQP9	Hamadryas baboon	*Papio hamadryas*	Euarchontoglires	Primates	Cercopithecidae
AHZZ01026334	AQP9	Olive baboon	*Papio anubis*	Euarchontoglires	Primates	Cercopithecidae
ENSCJAP00000014666	AQP9	White-tufted-ear marmoset	*Callithrix jacchus*	Euarchontoglires	Primates	Cebidae
XP_003929026	AQP9	Bolivian squirrel monkey	*Saimiri boliviensis boliviensis*	Euarchontoglires	Primates	Cebidae
ENSTSYP00000001355	AQP9	Philippine tarsier	*Tarsius syrichta*	Euarchontoglires	Primates	Tarsiidae
ENSMICP00000005588	AQP9	Gray mouse lemur	*Microcebus murinus*	Euarchontoglires	Primates	Cheirogaleidae
ENSOGAP00000000433	AQP9	Small-eared galago/Bushbaby	*Otolemur garnettii*	Euarchontoglires	Primates	Galagidae
ENSTBEP00000002203	AQP9	Northern tree shrew	*Tupaia belangeri*	Euarchontoglires	Scandentia	Tupaiidae
ELW71355	AQP9	Chinese tree shrew	*Tupaia chinensis*	Euarchontoglires	Scandentia	Tupaiidae
ENSMUSP00000109200	AQP9	Mouse	*Mus musculus*	Euarchontoglires	Rodentia	Muridae
ENSRNOP00000021442	AQP9	Norway rat	*Rattus norvegicus*	Euarchontoglires	Rodentia	Muridae
XP_003495086	AQP9	Chinese hamster	*Cricetulus griseus*	Euarchontoglires	Rodentia	Cricetidae
ENSDORP00000007120	AQP9	Ord's kangaroo rat	*Dipodomys ordii*	Euarchontoglires	Rodentia	Heteromyidae
ENSCPOP00000011915/XP_003462107	AQP9	Domestic guinea pig	*Cavia porcellus*	Euarchontoglires	Rodentia	Caviidae
ENSSTOP00000012659	AQP9	Thirteen-lined ground squirrel	*Ictidomys tridecemlineatus*	Euarchontoglires	Rodentia	Sciuridae
ENSOPRP00000014273	AQP9	American pika	*Ochotona princeps*	Euarchontoglires	Lagomorpha	Ochotonidae
ENSOCUP00000008440	AQP9	Rabbit	*Oryctolagus cuniculus*	Euarchontoglires	Lagomorpha	Leporidae
ENSEEUP00000013310	AQP6	Western European hedgehog	*Erinaceus europaeus*	Laurasiatheria	Insectivora	Erinaceinae
ENSSARP00000010833	AQP9	Common shrew	*Sorex araneus*	Laurasiatheria	Insectivora	Soricidae
ENSPVAP00000008897	AQP9	Large flying fox/Megabat	*Pteropus vampyrus*	Laurasiatheria	Chiroptera	Pteropodidae
ELK05380/ALWS01090306	AQP9	Black flying fox	*Pteropus alecto*	Laurasiatheria	Chiroptera	Pteropodidae
ALEH01043917	AQP9-1	Big brown bat	*Eptesicus fuscus*	Laurasiatheria	Chiroptera	Vespertilionidae
ALEH01022282	AQP9-2	Big brown bat	*Eptesicus fuscus*	Laurasiatheria	Chiroptera	Vespertilionidae
ALEH01006118	AQP9-3	Big brown bat	*Eptesicus fuscus*	Laurasiatheria	Chiroptera	Vespertilionidae
AAPE02011488/AAPE02011487/ ENSMLUP00000001007	AQP9-1	Little brown bat/Microbat	*Myotis lucifugus*	Laurasiatheria	Chiroptera	Vespertilionidae
AAPE02022705/ ENSMLUP00000017800	AQP9-2	Little brown bat/Microbat	*Myotis lucifugus*	Laurasiatheria	Chiroptera	Vespertilionidae
AAPE02001507/ ENSMLUG00000024335	AQP9-3	Little brown bat/Microbat	*Myotis lucifugus*	Laurasiatheria	Chiroptera	Vespertilionidae
ALWT01117125	AQP9-1	David´s myotis	*Myotis davidii*	Laurasiatheria	Chiroptera	Vespertilionidae
ALWT01078989	AQP9-2	David´s myotis	*Myotis davidii*	Laurasiatheria	Chiroptera	Vespertilionidae
ENSVPAP00000010369	AQP9	Alpaca	*Vicugna pacos*	Laurasiatheria	Tylopoda	Camelidae
ENSBTAP00000017894	AQP9	Sheep	*Ovis aries*	Laurasiatheria	Ruminantia	Bovidae
ENSBTAP00000017894	AQP9	Cow	*Bos taurus*	Laurasiatheria	Ruminantia	Bovidae
ELR45536	AQP9	Yak	*Bos mutus*	Laurasiatheria	Ruminantia	Bovidae
ANOL02038131	AQP9	Killer whale	*Orcinus orca*	Laurasiatheria	Cetacea	Delphinidae
ENSTTRP00000004860	AQP9	Bottlenosed dolphin	*Tursiops truncatus*	Laurasiatheria	Cetacea	Delphinidae
ENSSSCP00000004954	AQP9	Pig	*Sus scrofa*	Laurasiatheria	Suina	Suidae
EFB23520	AQP9	Giant panda	*Ailuropoda melanoleuca*	Laurasiatheria	Carnivora	Ursidae
ENSCAFP00000024352	AQP9	Dog	*Canis lupus familiaris*	Laurasiatheria	Carnivora	Canidae
ENSCAFP00000024352	AQP9	Domestic ferret	*Mustela putorius furo*	Laurasiatheria	Carnivora	Mustelidae
ENSFCAP00000010263	AQP9	Domestic cat	*Felis catus*	Laurasiatheria	Carnivora	Felidae
ENSECAP00000010600	AQP9	Horse	*Equus caballus*	Laurasiatheria	Perissodactyla	Equidae
XP_004421682	AQP9	Southern white rhinocerus	*Ceratotherium simum simum*	Laurasiatheria	Perissodactyla	Rhinocerotidae
ENSPCAP00000004107	AQP9	Cape rock hyrax	*Procavia capensis*	Afrotheria	Hyracoidea	Procaviidae
ENSLAFP00000010161	AQP9	African savanna elephant	*Loxodonta africana*	Afrotheria	Proboscidea	Elephantidae
AHIN01039528	AQP9	Florida manatee	*Trichechus manatus latirostris*	Afrotheria	Sirenia	Trichechidae
ENSETEP00000000309	AQP9	Lesser hedgehog tenrec	*Echinops telfairi*	Afrotheria	Afrosoricida	Tenrecidae
ALYB01031433	AQP9	Ardvaark	*Orycteropus afer afer*	Afrotheria	Tubulidentata	Orycteropodidae
ENSDNOP00000000976	AQP9	Nine-banded armadillo	*Dasypus novemcinctus*	Xenarthra	Cingulata	Dasypodidae
ENSCHOP00000005421	AQP9	Hoffmann's two-fingered sloth	*Choloepus hoffmanni*	Xenarthra	Pilosa	Megalonychidae
ENSMEUP00000012468	AQP9	Tammar wallaby	*Macropus eugenii*	Metatheria	Diprotodontia	Macropodidae
ENSSHAP00000013384	AQP9	Tasmanian devil	*Sarcophilus harrisii*	Metatheria	Dasyuromorphia	Dasyuridae
ENSMODP00000010777	AQP9	Gray short-tailed opossum	*Monodelphis domestica*	Metatheria	Didelphimorphia	Didelphidae
ENSOANP00000021925	AQP9	Platypus	*Ornithorhynchus anatinus*	Prototheria	Monotremata	Ornithorhynchidae
AGAI01054226/AGAI01054227	AQP9	Budgerigar	*Melopsittacus undulatus*	Aves	Psittaciformes	Psittacidae

AMXX01076578/AMXX01055805/AMXX01021731/ AMXX01068820/AMXX01160063/ AOUJ01245559/ AOUJ01394219/AOUJ01082148/AOUJ01278220/ AOUJ01085378	AQP9	Scarlet macaw	*Ara macao*	Aves	Psittaciformes	Psittacidae
CAVT010016951	AQP9	Common canary	*Serinus canaria*	Aves	Passeriformes	Fringillidae
AKZB01010303	AQP9	Medium ground finch	*Geospiza fortis*	Aves	Passeriformes	Fringillidae
ENSTGUP00000006336	AQP9	Zebra finch	*Taeniopygia guttata*	Aves	Passeriformes	Estrildidae
AGTO01020818	AQP9	Collared flycatcher	*Ficedula albicollis*	Aves	Passeriformes	Muscicapidae
EMC81258	AQP9	Rock pigeon	*Columba livia*	Aves	Columbiformes	Columbidae
ENSAPLP00000009910	AQP9	Mallard	*Anas platyrhynchos*	Aves	Anseriformes	Anatidae
ENSMGAP00000005421	AQP9	Turkey	*Meleagris gallopavo*	Aves	Galliformes	Phasianidae
ENSGALP00000038464	AQP9	Chicken	*Gallus gallus*	Aves	Galliformes	Phasianidae
AKHW01033073	AQP9	American alligator	*Alligator mississippiensis*	Archosauria	Crocodylia	Alligatoridae
AVPB01021124/AVPB01021125	AQP9	Chinese alligator	*Alligator sinensis*	Archosauria	Crocodylia	Alligatoridae
ENSPSIP00000018757	AQP9	Chinese softshell turtle	*Pelodiscus sinensis*	Sauropsida	Testudines	Trionychidae
APJP01469951/APJP01460469/APJP01630858	AQP9	Spiny softshell turtle	*Apalone spinifera*	Sauropsida	Testudines	Trionychidae
EMP38987	AQP9	Green seaturtle	*Chelonia mydas*	Sauropsida	Testudines	Cheloniidae
AHGY01440703/AHGY01440705/AHGY01440707	AQP9	Western painted turtle	*Chrysemys picta bellii*	Sauropsida	Testudines	Emydidae
ETE57537/AZIM01000142	AQP9	King cobra	*Ophiophagus hannah*	Lepidosauria	Squamata	Elapidae
AEQU02169704/AEQU02169705	AQP9	Burmese python	*Python molurus bivittatus*	Lepidosauria	Squamata	Pythonidae
ENSACAP00000014594	AQP9	Green anole	*Anolis carolinensis*	Lepidosauria	Squamata	Iguanidae
GAEI01001730	AQP2	Pacific treefrog	*Pseudacris regilla*	Amphibia	Anura	Hylidae
ENSXETP00000023755	AQP9	Western clawed frog	*Xenopus (Silurana) tropicalis*	Amphibia	Anura	Pipidae
GAQK01104642	AQP9	Chinese salamander	*Hynobius chinensis*	Amphibia	Caudata	Hynobiidae
AFYH01095932/AFYH01095933/AFYH01095935/ ENSLACP00000022282	Aqp9	Coelacanth	*Latimeria chalumnae*	Actinistia	Coelacanthiformes	Coelacanthidae
GAPS01056465	Aqp9	Menado coelacanth	*Latimeria menadoensis*	Actinistia	Coelacanthiformes	Coelacanthidae
ENSTRUP00000021028	Aqp9a	Torafugu	*Takifugu rubripes*	Acanthopterygii	Tetraodontiformes	Tetraodontidae
AOOT01011453/AOOT01011454/AOOT01011455/ AOOT01011456/AOOT01011457	Aqp9a	Sansaifugu	*Takifugu flavidus*	Acanthopterygii	Tetraodontiformes	Tetraodontidae
ENSTNIP00000016703	Aqp9a	Green-spotted pufferfish	*Tetraodon nigroviridis*	Acanthopterygii	Tetraodontiformes	Tetraodontidae
AGRG01021247	Aqp9a	Tongue sole	*Cynoglossus semilaevis*	Acanthopterygii	Pleuronectiformes	Cynoglossidae
AGTA02045424	Aqp9a	Zebra mbuna	*Maylandia zebra*	Acanthopterygii	Perciformes	Cichlidae
AFNX01011372	Aqp9a	Red mwanza	*Pundamilia nyererei*	Acanthopterygii	Perciformes	Cichlidae
AFNY01021954	Aqp9a	Lyretail cichlid	*Neolamprologus brichardi*	Acanthopterygii	Perciformes	Cichlidae
AFNZ01017267	Aqp9a	Burton's mouthbrooder	*Haplochromis burtoni*	Acanthopterygii	Perciformes	Cichlidae
ENSONIP00000010493	Aqp9a	Nile tilapia	*Oreochromis niloticus*	Acanthopterygii	Perciformes	Cichlidae
BADN01052475	Aqp9a	Pacific bluefin tuna	*Thunnus orientalis*	Acanthopterygii	Perciformes	Scombridae
AWGY01074468	Aqp9a	Sablefish	*Anoplopoma fimbria*	Acanthopterygii	Scorpaeniformes	Anoplopomatidae
AUPQ01002806	Aqp9a	Flag rockfish	*Sebastes rubrivinctus*	Acanthopterygii	Scorpaeniformes	Sebastidae
AUPR01101625	Aqp9a	Tiger rockfish	*Sebastes nigrocinctus*	Acanthopterygii	Scorpaeniformes	Sebastidae
ENSGACP00000008112	Aqp9a	Three-spined stickleback	*Gasterosteus aculeatus*	Acanthopterygii	Gasterosteiformes	Gasterosteidae
BAAE01185479	Aqp9a	Japanese medaka	*Oryzias latipes*	Acanthopterygii	Beloniformes	Adrianichthyidae
GAIB01121404	Aqp9a	Turquoise killifish	*Nothobranchius furzeri*	Acanthopterygii	Cyprinodontiformes	Nothobranchiidae
AYCK01018417	Aqp9a	Amazon molly	*Poecilia formosa*	Acanthopterygii	Cyprinodontiformes	Poeciliidae
ENSXMAP00000006643	Aqp9a	Southern platyfish	*Xiphophorus maculatus*	Acanthopterygii	Cyprinodontiformes	Poeciliidae
ENSGMOP00000004525	Aqp9a	Atlantic cod	*Gadus morhua*	Paracanthopterygii	Gadiformes	Gadidae
CCAF010107422	Aqp9a1	Rainbow trout	*Oncorhynchus mykiss*	Protacanthopterygii	Salmoniformes	Salmonidae
AGKD01252585/AGKD01126390/AGKD01010521	Aqp9a1	Atlantic salmon	*Salmo salar*	Protacanthopterygii	Salmoniformes	Salmonidae
AGKD01109773/AGKD01265939/AGKD01211321/ AGKD01370165/AGKD01142451	Aqp9a2	Atlantic salmon	*Salmo salar*	Protacanthopterygii	Salmoniformes	Salmonidae
GAHO01107147/GAHO01051501/GAHO01027437	Aqp9a	Small gill opening goldenline barbel	*Sinocyclocheilus angustiporus*	Ostariophysi	Cypriniformes	Cyprinidae
GAHL01053586/GAHL01053586	Aqp9a	Blind goldenline barbel	*Sinocyclocheilus anophthalmus*	Ostariophysi	Cypriniformes	Cyprinidae
FJ655387	Aqp9a	Zebrafish	*Danio rerio*	Ostariophysi	Cypriniformes	Cyprinidae
APWO01074714	Aqp9a	Mexican tetra	*Astyanax mexicanus*	Ostariophysi	Characiformes	Characidae
AVPY01019823/AVPY01019824	Aqp9a	Japanese eel	*Anguilla japonica*	Elopomorpha	Anguilliformes	Anguillidae
ENSTRUP00000045656	Aqp9b	Torafugu	*Takifugu rubripes*	Acanthopterygii	Tetraodontiformes	Tetraodontidae
AOOT01040226	Aqp9b	Sansaifugu	*Takifugu flavidus*	Acanthopterygii	Tetraodontiformes	Tetraodontidae
ENSTNIP00000012198	Aqp9b	Green-spotted pufferfish	*Tetraodon nigroviridis*	Acanthopterygii	Tetraodontiformes	Tetraodontidae
AGRG01025282	Aqp9b	Tongue sole	*Cynoglossus semilaevis*	Acanthopterygii	Pleuronectiformes	Cynoglossidae
AGTA02006660	Aqp9b	Zebra mbuna	*Maylandia zebra*	Acanthopterygii	Perciformes	Cichlidae
AFNX01029189	Aqp9b	Red mwanza	*Pundamilia nyererei*	Acanthopterygii	Perciformes	Cichlidae
AFNY01001740/AFNY01001741	Aqp9b	Lyretail cichlid	*Neolamprologus brichardi*	Acanthopterygii	Perciformes	Cichlidae
AFNZ01017868/AFNZ01017869	Aqp9b	Burton's mouthbrooder	*Haplochromis burtoni*	Acanthopterygii	Perciformes	Cichlidae
ENSONIP00000007337	Aqp9b	Nile tilapia	*Oreochromis niloticus*	Acanthopterygii	Perciformes	Cichlidae
BADN01003195	Aqp9b	Pacific bluefin tuna	*Thunnus orientalis*	Acanthopterygii	Perciformes	Scombridae
AWGY01022394/AWGY01133405	Aqp9b	Sablefish	*Anoplopoma fimbria*	Acanthopterygii	Scorpaeniformes	Anoplopomatidae
AUPQ01023380	Aqp9b	Flag rockfish	*Sebastes rubrivinctus*	Acanthopterygii	Scorpaeniformes	Sebastidae
AUPR01174017	Aqp9b	Tiger rockfish	*Sebastes nigrocinctus*	Acanthopterygii	Scorpaeniformes	Sebastidae
ENSGACP00000020886	Aqp9b	Three-spined stickleback	*Gasterosteus aculeatus*	Acanthopterygii	Gasterosteiformes	Gasterosteidae
ENSORLP00000010364	Aqp9b	Japanese medaka	*Oryzias latipes*	Acanthopterygii	Beloniformes	Adrianichthyidae
GAIB01109759	Aqp9b	Turquoise killifish	*Nothobranchius furzeri*	Acanthopterygii	Cyprinodontiformes	Nothobranchiidae
AYCK01016323	Aqp9b	Amazon molly	*Poecilia formosa*	Acanthopterygii	Cyprinodontiformes	Poeciliidae
ENSXMAP00000014456	Aqp9b	Southern platyfish	*Xiphophorus maculatus*	Acanthopterygii	Cyprinodontiformes	Poeciliidae
ENSGMOP00000016698	Aqp9b	Atlantic cod	*Gadus morhua*	Paracanthopterygii	Gadiformes	Gadidae

JP742598	Aqp9b	Ayu	*Plecoglossus altivelis*	Protacanthopterygii	Osmeriformes	Osmeridae
ABG24574	Aqp9b	Rainbow smelt	*Osmerus mordax*	Protacanthopterygii	Osmeriformes	Osmeridae
CCAF010175523	Aqp9b1	Rainbow trout	*Oncorhynchus mykiss*	Protacanthopterygii	Salmoniformes	Salmonidae
AGKD01025380/AGKD01043301	Aqp9b1	Atlantic salmon	*Salmo salar*	Protacanthopterygii	Salmoniformes	Salmonidae
AGKD01037951/AGKD01133133/AGKD01006400	Aqp9b2	Atlantic salmon	*Salmo salar*	Protacanthopterygii	Salmoniformes	Salmonidae
DT167910	Aqp9b	Fathead minnow	*Pimephales promelas*	Ostariophysi	Cypriniformes	Cyprinidae
GAHO01100067	Aqp9b	Small gill opening goldenline barbel	*Sinocyclocheilus angustiporus*	Ostariophysi	Cypriniformes	Cyprinidae
GAHL01087848/GAHL01093417	Aqp9b	Blind goldenline barbel	*Sinocyclocheilus anophthalmus*	Ostariophysi	Cypriniformes	Cyprinidae
EU341835/ENSDARP00000069995	Aqp9b	Zebrafish	*Danio rerio*	Ostariophysi	Cypriniformes	Cyprinidae
APWO01008619/APWO01008620/APWO01008621	Aqp9b	Mexican tetra	*Astyanax mexicanus*	Ostariophysi	Characiformes	Characidae
AVPY01127203	Aqp9b	Japanese eel	*Anguilla japonica*	Elopomorpha	Anguilliformes	Anguillidae
AHAT01024872	Aqp9	Spotted gar	*Lepisosteus oculatus*	Holostei	Semionotiformes	Lepisosteidae
AESE010685088/AESE011074221/AESE010154666 /AESE011591079	Aqp9	Little skate	*Leucoraja erinacea*	Chondrichthyes	Rajiformes	Rajidae
AAVX02002482	Aqp9	Ghost shark	*Callorhinchus milii*	Chondrichthyes	Chimaeriformes	Callorhinchidae
**AQP10**						
ENSP00000318355	AQP10	Human	*Homo sapiens*	Euarchontoglires	Primates	Hominidae
ENSPTRP00000044066	AQP10	Chimpanzee	*Pan troglodytes*	Euarchontoglires	Primates	Hominidae
ENSGGOP00000011599	AQP10	Western lowland gorilla	*Gorilla gorilla gorilla*	Euarchontoglires	Primates	Hominidae
ENSPPYP00000000910	AQP10	Sumatran orangutan	*Pongo abelii*	Euarchontoglires	Primates	Hominidae
ENSNLEP00000013741	AQP10	Northern white-cheeked gibbon	*Nomascus leucogenys*	Euarchontoglires	Primates	Hylobatidae
ENSMMUP00000030755	AQP10	Rhesus macaque	*Macaca mulatta*	Euarchontoglires	Primates	Cercopithecidae
ENSP00000318355/GENSCAN00000073042	AQP10	Hamadryas baboon	*Papio hamadryas*	Euarchontoglires	Primates	Cercopithecidae
AHZZ01032599	AQP10	Olive baboon	*Papio anubis*	Euarchontoglires	Primates	Cercopithecidae
ENSCJAP00000017692	AQP10	White-tufted-ear marmoset	*Callithrix jacchus*	Euarchontoglires	Primates	Cebidae
XP_003941910	AQP10	Bolivian squirrel monkey	*Saimiri boliviensis boliviensis*	Euarchontoglires	Primates	Cebidae
ENSMICP00000003203	AQP10	Gray mouse lemur	*Microcebus murinus*	Euarchontoglires	Primates	Cheirogaleidae
ENSOGAP00000004925	AQP10	Small-eared galago/Bushbaby	*Otolemur garnettii*	Euarchontoglires	Primates	Galagidae
ENSTBEP00000002048	AQP10	Northern tree shrew	*Tupaia belangeri*	Euarchontoglires	Scandentia	Tupaiidae
ALAR01116048	AQP10	Chinese tree shrew	*Tupaia chinensis*	Euarchontoglires	Scandentia	Tupaiidae
AEKR01029399	AQP10	Mouse	*Mus musculus*	Euarchontoglires	Rodentia	Muridae
AAHX01018619	AQP10	Norway rat	*Rattus norvegicus*	Euarchontoglires	Rodentia	Muridae
XP_003501706	AQP10	Chinese hamster	*Cricetulus griseus*	Euarchontoglires	Rodentia	Cricetidae
ENSDORP00000008117	AQP10	Ord's kangaroo rat	*Dipodomys ordii*	Euarchontoglires	Rodentia	Heteromyidae
AAKN02015643	AQP10	Domestic guinea pig	*Cavia porcellus*	Euarchontoglires	Rodentia	Caviidae
EHB07289	AQP10	Naked mole-rat	*Heterocephalus glaber*	Euarchontoglires	Rodentia	Bathyergidae
ENSSTOP00000013093	AQP10	Thirteen-lined ground squirrel	*Ictidomys tridecemlineatus*	Euarchontoglires	Rodentia	Sciuridae
ENSOPRP00000011430	AQP10	American pika	*Ochotona princeps*	Euarchontoglires	Lagomorpha	Ochotonidae
ENSOCUP00000014024	AQP10	Rabbit	*Oryctolagus cuniculus*	Euarchontoglires	Lagomorpha	Leporidae
AANN01083412/AANN01311780/AMDU01107128	AQP10	Western European hedgehog	*Erinaceus europaeus*	Laurasiatheria	Insectivora	Erinaceinae
ENSSARP00000008216	AQP10	Common shrew	*Sorex araneus*	Laurasiatheria	Insectivora	Soricidae
ENSPVAP00000002580	AQP10	Large flying fox/Megabat	*Pteropus vampyrus*	Laurasiatheria	Chiroptera	Pteropodidae
ELK02825	AQP10	Black flying fox	*Pteropus alecto*	Laurasiatheria	Chiroptera	Pteropodidae
ENSMLUP00000007752	AQP10	Little brown bat/Microbat	*Myotis lucifugus*	Laurasiatheria	Chiroptera	Vespertilionidae
ENSP00000318355/AMGL01004792	AQP10	Sheep	*Ovis aries*	Laurasiatheria	Ruminantia	Bovidae
XP_003581985	AQP10	Cow	*Bos taurus*	Laurasiatheria	Ruminantia	Bovidae
AGSK01099059	AQP10	Yak	*Bos mutus*	Laurasiatheria	Ruminantia	Bovidae
ANOL02071573	AQP10	Killer whale	*Orcinus orca*	Laurasiatheria	Cetacea	Delphinidae
ENSTTRP00000015030	AQP10	Bottlenosed dolphin	*Tursiops truncatus*	Laurasiatheria	Cetacea	Delphinidae
ENSSSCP00000006983	AQP10	Pig	*Sus scrofa*	Laurasiatheria	Suina	Suidae
ENSAMEP00000012346	AQP10	Giant panda	*Ailuropoda melanoleuca*	Laurasiatheria	Carnivora	Ursidae
ENSCAFP00000025344	AQP10	Dog	*Canis lupus familiaris*	Laurasiatheria	Carnivora	Canidae
ENSCAFP00000025344	AQP10	Domestic ferret	*Mustela putorius furo*	Laurasiatheria	Carnivora	Mustelidae
ENSFCAP00000006561	AQP10	Domestic cat	*Felis catus*	Laurasiatheria	Carnivora	Felidae
JU292194	AQP10	Spotted seal	*Phoca largha*	Laurasiatheria	Carnivora	Felidae
XP_001494035	AQP10	Horse	*Equus caballus*	Laurasiatheria	Perissodactyla	Equidae
ABRQ01433123	AQP10	Cape rock hyrax	*Procavia capensis*	Afrotheria	Hyracoidea	Procaviidae
ENSLAFP00000011791	AQP10	African savanna elephant	*Loxodonta africana*	Afrotheria	Proboscidea	Elephantidae
AHIN01132419	AQP10	Florida manatee	*Trichechus manatus latirostris*	Afrotheria	Sirenia	Trichechidae
AAIY02236968	AQP10	Lesser hedgehog tenrec	*Echinops telfairi*	Afrotheria	Afrosoricida	Tenrecidae
ALYB01202654/ALYB01202653	AQP10	Ardvaark	*Orycteropus afer afer*	Afrotheria	Tubulidentata	Orycteropodidae
AAGV03212996	AQP10	Nine-banded armadillo	*Dasypus novemcinctus*	Xenarthra	Cingulata	Dasypodidae
ENSCHOP00000011475	AQP10	Hoffmann's two-fingered sloth	*Choloepus hoffmanni*	Xenarthra	Pilosa	Megalonychidae
ENSMEUP00000001059	AQP10	Tammar wallaby	*Macropus eugenii*	Metatheria	Diprotodontia	Macropodidae
AEFK01161835	AQP10	Tasmanian devil	*Sarcophilus harrisii*	Metatheria	Dasyuromorphia	Dasyuridae
DY589380	AQP10	Common brushtail	*Trichosurus vulpecula*	Metatheria	Diprotodontia	Phalangeridae
ENSMODP00000021447	AQP10	Gray short-tailed opossum	*Monodelphis domestica*	Metatheria	Didelphimorphia	Didelphidae
AGAI01011169	AQP10	Budgerigar	*Melopsittacus undulatus*	Aves	Psittaciformes	Psittacidae
AMXX01268820/AMXX01186663/AOUJ01266258	AQP10	Scarlet macaw	*Ara macao*	Aves	Psittaciformes	Psittacidae
CAVT010044489/CAVT010044488	AQP10	Common canary	*Serinus canaria*	Aves	Passeriformes	Fringillidae
AKZB01051931	AQP10	Medium ground finch	*Geospiza fortis*	Aves	Passeriformes	Fringillidae
XP_002188060	AQP10	Zebra finch	*Taeniopygia guttata*	Aves	Passeriformes	Estrildidae
ANZD01004762	AQP10	Tibetan ground-tit	*Pseudopodoces humilis*	Aves	Passeriformes	Paridae

AGTO01012145	AQP10	Collared flycatcher	*Ficedula albicollis*	Aves	Passeriformes	Muscicapidae
ENSMGAP00000013807	AQP10	Turkey	*Meleagris gallopavo*	Aves	Galliformes	Phasianidae
AKHW01034781	AQP10	American alligator	*Alligator mississippiensis*	Archosauria	Crocodylia	Alligatoridae
AVPB01053647/AVPB01053648	AQP10	Chinese alligator	*Alligator sinensis*	Archosauria	Crocodylia	Alligatoridae
AZIM01117632/AZIM01026944/AZIM01010803/ AZIM01034621	AQP10	King cobra	*Ophiophagus hannah*	Lepidosauria	Squamata	Elapidae
AEQU02076485/AEQU02076486	AQP10	Burmese python	*Python molurus bivittatus*	Lepidosauria	Squamata	Pythonidae
ENSACAP00000011333	AQP10	Green anole	*Anolis carolinensis*	Lepidosauria	Squamata	Iguanidae
GAEG01016981	AQP10	Green frog	*Rana clamitans*	Amphibia	Anura	Ranidae
ENSXETP00000049658	AQP10	Western clawed frog	*Xenopus (Silurana) tropicalis*	Amphibia	Anura	Pipidae
GAQK01144279	AQP10	Chinese salamander	*Hynobius chinensis*	Amphibia	Caudata	Hynobiidae
AFYH01073904/AFYH01073906/AFYH01073908	Aqp10	Coelacanth	*Latimeria chalumnae*	Actinistia	Coelacanthiformes	Coelacanthidae
GAPS01044178	Aqp10	Menado coelacanth	*Latimeria menadoensis*	Actinistia	Coelacanthiformes	Coelacanthidae
AOOT01054180	Aqp10a	Sansaifugu	*Takifugu flavidus*	Acanthopterygii	Tetraodontiformes	Tetraodontidae
ENSTNIP00000021508	Aqp10aa	Green-spotted pufferfish	*Tetraodon nigroviridis*	Acanthopterygii	Tetraodontiformes	Tetraodontidae
ENSTNIP00000021507	Aqp10ab	Green-spotted pufferfish	*Tetraodon nigroviridis*	Acanthopterygii	Tetraodontiformes	Tetraodontidae
AGRG01014417	Aqp10a	Tongue sole	*Cynoglossus semilaevis*	Acanthopterygii	Pleuronectiformes	Cynoglossidae
AGTA02004822/AGTA02004823	Aqp10a	Zebra mbuna	*Maylandia zebra*	Acanthopterygii	Perciformes	Cichlidae
AFNX01016620	Aqp10a	Red mwanza	*Pundamilia nyererei*	Acanthopterygii	Perciformes	Cichlidae
AFNY01078024/AFNY01078025	Aqp10a	Lyretail cichlid	*Neolamprologus brichardi*	Acanthopterygii	Perciformes	Cichlidae
AFNZ01026154	Aqp10a	Burton's mouthbrooder	*Haplochromis burtoni*	Acanthopterygii	Perciformes	Cichlidae
ENSONIP00000007893	Aqp10a	Nile tilapia	*Oreochromis niloticus*	Acanthopterygii	Perciformes	Cichlidae
BADN01024342/BADN01024341	Aqp10a	Pacific bluefin tuna	*Thunnus orientalis*	Acanthopterygii	Perciformes	Scombridae
AWGY01171536	Aqp10a	Sablefish	*Anoplopoma fimbria*	Acanthopterygii	Scorpaeniformes	Anoplopomatidae
AUPQ01000856/AUPQ01018903	Aqp10a	Flag rockfish	*Sebastes rubrivinctus*	Acanthopterygii	Scorpaeniformes	Sebastidae
AUPR01022718/AUPR01022717	Aqp10a	Tiger rockfish	*Sebastes nigrocinctus*	Acanthopterygii	Scorpaeniformes	Sebastidae
ENSGACP00000016074	Aqp10a	Three-spined stickleback	*Gasterosteus aculeatus*	Acanthopterygii	Gasterosteiformes	Gasterosteidae
ENSORLP00000012050	Aqp10a	Japanese medaka	*Oryzias latipes*	Acanthopterygii	Beloniformes	Adrianichthyidae
GAIB01200719	Aqp10a	Turquoise killifish	*Nothobranchius furzeri*	Acanthopterygii	Cyprinodontiformes	Nothobranchiidae
ENSXMAP00000007174	Aqp10aa	Southern platyfish	*Xiphophorus maculatus*	Acanthopterygii	Cyprinodontiformes	Poeciliidae
AYCK01006466	Aqp10aa	Amazon molly	*Poecilia formosa*	Acanthopterygii	Cyprinodontiformes	Poeciliidae
ENSXMAP00000007179	Aqp10ab	Southern platyfish	*Xiphophorus maculatus*	Acanthopterygii	Cyprinodontiformes	Poeciliidae
AYCK01006466	Aqp10ab	Amazon molly	*Poecilia formosa*	Acanthopterygii	Cyprinodontiformes	Poeciliidae
ENSGMOP00000008513	Aqp10a	Atlantic cod	*Gadus morhua*	Paracanthopterygii	Gadiformes	Gadidae
CCAF010191786/CCAF010155489	Aqp10a1	Rainbow trout	*Oncorhynchus mykiss*	Protacanthopterygii	Salmoniformes	Salmonidae
AGKD01044249	Aqp10a1	Atlantic salmon	*Salmo salar*	Protacanthopterygii	Salmoniformes	Salmonidae
AGKD01200314	Aqp10a2	Atlantic salmon	*Salmo salar*	Protacanthopterygii	Salmoniformes	Salmonidae
EW688162	Aqp10a	Grass carp	*Ctenopharyngodon idella*	Ostariophysi	Cypriniformes	Cyprinidae
FJ655388/ENSDARP00000012559	Aqp10a	Zebrafish	*Danio rerio*	Ostariophysi	Cypriniformes	Cyprinidae
APWO01105564	Aqp10a	Mexican tetra	*Astyanax mexicanus*	Ostariophysi	Characiformes	Characidae
CK402696	Aqp10a	Blue catfish	*Ictalurus furcatus*	Ostariophysi	Siluriformes	Ictaluridae
FD105608	Aqp10a	Channel catfish	*Ictalurus punctatus*	Ostariophysi	Siluriformes	Ictaluridae
AVPY01517596/AVPY01517595/AVPY01161649	Aqp10a	Japanese eel	*Anguilla japonica*	Elopomorpha	Anguilliformes	Anguillidae
ENSTRUP00000024382	Aqp10b	Torafugu	*Takifugu rubripes*	Acanthopterygii	Tetraodontiformes	Tetraodontidae
AOOT01088343/AOOT01088344/AOOT01088345/ AOOT01088346	Aqp10b	Sansaifugu	*Takifugu flavidus*	Acanthopterygii	Tetraodontiformes	Tetraodontidae
ENSTNIP00000020714	Aqp10b	Green-spotted pufferfish	*Tetraodon nigroviridis*	Acanthopterygii	Tetraodontiformes	Tetraodontidae
AGRG01015696	Aqp10b	Tongue sole	*Cynoglossus semilaevis*	Acanthopterygii	Pleuronectiformes	Cynoglossidae
AAR13054	Aqp10b	Gilthead seabream	*Sparus aurata*	Acanthopterygii	Perciformes	Sparidae
AGTA02013872	Aqp10b	Zebra mbuna	*Maylandia zebra*	Acanthopterygii	Perciformes	Cichlidae
AFNX01027601	Aqp10b	Red mwanza	*Pundamilia nyererei*	Acanthopterygii	Perciformes	Cichlidae
AFNY01020099	Aqp10b	Lyretail cichlid	*Neolamprologus brichardi*	Acanthopterygii	Perciformes	Cichlidae
AFNZ01015334	Aqp10b	Burton's mouthbrooder	*Haplochromis burtoni*	Acanthopterygii	Perciformes	Cichlidae
ENSONIP00000024860	Aqp10b	Nile tilapia	*Oreochromis niloticus*	Acanthopterygii	Perciformes	Cichlidae
DQ889224	Aqp10b	European seabass	*Dicentrarchus labrax*	Acanthopterygii	Perciformes	Moronidae
BADN01043465/BADN01043464	Aqp10b	Pacific bluefin tuna	*Thunnus orientalis*	Acanthopterygii	Perciformes	Scombridae
ACQ58348/AWGY01169699	Aqp10b	Sablefish	*Anoplopoma fimbria*	Acanthopterygii	Scorpaeniformes	Anoplopomatidae
AUPQ01078292	Aqp10b	Flag rockfish	*Sebastes rubrivinctus*	Acanthopterygii	Scorpaeniformes	Sebastidae
AUPR01011084	Aqp10b	Tiger rockfish	*Sebastes nigrocinctus*	Acanthopterygii	Scorpaeniformes	Sebastidae
ENSGACP00000005841	Aqp10b	Three-spined stickleback	*Gasterosteus aculeatus*	Acanthopterygii	Gasterosteiformes	Gasterosteidae
ENSORLP00000000413	Aqp10b	Japanese medaka	*Oryzias latipes*	Acanthopterygii	Beloniformes	Adrianichthyidae
GAIB01023516	Aqp10b	Turquoise killifish	*Nothobranchius furzeri*	Acanthopterygii	Cyprinodontiformes	Nothobranchiidae
AYCK01023622	Aqp10b	Amazon molly	*Poecilia formosa*	Acanthopterygii	Cyprinodontiformes	Poeciliidae
ENSXMAP00000015292	Aqp10b	Southern platyfish	*Xiphophorus maculatus*	Acanthopterygii	Cyprinodontiformes	Poeciliidae
ENSGMOP00000011541	Aqp10b	Atlantic cod	*Gadus morhua*	Paracanthopterygii	Gadiformes	Gadidae
EL537770	Aqp10b	Rainbow smelt	*Osmerus mordax*	Protacanthopterygii	Osmeriformes	Osmeridae
BX910627/CCAF010001940	Aqp10b1	Rainbow trout	*Oncorhynchus mykiss*	Protacanthopterygii	Salmoniformes	Salmonidae
AGKD01090696	Aqp10b1	Atlantic salmon	*Salmo salar*	Protacanthopterygii	Salmoniformes	Salmonidae
AGKD01157343	Aqp10b2	Atlantic salmon	*Salmo salar*	Protacanthopterygii	Salmoniformes	Salmonidae
EV249895	Aqp10b	Lake whitefish	*Coregonus clupeaformis*	Protacanthopterygii	Salmoniformes	Salmonidae
DT173352	Aqp10b	Fathead minnow	*Pimephales promelas*	Ostariophysi	Cypriniformes	Cyprinidae
EU341836/ENSDARP00000076033	Aqp10b	Zebrafish	*Danio rerio*	Ostariophysi	Cypriniformes	Cyprinidae
APWO01008903	Aqp10b	Mexican tetra	*Astyanax mexicanus*	Ostariophysi	Characiformes	Characidae
FD177421	Aqp10b	Channel catfish	*Ictalurus punctatus*	Ostariophysi	Siluriformes	Ictaluridae
CAH04573	Aqp10b	European eel	*Anguilla anguilla*	Elopomorpha	Anguilliformes	Anguillidae

BAH89255/ AVPY01266395/AVPY01294745/AVPY01294744/ AVPY01294743/ AVPY01779473	Aqp10b1	Japanese eel	*Anguilla japonica*	Elopomorpha	Anguilliformes	Anguillidae
AVPY01294751/AVPY01266384/AVPY01266386/ AVPY01266387/AVPY01266388/ AVPY01753058	Aqp10b2	Japanese eel	*Anguilla japonica*	Elopomorpha	Anguilliformes	Anguillidae
AHAT01020045/AHAT01020044/AHAT01020043/ ENSLOCP00000009159	Aqp10	Spotted gar	*Lepisosteus oculatus*	Holostei	Semionotiformes	Lepisosteidae
AHAT01020047/AHAT01020046/ GENSCAN00000012520	Aqp10L	Spotted gar	*Lepisosteus oculatus*	Holostei	Semionotiformes	Lepisosteidae
AESE011698308/AESE011634621/AESE011756722 /AESE011256220/AESE010860518	Aqp10	Little skate	*Leucoraja erinacea*	Chondrichthyes	Rajiformes	Rajidae
AAVX02008359	Aqp10	Ghost shark	*Callorhinchus milii*	Chondrichthyes	Chimaeriformes	Callorhinchidae
ENSPMAP00000008286/AEFG01045232	Aqp10L1	Sea lamprey	*Petromyzon marinus*	Hyperoartia	Petromyzontiformes	Petromyzontidae
APJL01010233	Aqp10L1	Arctic lamprey	*Lethenteron camtschaticum*	Hyperoartia	Petromyzontiformes	Petromyzontidae
ENSPMAP00000003207/AEFG01052647	Aqp10L2	Sea lamprey	*Petromyzon marinus*	Hyperoartia	Petromyzontiformes	Petromyzontidae
APJL01010233	Aqp10L2	Arctic lamprey	*Lethenteron camtschaticum*	Hyperoartia	Petromyzontiformes	Petromyzontidae
**AQP11**						
ENSP00000318770	AQP11	Human	*Homo sapiens*	Euarchontoglires	Primates	Hominidae
ENSPTRP00000007062	AQP11	Chimpanzee	*Pan troglodytes*	Euarchontoglires	Primates	Hominidae
ENSGGOP00000011686	AQP11	Western lowland gorilla	*Gorilla gorilla gorilla*	Euarchontoglires	Primates	Hominidae
ENSPPYP00000004239	AQP11	Sumatran orangutan	*Pongo abelii*	Euarchontoglires	Primates	Hominidae
ENSNLEP00000020273	AQP11	Northern white-cheeked gibbon	*Nomascus leucogenys*	Euarchontoglires	Primates	Hylobatidae
ENSMMUP00000024926	AQP11	Rhesus macaque	*Macaca mulatta*	Euarchontoglires	Primates	Cercopithecidae
ENSP00000318770	AQP11	Hamadryas baboon	*Papio hamadryas*	Euarchontoglires	Primates	Cercopithecidae
XM_003910458	AQP11	Olive baboon	*Papio anubis*	Euarchontoglires	Primates	Cercopithecidae
ENSCJAP00000027512	AQP11	White-tufted-ear marmoset	*Callithrix jacchus*	Euarchontoglires	Primates	Cebidae
XP_003935086	AQP11	Bolivian squirrel monkey	*Saimiri boliviensis boliviensis*	Euarchontoglires	Primates	Cebidae
ABRT010057814	AQP11	Philippine tarsier	*Tarsius syrichta*	Euarchontoglires	Primates	Tarsiidae
ABDC01304253	AQP11	Gray mouse lemur	*Microcebus murinus*	Euarchontoglires	Primates	Cheirogaleidae
ENSOGAP00000008223	AQP11	Small-eared galago/Bushbaby	*Otolemur garnettii*	Euarchontoglires	Primates	Galagidae
AAPY01308260	AQP11	Northern tree shrew	*Tupaia belangeri*	Euarchontoglires	Scandentia	Tupaiidae
ALAR01061175	AQP11	Chinese tree shrew	*Tupaia chinensis*	Euarchontoglires	Scandentia	Tupaiidae
ENSMUSP00000082054	AQP11	Mouse	*Mus musculus*	Euarchontoglires	Rodentia	Muridae
ENSRNOP00000018091	AQP11	Norway rat	*Rattus norvegicus*	Euarchontoglires	Rodentia	Muridae
XP_003510445	AQP11	Chinese hamster	*Cricetulus griseus*	Euarchontoglires	Rodentia	Cricetidae
ENSDORP00000015180	AQP11	Ord's kangaroo rat	*Dipodomys ordii*	Euarchontoglires	Rodentia	Heteromyidae
ENSCPOP00000004727	AQP11	Domestic guinea pig	*Cavia porcellus*	Euarchontoglires	Rodentia	Caviidae
EHB01284	AQP11	Naked mole-rat	*Heterocephalus glaber*	Euarchontoglires	Rodentia	Bathyergidae
ENSSTOP00000003585	AQP11	Thirteen-lined ground squirrel	*Ictidomys tridecemlineatus*	Euarchontoglires	Rodentia	Sciuridae
ENSOPRP00000009607	AQP11	American pika	*Ochotona princeps*	Laurasiatheria	Lagomorpha	Ochotonidae
ENSOCUP00000002810	AQP11	Rabbit	*Oryctolagus cuniculus*	Laurasiatheria	Lagomorpha	Leporidae
ENSSARP00000008545	AQP11	Common shrew	*Sorex araneus*	Laurasiatheria	Insectivora	Soricidae
XP_004683290	AQP11	Star-nosed mole	*Condylura cristata*	Laurasiatheria	Insectivora	Talpidae
ENSPVAP00000012492	AQP11	Large flying fox/Megabat	*Pteropus vampyrus*	Laurasiatheria	Chiroptera	Pteropodidae
ENSMLUP00000008870	AQP11	Little brown bat/Microbat	*Myotis lucifugus*	Laurasiatheria	Chiroptera	Vespertilionidae
ENSVPAP00000009668/XP_006213705	AQP11	Alpaca	*Vicugna pacos*	Laurasiatheria	Tylopoda	Camelidae
XP_006191706	AQP11	Wild Bactrian camel	*Camelus ferus*	Laurasiatheria	Tylopoda	Camelidae
XP_004019484/AMGL01060868	AQP11	Sheep	*Ovis aries*	Laurasiatheria	Ruminantia	Bovidae
AJPT01229372	AQP11	Goat	*Capra hircus*	Laurasiatheria	Ruminantia	Bovidae
ENSBTAP00000026547	AQP11	Cow	*Bos taurus*	Laurasiatheria	Ruminantia	Bovidae
ELR47367	AQP11	Yak	*Bos mutus*	Laurasiatheria	Ruminantia	Bovidae
AGFL01204710	AQP11	Zebu	*Bos indicus*	Laurasiatheria	Ruminantia	Bovidae
ENSTTRP00000006655	AQP11	Bottlenosed dolphin	*Tursiops truncatus*	Laurasiatheria	Cetacea	Delphinidae
ANOL02054196/XP_004279877	AQP11	Killer whale	*Orcinus orca*	Laurasiatheria	Cetacea	Delphinidae
ENSSSCP00000015800	AQP11	Pig	*Sus scrofa*	Laurasiatheria	Suina	Suidae
ENSAMEP00000005959	AQP11	Giant panda	*Ailuropoda melanoleuca*	Laurasiatheria	Carnivora	Ursidae
ENSCAFP00000007308	AQP11	Dog	*Canis lupus familiaris*	Laurasiatheria	Carnivora	Canidae
ENSCAFP00000007308	AQP11	Domestic ferret	*Mustela putorius furo*	Laurasiatheria	Carnivora	Mustelidae
ENSFCAP00000006902/XP_003992750	AQP11	Domestic cat	*Felis catus*	Laurasiatheria	Carnivora	Felidae
ENSECAP00000015158/XP_001494555	AQP11	Horse	*Equus caballus*	Laurasiatheria	Perissodactyla	Equidae
XP_004434089	AQP11	Southern white rhinocerus	*Ceratotherium simum simum*	Laurasiatheria	Perissodactyla	Rhinocerotidae
ENSLAFP00000023596	AQP11	African savanna elephant	*Loxodonta africana*	Afrotheria	Proboscidea	Elephantidae
AHIN01084737/XP_004382050	AQP11	Florida manatee	*Trichechus manatus latirostris*	Afrotheria	Sirenia	Trichechidae
ABRQ01192979	AQP11	Cape rock hyrax	*Procavia capensis*	Afrotheria	Hyracoidea	Procaviidae
ENSETEP00000004273	AQP11	Lesser hedgehog tenrec	*Echinops telfairi*	Afrotheria	Afrosoricida	Tenrecidae
ALYB01103069	AQP11	Ardvaark	*Orycteropus afer afer*	Afrotheria	Tubulidentata	Orycteropodidae
ENSDNOP00000007102	AQP11	Nine-banded armadillo	*Dasypus novemcinctus*	Xenarthra	Cingulata	Dasypodidae
ENSCHOP00000011681	AQP11	Hoffmann's two-fingered sloth	*Choloepus hoffmanni*	Xenarthra	Pilosa	Megalonychidae
ENSMEUP00000004424	AQP11	Tammar wallaby	*Macropus eugenii*	Metatheria	Diprotodontia	Macropodidae
ENSSHAP00000012436	AQP11	Tasmanian devil	*Sarcophilus harrisii*	Metatheria	Dasyuromorphia	Dasyuridae
ENSMODP00000006192	AQP11	Gray short-tailed opossum	*Monodelphis domestica*	Metatheria	Didelphimorphia	Didelphidae
ENSOANP00000015652	AQP11	Platypus	*Ornithorhynchus anatinus*	Prototheria	Monotremata	Ornithorhynchidae
AGAI01051433	AQP11	Budgerigar	*Melopsittacus undulatus*	Aves	Psittaciformes	Psittacidae
AMXX01102566/AMXX01186982/AOUJ01032445/ AOUJ01032441	AQP11	Scarlet macaw	*Ara macao*	Aves	Psittaciformes	Psittacidae
CAVT010007100	AQP11	Common canary	*Serinus canaria*	Aves	Passeriformes	Fringillidae

AKZB01048616	AQP11	Medium ground finch	*Geospiza fortis*	Aves	Passeriformes	Fringillidae
XP_005495534	AQP11	White-throated sparrow	*Zonotrichia albicollis*	Aves	Passeriformes	Fringillidae
XP_005531056	AQP11	Tibetan ground-tit	*Pseudopodoces humilis*	Aves	Passeriformes	Paridae
ENSTGUP00000013419/ABQF01027693/ABQF01027694	AQP11	Zebra finch	*Taeniopygia guttata*	Aves	Passeriformes	Estrildidae
JR864707	AQP11	Vinous-throated parrotbill	*Paradoxornis webbianus*	Aves	Passeriformes	Muscicapidae
AGTO01000778	AQP11	Collared flycatcher	*Ficedula albicollis*	Aves	Passeriformes	Muscicapidae
XP_005231201	AQP11	Peregrin falcon	*Falco peregrinus*	Aves	Falconiformes	Falconidae
XP_005441017	AQP11	Saker falcon	*Falco cherrug*	Aves	Falconiformes	Falconidae
XP_005500642	AQP11	Rock pigeon	*Columba livia*	Aves	Columbiformes	Columbidae
ENSAPLP00000006367	AQP11	Mallard	*Anas platyrhynchos*	Aves	Anseriformes	Anatidae
ENSMGAP00000016194	AQP11	Turkey	*Meleagris gallopavo*	Aves	Galliformes	Phasianidae
ENSGALP00000002455	AQP11	Chicken	*Gallus gallus*	Aves	Galliformes	Phasianidae
AKHW01075947	AQP11	American alligator	*Alligator mississippiensis*	Archosauria	Crocodylia	Alligatoridae
AVPB01133327/AVPB01133328	AQP11	Chinese alligator	*Alligator sinensis*	Archosauria	Crocodylia	Alligatoridae
ENSPSIP00000005055	AQP11	Chinese softshell turtle	*Pelodiscus sinensis*	Sauropsida	Testudines	Trionychidae
EMP28605	AQP11	Green seaturtle	*Chelonia mydas*	Sauropsida	Testudines	Cheloniidae
JW302207	AQP11	Red-eared slider turtle	*Trachemys scripta elegans*	Sauropsida	Testudines	Emydidae
AHGY01077190	AQP11	Western painted turtle	*Chrysemys picta bellii*	Sauropsida	Testudines	Emydidae
AZIM01006374	AQP11	King cobra	*Ophiophagus hannah*	Lepidosauria	Squamata	Elapidae
AEQU02153142/AEQU02153141	AQP11	Burmese python	*Python molurus bivittatus*	Lepidosauria	Squamata	Pythonidae
AAWZ02025389/ENSACAP00000001854	AQP11	Green anole	*Anolis carolinensis*	Lepidosauria	Squamata	Iguanidae
AAI33223	AQP11	African clawed frog	*Xenopus laevis*	Amphibia	Anura	Pipidae
ENSXETP00000058403	AQP11	Western clawed frog	*Xenopus (Silurana) tropicalis*	Amphibia	Anura	Pipidae
FS311168	AQP11	Japanese firebelly newt	*Cynops pyrrhogaster*	Amphibia	Caudata	Salamandridae
GAQK01082401	AQP11	Chinese salamander	*Hynobius chinensis*	Amphibia	Caudata	Hynobiidae
AFYH01170435/AFYH01170433/ENSLACP00000007328	Aqp11	Coelacanth	*Latimeria chalumnae*	Actinistia	Coelacanthiformes	Coelacanthidae
GAPS01010023	Aqp11	Menado coelacanth	*Latimeria menadoensis*	Actinistia	Coelacanthiformes	Coelacanthidae
ENSTRUP00000025165	Aqp11a	Torafugu	*Takifugu rubripes*	Acanthopterygii	Tetraodontiformes	Tetraodontidae
GSTENT10023417001	Aqp11a	Green-spotted pufferfish	*Tetraodon nigroviridis*	Acanthopterygii	Tetraodontiformes	Tetraodontidae
AGRG01015803	Aqp11a	Tongue sole	*Cynoglossus semilaevis*	Acanthopterygii	Pleuronectiformes	Cynoglossidae
AGTA02049030	Aqp11a	Zebra mbuna	*Maylandia zebra*	Acanthopterygii	Perciformes	Cichlidae
AFNX01034372	Aqp11a	Red mwanza	*Pundamilia nyererei*	Acanthopterygii	Perciformes	Cichlidae
AFNY01079048/AFNY01079049	Aqp11a	Lyretail cichlid	*Neolamprologus brichardi*	Acanthopterygii	Perciformes	Cichlidae
AFNZ01043460	Aqp11a	Burton's mouthbrooder	*Haplochromis burtoni*	Acanthopterygii	Perciformes	Cichlidae
ENSONIP00000012033/AERX01031972	Aqp11a	Nile tilapia	*Oreochromis niloticus*	Acanthopterygii	Perciformes	Cichlidae
DB864935	Aqp11a	Matumbi hunter	*Lipochromis sp.*	Acanthopterygii	Perciformes	Cichlidae
BADN01081994/BADN01081995	Aqp11a	Pacific bluefin tuna	*Thunnus orientalis*	Acanthopterygii	Perciformes	Scombridae
GAJJ01007087/GAJJ01013977/AWGY01082912/AWGY01127580	Aqp11a	Sablefish	*Anoplopoma fimbria*	Acanthopterygii	Scorpaeniformes	Anoplopomatidae
AUPQ01080838	Aqp11a	Flag rockfish	*Sebastes rubrivinctus*	Acanthopterygii	Scorpaeniformes	Sebastidae
AUPR01165769	Aqp11a	Tiger rockfish	*Sebastes nigrocinctus*	Acanthopterygii	Scorpaeniformes	Sebastidae
ENSGACP00000027582	Aqp11a	Three-spined stickleback	*Gasterosteus aculeatus*	Acanthopterygii	Gasterosteiformes	Gasterosteidae
ENSORLP00000018866	Aqp11a	Japanese medaka	*Oryzias latipes*	Acanthopterygii	Beloniformes	Adrianichthyidae
GAIB01110151	Aqp11a	Turquoise killifish	*Nothobranchius furzeri*	Acanthopterygii	Cyprinodontiformes	Nothobranchiidae
HO912098	Aqp11a	Blackspotted livebearer	*Poeciliopsis turneri*	Acanthopterygii	Cyprinodontiformes	Poeciliidae
AYCK01009593	Aqp11a	Amazon molly	*Poecilia formosa*	Acanthopterygii	Cyprinodontiformes	Poeciliidae
ENSXMAP00000000645	Aqp11a	Southern platyfish	*Xiphophorus maculatus*	Acanthopterygii	Cyprinodontiformes	Poeciliidae
ENSGMOP00000006569	Aqp11a	Atlantic cod	*Gadus morhua*	Paracanthopterygii	Gadiformes	Gadidae
JP737092	Aqp11a	Ayu	*Plecoglossus altivelis*	Protacanthopterygii	Osmeriformes	Osmeridae
EZ807701/EZ814810/CCAF010005608	Aqp11a1	Rainbow trout	*Oncorhynchus mykiss*	Protacanthopterygii	Salmoniformes	Salmonidae
AGKD01092847/GE766931	Aqp11a1	Atlantic salmon	*Salmo salar*	Protacanthopterygii	Salmoniformes	Salmonidae
CCAF010031865	Aqp11a2	Rainbow trout	*Oncorhynchus mykiss*	Protacanthopterygii	Salmoniformes	Salmonidae
AGKD01029804	Aqp11a2	Atlantic salmon	*Salmo salar*	Protacanthopterygii	Salmoniformes	Salmonidae
APWO01075838/ENSAMXP00000007062	Aqp11a	Mexican tetra	*Astyanax mexicanus*	Ostariophysi	Characiformes	Characidae
AVPY01012376	Aqp11aL	Japanese eel	*Anguilla japonica*	Elopomorpha	Anguilliformes	Anguillidae
ENSTRUP00000017679	Aqp11b	Torafugu	*Takifugu rubripes*	Acanthopterygii	Tetraodontiformes	Tetraodontidae
AOOT01001028/AOOT01001029/AOOT01001030	Aqp11b	Sansaifugu	*Takifugu flavidus*	Acanthopterygii	Tetraodontiformes	Tetraodontidae
BAL44701	Aqp11b	Mefugu	*Takifugu obscurus*	Acanthopterygii	Tetraodontiformes	Tetraodontidae
AGRG01014057	Aqp11b	Tongue sole	*Cynoglossus semilaevis*	Acanthopterygii	Pleuronectiformes	Cynoglossidae
AGTA02016585	Aqp11b	Zebra mbuna	*Maylandia zebra*	Acanthopterygii	Perciformes	Cichlidae
AFNX01007330_1	Aqp11b	Red mwanza	*Pundamilia nyererei*	Acanthopterygii	Perciformes	Cichlidae
AFNY01013174	Aqp11b	Lyretail cichlid	*Neolamprologus brichardi*	Acanthopterygii	Perciformes	Cichlidae
AFNZ01003545	Aqp11b	Burton's mouthbrooder	*Haplochromis burtoni*	Acanthopterygii	Perciformes	Cichlidae
ENSONIP00000001816/AERX01020914/AERX01020915/XR_269848	Aqp11b	Nile tilapia	*Oreochromis niloticus*	Acanthopterygii	Perciformes	Cichlidae
BADN01123543/BADN01119082	Aqp11b	Pacific bluefin tuna	*Thunnus orientalis*	Acanthopterygii	Perciformes	Scombridae
AWGY01083649/AWGY01116558	Aqp11b	Sablefish	*Anoplopoma fimbria*	Acanthopterygii	Scorpaeniformes	Anoplopomatidae
AUPQ01071328	Aqp11b	Flag rockfish	*Sebastes rubrivinctus*	Acanthopterygii	Scorpaeniformes	Sebastidae
AUPR01089814	Aqp11b	Tiger rockfish	*Sebastes nigrocinctus*	Acanthopterygii	Scorpaeniformes	Sebastidae
DK099434	Aqp11b	Japanese medaka	*Oryzias latipes*	Acanthopterygii	Beloniformes	Adrianichthyidae
GAIB01048953	Aqp11b	Turquoise killifish	*Nothobranchius furzeri*	Acanthopterygii	Cyprinodontiformes	Nothobranchiidae
AYCK01004885	Aqp11b	Amazon molly	*Poecilia formosa*	Acanthopterygii	Cyprinodontiformes	Poeciliidae
FK039477	Aqp11b	Platyfish hybrid	*X. maculatus x X. hellerii*	Acanthopterygii	Cyprinodontiformes	Poeciliidae

AGAJ01029174	Aqp11b	Southern platyfish	*Xiphophorus maculatus*	Acanthopterygii	Cyprinodontiformes	Poeciliidae
ENSGMOP00000020707	Aqp11b	Atlantic cod	*Gadus morhua*	Paracanthopterygii	Gadiformes	Gadidae
ACO09336	Aqp11b	Rainbow smelt	*Osmerus mordax*	Protacanthopterygii	Osmeriformes	Osmeridae
CCAF010079483	Aqp11b1	Rainbow trout	*Oncorhynchus mykiss*	Protacanthopterygii	Salmoniformes	Salmonidae
AGKD01193048	Aqp11b1	Atlantic salmon	*Salmo salar*	Protacanthopterygii	Salmoniformes	Salmonidae
CCAF010068337	Aqp11b2	Rainbow trout	*Oncorhynchus mykiss*	Protacanthopterygii	Salmoniformes	Salmonidae
AGKD01088157	Aqp11b2	Atlantic salmon	*Salmo salar*	Protacanthopterygii	Salmoniformes	Salmonidae
DT287811	Aqp11b	Fathead minnow	*Pimephales promelas*	Ostariophysi	Cypriniformes	Cyprinidae
GAHO01136304/GAHO01095742/GAHO01095336	Aqp11b	Small gill opening goldenline barbel	*Sinocyclocheilus angustiporus*	Ostariophysi	Cypriniformes	Cyprinidae
GAHL01087546	Aqp11b	Blind goldenline barbel	*Sinocyclocheilus anophthalmus*	Ostariophysi	Cypriniformes	Cyprinidae
BC095775/ENSDARP00000062519	Aqp11b	Zebrafish	*Danio rerio*	Ostariophysi	Cypriniformes	Cyprinidae
APWO01110971/ENSAMXP00000014175	Aqp11b	Mexican tetra	*Astyanax mexicanus*	Ostariophysi	Characiformes	Characidae
FD343108	Aqp11b	Channel catfish	*Ictalurus punctatus*	Ostariophysi	Siluriformes	Ictaluridae
GAGX01152394	Aqp11b	Brown bullhead	*Ameiurus nebulosus*	Ostariophysi	Siluriformes	Ictaluridae
AVPY01077179	Aqp11b	Japanese eel	*Anguilla japonica*	Elopomorpha	Anguilliformes	Anguillidae
AHAT01003066/XP_006628169	Aqp11-1	Spotted gar	*Lepisosteus oculatus*	Holostei	Semionotiformes	Lepisosteidae
AHAT01003066/XP_006628168	Aqp11-2	Spotted gar	*Lepisosteus oculatus*	Holostei	Semionotiformes	Lepisosteidae
AESE010020706/AESE012695651/AESE012564622	Aqp11	Little skate	*Leucoraja erinacea*	Chondrichthyes	Rajiformes	Rajidae

AQP12

ENSP00000337144	AQP12A	Human	*Homo sapiens*	Euarchontoglires	Primates	Hominidae
ENSP00000384894	AQP12B	Human	*Homo sapiens*	Euarchontoglires	Primates	Hominidae
ENSGGOP00000019546	AQP12A	Western lowland gorilla	*Gorilla gorilla gorilla*	Euarchontoglires	Primates	Hominidae
ENSGGOP00000022773	AQP12B	Western lowland gorilla	*Gorilla gorilla gorilla*	Euarchontoglires	Primates	Hominidae
ENSPPYP00000014971	AQP12A	Sumatran orangutan	*Pongo abelii*	Euarchontoglires	Primates	Hominidae
ENSNLEP00000016587	AQP12	Northern white-cheeked gibbon	*Nomascus leucogenys*	Euarchontoglires	Primates	Hylobatidae
AEHK01046637	AQP12	Rhesus macaque	*Macaca mulatta*	Euarchontoglires	Primates	Cercopithecidae
EHH55306	AQP12	Crab-eating macaque	*Macaca fascicularis*	Euarchontoglires	Primates	Cercopithecidae
ENSP00000405899	AQP12	Hamadryas baboon	*Papio hamadryas*	Euarchontoglires	Primates	Cercopithecidae
XP_003908224	AQP12	Olive baboon	*Papio anubis*	Euarchontoglires	Primates	Cercopithecidae
ENSCJAP00000012100	AQP12	White-tufted-ear marmoset	*Callithrix jacchus*	Euarchontoglires	Primates	Cebidae
XP_003936813	AQP12	Bolivian squirrel monkey	*Saimiri boliviensis boliviensis*	Euarchontoglires	Primates	Cebidae
ABRT02338252	AQP12	Philippine tarsier	*Tarsius syrichta*	Euarchontoglires	Primates	Tarsiidae
ENSOGAP00000012879/XP_003798328	AQP12	Small-eared galago/Bushbaby	*Otolemur garnettii*	Euarchontoglires	Primates	Galagidae
ALAR01071477/ELW63513	AQP12	Chinese tree shrew	*Tupaia chinensis*	Euarchontoglires	Scandentia	Tupaiidae
ENSMUSP00000060622	AQP12	Mouse	*Mus musculus*	Euarchontoglires	Rodentia	Muridae
ENSRNOP00000005935	AQP12	Norway rat	*Rattus norvegicus*	Euarchontoglires	Rodentia	Muridae
XP_003499323	AQP12	Chinese hamster	*Cricetulus griseus*	Euarchontoglires	Rodentia	Cricetidae
ABRO01568331	AQP12	Ord's kangaroo rat	*Dipodomys ordii*	Euarchontoglires	Rodentia	Heteromyidae
ENSCPOP00000005935	AQP12	Domestic guinea pig	*Cavia porcellus*	Euarchontoglires	Rodentia	Caviidae
EHB16168	AQP12	Naked mole-rat	*Heterocephalus glaber*	Euarchontoglires	Rodentia	Bathyergidae
AGTP01102324	AQP12	Thirteen-lined ground squirrel	*Ictidomys tridecemlineatus*	Euarchontoglires	Rodentia	Sciuridae
ENSOPRP00000004455	AQP12	American pika	*Ochotona princeps*	Euarchontoglires	Lagomorpha	Ochotonidae
AAGW02082056	AQP12	Rabbit	*Oryctolagus cuniculus*	Euarchontoglires	Lagomorpha	Leporidae
ENSSARP00000004517	AQP12	Common shrew	*Sorex araneus*	Laurasiatheria	Insectivora	Soricidae
XP_004695608	AQP12	Star-nosed mole	*Condylura cristata*	Laurasiatheria	Insectivora	Talpidae
ALEH01019818	AQP12	Big brown bat	*Eptesicus fuscus*	Laurasiatheria	Chiroptera	Vespertilionidae
ENSMLUP00000003942	AQP12	Little brown bat/Microbat	*Myotis lucifugus*	Laurasiatheria	Chiroptera	Vespertilionidae
ELK32658	AQP12	David´s myotis	*Myotis davidii*	Laurasiatheria	Chiroptera	Vespertilionidae
ACIV010275453/ACIV010275458	AQP12	Sheep	*Ovis aries*	Laurasiatheria	Ruminantia	Bovidae
ENSBTAP00000004395/XP_005205120	AQP12	Cow	*Bos taurus*	Laurasiatheria	Ruminantia	Bovidae
ELR52245	AQP12	Yak	*Bos mutus*	Laurasiatheria	Ruminantia	Bovidae
AGFL01032459	AQP12	Zebu	*Bos indicus*	Laurasiatheria	Ruminantia	Bovidae
ATDI01036423	AQP12	Minke whale	*Balaenoptera acutorostrata*	Laurasiatheria	Cetacea	Balaenopteridae
AOCR01104471	AQP12	Pig	*Sus scrofa*	Laurasiatheria	Suina	Suidae
XP_006209258	AQP12	Alpaca	*Vicugna pacos*	Laurasiatheria	Tylopoda	Camelidae
XP_006175578	AQP12	Wild Bactrian camel	*Camelus ferus*	Laurasiatheria	Tylopoda	Camelidae
ENSAMEP00000017752	AQP12	Giant panda	*Ailuropoda melanoleuca*	Laurasiatheria	Carnivora	Ursidae
ENSCAFP00000032136/AACN011020224/AAEX03014544	AQP12	Dog	*Canis lupus familiaris*	Laurasiatheria	Carnivora	Canidae
ENSMPUP00000000138	AQP12	Domestic ferret	*Mustela putorius furo*	Laurasiatheria	Carnivora	Mustelidae
ENSFCAP00000009684/XP_003991374	AQP12	Domestic cat	*Felis catus*	Laurasiatheria	Carnivora	Felidae
ENSECAP00000011522	AQP12	Horse	*Equus caballus*	Laurasiatheria	Perissodactyla	Equidae
XP_004427863	AQP12	Southern white rhinocerus	*Ceratotherium simum simum*	Laurasiatheria	Perissodactyla	Rhinocerotidae
ABRQ01627555	AQP12	Cape rock hyrax	*Procavia capensis*	Afrotheria	Hyracoidea	Procaviidae
ENSLAFP00000001690	AQP12	African savanna elephant	*Loxodonta africana*	Afrotheria	Proboscidea	Elephantidae
ENSETEP00000015332	AQP12	Lesser hedgehog tenrec	*Echinops telfairi*	Afrotheria	Afrosoricida	Tenrecidae
AHIN01119182	AQP12	Florida manatee	*Trichechus manatus latirostris*	Afrotheria	Sirenia	Trichechidae
ALYB01014398	AQP12	Ardvaark	*Orycteropus afer afer*	Afrotheria	Tubulidentata	Orycteropodidae
AAGV03163531/AAGV03163532/AAGV03310742	AQP12	Nine-banded armadillo	*Dasypus novemcinctus*	Xenarthra	Cingulata	Dasypodidae
ENSMEUP00000006074	AQP12	Tammar wallaby	*Macropus eugenii*	Metatheria	Diprotodontia	Macropodidae
ENSSHAP00000013776	AQP12	Tasmanian devil	*Sarcophilus harrisii*	Metatheria	Dasyuromorphia	Dasyuridae
ENSMODP00000023015	AQP12	Gray short-tailed opossum	*Monodelphis domestica*	Metatheria	Didelphimorphia	Didelphidae
XP_001507166	AQP12	Platypus	*Ornithorhynchus anatinus*	Prototheria	Monotremata	Ornithorhynchidae

AGAI01047968	AQP12	Budgerigar	*Melopsittacus undulatus*	Aves	Psittaciformes	Psittacidae
AOCU01008568	AQP12	Puerto Rican parrot	*Amazona vittata*	Aves	Psittaciformes	Psittacidae
AOUJ01133298/AMXX01085365	AQP12	Scarlet macaw	*Ara macao*	Aves	Psittaciformes	Psittacidae
CAVT010015752	AQP12	Common canary	*Serinus canaria*	Aves	Passeriformes	Fringillidae
AKZB01087418	AQP12	Medium ground finch	*Geospiza fortis*	Aves	Passeriformes	Fringillidae
XP_005489598	AQP12	White-throated sparrow	*Zonotrichia albicollis*	Aves	Passeriformes	Fringillidae
ENSTGUP00000005991	AQP12	Zebra finch	*Taeniopygia guttata*	Aves	Passeriformes	Estrildidae
XP_005050802	AQP12	Tibetan ground-tit	*Pseudopodoces humilis*	Aves	Passeriformes	Paridae
AGTO01011305	AQP12	Collared flycatcher	*Ficedula albicollis*	Aves	Passeriformes	Muscicapidae
XP_005241620	AQP12	Peregrin falcon	*Falco peregrinus*	Aves	Falconiformes	Falconidae
XP_005437199	AQP12	Saker falcon	*Falco cherrug*	Aves	Falconiformes	Falconidae
XP_005501622	AQP12	Rock pigeon	*Columba livia*	Aves	Columbiformes	Columbidae
ENSAPLP00000015645	AQP12	Mallard	*Anas platyrhynchos*	Aves	Anseriformes	Anatidae
ENSMGAP00000002820	AQP12	Turkey	*Meleagris gallopavo*	Aves	Galliformes	Phasianidae
ENSGALP00000010373	AQP12	Chicken	*Gallus gallus*	Aves	Galliformes	Phasianidae
AKHW01049300	AQP12	American alligator	*Alligator mississippiensis*	Archosauria	Crocodylia	Alligatoridae
AVPB01013368	AQP12	Chinese alligator	*Alligator sinensis*	Archosauria	Crocodylia	Alligatoridae
ENSPSIP00000008491	AQP12	Chinese softshell turtle	*Pelodiscus sinensis*	Sauropsida	Testudines	Trionychidae
EMP38415	AQP12	Green seaturtle	*Chelonia mydas*	Sauropsida	Testudines	Cheloniidae
AHGY01268029/AHGY01268030	AQP12	Western painted turtle	*Chrysemys picta bellii*	Sauropsida	Testudines	Emydidae
AZIM01003495	AQP12	King cobra	*Ophiophagus hannah*	Lepidosauria	Squamata	Elapidae
AEQU02097685	AQP12	Burmese python	*Python molurus bivittatus*	Lepidosauria	Squamata	Pythonidae
ENSACAP00000015972	AQP12	Green anole	*Anolis carolinensis*	Lepidosauria	Squamata	Iguanidae
AAH82904	AQP12	African clawed frog	*Xenopus laevis*	Amphibia	Anura	Pipidae
ENSXETP00000059277	AQP12	Western clawed frog	*Xenopus (Silurana) tropicalis*	Amphibia	Anura	Pipidae
GAQK01013142	AQP12	Chinese salamander	*Hynobius chinensis*	Amphibia	Caudata	Hynobiidae
BAHO01076624/AFYH01210544	Aqp12	Coelacanth	*Latimeria chalumnae*	Actinistia	Coelacanthiformes	Coelacanthidae
GAPS01018381	Aqp12	Menado coelacanth	*Latimeria menadoensis*	Actinistia	Coelacanthiformes	Coelacanthidae
ENSTRUP00000041552	Aqp12	Torafugu	*Takifugu rubripes*	Acanthopterygii	Tetraodontiformes	Tetraodontidae
AOOT01027882	Aqp12	Sansaifugu	*Takifugu flavidus*	Acanthopterygii	Tetraodontiformes	Tetraodontidae
BAL44702	Aqp12	Mefugu	*Takifugu obscurus*	Acanthopterygii	Tetraodontiformes	Tetraodontidae
ENSTNIP00000020773	Aqp12	Green-spotted pufferfish	*Tetraodon nigroviridis*	Acanthopterygii	Tetraodontiformes	Tetraodontidae
JU383146	Aqp12	Turbot	*Scophthalmus maximus*	Acanthopterygii	Pleuronectiformes	Scophthalmidae
GAAQ01001383	Aqp12	Dover sole	*Solea solea*	Acanthopterygii	Pleuronectiformes	Soleidae
AGRG01015271	Aqp12	Tongue sole	*Cynoglossus semilaevis*	Acanthopterygii	Pleuronectiformes	Cynoglossidae
FM150229	Aqp12	Gilthead seabream	*Sparus aurata*	Acanthopterygii	Perciformes	Sparidae
GW670618	Aqp12	Mi-iuy croaker	*Miichthys miiuy*	Acanthopterygii	Perciformes	Sciaenidae
AGTA02025659	Aqp12	Zebra mbuna	*Maylandia zebra*	Acanthopterygii	Perciformes	Cichlidae
AFNX01016726	Aqp12	Red mwanza	*Pundamilia nyererei*	Acanthopterygii	Perciformes	Cichlidae
AFNY01023777	Aqp12	Lyretail cichlid	*Neolamprologus brichardi*	Acanthopterygii	Perciformes	Cichlidae
AFNZ01010976	Aqp12	Burton's mouthbrooder	*Haplochromis burtoni*	Acanthopterygii	Perciformes	Cichlidae
ENSONIP00000003560	Aqp12	Nile tilapia	*Oreochromis niloticus*	Acanthopterygii	Perciformes	Cichlidae
BADN01023638	Aqp12	Pacific bluefin tuna	*Thunnus orientalis*	Acanthopterygii	Perciformes	Scombridae
GW344888	Aqp12	Antarctic spiny plunderfish	*Harpagifer antarcticus*	Acanthopterygii	Perciformes	Harpagiferidae
AWGY01139415/AWGY01139417/AWGY01148047/ GAJJ01023328	Aqp12	Sablefish	*Anoplopoma fimbria*	Acanthopterygii	Scorpaeniformes	Anoplopomatidae
AUPQ01029045	Aqp12	Flag rockfish	*Sebastes rubrivinctus*	Acanthopterygii	Scorpaeniformes	Sebastidae
AUPR01054694	Aqp12	Tiger rockfish	*Sebastes nigrocinctus*	Acanthopterygii	Scorpaeniformes	Sebastidae
ENSGACP00000014661	Aqp12	Three-spined stickleback	*Gasterosteus aculeatus*	Acanthopterygii	Gasterosteiformes	Gasterosteidae
ENSORLP00000001949	Aqp12	Japanese medaka	*Oryzias latipes*	Acanthopterygii	Beloniformes	Adrianichthyidae
GAIB01022997	Aqp12	Turquoise killifish	*Nothobranchius furzeri*	Acanthopterygii	Cyprinodontiformes	Nothobranchiidae
CN980894	Aqp12	Common mummichog	*Fundulus heteroclitus*	Acanthopterygii	Cyprinodontiformes	Fundulidae
ES376331	Aqp12	Guppy	*Poecilia reticulata*	Acanthopterygii	Cyprinodontiformes	Poeciliidae
AYCK01004047	Aqp12	Amazon molly	*Poecilia formosa*	Acanthopterygii	Cyprinodontiformes	Poeciliidae
ENSXMAP00000011346	Aqp12	Southern platyfish	*Xiphophorus maculatus*	Acanthopterygii	Cyprinodontiformes	Poeciliidae
ENSGMOP00000020613	Aqp12	Atlantic cod	*Gadus morhua*	Paracanthopterygii	Gadiformes	Gadidae
CCAF010102094	Aqp12-1	Rainbow trout	*Oncorhynchus mykiss*	Protacanthopterygii	Salmoniformes	Salmonidae
AGKD01402623	Aqp12-1	Atlantic salmon	*Salmo salar*	Protacanthopterygii	Salmoniformes	Salmonidae
CCAF010023537	Aqp12-2	Rainbow trout	*Oncorhynchus mykiss*	Protacanthopterygii	Salmoniformes	Salmonidae
AGKD01127630	Aqp12-2	Atlantic salmon	*Salmo salar*	Protacanthopterygii	Salmoniformes	Salmonidae
EE396542/EE393754	Aqp12	Rare gudgeon	*Gobiocypris rarus*	Ostariophysi	Cypriniformes	Cyprinidae
CA968566	Aqp12	Common carp	*Cyprinus carpio*	Ostariophysi	Cypriniformes	Cyprinidae
GAHO01106926/GAHO01011211/GAHO01072357/ GAHO01107961/GAHO01134744	Aqp12	Small gill opening goldenline barbel	*Sinocyclocheilus angustiporus*	Ostariophysi	Cypriniformes	Cyprinidae
DT137447/GH711388	Aqp12	Fathead minnow	*Pimephales promelas*	Ostariophysi	Cypriniformes	Cyprinidae
BC095564/ENSDARP00000063532	Aqp12	Zebrafish	*Danio rerio*	Ostariophysi	Cypriniformes	Cyprinidae
APWO01096620	Aqp12	Mexican tetra	*Astyanax mexicanus*	Ostariophysi	Characiformes	Characidae
CK408292	Aqp12	Blue catfish	*Ictalurus furcatus*	Ostariophysi	Siluriformes	Ictaluridae
FD343108	Aqp12	Channel catfish	*Ictalurus punctatus*	Ostariophysi	Siluriformes	Ictaluridae
GAGX01084582/GAGX01029051/GAGX01074097	Aqp12	Brown bullhead	*Ameiurus nebulosus*	Ostariophysi	Siluriformes	Ictaluridae
AVPY01366016	Aqp12	Japanese eel	*Anguilla japonica*	Elopomorpha	Anguilliformes	Anguillidae
AHAT01015202	Aqp12	Spotted gar	*Lepisosteus oculatus*	Holostei	Semionotiformes	Lepisosteidae
AESE011498082/AESE010748237/AESE011637899	Aqp12	Little skate	*Leucoraja erinacea*	Chondrichthyes	Rajiformes	Rajidae
AAVX02000347	Aqp12	Ghost shark	*Callorhinchus milii*	Chondrichthyes	Chimaeriformes	Callorhinchidae
APJL01034632/APJL01136199/APJL01075977	Aqp12L	Arctic lamprey	*Lethenteron camtschaticum*	Hyperoartia	Petromyzontiformes	Petromyzontidae

XP_002592039 /ABEP02030099	Aqp12L1	Florida lancelet	*Branchiostoma floridae*	Cephalochordata	Amphioxiformes	Branchiostomidae
XP_002739302	Aqp12L1	Acorn worm	*Saccoglossus kowalevskii*	Hemichordata	Enteropneusta	Harrimaniidae
XP_002592040/ABEP02030099	Aqp12L2	Florida lancelet	*Branchiostoma floridae*	Cephalochordata	Amphioxiformes	Branchiostomidae
XP_002741885	Aqp12L2	Acorn worm	*Saccoglossus kowalevskii*	Hemichordata	Enteropneusta	Harrimaniidae
XP_780933/SPU_005585-tr	Aqp12L1	Purple sea urchin	*Strongylocentrotus purpuratus*	Echinodermata	Echinozoa	Strongylocentrotida
GAPB01023608	Aqp12L1	Kina	*Evechinus chloroticus*	Echinodermata	Echinozoa	Echinometridae
AGCV01082820	Aqp12L1	Green sea urchin	*Lytechinus variegatus*	Echinodermata	Echinozoa	Toxopneustidae
AKZP01011511	Aqp12L1	Bat star	*Patiria miniata*	Echinodermata	Asterozoa	Asterinidae
JT108126/SPU_005947-tr	Aqp12L2	Purple sea urchin	*Strongylocentrotus purpuratus*	Echinodermata	Echinozoa	Strongylocentrotida
GAPB01014568	Aqp12L2	Kina	*Evechinus chloroticus*	Echinodermata	Echinozoa	Echinometridae
AGCV01019958	Aqp12L2	Green sea urchin	*Lytechinus variegatus*	Echinodermata	Echinozoa	Toxopneustidae
AKZP01028600	Aqp12L2	Bat star	*Patiria miniata*	Echinodermata	Echinozoa	Asterinidae
**Radiata Aqp12 orthologs**						
BACK01037703	Aqp12	Stony coral	*Acropora digitifera*	Cnidaria	Scleractinia	Acroporidae
JT003875	Aqp12	Stony coral	*Acropora millepora*	Cnidaria	Scleractinia	Acroporidae
GASU01017125	Aqp12	Stony coral	*Acropora cervicornis*	Cnidaria	Scleractinia	Acroporidae
DR983976/DR983975	Aqp12	Stony coral	*Acropora palmata*	Cnidaria	Scleractinia	Acroporidae
FX461932	Aqp12	Stony coral	*Porites australiensis*	Cnidaria	Scleractinia	Acroporidae
XP_001647522	Aqp12	Starlet sea anemone	*Nematostella vectensis*	Cnidaria	Actiniaria	Edwardsiidae
ACZU01104846	Aqp12	Freshwater anemone	*Hydra vulgaris*	Cnidaria	Hydroida	Hydridae
FP957939	Aqp12	Thecate hydroid	*Clytia hemisphaerica*	Cnidaria	Hydroida	Campanulariidae
**AQP13**						
ENSOANP00000015441/XP_001520638	AQP13	Platypus	*Ornithorhynchus anatinus*	Prototheria	Monotremata	Ornithorhynchidae
NP_001082310/AAM81580	AQP13	African clawed frog	*Xenopus laevis*	Amphibia	Anura	Pipidae
ENSXETP00000035583/AAMC02034374	AQP13	Western clawed frog	*Xenopus (Silurana) tropicalis*	Amphibia	Anura	Pipidae
GAEI01021410/GAEI01008988	AQP13	Pacific treefrog	*Pseudacris regilla*	Amphibia	Anura	Hylidae
GAEG01041111/GAEG01028508	AQP13	Green frog	*Rana clamitans*	Amphibia	Anura	Ranidae
FS313448	AQP13	Japanese firebelly newt	*Cynops pyrrhogaster*	Amphibia	Caudata	Salamandridae
GAQK01049134	AQP13	Chinese salamander	*Hynobius chinensis*	Amphibia	Caudata	Hynobiidae
JV201948	AQP13	Axolotl	*Ambystoma mexicanum*	Amphibia	Caudata	Ambystomatidae
**AQP14**						
ABRP01010038	AQP14	Large flying fox/Megabat	*Pteropus vampyrus*	Laurasiatheria	Chiroptera	Pteropodidae
AWHB01448805	AQP14	Indian false vampire	*Megaderma lyra*	Laurasiatheria	Chiroptera	Megadermatidae
ACIV010529105	AQP14	Sheep	*Ovis aries*	Laurasiatheria	Ruminantia	Bovidae
AJPT01056746	AQP14	Goat	*Capra hircus*	Laurasiatheria	Ruminantia	Bovidae
AGTT01249570	AQP14	Chiru	*Pantholops hodgsonii*	Laurasiatheria	Ruminantia	Bovidae
AAFC03087741	AQP14	Cow	*Bos taurus*	Laurasiatheria	Ruminantia	Bovidae
AGSK01134320	AQP14	Yak	*Bos mutus*	Laurasiatheria	Ruminantia	Bovidae
AGFL01047914	AQP14	Zebu	*Bos indicus*	Laurasiatheria	Ruminantia	Bovidae
AWWX01580831	AQP14	Water buffalo	*Bubalus bubalis*	Laurasiatheria	Ruminantia	Bovidae
ABRN02322768	AQP14	Bottlenosed dolphin	*Tursiops truncatus*	Laurasiatheria	Cetacea	Delphinidae
ANOL02036785	AQP14	Killer whale	*Orcinus orca*	Laurasiatheria	Cetacea	Delphinidae
AUPI01131496	AQP14	Yangtze River dolphin	*Lipotes vexillifer*	Laurasiatheria	Cetacea	Lipotidae
AWZP01107178	AQP14	Sperm whale	*Physeter catodon*	Laurasiatheria	Cetacea	Physeteridae
ATDI01166774	AQP14	Minke whale	*Balaenoptera acutorostrata*	Laurasiatheria	Cetacea	Balaenopteridae
AEYP01080245	AQP14	Domestic ferret	*Mustela putorius furo*	Laurasiatheria	Carnivora	Mustelidae
APMU01097947	AQP14	Weddell seal	*Leptonychotes weddellii*	Laurasiatheria	Carnivora	Phocidae
ABVD01460925	AQP14	Hoffmann's two-fingered sloth	*Choloepus hoffmanni*	Xenarthra	Pilosa	Megalonychidae
ABQO020460914	AQP14	Tammar wallaby	*Macropus eugenii*	Metatheria	Diprotodontia	Macropodidae
AFEY01397709	AQP14	Tasmanian devil	*Sarcophilus harrisii*	Metatheria	Dasyuromorphia	Dasyuridae
AAPN01303265	AQP14	Platypus	*Ornithorhynchus anatinus*	Prototheria	Monotremata	Ornithorhynchidae
GENSCAN00000046252	AQP14	Zebra finch	*Taeniopygia guttata*	Aves	Passeriformes	Estrildidae
ANZD01003873	AQP14	Tibetan ground-tit	*Pseudopodoces humilis*	Aves	Passeriformes	Paridae
AGTO02006564	AQP14	Collared flycatcher	*Ficedula albicollis*	Aves	Passeriformes	Muscicapidae
AKMT01045875	AQP14	Peregrin falcon	*Falco peregrinus*	Aves	Falconiformes	Falconidae
AKMU01048960	AQP14	Saker falcon	*Falco cherrug*	Aves	Falconiformes	Falconidae
ADDD01152144	AQP14	Turkey	*Meleagris gallopavo*	Aves	Galliformes	Phasianidae
AADN03015245/ENSGALP00000041082	AQP14	Chicken	*Gallus gallus*	Aves	Galliformes	Phasianidae
AKHW01052381	AQP14	American alligator	*Alligator mississippiensis*	Archosauria	Crocodylia	Alligatoridae
AVPB01160522	AQP14	Chinese alligator	*Alligator sinensis*	Archosauria	Crocodylia	Alligatoridae
AGCU01140840/AGCU01140841/ GENSCAN00000013674	AQP14	Chinese softshell turtle	*Pelodiscus sinensis*	Sauropsida	Testudines	Trionychidae
AJIM01220996	AQP14	Green seaturtle	*Chelonia mydas*	Sauropsida	Testudines	Cheloniidae
JW499633	AQP14	Red-eared slider turtle	*Trachemys scripta elegans*	Sauropsida	Testudines	Emydidae
AHGY01503377	AQP14	Western painted turtle	*Chrysemys picta bellii*	Sauropsida	Testudines	Emydidae
AZIM01000024	AQP14	King cobra	*Ophiophagus hannah*	Lepidosauria	Squamata	Elapidae
AEQU02069337	AQP14	Burmese python	*Python molurus bivittatus*	Lepidosauria	Squamata	Pythonidae
AAWZ02009718/ENSACAP00000023015	AQP14	Green anole	*Anolis carolinensis*	Lepidosauria	Squamata	Iguanidae
AAMC02007443/ENSXETP00000060652	AQP14	Western clawed frog	*Xenopus (Silurana) tropicalis*	Amphibia	Anura	Pipidae
BAHO01111606/BAHO01111607/ ENSLACP00000021646	Aqp14	Coelacanth	*Latimeria chalumnae*	Actinistia	Coelacanthiformes	Coelacanthidae
GENSCAN00000009007	Aqp14	Torafugu	*Takifugu rubripes*	Acanthopterygii	Tetraodontiformes	Tetraodontidae

AOOT01015864	Aqp14	Sansaifugu	*Takifugu flavidus*	Acanthopterygii	Tetraodontiformes	Tetraodontidae
CAAE01014729	Aqp14	Green-spotted pufferfish	*Tetraodon nigroviridis*	Acanthopterygii	Tetraodontiformes	Tetraodontidae
AGRG01001236/AGRG01001237	Aqp14	Tongue sole	*Cynoglossus semilaevis*	Acanthopterygii	Pleuronectiformes	Cynoglossidae
AERX01002680/ENSONIP00000021523	Aqp14	Nile tilapia	*Oreochromis niloticus*	Acanthopterygii	Perciformes	Cichlidae
AFNZ01014050/AFNZ01014051	Aqp14	Burton's mouthbrooder	*Haplochromis burtoni*	Acanthopterygii	Perciformes	Cichlidae
AGTA02010417	Aqp14	Zebra mbuna	*Maylandia zebra*	Acanthopterygii	Perciformes	Cichlidae
AFNX01016365	Aqp14	Red mwanza	*Pundamilia nyererei*	Acanthopterygii	Perciformes	Cichlidae
AFNY01042433	Aqp14	Lyretail cichlid	*Neolamprologus brichardi*	Acanthopterygii	Perciformes	Cichlidae
BADN01090219	Aqp14	Pacific bluefin tuna	*Thunnus orientalis*	Acanthopterygii	Perciformes	Scombridae
AWGY01125078	Aqp14	Sablefish	*Anoplopoma fimbria*	Acanthopterygii	Scorpaeniformes	Anoplopomatidae
AUPQ01088341	Aqp14	Flag rockfish	*Sebastes rubrivinctus*	Acanthopterygii	Scorpaeniformes	Sebastidae
AUPR01097278	Aqp14	Tiger rockfish	*Sebastes nigrocinctus*	Acanthopterygii	Scorpaeniformes	Sebastidae
AANH01010881	Aqp14	Three-spined stickleback	*Gasterosteus aculeatus*	Acanthopterygii	Gasterosteiformes	Gasterosteidae
GENSCAN00000045613	Aqp14	Japanese medaka	*Oryzias latipes*	Acanthopterygii	Beloniformes	Adrianichthyidae
GAIB01092986	Aqp14	Turquoise killifish	*Nothobranchius furzeri*	Acanthopterygii	Cyprinodontiformes	Nothobranchiidae
AYCK01011345	Aqp14	Amazon molly	*Poecilia formosa*	Acanthopterygii	Cyprinodontiformes	Poeciliidae
AGAJ01011673/ENSXMAP00000017491	Aqp14	Southern platyfish	*Xiphophorus maculatus*	Acanthopterygii	Cyprinodontiformes	Poeciliidae
CAEA01036752/GENSCAN00000078012/ GENSCAN00000074903	Aqp14	Atlantic cod	*Gadus morhua*	Paracanthopterygii	Gadiformes	Gadidae
CCAF010085061	Aqp14-1	Rainbow trout	*Oncorhynchus mykiss*	Protacanthopterygii	Salmoniformes	Salmonidae
CCAF010053309	Aqp14-2	Rainbow trout	*Oncorhynchus mykiss*	Protacanthopterygii	Salmoniformes	Salmonidae
AGKD01272622/AGKD01346587	Aqp14-2	Atlantic salmon	*Salmo salar*	Protacanthopterygii	Salmoniformes	Salmonidae
GAHO01028309	Aqp14	Small gill opening goldenline barbel	*Sinocyclocheilus angustiporus*	Ostariophysi	Cypriniformes	Cyprinidae
GAHL01139706	Aqp14	Blind goldenline barbel	*Sinocyclocheilus anophthalmus*	Ostariophysi	Cypriniformes	Cyprinidae
CABZ01016213	Aqp14	Zebrafish	*Danio rerio*	Ostariophysi	Cypriniformes	Cyprinidae
AVPY01025849/AVPY01025850	Aqp14	Japanese eel	*Anguilla japonica*	Elopomorpha	Anguilliformes	Anguillidae
AHAT01018940/AHAT01018941	Aqp14	Spotted gar	*Lepisosteus oculatus*	Holostei	Semionotiformes	Lepisosteidae
AESE011117157/AESE011019981/AESE011617837 /AESE010654055	Aqp14	Little skate	*Leucoraja erinacea*	Chondrichthyes	Rajiformes	Rajidae
AAVX02050002	Aqp14	Ghost shark	*Callorhinchus milii*	Chondrichthyes	Chimaeriformes	Callorhinchidae
AEFG01022836/AEFG01022837/ ENSPMAP00000004935	Aqp14	Sea lamprey	*Petromyzon marinus*	Hyperoartia	Petromyzontiformes	Petromyzontidae
APJL01031881	Aqp14	Arctic lamprey	*Lethenteron camtschaticum*	Hyperoartia	Petromyzontiformes	Petromyzontidae

AQP15

AKHW01056389	AQP15	American alligator	*Alligator mississippiensis*	Archosauria	Crocodylia	Alligatoridae
AVPB01041626/AVPB01041625	AQP15	Chinese alligator	*Alligator sinensis*	Archosauria	Crocodylia	Alligatoridae
AGCU01111242/ENSPSIP00000010966	AQP15	Chinese softshell turtle	*Pelodiscus sinensis*	Sauropsida	Testudines	Trionychidae
APJP01651563/APJP01436353/APJP01730811	AQP15	Spiny softshell turtle	*Apalone spinifera*	Sauropsida	Testudines	Trionychidae
AJIM01202371/AJIM01202367/AJIM01202366	AQP15	Green seaturtle	*Chelonia mydas*	Sauropsida	Testudines	Cheloniidae
JW437790	AQP15	Red-eared slider turtle	*Trachemys scripta elegans*	Sauropsida	Testudines	Emydidae
AHGY01269025/AHGY01269019/AHGY01269017	AQP15	Western painted turtle	*Chrysemys picta bellii*	Sauropsida	Testudines	Emydidae
AFYH01058142/AFYH01058143	Aqp15	Coelacanth	*Latimeria chalumnae*	Actinistia	Coelacanthiformes	Coelacanthidae
XP_003442458	Aqp15	Nile tilapia	*Oreochromis niloticus*	Acanthopterygii	Perciformes	Cichlidae
GAIB01108029	Aqp15	Turquoise killifish	*Nothobranchius furzeri*	Acanthopterygii	Cyprinodontiformes	Nothobranchiidae
AYCK01010468	Aqp15	Amazon molly	*Poecilia formosa*	Acanthopterygii	Cyprinodontiformes	Poeciliidae
ENSXMAP00000016987/XP_005817244	Aqp15	Southern platyfish	*Xiphophorus maculatus*	Acanthopterygii	Cyprinodontiformes	Poeciliidae
CCAF010016359	Aqp15-1	Rainbow trout	*Oncorhynchus mykiss*	Protacanthopterygii	Salmoniformes	Salmonidae
AGKD01027288	Aqp15-1	Atlantic salmon	*Salmo salar*	Protacanthopterygii	Salmoniformes	Salmonidae
AGKD01030871/AGKD01079866	Aqp15-2	Atlantic salmon	*Salmo salar*	Protacanthopterygii	Salmoniformes	Salmonidae
CABZ01067344/ENSDARP00000055694	Aqp15	Zebrafish	*Danio rerio*	Ostariophysi	Cypriniformes	Cyprinidae
APWO01044624/APWO01044625/APWO01044626	Aqp15	Mexican tetra	*Astyanax mexicanus*	Ostariophysi	Characiformes	Characidae
AVPY01684432/AVPY01621650/AVPY01386739/ AVPY01669299/AVPY01386736	Aqp15	Japanese eel	*Anguilla japonica*	Elopomorpha	Anguilliformes	Anguillidae
AHAT01031086	Aqp15	Spotted gar	*Lepisosteus oculatus*	Holostei	Semionotiformes	Lepisosteidae
KJ815007	Aqp15	Spiny dogfish	*Squalus acanthias*	Chondrichthyes	Squaliformes	Squalidae
AESE010364165/AESE011605739/AESE010206814 /AESE010090626	Aqp15	Little skate	*Leucoraja erinacea*	Chondrichthyes	Rajiformes	Rajidae
AAVX02031965/AAVX02031964	Aqp15	Ghost shark	*Callorhinchus milii*	Chondrichthyes	Chimaeriformes	Callorhinchidae

AQP16

AKHW01050368	AQP16	American alligator	*Alligator mississippiensis*	Archosauria	Crocodylia	Alligatoridae
AVPB01142836	AQP16	Chinese alligator	*Alligator sinensis*	Archosauria	Crocodylia	Alligatoridae
AJIM01273747	AQP16	Green seaturtle	*Chelonia mydas*	Sauropsida	Testudines	Cheloniidae
AHGY01502013/AHGY01502015	AQP16	Western painted turtle	*Chrysemys picta bellii*	Sauropsida	Testudines	Emydidae
AAH99368/NP_001089643	AQP16	African clawed frog	*Xenopus laevis*	Amphibia	Anura	Pipidae
GENSCAN00000004031	AQP16	Western clawed frog	*Xenopus (Silurana) tropicalis*	Amphibia	Anura	Pipidae
GAQK01103769	AQP16	Chinese salamander	Hynobius chinensis	Amphibia	Caudata	Hynobiidae

Glp

KJ815008	Glp	Atlantic hagfish	*Myxine glutinosa*	Hyperotreti	Myxiniformes	Myxinidae
ENSCINP00000019491	Glp	Vase tunicate	*Ciona intestinalis*	Tunicata	Enterogona	Cionidae
SINCSAVP00000010319	Glp	Pacific transparent sea squirt	*Ciona savignyi*	Tunicata	Enterogona	Cionidae
XP_002604987	Glp	Florida lancelet	*Branchiostoma floridae*	Cephalochordata	Amphioxiformes	Branchiostomidae
XP_002609960	Glp	Florida lancelet	*Branchiostoma floridae*	Cephalochordata	Amphioxiformes	Branchiostomidae
FN991824	Glp	Pterobranch	*Rhabdopleura compacta*	Hemichordata	Rhabdopleurida	Rhabdopleuridae

XP_002736840	Glp	Acorn worm	*Saccoglossus kowalevskii*	Hemichordata	Enteropneusta	Harrimaniidae
AAGJ04123740/AAGJ04123724/AAGJ04123722/ XP_786125/SPU_024384_tr	Glp 3	Purple sea urchin	*Strongylocentrotus purpuratus*	Echinodermata	Echinozoa	Strongylocentrotida
GAPB01003116	Glp 3	Kina	*Evechinus chloroticus*	Echinodermata	Echinozoa	Echinometridae
AGCV01322870/AGCV01012909/AGCV01012912	Glp 3	Green sea urchin	*Lytechinus variegatus*	Echinodermata	Echinozoa	Toxopneustidae
AKZP01133424/AKZP01133423	Glp 3	Bat star	*Patiria miniata*	Echinodermata	Asterozoa	Asterinidae
AAGJ04114907/XP_789770/SPU_004511_tr	Glp 2	Purple sea urchin	*Strongylocentrotus purpuratus*	Echinodermata	Echinozoa	Strongylocentrotida
GAPB01012573	Glp 2	Kina	*Evechinus chloroticus*	Echinodermata	Echinozoa	Echinometridae
AGCV01043128	Glp 2	Green sea urchin	*Lytechinus variegatus*	Echinodermata	Echinozoa	Toxopneustidae
AKZP01130689/AKZP01138585	Glp 2	Bat star	*Patiria miniata*	Echinodermata	Asterozoa	Asterinidae
AAGJ04134789/AAGJ04134789/XP_796513	Glp 1b	Purple sea urchin	*Strongylocentrotus purpuratus*	Echinodermata	Echinozoa	Strongylocentrotida
AAGJ04062807/AAGJ04062804/AAGJ04062803/ AAGJ04062801/SPU_023979_tr	Glp 1	Purple sea urchin	*Strongylocentrotus purpuratus*	Echinodermata	Echinozoa	Strongylocentrotida
GAPB01053568	Glp 1	Kina	*Evechinus chloroticus*	Echinodermata	Echinozoa	Echinometridae
AGCV01341974/AGCV01341972/AGCV01341971/ AGCV01395938/AGCV01395937	Glp 1	Green sea urchin	*Lytechinus variegatus*	Echinodermata	Echinozoa	Toxopneustidae
AKZP01128013/AKZP01128014/AKZP01038911	Glp 1a	Bat star	*Patiria miniata*	Echinodermata	Asterozoa	Asterinidae
AKZP01008734	Glp 1b	Bat star	*Patiria miniata*	Echinodermata	Asterozoa	Asterinidae
AKZP01008737/AKZP01008738	Glp 1c	Bat star	*Patiria miniata*	Echinodermata	Asterozoa	Asterinidae
XP_001637091	Glp	Starlet sea anemone	*Nematostella vectensis*	Cnidaria	Actiniaria	Edwardsiidae
XP_002156497	Glp	Hydra	*Hydra vulgaris*	Cnidaria	Hydroida	Hydridae
XP_003383528/PAC_15726348	Glp	Sponge	*Amphimedon queenslandica*	Porifera	Haplosclerida	Niphatidae
**Parazoa-Radiata Glp**						
BACK01014751/BACK01014752	Glp 1	Stony coral	*Acropora digitifera*	Cnidaria	Scleractinia	Acroporidae
JR999939	Glp 1	Stony coral	*Acropora millepora*	Cnidaria	Scleractinia	Acroporidae
DC999942	Glp 1	Stony coral	*Acropora tenuis*	Cnidaria	Scleractinia	Acroporidae
GASU01015432	Glp 1	Stony coral	*Acropora cervicornis*	Cnidaria	Scleractinia	Acroporidae
GW195723	Glp 1	Stony coral	*Acropora palmata*	Cnidaria	Scleractinia	Acroporidae
GARY01000511	Glp 1	Stony coral	*Stylophora pistillata*	Cnidaria	Scleractinia	Pocilloporidae
BACK01014753/BACK01014752	Glp 1b	Stony coral	*Acropora digitifera*	Cnidaria	Scleractinia	Acroporidae
JR980480	Glp 1b	Stony coral	*Acropora millepora*	Cnidaria	Scleractinia	Acroporidae
GASU01059074	Glp 1b	Stony coral	*Acropora cervicornis*	Cnidaria	Scleractinia	Acroporidae
FX438739	Glp 1b	Stony coral	*Porites australiensis*	Cnidaria	Scleractinia	Poritidae
GE909075	Glp 1b	Stony coral	*Porites astreoides*	Cnidaria	Scleractinia	Poritidae
GARY01000511	Glp 1b	Stony coral	*Stylophora pistillata*	Cnidaria	Scleractinia	Pocilloporidae
BACK01014751	Glp 2	Stony coral	*Acropora digitifera*	Cnidaria	Scleractinia	Acroporidae
JR978591	Glp 2	Stony coral	*Acropora millepora*	Cnidaria	Scleractinia	Acroporidae
GASU01092011	Glp 2	Stony coral	*Acropora cervicornis*	Cnidaria	Scleractinia	Acroporidae
GW270188	Glp 2	Stony coral	*Montastraea faveolata*	Cnidaria	Scleractinia	Montastraeidae
FX459378	Glp 2	Stony coral	*Porites australiensis*	Cnidaria	Scleractinia	Poritidae
GARY01025768	Glp 2	Stony coral	*Stylophora pistillata*	Cnidaria	Scleractinia	Pocilloporidae
v1g162785/XM_001637091	Glp 2a	Starlet sea anemone	*Nematostella vectensis*	Cnidaria	Actiniaria	Edwardsiidae
v1g93479/ XP_001637090	Glp 2b	Starlet sea anemone	*Nematostella vectensis*	Cnidaria	Actiniaria	Edwardsiidae
v1g4182/XP_001620982	Glp 2c	Starlet sea anemone	*Nematostella vectensis*	Cnidaria	Actiniaria	Edwardsiidae
v1g243020/XP_001633058	Glp 2d	Starlet sea anemone	*Nematostella vectensis*	Cnidaria	Actiniaria	Edwardsiidae
JV099621	Glp 2a	Symbiotic anemone	*Aiptasia pallida*	Cnidaria	Actiniaria	Aiptasiidae
JV119766	Glp 2b	Symbiotic anemone	*Aiptasia pallida*	Cnidaria	Actiniaria	Aiptasiidae
JV113575	Glp 2c	Symbiotic anemone	*Aiptasia pallida*	Cnidaria	Actiniaria	Aiptasiidae
XP_002162049	Glp 1a	Freshwater anemone	*Hydra vulgaris*	Cnidaria	Hydroida	Hydridae
XP_002165391	Glp 1b	Freshwater anemone	*Hydra vulgaris*	Cnidaria	Hydroida	Hydridae
XP_002165440	Glp 1c	Freshwater anemone	*Hydra vulgaris*	Cnidaria	Hydroida	Hydridae
FP974057	Glp 1	Thecate hydroid	*Clytia hemisphaerica*	Cnidaria	Hydroida	Campanulariidae
XP_002156497	Glp 2	Freshwater anemone	*Hydra vulgaris*	Cnidaria	Hydroida	Hydridae
CU427936	Glp 2	Thecate hydroid	*Clytia hemisphaerica*	Cnidaria	Hydroida	Campanulariidae
XP_002108654	Glp	Placozoan	*Trichoplax adhaerens*	Placazoa		
Aqu1_227820/XP_003383528/PAC_15726348/ ACUQ01000663	Glp 1	Sea sponge	*Amphimedon queenslandica*	Porifera	Haplosclerida	Niphatidae
AM760644	Glp 1	Mueller's freshwater sponge	*Ephydatia muelleri*	Porifera	Haplosclerida	Spongillidae
CAK22281	Glp 1	Lake Baikal sponge	*Lubomirskia baicalensis*	Porifera	Haplosclerida	Lubomirskiidae
GO086050	Glp 1a	Sponge	*Heterochone calyx*	Porifera	Hexactinosida	Aphrocallistidae
GO087389	Glp 1b	Sponge	*Heterochone calyx*	Porifera	Hexactinosida	Aphrocallistidae
Aqu1_227913/PAC_15726441/XP_003383384/ ACUQ01000619	Glp 1a	Sea sponge	*Amphimedon queenslandica*	Porifera	Haplosclerida	Niphatidae
PAC_1571972Aqu1_221200/8/XP_003386535/ ACUQ01003032	Glp 1b	Sea sponge	*Amphimedon queenslandica*	Porifera	Haplosclerida	Niphatidae
PAC_15719729/ACUQ01003032	Glp 1c	Sea sponge	*Amphimedon queenslandica*	Porifera	Haplosclerida	Niphatidae
ETX05898	Glp F	Sea sponge symbiont	*Candidatus Entotheonella sp.*	Proteobacteria		
ETW98335	Glp F	Sea sponge symbiont	*Candidatus Entotheonella sp.*	Proteobacteria		
**Protists-Fungi Glp**						
DDB0167571	Glp 1	Cellular slime mold	*Dictyostelium discoideum*	Amoebozoa	Dictyosteliida	
AJWI01003455	Glp 1	Cellular slime mold	*Dictyostelium intermedium*	Amoebozoa	Dictyosteliida	
XP_004350034	Glp 1	Cellular slime mold	*Dictyostelium fasciculatum*	Amoebozoa	Dictyosteliida	
DDB0189011	Glp 2	Cellular slime mold	*Dictyostelium discoideum*	Amoebozoa	Dictyosteliida	
AJWI01002093	Glp 2	Cellular slime mold	*Dictyostelium intermedium*	Amoebozoa	Dictyosteliida	
XP_003294465	Glp 2	Cellular slime mold	*Dictyostelium purpureum*	Amoebozoa	Dictyosteliida	

XP_001615442/PVX_092245	Glp	Plasmodium parasite	*Plasmodium vivax*	Apicomplexa	Haemosporida	
PKH_093060	Glp	Plasmodium parasite	*Plasmodium knowlesi*	Apicomplexa	Haemosporida	
PF11_0338-1	Glp	Plasmodium parasite	*Plasmodium falciparum*	Apicomplexa	Haemosporida	
Phatr44871	Glp	Diatom	*Phaeodactylum tricornutum*	Bacillariophyta	Naviculales	Phaeodactylaceae
Phatrdraft1692	Glp	Diatom	*Phaeodactylum tricornutum*	Bacillariophyta	Naviculales	Phaeodactylaceae
EDP47128	Glp	Fungus	*Aspergillus fumigatus*	Ascomycota	Eurotiales	Aspergillaceae
XP_001267577	Glp	Fungus	*Neosartorya fischeri*	Ascomycota	Eurotiales	Aspergillaceae
XP_001270514	Glp	Fungus	*Aspergillus clavatus*	Ascomycota	Eurotiales	Aspergillaceae
CAP99447	Glp	Fungus	*Penicillium chrysogenum Wisconsin*	Ascomycota	Eurotiales	Aspergillaceae
YFL054C	Glp	Baker´s yeast	*Saccharomyces cerevisiae*	Ascomycota	Saccharomycetales	Saccharomycetacea
NP_592788	Glp	Fungus	*Schizosaccharomyces pombe*	Ascomycota	Saccharomycetales	Saccharomycetacea
**Bacteria**						
AAC43518/EBESCP00000004569	Aqp Z	Bacteria	*Escherichia coli*	Proteobacteria	Enterobacteriales	Enterobacteriaceae
WP_005067324/EBESCP00000088383	Aqp Z	Bacteria	*Shigella flexneri*	Proteobacteria	Enterobacteriales	Enterobacteriaceae
YP_403953/EBESCP00000078866	Aqp Z	Bacteria	*Shigella dysenteriae*	Proteobacteria	Enterobacteriales	Enterobacteriaceae
YP_890679/EBMYCP00000044909	Aqp Z	Bacteria	*Mycobacterium smegmatis*	Actinobacteria	Actinomycetales	Mycobacteriaceae
YP_005000292	Aqp Z	Bacteria	*Mycobacterium rhodesiae*	Actinobacteria	Actinomycetales	Mycobacteriaceae
YP_006455412	Aqp Z	Bacteria	*Mycobacterium chubuense*	Actinobacteria	Actinomycetales	Mycobacteriaceae
YP_004669618	Aqp Z	Bacteria	*Myxococcus fulvus*	Proteobacteria	Myxococcales	Myxococcaceae
NP_359196/EBSTRP00000015353	Aqp Z	Bacteria	*Streptococcus pneumoniae*	Firmicutes	Lactobacillales	Streptococcaceae
WP_006154600	Aqp Z	Bacteria	*Streptococcus infantis*	Firmicutes	Lactobacillales	Streptococcaceae
WP_006595935	Aqp Z	Bacteria	*Streptococcus australis*	Firmicutes	Lactobacillales	Streptococcaceae
EFU62379	Aqp Z	Bacteria	*Streptococcus oralis*	Firmicutes	Lactobacillales	Streptococcaceae
YP_005004169	Aqp Z	Bacteria	*Pediococcus claussenii*	Firmicutes	Lactobacillales	Lactobacillaceae
WP_024002254	Aqp Z	Bacteria	*Lactobacillus plantarum*	Firmicutes	Lactobacillales	Lactobacillaceae
WP_023439950	Aqp Z	Bacteria	*Pediococcus pentosaceus*	Firmicutes	Bacillales	Lactobacillaceae
YP_002340560/EBBACP00000092199	Aqp Z	Bacteria	*Bacillus cereus*	Firmicutes	Bacillales	Bacillaceae
YP_038558/EBBACP00000072619	Aqp Z	Bacteria	*Bacillus thuringiensis*	Firmicutes	Bacillales	Bacillaceae
WP_003200733	Aqp Z	Bacteria	*Bacillus mycoides*	Firmicutes	Bacillales	Bacillaceae
WP_010710463	Aqp Z	Bacteria	*Enterococcus faecalis*	Firmicutes	Lactobacillales	Enterococcaceae
YP_302578	Aqp Z	Bacteria	*Staphylococcus saprophyticus subsp.*	Firmicutes	Bacillales	Staphylococcaceae
WP_024029819	Aqp N	Bacteria	*Bacillus vireti*	Firmicutes	Bacillales	Bacillaceae
WP_004433890	Aqp N	Bacteria	*Bacillus methanolicus*	Firmicutes	Bacillales	Bacillaceae
NP_982172	Aqp N	Bacteria	*Bacillus cereus*	Firmicutes	Bacillales	Bacillaceae
WP_007474939	Aqp N	Bacteria	*Caminibacter mediatlanticus*	Proteobacteria	Nautiliales	Nautiliaceae
ETZ22260	Aqp N	Bacteria	*Pedobacter sp.*	Bacteroidetes	Sphingobacteriales	Sphingobacteriaceae
WP_017259290	Aqp N	Bacteria	*Pedobacter arcticus*	Bacteroidetes	Sphingobacteriales	Sphingobacteriaceae
WP_008271191	Aqp N	Bacteria	*Flavobacteriales bacterium*	Bacteroidetes	Flavobacteriales	
YP_006419200	Aqp N	Bacteria	*Aequorivita sublithincola*	Bacteroidetes	Flavobacteriales	Flavobacteriaceae
WP_002696154	Aqp N	Bacteria	*Microscilla marina*	Bacteroidetes	Cytophagales	Cytophagaceae
WP_007910121	Aqp N1	Bacteria	*Ktedonobacter racemifer*	Chloroflexi	Ktedonobacterales	Ktedonobacteraceae
WP_007911570	Aqp N2	Bacteria	*Ktedonobacter racemifer*	Chloroflexi	Ktedonobacterales	Ktedonobacteraceae
WP_008479677	Aqp N	Bacteria	*Nitrolancea hollandica*	Chloroflexi	Sphaerobacterales	Sphaerobacteraceae
YP_886482/EBMYCP00000043363	Aqp M	Bacteria	*Mycobacterium smegmatis*	Actinobacteria	Actinomycetales	Mycobacteriaceae
WP_020385508	Aqp M	Bacteria	*Kribbella catacumbae*	Actinobacteria	Actinomycetales	Propionibacterineae
WP_009773038	Aqp M	Bacteria	*marine actinobacterium*	Actinobacteria		
YP_004495611	Aqp M	Bacteria	*Amycolicicoccus subflavus*	Actinobacteria	Actinomycetales	Mycobacteriaceae
YP_003111867	Aqp M	Bacteria	*Catenulispora acidiphila*	Actinobacteria	Actinomycetales	Catenulisporaceae
WP_023549603	Aqp M	Bacteria	*Streptomyces roseochromogenes*	Actinobacteria	Actinomycetales	Streptomycetaceae
WP_010280944	Aqp M	Bacteria	*Bacillus timonensis*	Firmicutes	Bacillales	Bacillaceae
WP_010144684	Aqp M	Bacteria	*Citricoccus* sp.	Actinobacteria	Actinomycetales	Micrococcaceae
WP_005268377	Aqp M	Bacteria	*Arthrobacter crystallopoietes*	Actinobacteria	Actinomycetales	Micrococcaceae
YP_004100076	Aqp M	Bacteria	*Intrasporangium calvum*	Actinobacteria	Actinomycetales	Intrasporangiaceae
WP_010834136	Aqp M	Bacteria	*Nocardioides* sp.	Actinobacteria	Actinomycetales	Nocardioidaceae
NP_290556/EBESCP00000004421	Glp F	Bacteria	*Escherichia coli*	Proteobacteria	Enterobacteriales	Enterobacteriaceae
WP_000084271/EBESCP00000089272	Glp F	Bacteria	*Shigella flexneri*	Proteobacteria	Enterobacteriales	Enterobacteriaceae
YP_405253/EBESCP00000079777	Glp F	Bacteria	*Shigella dysenteriae*	Proteobacteria	Enterobacteriales	Enterobacteriaceae
YP_890966/EBMYCP00000044076	Glp F	Bacteria	*Mycobacterium smegmatis*	Actinobacteria	Actinomycetales	Mycobacteriaceae
WP_008355330	Glp F	Bacteria	*Nocardioidaceae bacterium*	Actinobacteria	Actinomycetales	Propionibacterineae
YP_003115825	Glp F	Bacteria	*Catenulispora acidiphila*	Actinobacteria	Actinomycetales	Catenulisporaceae
EXU67399	Glp F	Bacteria	*Streptomyces* sp.	Actinobacteria	Actinomycetales	Streptomycetaceae
YP_709683/EBBORP00000003629	Glp F	Bacteria	*Borrelia afzelii*	Spirochaetes	Spirochaetales	Spirochaetaceae
YP_006202933	Glp F	Bacteria	*Borrelia garinii*	Spirochaetes	Spirochaetales	Spirochaetaceae
YP_004777489	Glp F	Bacteria	*Borrelia bissettii*	Spirochaetes	Spirochaetales	Spirochaetaceae
YP_005005624	Glp F4	Bacteria	*Pediococcus claussenii*	Firmicutes	Lactobacillales	Lactobacillaceae
WP_022638082	Glp F4	Bacteria	*Lactobacillus plantarum*	Firmicutes	Lactobacillales	Lactobacillaceae
EKQ06312	Glp F4	Bacteria	*Lactobacillus casei*	Firmicutes	Lactobacillales	Lactobacillaceae
WP_010010194	Glp F4	Bacteria	*Lactobacillus coryniformis*	Firmicutes	Lactobacillales	Lactobacillaceae
WP_002896339	Glp F4	Bacteria	*Streptococcus sanguinis*	Firmicutes	Lactobacillales	Streptococcaceae
EGU64862	Glp F4	Bacteria	*Streptococcus australis*	Firmicutes	Lactobacillales	Streptococcaceae
NP_977447/EBBACP00000096005	Glp F	Bacteria	*Bacillus cereus*	Firmicutes	Bacillales	Bacillaceae
YP_035285/EBBACP00000073529	Glp F	Bacteria	*Bacillus thuringiensis*	Firmicutes	Bacillales	Bacillaceae

NP_388809/EBBACP00000003509	Glp F	Bacteria	*Bacillus subtilis subsp*	Firmicutes	Bacillales	Bacillaceae
YP_499808	Glp F	Bacteria	*Staphylococcus aureus*	Firmicutes	Bacillales	Staphylococcaceae
WP_002442630	Glp F	Bacteria	*Staphylococcus caprae*	Firmicutes	Bacillales	Staphylococcaceae
WP_002483419	Glp F	Bacteria	*Staphylococcus saprophyticus*	Firmicutes	Bacillales	Staphylococcaceae
WP_002832951	Glp F3	Bacteria	*Pediococcus pentosaceus*	Firmicutes	Lactobacillales	Lactobacillaceae
ERO40491	Glp F3	Bacteria	*Lactobacillus plantarum*	Firmicutes	Lactobacillales	Lactobacillaceae
WP_010011495	Glp F3	Bacteria	*Lactobacillus coryniformis*	Firmicutes	Lactobacillales	Lactobacillaceae
AGP73310	Glp F3a	Bacteria	*Lactobacillus casei*	Firmicutes	Lactobacillales	Lactobacillaceae
CCK21672	Glp F3	Bacteria	*Lactobacillus casei*	Firmicutes	Lactobacillales	Lactobacillaceae
YP_005708647	Glp F3	Bacteria	*Enterococcus faecalis*	Firmicutes	Lactobacillales	Enterococcaceae
EGU64844	Glp F3	Bacteria	*Streptococcus australis*	Firmicutes	Lactobacillales	Streptococcaceae
EGV13404	Glp F3	Bacteria	*Streptococcus infantis*	Firmicutes	Lactobacillales	Streptococcaceae
EFX94407	Glp F3	Bacteria	*Streptococcus sanguinis*	Firmicutes	Lactobacillales	Streptococcaceae
NP_359579/EBSTRP00000014534	Glp F3	Bacteria	*Streptococcus pneumoniae*	Firmicutes	Lactobacillales	Streptococcaceae
EGD30659	Glp F2	Bacteria	*Streptococcus sanguinis*	Firmicutes	Lactobacillales	Streptococcaceae
WP_000344873	Glp F2	Bacteria	*Streptococcus infantis*	Firmicutes	Lactobacillales	Streptococcaceae
WP_006596595	Glp F2	Bacteria	*Streptococcus australis*	Firmicutes	Lactobacillales	Streptococcaceae
YP_005004704	Glp F2	Bacteria	*Pediococcus claussenii*	Firmicutes	Lactobacillales	Lactobacillaceae
WP_002834367	Glp F2	Bacteria	*Pediococcus pentosaceus*	Firmicutes	Lactobacillales	Lactobacillaceae
ADK18029	Glp F2	Bacteria	*Lactobacillus casei*	Firmicutes	Lactobacillales	Lactobacillaceae
EFK28283	Glp F2	Bacteria	*Lactobacillus plantarum*	Firmicutes	Lactobacillales	Lactobacillaceae
YP_005005669	Glp F1	Bacteria	*Pediococcus claussenii*	Firmicutes	Lactobacillales	Lactobacillaceae
YP_805154	Glp F1	Bacteria	*Pediococcus pentosaceus*	Firmicutes	Lactobacillales	Lactobacillaceae
YP_005708605	Glp F1	Bacteria	*Enterococcus faecalis*	Firmicutes	Lactobacillales	Enterococcaceae
WP_003679683	Glp F1	Bacteria	*Lactobacillus coryniformis*	Firmicutes	Lactobacillales	Lactobacillaceae
AGL62833	Glp F1	Bacteria	*Lactobacillus plantarum*	Firmicutes	Lactobacillales	Lactobacillaceae
WP_010011356	Glp F0	Bacteria	*Lactobacillus coryniformis*	Firmicutes	Lactobacillales	Lactobacillaceae
WP_021730627	Glp F0	Bacteria	*Lactobacillus plantarum*	Firmicutes	Lactobacillales	Lactobacillaceae
WP_022834799	Glp F	Bacteria	*Salisaeta longa*	Bacteroidetes	Bacteroidetes Order II. Incertae sedis	Rhodothermaceae
WP_009538778	Glp F	Bacteria	*Caenispirillum salinarum*	Proteobacteria	Rhodospirillales	Rhodospirillaceae
WP_002983176	Glp F	Bacteria	*Chryseobacterium gleum*	Bacteroidetes	Flavobacteriales	Flavobacteriaceae
WP_007839524	Glp F	Bacteria	*Chryseobacterium* sp.	Bacteroidetes	Flavobacteriales	Flavobacteriaceae
WP_017495266	Glp F	Bacteria	*Flavobacterium* sp.	Bacteroidetes	Flavobacteriales	Flavobacteriaceae
YP_003516494	Glp F	Bacteria	*Helicobacter mustelae*	Proteobacteria	Campylobacterales	Helicobacteraceae
**Archaea**						
ENO12408	Aqp Z	Archaea	*Thermoplasmatales archaeon*	Euryarchaeota	Thermoplasmatales	
ABS54535	Aqp Z	Archaea	*Methanoregula boonei*	Euryarchaeota	Methanomicrobiales	Methanoregulaceae
CAJE01000012	Aqp Z	Archaea	*Methanomassiliicoccus luminyensis*	Euryarchaeota		
CBWS010000034	Aqp Z	Archaea	*Methanobrevibacter oralis*	Euryarchaeota	Methanomicrobiales	Methanobacteriacea
AFPU01000001/WP_007549899	Aqp Z	Archaea	*Candidatus Nitrosoarchaeum koreensis*	Thaumarchaeota	Nitrosopumilales	Nitrosopumilaceae
WP_010194415	Aqp Z	Archaea	*Candidatus Nitrosoarchaeum limnia*	Thaumarchaeota	Nitrosopumilales	Nitrosopumilaceae
WP_013481461	Aqp Z	Archaea	*Cenarchaeum symbiosum*	Thaumarchaeota	Cenarchaeales	Cenarchaeaceae
ABZ07323	Aqp Z	Archaea	*uncultured marine crenarchaeote*	Thaumarchaeota	Nitrosopumilales	
WP_020248619	Aqp Z	Archaea	*Marine Group I thaumarchaeote*	Thaumarchaeota		
YP_001581895	Aqp Z	Archaea	*Nitrosopumilus maritimus*	Thaumarchaeota	Nitrosopumilales	Nitrosopumilaceae
YP_006862679	Aqp Z	Archaea	*Candidatus Nitrososphaera gargensisA*	Thaumarchaeota	Nitrososphaerales	Nitrososphaeraceae
YP_001581823	Aqp N	Archaea	*Nitrosopumilus maritimus*	Thaumarchaeota	Nitrosopumilales	Nitrosopumilaceae
WP_016939606	Aqp N	Archaea	*Nitrosopumilus*	Thaumarchaeota	Nitrosopumilales	Nitrosopumilaceae
YP_006775241	Aqp N	Archaea	*Candidatus Nitrosopumilus*	Thaumarchaeota	Nitrosopumilales	Nitrosopumilaceae
AFPU01000001/WP_007549899	Aqp N	Archaea	*Candidatus Nitrosoarchaeum koreensis*	Thaumarchaeota	Nitrosopumilales	Nitrosopumilaceae
WP_007401177	Aqp N	Archaea	*Candidatus Nitrosoarchaeum limnia*	Thaumarchaeota	Nitrosopumilales	Nitrosopumilaceae
CDI05057	Aqp N	Archaea	*Thaumarchaeota archaeon*	Thaumarchaeota		
YP_006864154	Aqp N	Archaea	*Candidatus Nitrososphaera gargensis*	Thaumarchaeota	Nitrososphaerales	Nitrososphaeraceae
ADI23412	Aqp N	g-proteobacteria	*uncultured gamma proteobacterium*			
WP_020248263	Aqp N	Archaea	*Marine Group I thaumarchaeote*	Thaumarchaeota		
ABZ06902	Aqp N	Archaea	*uncultured marine crenarchaeote*	Thaumarchaeota	Nitrosopumilales	
YP_876286	Aqp N	Archaea	*Cenarchaeum symbiosum A*	Thaumarchaeota	Cenarchaeales	Cenarchaeaceae
AB055880	Aqp M	Archaea	*Methanothermobacter thermautotrophicus*	Euryarchaeota	Methanobacteriales	Methanobacteriacea
NP_988083	Aqp M	Archaea	*Methanococcus maripaludis*	Euryarchaeota	Methanococcales	Methanococcaceae
YP_003435795	Aqp M	Archaea	*Ferroglobus placidus*	Euryarchaeota	Archaeoglobales	Archaeoglobaceae
YP_843562	Aqp M	Archaea	*Methanosaeta thermophila*	Euryarchaeota	Methanosarcinales	Methanosaetaceae
EHQ34940	Aqp M	Archaea	*Methanoplanus limicola*	Euryarchaeota	Methanomicrobiales	Methanomicrobiacea
YP_502322	Aqp M	Archaea	*Methanospirillum hungatei*	Euryarchaeota	Methanomicrobiales	Methanospirillaceae
CP003362	Aqp M	Archaea	*Methanomethylovorans hollandica*	Euryarchaeota	Methanosarcinales	Methanosarcinaceae
WP_023844279	Aqp M	Archaea	*Methanolobus tindarius*	Euryarchaeota	Methanosarcinales	Methanosarcinaceae
YP_305556	Aqp M	Archaea	*Methanosarcina barkeri*	Euryarchaeota	Methanosarcinales	Methanosarcinaceae

YP_003424112	Aqp	M	Archaea	*Methanobrevibacter ruminantium*	Euryarchaeota	Methanobacteriales	Methanobacteriacea
BAGX02000010	Aqp	M	Archaea	*Methanobrevibacter boviskoreani*	Euryarchaeota	Methanobacteriales	Methanobacteriacea
ELZ31998	Glp	F	Archaea	*Halorubrum tebenquichense*	Euryarchaeota	Halobacteriales	Halobacteriaceae
CDK40396	Glp	F	Archaea	*Halorubrum* sp.	Euryarchaeota	Halobacteriales	Halobacteriaceae
AQZY01000011	Glp	F	Archaea	*Halomicrobium katesii*	Euryarchaeota	Halobacteriales	Halobacteriaceae
YP_003178328	Glp	F	Archaea	*Halomicrobium mukohataei*	Euryarchaeota	Halobacteriales	Halobacteriaceae
