# Supplementary material for: The Lineage-Specific Evolution of Aquaporin Gene Clusters Facilitated Tetrapod Terrestrial Adaptation
Source: PLoS One. 2014 Nov 26;9(11):e113686. doi: 10.1371/journal.pone.0113686 (PMC4245216; doi:10.1371/journal.pone.0113686)
Supplement: Table S3 — List of nuclear receptor accession numbers used in the study. (PDF) [file pone.0113686.s024.pdf]

Table S3: List of nuclear receptor accession numbers used in the study

Accession #	ortholog	Animal	Species	Rank	Order	Family
NR1B1 (RARA)						
ENSP00000377643	RAR A	Human	*Homo sapiens*	Euarchontoglires	Primates	Hominidae
XP_003786444	RAR A	Bushbaby	*Otolemur garnettii*	Euarchontoglires	Primates	Galagidae
ENSMUSP00000103097	RAR A	Mouse	*Mus musculus*	Euarchontoglires	Rodentia	Muridae
AAC23439	RAR A	Norway rat	*Rattus norvegicus*	Euarchontoglires	Rodentia	Muridae
XP_003510532	RAR A	Chinese hamster	*Cricetulus griseus*	Euarchontoglires	Rodentia	Cricetidae
XP_002719400	RAR A	Rabbit	*Oryctolagus cuniculus*	Euarchontoglires	Lagomorpha	Leporidae
XP_003131521	RAR A	Pig	*Sus scrofa*	Laurasiatheria	Suina	Suidae
ENSCAFP00000023667	RAR A	Dog	*Canis lupus familiaris*	Laurasiatheria	Carnivora	Canidae
XP_003414759	RAR A	African savanna elephant	*Loxodonta africana*	Afrotheria	Proboscidea	Elephantidae
ENSSHAP00000014651	RAR A	Tasmanian devil	*Sarcophilus harrisii*	Metatheria	Dasyuromorphia	Dasyuridae
ENSMODP00000016410	RAR A	Gray short-tailed opossum	*Monodelphis domestica*	Metatheria	Didelphimorphia	Didelphidae
CAA55134	RAR A	Chicken	*Gallus gallus*	Aves	Galliformes	Phasianidae
AGAI01014909	RAR A	Budgerigar	*Melopsittacus undulatus*	Aves	Psittaciformes	Psittacidae
AKZB01029703/AKZB01029702	RAR A	Medium ground finch	*Geospiza fortis*	Aves	Passeriformes	Fringillidae
ENSTGUP00000014154	RAR A	Zebra finch	*Taeniopygia guttata*	Aves	Passeriformes	Estrildidae
AKHW01067159	RAR A	American alligator	*Alligator mississippiensis*	Archosauria	Crocodilia	Crocodylidae
AGCU01027702/AGCU01027703	RAR A	Chinese softshell turtle	*Pelodiscus sinensis*	Sauropsida	Testudines	Trionychidae
AHGY01322541/AHGY01322542/AHGY01499434/AHGY01129006	RAR A	Western painted turtle	*Chrysemys picta bellii*	Sauropsida	Testudines	Emydidae
XP_003222505	RAR A	Green anole	*Anolis carolinensis*	Lepidosauria	Squamata	Iguanidae
AEQU010041913/AEQU011121736/AEQU010189094/ AEQU010391131/AEQU010328433/AEQU010256334	RAR A	Burmese python	*Python molurus bivittatus*	Lepidosauria	Squamata	Pythonidae
AAI69954	RAR A	African clawed frog	*Xenopus laevis*	Amphibia	Anura	Pipidae
ENSXETP00000059385	RAR A	Western clawed frog	*Xenopus (Silurana) tropicalis*	Amphibia	Anura	Pipidae
CAA78621	RAR A	Eastern newt	*Notophthalmus viridescens*	Amphibia	Caudata	Salamandridae
ENSLACP00000007011/AFYH01112411/AFYH01112418/ AFYH01112422/AFYH01112426/AFYH01112427/ AFYH01112429/AFYH01112430	Rar A	Coelacanth	*Latimeria chalumnae*	Actinistia	Coelacanthiformes	Coelacanthidae
ENSTRUP00000026288	Rar aa	Torafugu	*Takifugu rubripes*	Acanthopterygii	Tetraodontiformes	Tetraodontidae
ENSTNIP00000002381	Rar aa	Green-spotted pufferfish	*Tetraodon nigroviridis*	Acanthopterygii	Tetraodontiformes	Tetraodontidae
ACE78545	Rar aa	Spotted sand bass	*Paralabrax maculatofasciatus*	Acanthopterygii	Perciformes	Serranidae
CABK01017797	Rar aa	European seabass	*Dicentrarchus labrax*	Acanthopterygii	Perciformes	Moronidae
XP_003446494/ENSONIP00000007963	Rar aa	Nile tilapia	*Oreochromis niloticus*	Acanthopterygii	Perciformes	Cichlidae
AFNZ01044222/AFNZ01044219/AFNZ01044218	Rar aa	Burton's mouthbrooder	*Haplochromis burtoni*	Acanthopterygii	Perciformes	Cichlidae
ENSGACP00000007020	Rar aa	Three-spined stickleback	*Gasterosteus aculeatus*	Acanthopterygii	Gasterosteiformes	Gasterosteidae
ABY75226	Rar aa	False kelpfish	*Sebastiscus marmoratus*	Acanthopterygii	Scorpaeniformes	Sebastidae
CAEA01193379/CAEA01193377/CAEA01426566/ CAEA01102288/CAEA01193376/CAEA01414089/ CAEA01193375/CAEA01404776	Rar aa	Atlantic cod	*Gadus morhua*	Paracanthopterygii	Gadiformes	Gadidae
AGKD01043803/AGKD01260593/AGKD01364498	Rar aa1	Atlantic salmon	*Salmo salar*	Protacanthopterygii	Salmoniformes	Salmonidae
AGKD01013989/AGKD01260592	Rar aa2	Atlantic salmon	*Salmo salar*	Protacanthopterygii	Salmoniformes	Salmonidae
DT272322	Rar aa	Fathead minnow	*Pimephales promelas*	Ostariophysi	Cypriniformes	Cyprinidae
ABO64235	Rar aa	Rare gudgeon	*Gobiocypris rarus*	Ostariophysi	Cypriniformes	Cyprinidae
ENSDARP00000044676	Rar aa	Zebrafish	*Danio rerio*	Ostariophysi	Cypriniformes	Cyprinidae
FS997886	Rar aa	Crucian carp	*Carassius auratus langsdorfii*	Ostariophysi	Cypriniformes	Cyprinidae
FD026736	Rar aa	Channel catfish	*Ictalurus punctatus*	Ostariophysi	Siluriformes	Ictaluridae
ENSTRUP00000041964	Rar ab	Torafugu	*Takifugu rubripes*	Acanthopterygii	Tetraodontiformes	Tetraodontidae
ENSTNIP00000015825	Rar ab	Green-spotted pufferfish	*Tetraodon nigroviridis*	Acanthopterygii	Tetraodontiformes	Tetraodontidae
BAB71756	Rar ab	Japanese flounder	*Paralichthys olivaceus*	Acanthopterygii	Pleuronectiformes	Paralichthyidae
EU643830	Rar ab	Gilthead seabream	*Sparus aurata*	Acanthopterygii	Perciformes	Sparidae
ACE78544	Rar ab	Spotted sand bass	*Paralabrax maculatofasciatus*	Acanthopterygii	Perciformes	Serranidae
ABO15682	Rar ab	Striped trumpeter	*Latris lineata*	Acanthopterygii	Perciformes	Latridae
AEU04706	Rar ab	Japanese seabass	*Lateolabrax japonicus*	Acanthopterygii	Perciformes	Lateolabracidae
CBN81260	Rar ab	European seabass	*Dicentrarchus labrax*	Acanthopterygii	Perciformes	Moronidae
XP_003438797/ENSONIP00000025082/ ENSONIP00000025083	Rar ab	Nile tilapia	*Oreochromis niloticus*	Acanthopterygii	Perciformes	Cichlidae
AFNZ01022078	Rar ab	Burton's mouthbrooder	*Haplochromis burtoni*	Acanthopterygii	Perciformes	Cichlidae
ENSORLP00000005480	Rar ab	Japanese medaka	*Oryzias latipes*	Acanthopterygii	Beloniformes	Adrianichthyidae
ENSXMAP00000014668	Rar ab	Southern platyfish	*Xiphophorus maculatus*	Acanthopterygii	Cyprinodontiformes	Poeciliidae
CAEA01068110/CAEA01068116/CAEA01068118/ CAEA01054693/CAEA01469673/CAEA01068120	Rar ab	Atlantic cod	*Gadus morhua*	Paracanthopterygii	Gadiformes	Gadidae
CAI94599	Rar ab	Rainbow trout	*Oncorhynchus mykiss*	Protacanthopterygii	Salmoniformes	Salmonidae
AGKD01428981/AGKD01066346	Rar ab1	Atlantic salmon	*Salmo salar*	Protacanthopterygii	Salmoniformes	Salmonidae

AGKD01151497/AGKD01385309/AGKD01156704/ AGKD01074648	Rar ab2	Atlantic salmon	*Salmo salar*	Protacanthopterygii	Salmoniformes	Salmonidae
EG545841	Rar ab	Roach	*Rutilus rutilus*	Ostariophysi	Cypriniformes	Cyprinidae
ABY40364	Rar ab	Goldfish	*Carassius auratus*	Ostariophysi	Cypriniformes	Cyprinidae
ENSDARP00000073792	Rar ab	Zebrafish	*Danio rerio*	Ostariophysi	Cypriniformes	Cyprinidae
BAH03351	Rar a	Florida gar	*Lepisosteus platyrhincus*	Holostei	Semionotiformes	Lepisosteidae
AHAT01028997/AHAT01028996/AHAT01028995	Rar a	Spotted gar	*Lepisosteus oculatus*	Holostei	Semionotiformes	Lepisosteidae
BAH03356	Rar a	Cloudy catshark	*Scyliorhinus torazame*	Chondrichthyes	Carcharhiniformes	Scyliorhinidae
AESE010037082/AESE011569445/AESE011570210/ AESE010111519/AESE010498961/AESE010720032/ AESE010125191/AESE012510799	Rar a	Little skate	*Leucoraja erinacea*	Chondrichthyes	Rajiformes	Rajidae
BAH03354	Rar a	Plownose chimaera	*Callorhinchus callorynchus*	Chondrichthyes	Chimaeriformes	Callorhinchidae
AAVX01268707/AAVX01024001/AAVX01289872/ AAVX01524067/AAVX01131096	Rar a	Ghost shark	*Callorhinchus milii*	Chondrichthyes	Chimaeriformes	Callorhinchidae

NR1B2 (RARB)

ENSP00000332296	RAR B	Human	*Homo sapiens*	Euarchontoglires	Primates	Hominidae
ENSMUSP00000067694	RAR B	Mouse	*Mus musculus*	Euarchontoglires	Rodentia	Muridae
XP_001059523	RAR B	Norway rat	*Rattus norvegicus*	Euarchontoglires	Rodentia	Muridae
AAL02361	RAR B	Golden hamster	*Mesocricetus auratus*	Euarchontoglires	Rodentia	Cricetidae
ENSCPOP00000013894	RAR B	Domestic guinea pig	*Cavia porcellus*	Euarchontoglires	Rodentia	Caviidae
ENSCAFP00000008570	RAR B	Dog	*Canis lupus familiaris*	Laurasiatheria	Carnivora	Canidae
ENSECAP00000014556	RAR B	Horse	*Equus caballus*	Laurasiatheria	Perissodactyla	Equidae
ENSLAFP00000013272	RAR B	African savanna elephant	*Loxodonta africana*	Afrotheria	Proboscidea	Elephantidae
ENSSHAP00000011399	RAR B	Tasmanian devil	*Sarcophilus harrisii*	Metatheria	Dasyuromorphia	Dasyuridae
ENSMODP00000018662	RAR B	Gray short-tailed opossum	*Monodelphis domestica*	Metatheria	Didelphimorphia	Didelphidae
ENSOANP00000019364	RAR B	Platypus	*Ornithorhynchus anatinus*	Prototheria	Monotremata	Ornithorhynchida
CAA42077	RAR B	Chicken	*Gallus gallus*	Aves	Galliformes	Phasianidae
XP_003207239	RAR B	Turkey	*Meleagris gallopavo*	Aves	Galliformes	Phasianidae
AAD23397	RAR B	Common quail	*Coturnix coturnix*	Aves	Galliformes	Phasianidae
ENSAPLP00000006083	RAR B	Mallard	*Anas platyrhynchos*	Aves	Anseriformes	Anatidae
AGAI01066075/AGAI01066076	RAR B	Budgerigar	*Melopsittacus undulatus*	Aves	Psittaciformes	Psittacidae
AKZB01009618/AKZB01009619	RAR B	Medium ground finch	*Geospiza fortis*	Aves	Passeriformes	Fringillidae
ENSTGUP00000003501	RAR B	Zebra finch	*Taeniopygia guttata*	Aves	Passeriformes	Estrildidae
AKHW01034722/AKHW01034721/AKHW01034720/ AKHW01034718	RAR B	American alligator	*Alligator mississippiensis*	Archosauria	Crocodilia	Crocodylidae
BAE78778/ENSPSIP00000008839	RAR B	Chinese softshell turtle	*Pelodiscus sinensis*	Sauropsida	Testudines	Trionychidae
AHGY01380674/AHGY01380678/AHGY01380680/ AHGY01380683	RAR B	Western painted turtle	*Chrysemys picta bellii*	Sauropsida	Testudines	Emydidae
ENSACAP00000009133	RAR B	Green anole	*Anolis carolinensis*	Lepidosauria	Squamata	Iguanidae
AEQU011150482/AEQU010321079/AEQU011025087/ AEQU010174987/AEQU010303692/AEQU011137768/ AEQU010350387/AEQU010065410	RAR B	Burmese python	*Python molurus bivittatus*	Lepidosauria	Squamata	Pythonidae
ABE96637	RAR B	African clawed frog	*Xenopus laevis*	Amphibia	Anura	Pipidae
ENSXETP00000015821	RAR B	Western clawed frog	*Xenopus (Silurana) tropicalis*	Amphibia	Anura	Pipidae
AAW34271	RAR B	Eastern newt	*Notophthalmus viridescens*	Amphibia	Caudata	Salamandridae
ENSLACP00000007216/AFYH01175751/AFYH01175750/ AFYH01175748/AFYH01175744/AFYH01175743/ AFYH01175742	Rar b	Coelacanth	*Latimeria chalumnae*	Actinistia	Coelacanthiformes	Coelacanthidae
ENSTRUP00000018286	Rar ba	Torafugu	*Takifugu rubripes*	Acanthopterygii	Tetraodontiformes	Tetraodontidae
ENSTNIP00000021339	Rar ba	Green-spotted pufferfish	*Tetraodon nigroviridis*	Acanthopterygii	Tetraodontiformes	Tetraodontidae
BAB71757	Rar ba	Japanese flounder	*Paralichthys olivaceus*	Acanthopterygii	Pleuronectiformes	Paralichthyidae
ENSONIP00000012973	Rar ba	Nile tilapia	*Oreochromis niloticus*	Acanthopterygii	Perciformes	Cichlidae
AFNZ01028543/AFNZ01028548/AFNZ01028551/ AFNZ01028552	Rar ba	Burton's mouthbrooder	*Haplochromis burtoni*	Acanthopterygii	Perciformes	Cichlidae
ENSGACG00000007999	Rar ba	Three-spined stickleback	*Gasterosteus aculeatus*	Acanthopterygii	Gasterosteiformes	Gasterosteidae
ENSORLP00000010679	Rar ba	Japanese medaka	*Oryzias latipes*	Acanthopterygii	Beloniformes	Adrianichthyidae
ENSXMAP00000017097	Rar ba	Southern platyfish	*Xiphophorus maculatus*	Acanthopterygii	Cyprinodontiformes	Poeciliidae
CAEA01455142/CAEA01455139/CAEA01265622/ CAEA01455134/CAEA01109765/CAEA01455130/ CAEA01424100	Rar ba	Atlantic cod	*Gadus morhua*	Paracanthopterygii	Gadiformes	Gadidae
AGKD01290700/AGKD01183771/AGKD01054082/ AGKD01251166	Rar ba1	Atlantic salmon	*Salmo salar*	Protacanthopterygii	Salmoniformes	Salmonidae
AGKD01105144/AGKD01045564	Rar ba2	Atlantic salmon	*Salmo salar*	Protacanthopterygii	Salmoniformes	Salmonidae
BAB71755	Rar bb	Japanese flounder	*Paralichthys olivaceus*	Acanthopterygii	Pleuronectiformes	Paralichthyidae
CABK01018381/CABK01003564/CABK01004346	Rar bb	European seabass	*Dicentrarchus labrax*	Acanthopterygii	Perciformes	Moronidae
ENSONIP00000008188	Rar bb	Nile tilapia	*Oreochromis niloticus*	Acanthopterygii	Perciformes	Cichlidae
AFNZ01035702	Rar bb	Burton's mouthbrooder	*Haplochromis burtoni*	Acanthopterygii	Perciformes	Cichlidae
AANH01001239	Rar bb	Three-spined stickleback	*Gasterosteus aculeatus*	Acanthopterygii	Gasterosteiformes	Gasterosteidae
ENSORLP00000020526	Rar bb	Japanese medaka	*Oryzias latipes*	Acanthopterygii	Beloniformes	Adrianichthyidae
ENSXMAP00000018768	Rar bb	Southern platyfish	*Xiphophorus maculatus*	Acanthopterygii	Cyprinodontiformes	Poeciliidae
CAEA01544913/CAEA01168329/CAEA01058377/	Rar bb	Atlantic cod	*Gadus morhua*	Paracanthopterygii	Gadiformes	Gadidae

CAEA01438833							
AGKD01020798/AGKD01173114/AGKD01077839	Rar bb1	Atlantic salmon	*Salmo salar*	Protacanthopterygii	Salmoniformes	Salmonidae
AGKD01075519/AGKD01144317/AGKD01110751	Rar bb2	Atlantic salmon	*Salmo salar*	Protacanthopterygii	Salmoniformes	Salmonidae
BAH03352	Rar b	Florida gar	*Lepisosteus platyrhincus*	Holostei	Semionotiformes	Lepisosteidae
AHAT01018100	Rar b	Spotted gar	*Lepisosteus oculatus*	Holostei	Semionotiformes	Lepisosteidae
BAH03357	Rar b	Cloudy catshark	*Scyliorhinus torazame*	Chondrichthyes	Carcharhiniformes	Scyliorhinidae
AESE010036583/AESE011019117/AESE011675970/ AESE010035522/AESE012566423/AESE010143422/ AESE010649221/AESE012566423	Rar b	Little skate	*Leucoraja erinacea*	Chondrichthyes	Rajiformes	Rajidae
BAH03355	Rar b	Plownose chimaera	*Callorhinchus callorynchus*	Chondrichthyes	Chimaeriformes	Callorhinchidae
AAVX01614611/AAVX01058896/AAVX01034761/ AAVX01145199/AAVX01004709	Rar b	Ghost shark	*Callorhinchus milii*	Chondrichthyes	Chimaeriformes	Callorhinchidae
**NR1B3 (RARG)**						
ENSP00000343698	RARG	Human	*Homo sapiens*	Euarchontoglires	Primates	Hominidae
XP_003790471	RARG	Small-eared galago/Bushbaby	*Otolemur garnettii*	Euarchontoglires	Primates	Galagidae
ENSMUSP00000067266	RARG	Mouse	*Mus musculus*	Euarchontoglires	Rodentia	Muridae
EHB09080	RARG	Norway rat	*Rattus norvegicus*	Euarchontoglires	Rodentia	Muridae
AAL02362	RARG	Golden hamster	*Mesocricetus auratus*	Euarchontoglires	Rodentia	Cricetidae
XP_858281	RARG	Dog	*Canis lupus familiaris*	Laurasiatheria	Carnivora	Canidae
GENSCAN00000020267	RARG	Cape rock hyrax	*Procavia capensis*	Afrotheria	Hyracoidea	Procaviidae
ENSSHAP00000002688	RARG	Tasmanian devil	*Sarcophilus harrisii*	Metatheria	Dasyuromorphia	Dasyuridae
ENSOANP00000009414	RARG	Platypus	*Ornithorhynchus anatinus*	Prototheria	Monotremata	Ornithorhynchida
AGAI01037202	RARG	Budgerigar	*Melopsittacus undulatus*	Aves	Psittaciformes	Psittacidae
AKZB01008787	RARG	Medium ground finch	*Geospiza fortis*	Aves	Passeriformes	Fringillidae
ENSTGUP00000003933	RARG	Zebra finch	*Taeniopygia guttata*	Aves	Passeriformes	Estrildidae
AKHW01112913	RARG	American alligator	*Alligator mississippiensis*	Archosauria	Crocodilia	Crocodylidae
BAE78779/ENSPSIP00000003163	RARG	Chinese softshell turtle	*Pelodiscus sinensis*	Sauropsida	Testudines	Trionychidae
AHGY01101115/AHGY01101108/AHGY01101107/ AHGY01101106/AHGY01101105	RARG	Western painted turtle	*Chrysemys picta bellii*	Sauropsida	Testudines	Emydidae
XP_003216711	RARG	Green anole	*Anolis carolinensis*	Lepidosauria	Squamata	Iguanidae
AEQU010172570/AEQU011115330/AEQU010392377/ AEQU010164972/AEQU010196582/AEQU010192304/ AEQU011132843/AEQU011251275	RARG	Burmese python	*Python molurus bivittatus*	Lepidosauria	Squamata	Pythonidae
CAA42039	RARG	African clawed frog	*Xenopus laevis*	Amphibia	Anura	Pipidae
ENSXETP00000027722	RARG	Western clawed frog	*Xenopus (Silurana) tropicalis*	Amphibia	Anura	Pipidae
AAF80975	RARG	Axolotl	*Ambystoma mexicanum*	Amphibia	Caudata	Ambystomatidae
ENSLACP00000018412/AFYH01001224/AFYH01001236/ AFYH01001239/AFYH01001240/AFYH01001241/ AFYH01001242	Rar g	Coelacanth	*Latimeria chalumnae*	Actinistia	Coelacanthiformes	Coelacanthidae
ENSTRUP00000012109	Rar ga	Torafugu	*Takifugu rubripes*	Acanthopterygii	Tetraodontiformes	Tetraodontidae
ENSTNIP00000018102	Rar ga	Green-spotted pufferfish	*Tetraodon nigroviridis*	Acanthopterygii	Tetraodontiformes	Tetraodontidae
BAB71754	Rar ga	Japanese flounder	*Paralichthys olivaceus*	Acanthopterygii	Pleuronectiformes	Paralichthyidae
EU643831	Rar ga	Gilthead seabream	*Sparus aurata*	Acanthopterygii	Perciformes	Sparidae
ABO15683	Rar ga	Striped trumpeter	*Latris lineata*	Acanthopterygii	Perciformes	Latridae
AEU04705	Rar ga	Japanese seabass	*Lateolabrax japonicus*	Acanthopterygii	Perciformes	Lateolabracidae
CAD42660	Rar ga	European seabass	*Dicentrarchus labrax*	Acanthopterygii	Perciformes	Moronidae
ENSONIP00000015397	Rar ga	Nile tilapia	*Oreochromis niloticus*	Acanthopterygii	Perciformes	Cichlidae
AFNZ01002268	Rar ga	Burton's mouthbrooder	*Haplochromis burtoni*	Acanthopterygii	Perciformes	Cichlidae
ENSGACP00000012356	Rar ga	Three-spined stickleback	*Gasterosteus aculeatus*	Acanthopterygii	Gasterosteiformes	Gasterosteidae
ENSORLP00000009848	Rar ga	Japanese medaka	*Oryzias latipes*	Acanthopterygii	Beloniformes	Adrianichthyidae
ENSXMAP00000001617	Rar ga	Southern platyfish	*Xiphophorus maculatus*	Acanthopterygii	Cyprinodontiformes	Poeciliidae
CAEA01182950/CAEA01191319/CAEA01008904/ CAEA01182958/CAEA01182959/CAEA01308566	Rar ga	Atlantic cod	*Gadus morhua*	Paracanthopterygii	Gadiformes	Gadidae
EL545418	Rar ga	Rainbow smelt	*Osmerus mordax*	Protacanthopterygii	Osmeriformes	Osmeridae
CBY83981	Rar ga	Rainbow trout	*Oncorhynchus mykiss*	Protacanthopterygii	Salmoniformes	Salmonidae
AGKD01049338	Rar ga1	Atlantic salmon	*Salmo salar*	Protacanthopterygii	Salmoniformes	Salmonidae
AGKD01379461/AGKD01015805	Rar ga2	Atlantic salmon	*Salmo salar*	Protacanthopterygii	Salmoniformes	Salmonidae
ABO64236	Rar ga	Rare gudgeon	*Gobiocypris rarus*	Ostariophysi	Cypriniformes	Cyprinidae
ENSDARP00000049550	Rar ga	Zebrafish	*Danio rerio*	Ostariophysi	Cypriniformes	Cyprinidae
ABY40366	Rar ga	Goldfish	*Carassius auratus*	Ostariophysi	Cypriniformes	Cyprinidae
FD166645	Rar ga	Blue catfish	*Ictalurus furcatus*	Ostariophysi	Siluriformes	Ictaluridae
FD027780	Rar ga	Channel catfish	*Ictalurus punctatus*	Ostariophysi	Siluriformes	Ictaluridae
ENSTRUP00000047490	Rar gb	Torafugu	*Takifugu rubripes*	Acanthopterygii	Tetraodontiformes	Tetraodontidae
ENSTNIP00000004400	Rar gb	Green-spotted pufferfish	*Tetraodon nigroviridis*	Acanthopterygii	Tetraodontiformes	Tetraodontidae
CABK01008325	Rar gb	European seabass	*Dicentrarchus labrax*	Acanthopterygii	Perciformes	Moronidae
ENSONIP00000024125/ENSONIP00000024126	Rar gb	Nile tilapia	*Oreochromis niloticus*	Acanthopterygii	Perciformes	Cichlidae
AFNZ01012778	Rar gb	Burton's mouthbrooder	*Haplochromis burtoni*	Acanthopterygii	Perciformes	Cichlidae
ENSGACP00000000789	Rar gb	Three-spined stickleback	*Gasterosteus aculeatus*	Acanthopterygii	Gasterosteiformes	Gasterosteidae

ENSORLP00000019255	Rar gb	Japanese medaka	*Oryzias latipes*	Acanthopterygii	Beloniformes	Adrianichthyidae
ACS50391	Rar gb	Green swordtail	*Xiphophorus hellerii*	Acanthopterygii	Cyprinodontiformes	Poeciliidae
FK034608	Rar gb	Platyfish hybrid	*X. maculatusx X. helleri*	Acanthopterygii	Cyprinodontiformes	Poeciliidae
ENSXMAP00000002373	Rar gb	Southern platyfish	*Xiphophorus maculatus*	Acanthopterygii	Cyprinodontiformes	Poeciliidae
CAEA01223140/CAEA01270307/CAEA01270301/ CAEA01222100/CAEA01222101/CAEA01238594/ CAEA01238597	Rar gb	Atlantic cod	*Gadus morhua*	Paracanthopterygii	Gadiformes	Gadidae
ABW77530	Rar gb1	Atlantic salmon	*Salmo salar*	Protacanthopterygii	Salmoniformes	Salmonidae
AGKD01061507/AGKD01008455	Rar gb2	Atlantic salmon	*Salmo salar*	Protacanthopterygii	Salmoniformes	Salmonidae
ENSDARP00000052545	Rar gb	Zebrafish	*Danio rerio*	Ostariophysi	Cypriniformes	Cyprinidae
BAH03353	Rar g	Florida gar	*Lepisosteus platyrhincus*	Holostei	Semionotiformes	Lepisosteidae
AHAT01018847/AHAT01018848	Rar g	Spotted gar	*Lepisosteus oculatus*	Holostei	Semionotiformes	Lepisosteidae
DV498590	Rar g	Spiny dogfish	*Squalus acanthias*	Chondrichthyes	Squaliformes	Squalidae
AAVX01522265/AAVX01255602	Rar g	Ghost shark	*Callorhinchus milii*	Chondrichthyes	Chimaeriformes	Callorhinchidae
AAX54531/ENSPMAP00000000100	Rar 1	Sea lamprey	*Petromyzon marinus*	Hyperoartia	Petromyzontiformes	Petromyzontidae
BAH03333/APJL01000416/APJL01000417/ APJL01000418	Rar 1	Arctic lamprey	*Lethenteron camtschaticum*	Hyperoartia	Petromyzontiformes	Petromyzontidae
BAH03348	Rar 1	Southern lamprey	*Mordacia mordax*	Hyperoartia	Petromyzontiformes	Petromyzontidae
BAH03345	Rar 1	Inshore hagfish	*Eptatretus burgeri*	Hyperotreti	Myxiniformes	Myxinidae
ENSPMAP00000007854	Rar 2	Sea lamprey	*Petromyzon marinus*	Hyperoartia	Petromyzontiformes	Petromyzontidae
BAH03334/APJL01026247	Rar 2	Arctic lamprey	*Lethenteron camtschaticum*	Hyperoartia	Petromyzontiformes	Petromyzontidae
BAH03349	Rar 2	Southern lamprey	*Mordacia mordax*	Hyperoartia	Petromyzontiformes	Petromyzontidae
BAH03346	Rar 2	Inshore hagfish	*Eptatretus burgeri*	Hyperotreti	Myxiniformes	Myxinidae
ENSPMAP00000005382	Rar 3	Sea lamprey	*Petromyzon marinus*	Hyperoartia	Petromyzontiformes	Petromyzontidae
BAH03335/APJL01028812/APJL01028814/ APJL01028817/APJL01028819	Rar 3	Arctic lamprey	*Lethenteron camtschaticum*	Hyperoartia	Petromyzontiformes	Petromyzontidae
BAH03350	Rar 3	Southern lamprey	*Mordacia mordax*	Hyperoartia	Petromyzontiformes	Petromyzontidae
BAH03347	Rar 3	Inshore hagfish	*Eptatretus burgeri*	Hyperotreti	Myxiniformes	Myxinidae
ENSCINP00000024068	Rar	Vase tunicate	*Ciona intestinalis*	Tunicata	Enterogona	Cionidae
ENSCSAVP00000011583	Rar	Pacific transparent sea squirt	*Ciona savignyi*	Tunicata	Enterogona	Cionidae
D86615/BAA25569	Rar	Compound ascidian	*Polyandrocarpa misakiensis*	Tunicata	Stolidobranchia	Styelidae
AAM46149	Rar	Florida lancelet	*Branchiostoma floridae*	Cephalochordata	Amphioxiformes	Branchiostomidae
XP_002742241	Rar	Acorn worm	*Saccoglossus kowalevskii*	Hemichordata		Harrimaniidae
XP_001196076	Rar	Purple sea urchin	*Strongylocentrotus purpuratus*	Echinodermata	Echinozoa	Strongylocentroti ae
AGCV01396922/AGCV01396921	Rar	Green sea urchin	*Lytechinus variegatus*	Echinodermata	Echinozoa	Toxopneustidae
AKZP01136683/AKZP01023211/AKZP01023213/ AKZP01023214	Rar	Bat star	*Patiria miniata*	Echinodermata	Asterozoa	Asterinidae

NR1D1

ENSP00000246672	NR1D1	Human	*Homo sapiens*	Euarchontoglires	Primates	Hominidae
ENSMUSP00000069505	NR1D1	Mouse	*Mus musculus*	Euarchontoglires	Rodentia	Muridae
ENSOCUP00000008383	NR1D1	Rabbit	*Oryctolagus cuniculus*	Euarchontoglires	Lagomorpha	Leporidae
ENSECAP00000004793	NR1D1	Horse	*Equus caballus*	Laurasiatheria	Perissodactyla	Equidae
ENSCAFP00000023799	NR1D1	Dog	*Canis lupus familiaris*	Laurasiatheria	Carnivora	Canidae
ENSLAFP00000012586	NR1D1	African savanna elephant	*Loxodonta africana*	Afrotheria	Proboscidea	Elephantidae
ENSSHAP00000015917	NR1D1	Tasmanian devil	*Sarcophilus harrisii*	Metatheria	Dasyuromorphia	Dasyuridae
ADG08189	NR1D1	Long-nosed potoroo	*Potorous tridactylus*	Metatheria	Diprotodontia	Potoroidae
ENSMODP00000016298	NR1D1	Gray short-tailed opossum	*Monodelphis domestica*	Metatheria	Didelphimorphia	Didelphidae
AAPN01100082	NR1D1	Platypus	*Ornithorhynchus anatinus*	Prototheria	Monotremata	Ornithorhynchidae
GENSCAN00000041867	NR1D1	Mallard	*Anas platyrhynchos*	Aves	Anseriformes	Anatidae
AKZB01017790/AKZB01017789	NR1D1	Medium ground finch	*Geospiza fortis*	Aves	Passeriformes	Fringillidae
AKHW01067166	NR1D1	American alligator	*Alligator mississippiensis*	Archosauria	Crocodilia	Crocodylidae
AGCU01027641/AGCU01027640	NR1D1	Chinese softshell turtle	*Pelodiscus sinensis*	Sauropsida	Testudines	Trionychidae
AHGY01129020	NR1D1	Western painted turtle	*Chrysemys picta bellii*	Sauropsida	Testudines	Emydidae
ENSACAP00000002780	NR1D1	Green anole	*Anolis carolinensis*	Lepidosauria	Squamata	Iguanidae
AEQU010097485/AEQU011155290/AEQU011219681/ AEQU010343226/AEQU010283292	NR1D1	Burmese python	*Python molurus bivittatus*	Lepidosauria	Squamata	Pythonidae
NP_001085940	NR1D1	African clawed frog	*Xenopus laevis*	Amphibia	Anura	Pipidae
ENSXETP00000052764	NR1D1	Western clawed frog	*Xenopus (Silurana) tropicalis*	Amphibia	Anura	Pipidae
GAQK01020025	NR1D1	Chinese salamander	*Hynobius chinensis*	Amphibia	Caudata	Hynobiidae
FL668910	Nr1d1	Marbled lungfish	*Protopterus aethiopicus*	Dipnoi	Lepidosireniformes	Protopteridae
ENSLACP00000011083	Nr1d1	Coelacanth	*Latimeria chalumnae*	Actinistia	Coelacanthiformes	Coelacanthidae
ENSONIP00000011673	Nr1d1	Nile tilapia	*Oreochromis niloticus*	Acanthopterygii	Perciformes	Cichlidae
AFNZ01045817	Nr1d1	Burton's mouthbrooder	*Haplochromis burtoni*	Acanthopterygii	Perciformes	Cichlidae
ENSXMAP00000018990	Nr1d1	Southern platyfish	*Xiphophorus maculatus*	Acanthopterygii	Cyprinodontiformes	Poeciliidae
CAEA01004309/CAEA01024743/ENSGMOP00000012307	Nr1d1	Atlantic cod	*Gadus morhua*	Paracanthopterygii	Gadiformes	Gadidae
CA361593	Nr1d1	Rainbow trout	*Oncorhynchus mykiss*	Protacanthopterygii	Salmoniformes	Salmonidae
AGKD01078589/AGKD01001588	Nr1d1-1	Atlantic salmon	*Salmo salar*	Protacanthopterygii	Salmoniformes	Salmonidae

AGKD01191165	Nr1d1-2	Atlantic salmon	*Salmo salar*	Protacanthopterygii	Salmoniformes	Salmonidae
ENSDARP00000040467	Nr1d1	Zebrafish	*Danio rerio*	Ostariophysi	Cypriniformes	Cyprinidae
AHAT01030996	Nr1d1	Spotted gar	*Lepisosteus oculatus*	Holostei	Semionotiformes	Lepisosteidae
AESE012038357/AESE010971583/AESE011557077/AESE010712799/AESE010707536	Nr1d1	Little skate	*Leucoraja erinacea*	Chondrichthyes	Rajiformes	Rajidae
AAVX01075989/AAVX01260154/AAVX01621180/AAVX01450687/AAVX01626810	Nr1d1	Ghost shark	*Callorhinchus milii*	Chondrichthyes	Chimaeriformes	Callorhinchidae

NR1D2

ENSP00000310006	NR1D2	Human	*Homo sapiens*	Euarchontoglires	Primates	Hominidae
ENSMUSP00000088031	NR1D2	Mouse	*Mus musculus*	Euarchontoglires	Rodentia	Muridae
ENSOCUP00000017956	NR1D2	Rabbit	*Oryctolagus cuniculus*	Euarchontoglires	Lagomorpha	Leporidae
ENSCAFP00000008613	NR1D2	Dog	*Canis lupus familiaris*	Laurasiatheria	Carnivora	Canidae
ENSECAP00000005911	NR1D2	Horse	*Equus caballus*	Laurasiatheria	Perissodactyla	Equidae
ENSLAFP00000009901	NR1D2	African savanna elephant	*Loxodonta africana*	Afrotheria	Proboscidea	Elephantidae
AFEY01455850/ENSSHAP00000001535/ENSSHAP00000001536	NR1D2	Tasmanian devil	*Sarcophilus harrisii*	Metatheria	Dasyuromorphia	Dasyuridae
ENSMODP00000018624	NR1D2	Gray short-tailed opossum	*Monodelphis domestica*	Metatheria	Didelphimorphia	Didelphidae
ENSOANP00000005607	NR1D2	Platypus	*Ornithorhynchus anatinus*	Prototheria	Monotremata	Ornithorhynchida
ENSGALP00000018398	NR1D2	Chicken	*Gallus gallus*	Aves	Galliformes	Phasianidae
ENSMGAP00000011504	NR1D2	Turkey	*Meleagris gallopavo*	Aves	Galliformes	Phasianidae
ENSAPLP00000005377	NR1D2	Mallard	*Anas platyrhynchos*	Aves	Anseriformes	Anatidae
AGAI01066064	NR1D2	Budgerigar	*Melopsittacus undulatus*	Aves	Psittaciformes	Psittacidae
AKZB01009592	NR1D2	Medium ground finch	*Geospiza fortis*	Aves	Passeriformes	Fringillidae
ENSTGUP00000003445	NR1D2	Zebra finch	*Taeniopygia guttata*	Aves	Passeriformes	Estrildidae
AKHW01034761	NR1D2	American alligator	*Alligator mississippiensis*	Archosauria	Crocodilia	Crocodylidae
AGCU01160982	NR1D2	Chinese softshell turtle	*Pelodiscus sinensis*	Sauropsida	Testudines	Trionychidae
AHGY01380618	NR1D2	Western painted turtle	*Chrysemys picta bellii*	Sauropsida	Testudines	Emydidae
ENSACAP00000011193	NR1D2	Green anole	*Anolis carolinensis*	Lepidosauria	Squamata	Iguanidae
AEQU010169819/AEQU010367823/AEQU010100244/AEQU011100668/AEQU010242342	NR1D2	Burmese python	*Python molurus bivittatus*	Lepidosauria	Squamata	Pythonidae
ENSXETP00000008395	NR1D2	Western clawed frog	*Xenopus (Silurana) tropicalis*	Amphibia	Anura	Pipidae
GAQK01061527	NR1D2	Chinese salamander	*Hynobius chinensis*	Amphibia	Caudata	Hynobiidae
ENSLACP00000003604	Nr1d2	Coelacanth	*Latimeria chalumnae*	Actinistia	Coelacanthiformes	Coelacanthidae
ENSTRUP00000021186	Nr1d2a	Torafugu	*Takifugu rubripes*	Acanthopterygii	Tetraodontiformes	Tetraodontidae
ENSTNIP00000012684	Nr1d2a	Green-spotted pufferfish	*Tetraodon nigroviridis*	Acanthopterygii	Tetraodontiformes	Tetraodontidae
ENSONIP00000012960	Nr1d2a	Nile tilapia	*Oreochromis niloticus*	Acanthopterygii	Perciformes	Cichlidae
AFNZ01028508	Nr1d2a	Burton's mouthbrooder	*Haplochromis burtoni*	Acanthopterygii	Perciformes	Cichlidae
ENSGACP00000010577	Nr1d2a	Three-spined stickleback	*Gasterosteus aculeatus*	Acanthopterygii	Gasterosteiformes	Gasterosteidae
ENSXMAP00000017164	Nr1d2a	Southern platyfish	*Xiphophorus maculatus*	Acanthopterygii	Cyprinodontiformes	Poeciliidae
ENSGMOP00000008612	Nr1d2a	Atlantic cod	*Gadus morhua*	Paracanthopterygii	Gadiformes	Gadidae
AGKD01038291	Nr1d2a	Atlantic salmon	*Salmo salar*	Protacanthopterygii	Salmoniformes	Salmonidae
AGKD01009713	Nr1d2a	Atlantic salmon	*Salmo salar*	Protacanthopterygii	Salmoniformes	Salmonidae
ENSDARP00000095239	Nr1d2a	Zebrafish	*Danio rerio*	Ostariophysi	Cypriniformes	Cyprinidae
ENSTRUP00000023087	Nr1d2b	Torafugu	*Takifugu rubripes*	Acanthopterygii	Tetraodontiformes	Tetraodontidae
ENSTNIP00000014607	Nr1d2b	Green-spotted pufferfish	*Tetraodon nigroviridis*	Acanthopterygii	Tetraodontiformes	Tetraodontidae
FM149274	Nr1d2b	Gilthead seabream	*Sparus aurata*	Acanthopterygii	Perciformes	Sparidae
ENSONIP00000010933	Nr1d2b	Nile tilapia	*Oreochromis niloticus*	Acanthopterygii	Perciformes	Cichlidae
AFNZ01011498	Nr1d2b	Burton's mouthbrooder	*Haplochromis burtoni*	Acanthopterygii	Perciformes	Cichlidae
ENSGACP00000017128	Nr1d2b	Three-spined stickleback	*Gasterosteus aculeatus*	Acanthopterygii	Gasterosteiformes	Gasterosteidae
ENSORLP00000020574	Nr1d2b	Japanese medaka	*Oryzias latipes*	Acanthopterygii	Beloniformes	Adrianichthyidae
ENSXMAP00000018756	Nr1d2b	Southern platyfish	*Xiphophorus maculatus*	Acanthopterygii	Cyprinodontiformes	Poeciliidae
ENSGMOP00000020628	Nr1d2b	Atlantic cod	*Gadus morhua*	Paracanthopterygii	Gadiformes	Gadidae
AGKD01030814	Nr1d2b1	Atlantic salmon	*Salmo salar*	Protacanthopterygii	Salmoniformes	Salmonidae
AGKD01021247	Nr1d2b2	Atlantic salmon	*Salmo salar*	Protacanthopterygii	Salmoniformes	Salmonidae
ENSDARP00000089813	Nr1d2b	Zebrafish	*Danio rerio*	Ostariophysi	Cypriniformes	Cyprinidae
AHAT01018098	Nr1d2	Spotted gar	*Lepisosteus oculatus*	Holostei	Semionotiformes	Lepisosteidae
DV497271	Nr1d2	Spiny dogfish	*Squalus acanthias*	Chondrichthyes	Squaliformes	Squalidae
AESE010109085/AESE011522446/AESE012532739	Nr1d2	Little skate	*Leucoraja erinacea*	Chondrichthyes	Rajiformes	Rajidae
AAVX01171812/AAVX01264188/AAVX01405277	Nr1d2	Ghost shark	*Callorhinchus milii*	Chondrichthyes	Chimaeriformes	Callorhinchidae

NR1D4

AKHW01112917	NR1D4	American alligator	*Alligator mississippiensis*	Archosauria	Crocodilia	Crocodylidae
AGCU01201650/AGCU01201651/AGCU01201655	NR1D4	Chinese softshell turtle	*Pelodiscus sinensis*	Sauropsida	Testudines	Trionychidae
AHGY01101170/AHGY01101171	NR1D4	Western painted turtle	*Chrysemys picta bellii*	Sauropsida	Testudines	Emydidae
ENSACAP00000006630	NR1D4	Green anole	*Anolis carolinensis*	Lepidosauria	Squamata	Iguanidae
AEQU010262993/AEQU010287308/AEQU010178524/AEQU011174561/AEQU010340121	NR1D4	Burmese python	*Python molurus bivittatus*	Lepidosauria	Squamata	Pythonidae
GAQK01119034/GAQK01117772	NR1D4	Chinese salamander	*Hynobius chinensis*	Amphibia	Caudata	Hynobiidae

ENSLACP00000016868	Nr1d4	Coelacanth	*Latimeria chalumnae*	Actinistia	Coelacanthiformes	Coelacanthidae
ENSTRUP00000014050	Nr1d4a	Torafugu	*Takifugu rubripes*	Acanthopterygii	Tetraodontiformes	Tetraodontidae
ENSTNIP00000018103	Nr1d4a	Green-spotted pufferfish	*Tetraodon nigroviridis*	Acanthopterygii	Tetraodontiformes	Tetraodontidae
EY456504	Nr1d4a	Turbot	*Scophthalmus maximus*	Acanthopterygii	Pleuronectiformes	Scophthalmidae
ENSONIP00000015382/ENSONIP00000015383	Nr1d4a	Nile tilapia	*Oreochromis niloticus*	Acanthopterygii	Perciformes	Cichlidae
AFNZ01002269	Nr1d4a	Burton's mouthbrooder	*Haplochromis burtoni*	Acanthopterygii	Perciformes	Cichlidae
ENSGACP00000012345	Nr1d4a	Three-spined stickleback	*Gasterosteus aculeatus*	Acanthopterygii	Gasterosteiformes	Gasterosteidae
ENSORLP00000009821	Nr1d4a	Japanese medaka	*Oryzias latipes*	Acanthopterygii	Beloniformes	Adrianichthyidae
ENSXMAP00000011486	Nr1d4a	Southern platyfish	*Xiphophorus maculatus*	Acanthopterygii	Cyprinodontiformes	Poeciliidae
ENSGMOP00000005632	Nr1d4a	Atlantic cod	*Gadus morhua*	Paracanthopterygii	Gadiformes	Gadidae
AGKD01011416	Nr1d4a1	Atlantic salmon	*Salmo salar*	Protacanthopterygii	Salmoniformes	Salmonidae
EG536356	Nr1d4a	Roach	*Rutilus rutilus*	Ostariophysi	Cypriniformes	Cyprinidae
ENSDARP00000107788	Nr1d4a	Zebrafish	*Danio rerio*	Ostariophysi	Cypriniformes	Cyprinidae
ENSTRUP00000020506	Nr1d4b	Torafugu	*Takifugu rubripes*	Acanthopterygii	Tetraodontiformes	Tetraodontidae
ENSTNIP00000017591	Nr1d4b	Green-spotted pufferfish	*Tetraodon nigroviridis*	Acanthopterygii	Tetraodontiformes	Tetraodontidae
FM157437	Nr1d4b	Gilthead seabream	*Sparus aurata*	Acanthopterygii	Perciformes	Sparidae
ENSONIP00000024124	Nr1d4b	Nile tilapia	*Oreochromis niloticus*	Acanthopterygii	Perciformes	Cichlidae
AFNZ01012785	Nr1d4b	Burton's mouthbrooder	*Haplochromis burtoni*	Acanthopterygii	Perciformes	Cichlidae
GO627620	Nr1d4b	Sablefish	*Anoplopoma fimbria*	Acanthopterygii	Scorpaeniformes	Anoplopomatidae
ENSGACP00000000791	Nr1d4b	Three-spined stickleback	*Gasterosteus aculeatus*	Acanthopterygii	Gasterosteiformes	Gasterosteidae
ENSORLP00000019274	Nr1d4b	Japanese medaka	*Oryzias latipes*	Acanthopterygii	Beloniformes	Adrianichthyidae
ENSXMAP00000002354	Nr1d4b	Southern platyfish	*Xiphophorus maculatus*	Acanthopterygii	Cyprinodontiformes	Poeciliidae
ENSGMOP00000019693	Nr1d4b	Atlantic cod	*Gadus morhua*	Paracanthopterygii	Gadiformes	Gadidae
CA382526	Nr1d4b1	Rainbow trout	*Oncorhynchus mykiss*	Protacanthopterygii	Salmoniformes	Salmonidae
CA381586	Nr1d4b2	Rainbow trout	*Oncorhynchus mykiss*	Protacanthopterygii	Salmoniformes	Salmonidae
EV383714	Nr1d4b	Sockeye salmon	*Oncorhynchus nerka*	Protacanthopterygii	Salmoniformes	Salmonidae
AGKD01073284	Nr1d4b1	Atlantic salmon	*Salmo salar*	Protacanthopterygii	Salmoniformes	Salmonidae
AGKD01142222	Nr1d4b2	Atlantic salmon	*Salmo salar*	Protacanthopterygii	Salmoniformes	Salmonidae
DT263494	Nr1d4b	Fathead minnow	*Pimephales promelas*	Ostariophysi	Cypriniformes	Cyprinidae
ENSDARP00000076910	Nr1d4b	Zebrafish	*Danio rerio*	Ostariophysi	Cypriniformes	Cyprinidae
AHAT01018834/AHAT01018831/AHAT01018830	Nr1d4	Spotted gar	*Lepisosteus oculatus*	Holostei	Semionotiformes	Lepisosteidae
ENSPMAP00000009330	Nr1d1	Sea lamprey	*Petromyzon marinus*	Hyperoartia	Petromyzontiformes	Petromyzontidae
APJL01048006	Nr1d1	Arctic lamprey	*Lethenteron camtschaticum*	Hyperoartia	Petromyzontiformes	Petromyzontidae
ENSPMAP00000000579	Nr1d2	Sea lamprey	*Petromyzon marinus*	Hyperoartia	Petromyzontiformes	Petromyzontidae
APJL01026296	Nr1d2	Arctic lamprey	*Lethenteron camtschaticum*	Hyperoartia	Petromyzontiformes	Petromyzontidae
ENSCINP00000004784	Nr1d	Vase tunicate	*Ciona intestinalis*	Tunicata	Enterogona	Cionidae
ENSCSAVP00000009501	Nr1d	Pacific transparent sea squirt	*Ciona savignyi*	Tunicata	Enterogona	Cionidae
XP_002598635	Nr1d	Florida lancelet	*Branchiostoma floridae*	Cephalochordata	Amphioxiformes	Branchiostomidae
XP_002735636	Nr1d	Acorn worm	*Saccoglossus kowalevskii*	Hemichordata		Harrimaniidae
XP_783869	Nr1d	Purple sea urchin	*Strongylocentrotus purpuratus*	Echinodermata	Echinozoa	Strongylocentrotidae
AM596899	Nr1d	Common urchin	*Paracentrotus lividus*	Echinodermata	Echinozoa	Echinidae
AGCV01187286	Nr1d	Green sea urchin	*Lytechinus variegatus*	Echinodermata	Echinozoa	Toxopneustidae

NR1F1 (RORA)

ENSP00000402971	ROR A	Human	*Homo sapiens*	Euarchontoglires	Primates	Hominidae
ENSMUSP00000109254	ROR A	Mouse	*Mus musculus*	Euarchontoglires	Rodentia	Muridae
ENSECAP00000014205	ROR A	Horse	*Equus caballus*	Laurasiatheria	Perissodactyla	Equidae
ENSCAFP00000024559	ROR A	Dog	*Canis lupus familiaris*	Laurasiatheria	Carnivora	Canidae
ENSLAFP00000000483	ROR A	African savanna elephant	*Loxodonta africana*	Afrotheria	Proboscidea	Elephantidae
ENSSHAP00000018534	ROR A	Tasmanian devil	*Sarcophilus harrisii*	Metatheria	Dasyuromorphia	Dasyuridae
ENSMODP00000014431	ROR A	Gray short-tailed opossum	*Monodelphis domestica*	Metatheria	Didelphimorphia	Didelphidae
ENSOANP00000019532	ROR A	Platypus	*Ornithorhynchus anatinus*	Prototheria	Monotremata	Ornithorhynchidae
ENSGALP00000005957	ROR A	Chicken	*Gallus gallus*	Aves	Galliformes	Phasianidae
ENSMGAP00000004299	ROR A	Turkey	*Meleagris gallopavo*	Aves	Galliformes	Phasianidae
ENSAPLP00000005445	ROR A	Mallard	*Anas platyrhynchos*	Aves	Anseriformes	Anatidae
AGAI01053894	ROR A	Budgerigar	*Melopsittacus undulatus*	Aves	Psittaciformes	Psittacidae
ENSTGUP00000005503	ROR A	Zebra finch	*Taeniopygia guttata*	Aves	Passeriformes	Estrildidae
AKHW01008577	ROR A	American alligator	*Alligator mississippiensis*	Archosauria	Crocodilia	Crocodylidae
AGCU01176435/AGCU01176436	ROR A	Chinese softshell turtle	*Pelodiscus sinensis*	Sauropsida	Testudines	Trionychidae
AHGY01234675/AHGY01234674/AHGY01234673	ROR A	Western painted turtle	*Chrysemys picta bellii*	Sauropsida	Testudines	Emydidae
ENSACAP00000015648	ROR A	Green anole	*Anolis carolinensis*	Lepidosauria	Squamata	Iguanidae
AEQU010211683/AEQU010218000/AEQU010100612/ AEQU010275267/AEQU010342246/AEQU010272887/ AEQU010037329	ROR A	Burmese python	*Python molurus bivittatus*	Lepidosauria	Squamata	Pythonidae
ENSXETP00000045670	ROR A	Western clawed frog	*Xenopus (Silurana) tropicalis*	Amphibia	Anura	Pipidae
AFYH01009639/AFYH01009640/AFYH01009641/ ENSLACP00000019843	Ror a	Coelacanth	*Latimeria chalumnae*	Actinistia	Coelacanthiformes	Coelacanthidae

ENSTRUP00000026635	Ror aa	Torafugu	*Takifugu rubripes*	Acanthopterygii	Tetraodontiformes	Tetraodontidae
ENSTNIP00000011225	Ror aa	Green-spotted pufferfish	*Tetraodon nigroviridis*	Acanthopterygii	Tetraodontiformes	Tetraodontidae
GO576981	Ror aa	Yellow perch	*Perca flavescens*	Acanthopterygii	Perciformes	Percidae
ENSONIP00000019240	Ror aa	Nile tilapia	*Oreochromis niloticus*	Acanthopterygii	Perciformes	Cichlidae
AFNZ01016703	Ror aa	Burton's mouthbrooder	*Haplochromis burtoni*	Acanthopterygii	Perciformes	Cichlidae
ENSGACP00000014125	Ror aa	Three-spined stickleback	*Gasterosteus aculeatus*	Acanthopterygii	Gasterosteiformes	Gasterosteidae
ENSORLP00000009581	Ror aa	Japanese medaka	*Oryzias latipes*	Acanthopterygii	Beloniformes	Adrianichthyidae
ENSXMAP00000003249	Ror aa	Southern platyfish	*Xiphophorus maculatus*	Acanthopterygii	Cyprinodontiformes	Poeciliidae
ENSGMOP00000007376	Ror aa	Atlantic cod	*Gadus morhua*	Paracanthopterygii	Gadiformes	Gadidae
AGKD01039184	Ror aa1	Atlantic salmon	*Salmo salar*	Protacanthopterygii	Salmoniformes	Salmonidae
AGKD01017699	Ror aa2	Atlantic salmon	*Salmo salar*	Protacanthopterygii	Salmoniformes	Salmonidae
AM926578	Ror aa	Goldfish	*Carassius auratus*	Ostariophysi	Cypriniformes	Cyprinidae
EX823541	Ror aa	Common carp	*Cyprinus carpio*	Ostariophysi	Cypriniformes	Cyprinidae
AFC34772	Ror aa	Grass carp	*Ctenopharyngodon idella*	Ostariophysi	Cypriniformes	Cyprinidae
DT239351	Ror aa	Fathead minnow	*Pimephales promelas*	Ostariophysi	Cypriniformes	Cyprinidae
ENSDARP00000028873	Ror aa	Zebrafish	*Danio rerio*	Ostariophysi	Cypriniformes	Cyprinidae
ENSONIP00000019631	Ror ab	Nile tilapia	*Oreochromis niloticus*	Acanthopterygii	Perciformes	Cichlidae
AFNZ01036867/AFNZ01036868/AFNZ01036870	Ror ab	Burton's mouthbrooder	*Haplochromis burtoni*	Acanthopterygii	Perciformes	Cichlidae
ENSXMAP00000018498	Ror ab	Southern platyfish	*Xiphophorus maculatus*	Acanthopterygii	Cyprinodontiformes	Poeciliidae
AGKD01343283/AGKD01381530/AGKD01451997/ AGKD01108231	Ror ab	Atlantic salmon	*Salmo salar*	Protacanthopterygii	Salmoniformes	Salmonidae
AFC34771	Ror ab	Grass carp	*Ctenopharyngodon idella*	Ostariophysi	Cypriniformes	Cyprinidae
DT204095	Ror ab	Fathead minnow	*Pimephales promelas*	Ostariophysi	Cypriniformes	Cyprinidae
ENSDARP00000015111	Ror ab	Zebrafish	*Danio rerio*	Ostariophysi	Cypriniformes	Cyprinidae
AHAT01004926	Ror a	Spotted gar	*Lepisosteus oculatus*	Holostei	Semionotiformes	Lepisosteidae
AESE011450971/AESE010801300/AESE010121687/ AESE010202287/AESE012499234/AESE010685891/ AESE012563713/AESE012499234	Ror a	Little skate	*Leucoraja erinacea*	Chondrichthyes	Rajiformes	Rajidae
AAVX01528546/AAVX01019807/AAVX01096577/ AAVX01264271/AAVX01116599/AAVX01223540	Ror a	Ghost shark	*Callorhinchus milii*	Chondrichthyes	Chimaeriformes	Callorhinchidae
**NR1F2 (RORB)**						
ENSP00000366093	ROR B	Human	*Homo sapiens*	Euarchontoglires	Primates	Hominidae
ENSMUSP00000108451	ROR B	Mouse	*Mus musculus*	Euarchontoglires	Rodentia	Muridae
ENSCAFP00000031876	ROR B	Dog	*Canis lupus familiaris*	Laurasiatheria	Carnivora	Canidae
ENSECAP00000021862	ROR B	Horse	*Equus caballus*	Laurasiatheria	Perissodactyla	Equidae
ENSLAFP00000010666	ROR B	African savanna elephant	*Loxodonta africana*	Afrotheria	Proboscidea	Elephantidae
ENSSHAP00000022087	ROR B	Tasmanian devil	*Sarcophilus harrisii*	Metatheria	Dasyuromorphia	Dasyuridae
ENSMODP00000002493	ROR B	Gray short-tailed opossum	*Monodelphis domestica*	Metatheria	Didelphimorphia	Didelphidae
ENSOANP00000018466	ROR B	Platypus	*Ornithorhynchus anatinus*	Prototheria	Monotremata	Ornithorhynchida[illegible]
ENSGALP00000024399/ENSGALP00000024400	ROR B	Chicken	*Gallus gallus*	Aves	Galliformes	Phasianidae
ENSMGAP00000005896/ENSMGAP00000005892	ROR B	Turkey	*Meleagris gallopavo*	Aves	Galliformes	Phasianidae
ENSAPLP00000006801/ENSAPLP00000012858	ROR B	Mallard	*Anas platyrhynchos*	Aves	Anseriformes	Anatidae
AGAI01070642/AGAI01070643/AGAI01070644	ROR B	Budgerigar	*Melopsittacus undulatus*	Aves	Psittaciformes	Psittacidae
ENSTGUP00000000804	ROR B	Zebra finch	*Taeniopygia guttata*	Aves	Passeriformes	Estrildidae
AKHW01085565/AKHW01085566/AKHW01085568	ROR B	American alligator	*Alligator mississippiensis*	Archosauria	Crocodilia	Crocodylidae
AGCU01194454/AGCU01194450/AGCU01194447	ROR B	Chinese softshell turtle	*Pelodiscus sinensis*	Sauropsida	Testudines	Trionychidae
AHGY01542635/AHGY01542632/AHGY01542631	ROR B	Western painted turtle	*Chrysemys picta bellii*	Sauropsida	Testudines	Emydidae
ENSACAP00000005097	ROR B	Green anole	*Anolis carolinensis*	Lepidosauria	Squamata	Iguanidae
AEQU010393258/AEQU010067583/AEQU010390205/ AEQU010327093/AEQU010380194	ROR B	Burmese python	*Python molurus bivittatus*	Lepidosauria	Squamata	Pythonidae
ENSXETP00000045673	ROR B	Western clawed frog	*Xenopus (Silurana) tropicalis*	Amphibia	Anura	Pipidae
AFYH01160664/AFYH01160666/AFYH01160667/ AFYH01160668/ENSLACP00000006876	Ror b	Coelacanth	*Latimeria chalumnae*	Actinistia	Coelacanthiformes	Coelacanthidae
ENSTRUP00000006772	Ror b	Torafugu	*Takifugu rubripes*	Acanthopterygii	Tetraodontiformes	Tetraodontidae
ENSTNIP00000017712	Ror b	Green-spotted pufferfish	*Tetraodon nigroviridis*	Acanthopterygii	Tetraodontiformes	Tetraodontidae
ENSONIP00000013529/ENSONIP00000013530	Ror b	Nile tilapia	*Oreochromis niloticus*	Acanthopterygii	Perciformes	Cichlidae
AFNZ01010444	Ror b	Burton's mouthbrooder	*Haplochromis burtoni*	Acanthopterygii	Perciformes	Cichlidae
ENSGACP00000015289	Ror b	Three-spined stickleback	*Gasterosteus aculeatus*	Acanthopterygii	Gasterosteiformes	Gasterosteidae
ENSORLP00000015578	Ror b	Japanese medaka	*Oryzias latipes*	Acanthopterygii	Beloniformes	Adrianichthyidae
ENSXMAP00000015617	Ror b	Southern platyfish	*Xiphophorus maculatus*	Acanthopterygii	Cyprinodontiformes	Poeciliidae
CAEA01245277/ENSGMOP00000017693	Ror b	Atlantic cod	*Gadus morhua*	Paracanthopterygii	Gadiformes	Gadidae
BX864215	Ror b	Rainbow trout	*Oncorhynchus mykiss*	Protacanthopterygii	Salmoniformes	Salmonidae
AGKD01347468/AGKD01136849/AGKD01215889	Ror b1	Atlantic salmon	*Salmo salar*	Protacanthopterygii	Salmoniformes	Salmonidae
AGKD01066609/AGKD01026682	Ror b2	Atlantic salmon	*Salmo salar*	Protacanthopterygii	Salmoniformes	Salmonidae
DT203485	Ror b	Fathead minnow	*Pimephales promelas*	Ostariophysi	Cypriniformes	Cyprinidae
ENSDARP00000084181	Ror b	Zebrafish	*Danio rerio*	Ostariophysi	Cypriniformes	Cyprinidae
AHAT01002966	Ror b	Spotted gar	*Lepisosteus oculatus*	Holostei	Semionotiformes	Lepisosteidae

AESE011535637/AESE011606955/AESE010164811/ AESE010224634/AESE010062338/AESE012529471/ AESE010000504/AESE012612965/AESE010089656	Rorb	Little skate	*Leucoraja erinacea*	Chondrichthyes	Rajiformes	Rajidae
AAVX01169300/AAVX01535051/AAVX01358530/AAVX01 326383/AAVX01465090/AAVX01177560	Rorb	Ghost shark	*Callorhinchus milii*	Chondrichthyes	Chimaeriformes	Callorhinchidae

NR1F3 (RORC)

ENSP00000349164	RORC	Human	*Homo sapiens*	Euarchontoglires	Primates	Hominidae
ENSMUSP00000102913	RORC	Mouse	*Mus musculus*	Euarchontoglires	Rodentia	Muridae
ENSCAFP00000019006	RORC	Dog	*Canis lupus familiaris*	Laurasiatheria	Carnivora	Canidae
ENSECAP00000001630	RORC	Horse	*Equus caballus*	Laurasiatheria	Perissodactyla	Equidae
ENSLAFP00000015850	RORC	African savanna elephant	*Loxodonta africana*	Afrotheria	Proboscidea	Elephantidae
ENSSHAP00000018833	RORC	Tasmanian devil	*Sarcophilus harrisii*	Metatheria	Dasyuromorphia	Dasyuridae
ENSMODP00000005497	RORC	Gray short-tailed opossum	*Monodelphis domestica*	Metatheria	Didelphimorphia	Didelphidae
ENSOANP00000007144	RORC	Platypus	*Ornithorhynchus anatinus*	Prototheria	Monotremata	Ornithorhynchida
AADN03008343/XP_003642756	RORC	Chicken	*Gallus gallus*	Aves	Galliformes	Phasianidae
AGAI01012738	RORC	Budgerigar	*Melopsittacus undulatus*	Aves	Psittaciformes	Psittacidae
AKHW01088548	RORC	American alligator	*Alligator mississippiensis*	Archosauria	Crocodilia	Crocodylidae
AGCU01060020/AGCU01060019/AGCU01060018	RORC	Chinese softshell turtle	*Pelodiscus sinensis*	Sauropsida	Testudines	Trionychidae
AHGY01124572/AHGY01124573	RORC	Western painted turtle	*Chrysemys picta bellii*	Sauropsida	Testudines	Emydidae
ENSACAP00000004116	RORC	Green anole	*Anolis carolinensis*	Lepidosauria	Squamata	Iguanidae
AEQU010862291/AEQU010654006/AEQU010321922/ AEQU010026695	RORC	Burmese python	*Python molurus bivittatus*	Lepidosauria	Squamata	Pythonidae
NP_001088389	RORC	African clawed frog	*Xenopus laevis*	Amphibia	Anura	Pipidae
ENSXETP00000004549	RORC	Western clawed frog	*Xenopus (Silurana) tropicalis*	Amphibia	Anura	Pipidae
AFYH01036420/AFYH01036419/AFYH01036418/ AFYH01036417/ENSLACP00000020228	Rorc	Coelacanth	*Latimeria chalumnae*	Actinistia	Coelacanthiformes	Coelacanthidae
ENSTRUP00000003306	Rorc	Torafugu	*Takifugu rubripes*	Acanthopterygii	Tetraodontiformes	Tetraodontidae
ENSTNIP00000021515	Rorc	Green-spotted pufferfish	*Tetraodon nigroviridis*	Acanthopterygii	Tetraodontiformes	Tetraodontidae
CABK01029953/CABK01010475	Rorc	European seabass	*Dicentrarchus labrax*	Acanthopterygii	Perciformes	Moronidae
ENSONIP00000007839	Rorc	Nile tilapia	*Oreochromis niloticus*	Acanthopterygii	Perciformes	Cichlidae
AFNZ01026144	Rorc	Burton's mouthbrooder	*Haplochromis burtoni*	Acanthopterygii	Perciformes	Cichlidae
ENSGACP00000016191	Rorc	Three-spined stickleback	*Gasterosteus aculeatus*	Acanthopterygii	Gasterosteiformes	Gasterosteidae
ENSORLP00000011888	Rorc	Japanese medaka	*Oryzias latipes*	Acanthopterygii	Beloniformes	Adrianichthyidae
DR007798	Rorc	Common mummichog	*Fundulus heteroclitus*	Acanthopterygii	Cyprinodontiformes	Fundulidae
ENSXMAP00000007026	Rorc	Southern platyfish	*Xiphophorus maculatus*	Acanthopterygii	Cyprinodontiformes	Poeciliidae
ENSGMOP00000008579	Rorc	Atlantic cod	*Gadus morhua*	Paracanthopterygii	Gadiformes	Gadidae
EL541809	Rorc	Rainbow smelt	*Osmerus mordax*	Protacanthopterygii	Osmeriformes	Osmeridae
AGKD01121002	Rorc1	Atlantic salmon	*Salmo salar*	Protacanthopterygii	Salmoniformes	Salmonidae
AGKD01125160/AGKD01251413	Rorc2	Atlantic salmon	*Salmo salar*	Protacanthopterygii	Salmoniformes	Salmonidae
DT176112	Rorc	Fathead minnow	*Pimephales promelas*	Ostariophysi	Cypriniformes	Cyprinidae
ENSDARP00000109744	Rorc	Zebrafish	*Danio rerio*	Ostariophysi	Cypriniformes	Cyprinidae
AHAT01019981/AHAT01019980	Rorc	Spotted gar	*Lepisosteus oculatus*	Holostei	Semionotiformes	Lepisosteidae
AESE011521902/AESE011651149/AESE012827479/ AESE010001877/AESE010017334/AESE012830006	Rorc	Little skate	*Leucoraja erinacea*	Chondrichthyes	Rajiformes	Rajidae

RORD

FY702974	RORD	Tammar wallaby	*Macropus eugenii*	Metatheria	Diprotodontia	Macropodidae
AFEY01062175/AFEY01396210	RORD	Tasmanian devil	*Sarcophilus harrisii*	Metatheria	Dasyuromorphia	Dasyuridae
ENSMODP00000005498	RORD	Gray short-tailed opossum	*Monodelphis domestica*	Metatheria	Didelphimorphia	Didelphidae
ENSGALP00000001531	RORD	Chicken	*Gallus gallus*	Aves	Galliformes	Phasianidae
ENSMGAP00000001793	RORD	Turkey	*Meleagris gallopavo*	Aves	Galliformes	Phasianidae
ENSAPLP00000011268	RORD	Mallard	*Anas platyrhynchos*	Aves	Anseriformes	Anatidae
AGAI01044943	RORD	Budgerigar	*Melopsittacus undulatus*	Aves	Psittaciformes	Psittacidae
AKZB01035031/AKZB01035030/AKZB01035029	RORD	Medium ground finch	*Geospiza fortis*	Aves	Passeriformes	Fringillidae
ABQF01047554/ABQF01047552	RORD	Zebra finch	*Taeniopygia guttata*	Aves	Passeriformes	Estrildidae
AKHW01007024/AKHW01007025	RORD	American alligator	*Alligator mississippiensis*	Archosauria	Crocodilia	Crocodylidae
AGCU01126837/AGCU01126836	RORD	Chinese softshell turtle	*Pelodiscus sinensis*	Sauropsida	Testudines	Trionychidae
AHGY01219351	RORD	Western painted turtle	*Chrysemys picta bellii*	Sauropsida	Testudines	Emydidae
AEQU011134043/AEQU010188413/AEQU010182778/ AEQU010209170/AEQU010145426/AEQU010421123/ AEQU011132866/AEQU010126917/AEQU011142376	RORD	Burmese python	*Python molurus bivittatus*	Lepidosauria	Squamata	Pythonidae
ENSXETP00000017869	RORD	Western clawed frog	*Xenopus (Silurana) tropicalis*	Amphibia	Anura	Pipidae
AFYH01051191/AFYH01051190/ENSLACP00000014548	Rord	Coelacanth	*Latimeria chalumnae*	Actinistia	Coelacanthiformes	Coelacanthidae
ENSTRUP00000007474	Rorda	Torafugu	*Takifugu rubripes*	Acanthopterygii	Tetraodontiformes	Tetraodontidae
ENSTNIP00000013605	Rorda	Green-spotted pufferfish	*Tetraodon nigroviridis*	Acanthopterygii	Tetraodontiformes	Tetraodontidae
ENSONIP00000005902	Rorda	Nile tilapia	*Oreochromis niloticus*	Acanthopterygii	Perciformes	Cichlidae
AFNZ01021687	Rorda	Burton's mouthbrooder	*Haplochromis burtoni*	Acanthopterygii	Perciformes	Cichlidae
ENSGACP00000016239	Rorda	Three-spined stickleback	*Gasterosteus aculeatus*	Acanthopterygii	Gasterosteiformes	Gasterosteidae
ENSORLP00000004691	Rorda	Japanese medaka	*Oryzias latipes*	Acanthopterygii	Beloniformes	Adrianichthyidae

ENSXMAP00000013069	Ror da	Southern platyfish	*Xiphophorus maculatus*	Acanthopterygii	Cyprinodontiformes	Poeciliidae
ENSGMOP00000000786	Ror da	Atlantic cod	*Gadus morhua*	Paracanthopterygii	Gadiformes	Gadidae
NP_001186756	Ror da	Rainbow trout	*Oncorhynchus mykiss*	Protacanthopterygii	Salmoniformes	Salmonidae
EL559998	Ror da	Chinook salmon	*Oncorhynchus tshawytscha*	Protacanthopterygii	Salmoniformes	Salmonidae
AGKD01063391	Ror da1	Atlantic salmon	*Salmo salar*	Protacanthopterygii	Salmoniformes	Salmonidae
AGKD01085658	Ror da2	Atlantic salmon	*Salmo salar*	Protacanthopterygii	Salmoniformes	Salmonidae
FF839397	Ror da	Grayling	*Thymallus thymallus*	Protacanthopterygii	Salmoniformes	Salmonidae
AFC34773	Ror da	Grass carp	*Ctenopharyngodon idella*	Ostariophysi	Cypriniformes	Cyprinidae
ENSDARP00000091257	Ror da	Zebrafish	*Danio rerio*	Ostariophysi	Cypriniformes	Cyprinidae
ENSTRUP00000013422	Ror db	Torafugu	*Takifugu rubripes*	Acanthopterygii	Tetraodontiformes	Tetraodontidae
ENSTNIP00000018401	Ror db	Green-spotted pufferfish	*Tetraodon nigroviridis*	Acanthopterygii	Tetraodontiformes	Tetraodontidae
EB032539	Ror db	Atlantic halibut	*Hippoglossus hippoglossus*	Acanthopterygii	Pleuronectiformes	Pleuronectidae
ENSONIP00000012885/ENSONIP00000012886	Ror db	Nile tilapia	*Oreochromis niloticus*	Acanthopterygii	Perciformes	Cichlidae
DY629218/AFNZ01008195	Ror db	Burton's mouthbrooder	*Haplochromis burtoni*	Acanthopterygii	Perciformes	Cichlidae
ENSGACP00000020235	Ror db	Three-spined stickleback	*Gasterosteus aculeatus*	Acanthopterygii	Gasterosteiformes	Gasterosteidae
BAAF04030292/BAAF04030291/BAAF04030290/ BAAF04030289	Ror db	Japanese medaka	*Oryzias latipes*	Acanthopterygii	Beloniformes	Adrianichthyidae
ENSXMAP00000019345	Ror db	Southern platyfish	*Xiphophorus maculatus*	Acanthopterygii	Cyprinodontiformes	Poeciliidae
CAEA01084121	Ror db	Atlantic cod	*Gadus morhua*	Paracanthopterygii	Gadiformes	Gadidae
AGKD01001004	Ror db1	Atlantic salmon	*Salmo salar*	Protacanthopterygii	Salmoniformes	Salmonidae
AGKD01061152	Ror db2	Atlantic salmon	*Salmo salar*	Protacanthopterygii	Salmoniformes	Salmonidae
AFC34774	Ror db	Grass carp	*Ctenopharyngodon idella*	Ostariophysi	Cypriniformes	Cyprinidae
ENSDARP00000019810	Ror db	Zebrafish	*Danio rerio*	Ostariophysi	Cypriniformes	Cyprinidae
AHAT01010728	Ror d	Spotted gar	*Lepisosteus oculatus*	Holostei	Semionotiformes	Lepisosteidae
AESE012584459/AESE012476930/AESE010208767/ AESE011012068/AESE012678113/AESE011771814/ AESE010002806	Ror d	Little skate	*Leucoraja erinacea*	Chondrichthyes	Rajiformes	Rajidae
AAVX01577397/AAVX01241991/AAVX01294515/ AAVX01167838/AAVX01235766	Ror d	Ghost shark	*Callorhinchus milii*	Chondrichthyes	Chimaeriformes	Callorhinchidae
ENSPMAP00000010218	Ror	Sea lamprey	*Petromyzon marinus*	Hyperoartia	Petromyzontiformes	Petromyzontidae
APJL01013440	Ror	Arctic lamprey	*Lethenteron camtschaticum*	Hyperoartia	Petromyzontiformes	Petromyzontidae
ENSCINP00000022358	Ror	Vase tunicate	*Ciona intestinalis*	Tunicata	Enterogona	Cionidae
ENSCSAVP00000016983	Ror	Pacific transparent sea squirt	*Ciona savignyi*	Tunicata	Enterogona	Cionidae
XP_002597918	Ror-like	Florida lancelet	*Branchiostoma floridae*	Cephalochordata	Amphioxiformes	Branchiostomidae
XP_784691	Ror	Purple sea urchin	*Strongylocentrotus purpuratus*	Echinodermata	Echinozoa	Strongylocentroti ae
AGCV01372889/AGCV01372888/AGCV01372887	Ror	Green sea urchin	*Lytechinus variegatus*	Echinodermata	Echinozoa	Toxopneustidae
AKZP01068386/AKZP01068388	Ror	Bat star	*Patiria miniata*	Echinodermata	Asterozoa	Asterinidae

NR1I3 (CAR)

ENSP00000356959	CAR	Human	*Homo sapiens*	Euarchontoglires	Primates	Hominidae
ENSMUSP00000106960	CAR	Mouse	*Mus musculus*	Euarchontoglires	Rodentia	Muridae
ENSRNOP00000048836	CAR	Norway rat	*Rattus norvegicus*	Euarchontoglires	Rodentia	Muridae
ENSSTOP00000003417	CAR	Thirteen-lined ground squirrel	*Ictidomys tridecemlineatus*	Euarchontoglires	Rodentia	Sciuridae
XP_002715180	CAR	American pika	*Ochotona princeps*	Laurasiatheria	Lagomorpha	Ochotonidae
XP_002715180	CAR	Rabbit	*Oryctolagus cuniculus*	Euarchontoglires	Lagomorpha	Leporidae
ENSCAFP00000013438	CAR	Dog	*Canis lupus familiaris*	Laurasiatheria	Carnivora	Canidae
ENSECAP00000018532	CAR	Horse	*Equus caballus*	Laurasiatheria	Perissodactyla	Equidae
ENSPCAP00000013010	CAR	Cape rock hyrax	*Procavia capensis*	Afrotheria	Hyracoidea	Procaviidae
ENSLAFP00000000995	CAR	African savanna elephant	*Loxodonta africana*	Afrotheria	Proboscidea	Elephantidae
ENSSHAP00000019655	CAR	Tasmanian devil	*Sarcophilus harrisii*	Metatheria	Dasyuromorphia	Dasyuridae
ENSMODP00000006263	CAR	Gray short-tailed opossum	*Monodelphis domestica*	Metatheria	Didelphimorphia	Didelphidae
NP_990033	CAR	Chicken	*Gallus gallus*	Aves	Galliformes	Phasianidae
ENSMGAP00000005652	PXR	Turkey	*Meleagris gallopavo*	Aves	Galliformes	Phasianidae
BAF57043	CAR	Japanese quail	*Coturnix japonica*	Aves	Galliformes	Phasianidae
ENSAPLP00000001100	CAR	Mallard	*Anas platyrhynchos*	Aves	Anseriformes	Anatidae
AKMT01010461	CAR	Peregrin falcon	*Falco peregrinus*	Aves	Falconiformes	Falconidae
AGAI01015828	CAR	Budgerigar	*Melopsittacus undulatus*	Aves	Psittaciformes	Psittacidae
AKHW01087431	CAR	American alligator	*Alligator mississippiensis*	Archosauria	Crocodilia	Crocodylidae
ENSPSIP00000004770	CAR	Chinese softshell turtle	*Pelodiscus sinensis*	Sauropsida	Testudines	Trionychidae
JW436047	CAR	Red-eared slider	*Trachemys scripta elegans*	Sauropsida	Testudines	Emydidae
AHGY01125513/AHGY01125512/AHGY01125511	CAR	Western painted turtle	*Chrysemys picta bellii*	Sauropsida	Testudines	Emydidae
ENSACAP00000001463/ENSACAP00000010788	CAR	Green anole	*Anolis carolinensis*	Lepidosauria	Squamata	Iguanidae
AEQU010835272/AEQU010118400/AEQU010802902/ AEQU011186585/AEQU011010690/AEQU010127380	CAR	Burmese python	*Python molurus bivittatus*	Lepidosauria	Squamata	Pythonidae
ADW81976	CAR	African clawed frog	*Xenopus laevis*	Amphibia	Anura	Pipidae
XP_002941189	CAR	Western clawed frog	*Xenopus (Silurana) tropicalis*	Amphibia	Anura	Pipidae
ENSLACP00000011197	Car	Coelacanth	*Latimeria chalumnae*	Actinistia	Coelacanthiformes	Coelacanthidae

NR1I2 (PXR)

ENSP00000377319	PXR	Human	*Homo sapiens*	Euarchontoglires	Primates	Hominidae
ENSMUSP00000023504	PXR	Mouse	*Mus musculus*	Euarchontoglires	Rodentia	Muridae
ENSRNOP00000003934	PXR	Norway rat	*Rattus norvegicus*	Euarchontoglires	Rodentia	Muridae
ENSSTOP00000013428	PXR	Thirteen-lined ground squirrel	*Ictidomys tridecemlineatus*	Euarchontoglires	Rodentia	Sciuridae
ENSOCUP00000005307	PXR	Rabbit	*Oryctolagus cuniculus*	Euarchontoglires	Lagomorpha	Leporidae
ENSCAFP00000016344	PXR	Dog	*Canis lupus familiaris*	Laurasiatheria	Carnivora	Canidae
ENSECAP00000007024	PXR	Horse	*Equus caballus*	Laurasiatheria	Perissodactyla	Equidae
ENSLAFP00000006570	PXR	African savanna elephant	*Loxodonta africana*	Afrotheria	Proboscidea	Elephantidae
ENSSHAP00000005072	PXR	Tasmanian devil	*Sarcophilus harrisii*	Metatheria	Dasyuromorphia	Dasyuridae
EC354810	PXR	Common brushtail	*Trichosurus vulpecula*	Metatheria	Diprotodontia	Phalangeridae
ENSMODP00000037046	PXR	Gray short-tailed opossum	*Monodelphis domestica*	Metatheria	Didelphimorphia	Didelphidae
NP_001083606	PXR	African clawed frog	*Xenopus laevis*	Amphibia	Anura	Pipidae
ENSXETP00000039111	PXR	Western clawed frog	*Xenopus (Silurana) tropicalis*	Amphibia	Anura	Pipidae
ENSLACP00000011739	Pxr	Coelacanth	*Latimeria chalumnae*	Actinistia	Coelacanthiformes	Coelacanthidae
ENSTRUP00000007607	Pxr	Torafugu	*Takifugu rubripes*	Acanthopterygii	Tetraodontiformes	Tetraodontidae
ENSTNIP00000016937	Pxr	Green-spotted pufferfish	*Tetraodon nigroviridis*	Acanthopterygii	Tetraodontiformes	Tetraodontidae
FN566078	Pxr	European seabass	*Dicentrarchus labrax*	Acanthopterygii	Perciformes	Moronidae
JG740513	Pxr	Barramundi perch	*Lates calcarifer*	Acanthopterygii	Perciformes	Latidae
ENSONIP00000018088/ENSONIP00000018089	Pxr	Nile tilapia	*Oreochromis niloticus*	Acanthopterygii	Perciformes	Cichlidae
AFNZ01039003	Pxr	Burton's mouthbrooder	*Haplochromis burtoni*	Acanthopterygii	Perciformes	Cichlidae
GE817211	Pxr	Copper rockfish	*Sebastes caurinus*	Acanthopterygii	Scorpaeniformes	Sebastinae
ENSORLP00000022472	Pxr	Japanese medaka	*Oryzias latipes*	Acanthopterygii	Beloniformes	Adrianichthyidae
JW596740	Pxr	Gulf killifish	*Fundulus grandis*	Acanthopterygii	Cyprinodontiformes	Fundulidae
GAIB01011783	Pxr	Turquoise killifish	*Nothobranchius furzeri*	Acanthopterygii	Cyprinodontiformes	Nothobranchiidae
ENSXMAP00000004022	Pxr	Southern platyfish	*Xiphophorus maculatus*	Acanthopterygii	Cyprinodontiformes	Poeciliidae
ACP30432	Pxr	Delta smelt	*Hypomesus transpacificus*	Protacanthopterygii	Osmeriformes	Osmeridae
NP_001118144	Pxr	Rainbow trout	*Oncorhynchus mykiss*	Protacanthopterygii	Salmoniformes	Salmonidae
AGKD01060130/AGKD01072048/AGKD01056898	Pxr	Atlantic salmon	*Salmo salar*	Protacanthopterygii	Salmoniformes	Salmonidae
EE393911	Pxr	Rare gudgeon	*Gobiocypris rarus*	Ostariophysi	Cypriniformes	Cyprinidae
ENSDARP00000061524	Pxr	Zebrafish	*Danio rerio*	Ostariophysi	Cypriniformes	Cyprinidae
ADL27416	Pxr	Grass carp	*Ctenopharyngodon idella*	Ostariophysi	Cypriniformes	Cyprinidae
JT399788	Pxr	Channel catfish	*Ictalurus punctatus*	Ostariophysi	Siluriformes	Ictaluridae
AHAT01033742/AHAT01033741	Pxr	Spotted gar	*Lepisosteus oculatus*	Holostei	Semionotiformes	Lepisosteidae
AAVX02014085	Pxr	Ghost shark	*Callorhinchus milii*	Chondrichthyes	Chimaeriformes	Callorhinchidae

NR1I1 (VDR)

ENSP00000378734	VDR	Human	*Homo sapiens*	Euarchontoglires	Primates	Hominidae
ENSMUSP00000023119	VDR	Mouse	*Mus musculus*	Euarchontoglires	Rodentia	Muridae
ENSCAFP00000013418	VDR	Dog	*Canis lupus familiaris*	Laurasiatheria	Carnivora	Canidae
ENSECAP00000013634	VDR	Horse	*Equus caballus*	Laurasiatheria	Perissodactyla	Equidae
ENSPCAP00000007208	VDR	Cape rock hyrax	*Procavia capensis*	Afrotheria	Hyracoidea	Procaviidae
ENSLAFP00000005389	VDR	African savanna elephant	*Loxodonta africana*	Afrotheria	Proboscidea	Elephantidae
ENSSHAP00000001133	VDR	Tasmanian devil	*Sarcophilus harrisii*	Metatheria	Dasyuromorphia	Dasyuridae
XP_001378022	VDR	Gray short-tailed opossum	*Monodelphis domestica*	Metatheria	Didelphimorphia	Didelphidae
ENSOANP00000018058	VDR	Platypus	*Ornithorhynchus anatinus*	Prototheria	Monotremata	Ornithorhynchidae
AADN03018515	VDR	Chicken	*Gallus gallus*	Aves	Galliformes	Phasianidae
ENSMGAP00000004709	VDR	Turkey	*Meleagris gallopavo*	Aves	Galliformes	Phasianidae
AGAI01011058	VDR	Budgerigar	*Melopsittacus undulatus*	Aves	Psittaciformes	Psittacidae
AKZB01057355/AKZB01057356/AKZB01057357	VDR	Medium ground finch	*Geospiza fortis*	Aves	Passeriformes	Fringillidae
ENSTGUP00000017389	VDR	Zebra finch	*Taeniopygia guttata*	Aves	Passeriformes	Estrildidae
CAB56417	VDR	Nile crocodile	*Crocodylus niloticus*	Archosauria	Crocodilia	Crocodylidae
AKHW01080792/AKHW01080794	VDR	American alligator	*Alligator mississippiensis*	Archosauria	Crocodilia	Crocodylidae
ENSPSIP00000020473	VDR	Chinese softshell turtle	*Pelodiscus sinensis*	Sauropsida	Testudines	Trionychidae
CAC69550	VDR	Red-eared slider	*Trachemys scripta elegans*	Sauropsida	Testudines	Emydidae
AHGY01384694/AHGY01384690/AHGY01384684	VDR	Western painted turtle	*Chrysemys picta bellii*	Sauropsida	Testudines	Emydidae
ENSACAP00000013307	VDR	Green anole	*Anolis carolinensis*	Lepidosauria	Squamata	Iguanidae
AAP13096	VDR	Tokay gecko	*Gekko gecko*	Lepidosauria	Squamata	Gekkonidae
CAC69541	VDR	Rat snake	*Elaphe sp.*	Lepidosauria	Squamata	Colubridae
AFJ52030	VDR	Eastern diamondback rattlesnake	*Crotalus adamanteus*	Lepidosauria	Squamata	Viperidae
AEQU011219268/AEQU010364265/AEQU010201365/ AEQU010387209/AEQU010164362/AEQU010182350/ AEQU011153487	VDR	Burmese python	*Python molurus bivittatus*	Lepidosauria	Squamata	Pythonidae
AAP22715	VDR	Marine toad	*Rhinella marina*	Amphibia	Anura	Bufonidae
AAI69384	VDR	African clawed frog	*Xenopus laevis*	Amphibia	Anura	Pipidae
ENSXETP00000023342	VDR	Western clawed frog	*Xenopus (Silurana) tropicalis*	Amphibia	Anura	Pipidae

ENSLACP00000021740	Vdr	Coelacanth	*Latimeria chalumnae*	Actinistia	Coelacanthiformes	Coelacanthidae
ENSTRUP00000030103	Vdr a	Torafugu	*Takifugu rubripes*	Acanthopterygii	Tetraodontiformes	Tetraodontidae
ENSTNIP00000008258	Vdr a	Green-spotted pufferfish	*Tetraodon nigroviridis*	Acanthopterygii	Tetraodontiformes	Tetraodontidae
BAA95016	Vdr a	Japanese flounder	*Paralichthys olivaceus*	Acanthopterygii	Pleuronectiformes	Paralichthyidae
CAJ13719	Vdr a	European seabass	*Dicentrarchus labrax*	Acanthopterygii	Perciformes	Moronidae
ENSONIP00000011568	Vdr a	Nile tilapia	*Oreochromis niloticus*	Acanthopterygii	Perciformes	Cichlidae
AFNZ01020271	Vdr a	Burton's mouthbrooder	*Haplochromis burtoni*	Acanthopterygii	Perciformes	Cichlidae
ENSGACP00000006291_Ga_VDRa	Vdr a	Three-spined stickleback	*Gasterosteus aculeatus*	Acanthopterygii	Gasterosteiformes	Gasterosteidae
ACH78365	Vdr a	False kelpfish	*Sebastiscus marmoratus*	Acanthopterygii	Scorpaeniformes	Sebastidae
ENSORLP00000001310	Vdr a	Japanese medaka	*Oryzias latipes*	Acanthopterygii	Beloniformes	Adrianichthyidae
ENSXMAP00000004622	Vdr a	Southern platyfish	*Xiphophorus maculatus*	Acanthopterygii	Cyprinodontiformes	Poeciliidae
ENSGMOP00000013607/ATLCOD1As07556	Vdr a	Atlantic cod	*Gadus morhua*	Paracanthopterygii	Gadiformes	Gadidae
AAS99156	Vdr a	Rainbow trout	*Oncorhynchus mykiss*	Protacanthopterygii	Salmoniformes	Salmonidae
AGKD01081028/AGKD01155946/AGKD01000786	Vdr a1	Atlantic salmon	*Salmo salar*	Protacanthopterygii	Salmoniformes	Salmonidae
AGKD01082350/AGKD01029969/AGKD01006953	Vdr a2	Atlantic salmon	*Salmo salar*	Protacanthopterygii	Salmoniformes	Salmonidae
DT284079	Vdr a	Fathead minnow	*Pimephales promelas*	Ostariophysi	Cypriniformes	Cyprinidae
GAHO01057691	Vdr a	Small gill opening goldenline barbel	*Sinocyclocheilus angustiporus*	Ostariophysi	Cypriniformes	
NP_570994	Vdr a	Zebrafish	*Danio rerio*	Ostariophysi	Cypriniformes	Cyprinidae
CAH04518	Vdr a	Common carp	*Cyprinus carpio*	Ostariophysi	Cypriniformes	Cyprinidae
ENSTRUP00000029256	Vdr b	Torafugu	*Takifugu rubripes*	Acanthopterygii	Tetraodontiformes	Tetraodontidae
ENSTNIP00000009831	Vdr b	Green-spotted pufferfish	*Tetraodon nigroviridis*	Acanthopterygii	Tetraodontiformes	Tetraodontidae
BAA95015	Vdr b	Japanese flounder	*Paralichthys olivaceus*	Acanthopterygii	Pleuronectiformes	Paralichthyidae
CBN80914	Vdr b	European seabass	*Dicentrarchus labrax*	Acanthopterygii	Perciformes	Moronidae
ENSONIP00000024393	Vdr b	Nile tilapia	*Oreochromis niloticus*	Acanthopterygii	Perciformes	Cichlidae
AFNZ01006127	Vdr b	Burton's mouthbrooder	*Haplochromis burtoni*	Acanthopterygii	Perciformes	Cichlidae
ENSGACP00000010579	Vdr b	Three-spined stickleback	*Gasterosteus aculeatus*	Acanthopterygii	Gasterosteiformes	Gasterosteidae
ENSORLP00000020536	Vdr b	Japanese medaka	*Oryzias latipes*	Acanthopterygii	Beloniformes	Adrianichthyidae
JW581855	Vdr b	Gulf killifish	*Fundulus grandis*	Acanthopterygii	Cyprinodontiformes	Fundulidae
ENSXMAP00000018757	Vdr b	Southern platyfish	*Xiphophorus maculatus*	Acanthopterygii	Cyprinodontiformes	Poeciliidae
ENSGMOP00000020924	Vdr b	Atlantic cod	*Gadus morhua*	Paracanthopterygii	Gadiformes	Gadidae
FP319900/FP320245/BX076692	Vdr b	Rainbow trout	*Oncorhynchus mykiss*	Protacanthopterygii	Salmoniformes	Salmonidae
EV381228/EV381227	Vdr b	Sockeye salmon	*Oncorhynchus nerka*	Protacanthopterygii	Salmoniformes	Salmonidae
AGKD01003122/AGKD01047283/AGKD01347709/ AGKD01373969	Vdr b1	Atlantic salmon	*Salmo salar*	Protacanthopterygii	Salmoniformes	Salmonidae
AGKD01082349/AGKD01114959/AGKD01075205/ AGKD01101722	Vdr b2	Atlantic salmon	*Salmo salar*	Protacanthopterygii	Salmoniformes	Salmonidae
ENSDARP00000094891	Vdr b	Zebrafish	*Danio rerio*	Ostariophysi	Cypriniformes	Cyprinidae
JT410510	Vdr b	Channel catfish	*Ictalurus punctatus*	Ostariophysi	Siluriformes	Ictaluridae
AHAT01018745	Vdr	Spotted gar	*Lepisosteus oculatus*	Holostei	Semionotiformes	Lepisosteidae
AESE011506577/AESE011521565/AESE012515322/ AESE011665148/AESE012794671/AESE011787247/ AESE012576052	Vdr	Little skate	*Leucoraja erinacea*	Chondrichthyes	Rajiformes	Rajidae
AAVX02037598	Vdr	Ghost shark	*Callorhinchus milii*	Chondrichthyes	Chimaeriformes	Callorhinchidae
AAP05810/ENSPMAP00000000201/AEFG01007820/ AEFG01007821	Vdr 1	Sea lamprey	*Petromyzon marinus*	Hyperoartia	Petromyzontiformes	Petromyzontidae
APJL01026340/APJL01026341/APJL01026343	Vdr 1	Arctic lamprey	*Lethenteron camtschaticum*	Hyperoartia	Petromyzontiformes	Petromyzontidae
ENSPMAP00000004028/AEFG01016492/AEFG01016493/ AEFG01016494/AEFG01041377/ AEFG01045090	Vdr 2	Sea lamprey	*Petromyzon marinus*	Hyperoartia	Petromyzontiformes	Petromyzontidae
APJL01000063/APJL01000064/APJL01000065/ APJL01000066/APJL01000067/ APJL01000068	Vdr 2	Arctic lamprey	*Lethenteron camtschaticum*	Hyperoartia	Petromyzontiformes	Petromyzontidae
AEFG01041169	Vdr 3	Sea lamprey	*Petromyzon marinus*	Hyperoartia	Petromyzontiformes	Petromyzontidae
APJL01004437/APJL01004436	Vdr 3	Arctic lamprey	*Lethenteron camtschaticum*	Hyperoartia	Petromyzontiformes	Petromyzontidae
ENSCINP00000032788	Vdr-like	Vase tunicate	*Ciona intestinalis*	Tunicata	Enterogona	Cionidae
ENSCSAVP00000003227	Vdr-like	Pacific transparent sea squirt	*Ciona savignyi*	Tunicata	Enterogona	Cionidae
XP_002742261	Vdr	Acorn worm	*Saccoglossus kowalevskii*	Hemichordata		Harrimaniidae
XP_001189625	Vdr	Purple sea urchin	*Strongylocentrotus purpuratus*	Echinodermata	Echinozoa	Strongylocentrotiae
AGCV01063841/AGCV01063840	Vdr	Green sea urchin	*Lytechinus variegatus*	Echinodermata	Echinozoa	Toxopneustidae
AKZP01068386/AKZP01068388	Vdr	Bat star	*Patiria miniata*	Echinodermata	Asterozoa	Asterinidae

NR4A1

ENSP00000353427	NR4A 1	Human	*Homo sapiens*	Euarchontoglires	Primates	Hominidae
ENSMUSP00000023779	NR4A 1	Mouse	*Mus musculus*	Euarchontoglires	Rodentia	Muridae
ENSCAFP00000010883	NR4A 1	Dog	*Canis lupus familiaris*	Laurasiatheria	Carnivora	Canidae
ENSECAP00000014416	NR4A 1	Horse	*Equus caballus*	Laurasiatheria	Perissodactyla	Equidae
ENSLAFP00000011144	NR4A 1	African savanna elephant	*Loxodonta africana*	Afrotheria	Proboscidea	Elephantidae
ENSSHAP00000014809	NR4A 1	Tasmanian devil	*Sarcophilus harrisii*	Metatheria	Dasyuromorphia	Dasyuridae
ENSOANP00000001118	NR4A 1	Platypus	*Ornithorhynchus anatinus*	Prototheria	Monotremata	Ornithorhynchida
ENSAPLP00000013937	NR4A 1	Mallard	*Anas platyrhynchos*	Aves	Anseriformes	Anatidae

AGAI01015414	NR4A 1	Budgerigar	*Melopsittacus undulatus*	Aves	Psittaciformes	Psittacidae
AKZB01070170/AKZB01070171	NR4A 1	Medium ground finch	*Geospiza fortis*	Aves	Passeriformes	Fringillidae
ENSTGUP00000017115	NR4A 1	Zebra finch	*Taeniopygia guttata*	Aves	Passeriformes	Estrildidae
AKHW01036048	NR4A 1	American alligator	*Alligator mississippiensis*	Archosauria	Crocodilia	Crocodylidae
AGCU01140408	NR4A 1	Chinese softshell turtle	*Pelodiscus sinensis*	Sauropsida	Testudines	Trionychidae
AHGY01401775	NR4A 1	Western painted turtle	*Chrysemys picta bellii*	Sauropsida	Testudines	Emydidae
ENSACAP00000016537	NR4A 1	Green anole	*Anolis carolinensis*	Lepidosauria	Squamata	Iguanidae
AEQU011269007/AEQU010127134/AEQU010339800/AEQU010291225	NR4A 1	Burmese python	*Python molurus bivittatus*	Lepidosauria	Squamata	Pythonidae
AAH56025	NR4A 1	African clawed frog	*Xenopus laevis*	Amphibia	Anura	Pipidae
ENSXETP00000001266	NR4A 1	Western clawed frog	*Xenopus (Silurana) tropicalis*	Amphibia	Anura	Pipidae
ENSLACP00000021757	Nr4a 1	Coelacanth	*Latimeria chalumnae*	Actinistia	Coelacanthiformes	Coelacanthidae
ENSTRUP00000039650	Nr4a 1a	Torafugu	*Takifugu rubripes*	Acanthopterygii	Tetraodontiformes	Tetraodontidae
ENSTNIP00000015485	Nr4a 1a	Green-spotted pufferfish	*Tetraodon nigroviridis*	Acanthopterygii	Tetraodontiformes	Tetraodontidae
BB996939	Nr4a 1a	Japanese amberjack	*Seriola quinqueradiata*	Acanthopterygii	Perciformes	Carangidae
ENSONIP00000021057	Nr4a 1a	Nile tilapia	*Oreochromis niloticus*	Acanthopterygii	Perciformes	Cichlidae
AFNZ01006427	Nr4a 1a	Burton's mouthbrooder	*Haplochromis burtoni*	Acanthopterygii	Perciformes	Cichlidae
ENSGACP00000014291	Nr4a 1a	Three-spined stickleback	*Gasterosteus aculeatus*	Acanthopterygii	Gasterosteiformes	Gasterosteidae
ENSORLP00000019478	Nr4a 1a	Japanese medaka	*Oryzias latipes*	Acanthopterygii	Beloniformes	Adrianichthyidae
ENSXMAP00000003595	Nr4a 1a	Southern platyfish	*Xiphophorus maculatus*	Acanthopterygii	Cyprinodontiformes	Poeciliidae
ENSGMOP00000007805	Nr4a 1a	Atlantic cod	*Gadus morhua*	Paracanthopterygii	Gadiformes	Gadidae
FP320517	Nr4a 1a	Rainbow trout	*Oncorhynchus mykiss*	Protacanthopterygii	Salmoniformes	Salmonidae
AGKD01062584	Nr4a 1a1	Atlantic salmon	*Salmo salar*	Protacanthopterygii	Salmoniformes	Salmonidae
DT358238	Nr4a 1a	Fathead minnow	*Pimephales promelas*	Ostariophysi	Cypriniformes	Cyprinidae
ENSDARP00000001911	Nr4a 1a	Zebrafish	*Danio rerio*	Ostariophysi	Cypriniformes	Cyprinidae
FD144868	Nr4a 1a	Blue catfish	*Ictalurus furcatus*	Ostariophysi	Siluriformes	Ictaluridae
FD050395/FD336806	Nr4a 1a	Channel catfish	*Ictalurus punctatus*	Ostariophysi	Siluriformes	Ictaluridae
ENSTRUP00000031186	Nr4a 1b	Torafugu	*Takifugu rubripes*	Acanthopterygii	Tetraodontiformes	Tetraodontidae
ENSTNIP00000009884	Nr4a 1b	Green-spotted pufferfish	*Tetraodon nigroviridis*	Acanthopterygii	Tetraodontiformes	Tetraodontidae
AU090782	Nr4a 1b	Japanese flounder	*Paralichthys olivaceus*	Acanthopterygii	Pleuronectiformes	Paralichthyidae
DC608116	Nr4a 1b	Red seabream	*Pagrus major*	Acanthopterygii	Perciformes	Sparidae
ENSONIP00000024239/ENSONIP00000024240	Nr4a 1b	Nile tilapia	*Oreochromis niloticus*	Acanthopterygii	Perciformes	Cichlidae
AFNZ01012750	Nr4a 1b	Burton's mouthbrooder	*Haplochromis burtoni*	Acanthopterygii	Perciformes	Cichlidae
ENSGACP00000000578	Nr4a 1b	Three-spined stickleback	*Gasterosteus aculeatus*	Acanthopterygii	Gasterosteiformes	Gasterosteidae
ENSORLP00000019478	Nr4a 1b	Japanese medaka	*Oryzias latipes*	Acanthopterygii	Beloniformes	Adrianichthyidae
ENSXMAP00000003595	Nr4a 1b	Southern platyfish	*Xiphophorus maculatus*	Acanthopterygii	Cyprinodontiformes	Poeciliidae
ENSGMOP00000016296	Nr4a 1b	Atlantic cod	*Gadus morhua*	Paracanthopterygii	Gadiformes	Gadidae
AGKD01033644	Nr4a 1b1	Atlantic salmon	*Salmo salar*	Protacanthopterygii	Salmoniformes	Salmonidae
AGKD01027979	Nr4a 1b2	Atlantic salmon	*Salmo salar*	Protacanthopterygii	Salmoniformes	Salmonidae
AHAT01018683/AHAT01018682	Nr4a 1	Spotted gar	*Lepisosteus oculatus*	Holostei	Semionotiformes	Lepisosteidae
EW689579	Nr4a 1	Pacific electric ray	*Torpedo californica*	Chondrichthyes	Torpediniformes	Torpedinidae
AESE012136181/AESE012525732/AESE012798627/AESE012935512/AESE011898114/AESE012517441	Nr4a 1	Little skate	*Leucoraja erinacea*	Chondrichthyes	Rajiformes	Rajidae
AAVX01636020/AAVX01119325/AAVX01637673	Nr4a 1	Ghost shark	*Callorhinchus milii*	Chondrichthyes	Chimaeriformes	Callorhinchidae

NR4A2

ENSP00000344479	NR4A 2	Human	*Homo sapiens*	Euarchontoglires	Primates	Hominidae
ENSMUSP00000028166	NR4A 2	Mouse	*Mus musculus*	Euarchontoglires	Rodentia	Muridae
ENSCAFP00000013553	NR4A 2	Dog	*Canis lupus familiaris*	Laurasiatheria	Carnivora	Canidae
ENSECAP00000011967	NR4A 2	Horse	*Equus caballus*	Laurasiatheria	Perissodactyla	Equidae
ENSLAFP00000015516	NR4A 2	African savanna elephant	*Loxodonta africana*	Afrotheria	Proboscidea	Elephantidae
ENSSHAP00000015224	NR4A 2	Tasmanian devil	*Sarcophilus harrisii*	Metatheria	Dasyuromorphia	Dasyuridae
ENSMODP00000005293	NR4A 2	Gray short-tailed opossum	*Monodelphis domestica*	Metatheria	Didelphimorphia	Didelphidae
ENSOANP00000009765	NR4A 2	Platypus	*Ornithorhynchus anatinus*	Prototheria	Monotremata	Ornithorhynchida
ENSGALP00000020451	NR4A 2	Chicken	*Gallus gallus*	Aves	Galliformes	Phasianidae
ENSMGAP00000013564	NR4A 2	Turkey	*Meleagris gallopavo*	Aves	Galliformes	Phasianidae
ENSAPLP00000011861	NR4A 2	Mallard	*Anas platyrhynchos*	Aves	Anseriformes	Anatidae
AGAI01067815	NR4A 2	Budgerigar	*Melopsittacus undulatus*	Aves	Psittaciformes	Psittacidae
AKZB01018663/AKZB01018661	NR4A 2	Medium ground finch	*Geospiza fortis*	Aves	Passeriformes	Fringillidae
ENSTGUP00000012542/ENSTGUP00000012540	NR4A 2	Zebra finch	*Taeniopygia guttata*	Aves	Passeriformes	Estrildidae
AKHW01048586	NR4A 2	American alligator	*Alligator mississippiensis*	Archosauria	Crocodilia	Crocodylidae
AGCU01104285	NR4A 2	Chinese softshell turtle	*Pelodiscus sinensis*	Sauropsida	Testudines	Trionychidae
AHGY01239600	NR4A 2	Western painted turtle	*Chrysemys picta bellii*	Sauropsida	Testudines	Emydidae
ENSACAP00000007843	NR4A 2	Green anole	*Anolis carolinensis*	Lepidosauria	Squamata	Iguanidae
AEQU011269007/AEQU010127134/AEQU010339800/AEQU010291225	NR4A 2	Burmese python	*Python molurus bivittatus*	Lepidosauria	Squamata	Pythonidae
NP_001093678/ENSXETP00000011580	NR4A 2	Western clawed frog	*Xenopus (Silurana) tropicalis*	Amphibia	Anura	Pipidae
ENSLACP00000012047	Nr4a 2	Coelacanth	*Latimeria chalumnae*	Actinistia	Coelacanthiformes	Coelacanthidae

ENSTRUP00000018382	Nr4a 2a	Torafugu	*Takifugu rubripes*	Acanthopterygii	Tetraodontiformes	Tetraodontidae
ENSTNIP00000019583	Nr4a 2a	Green-spotted pufferfish	*Tetraodon nigroviridis*	Acanthopterygii	Tetraodontiformes	Tetraodontidae
ENSONIP00000011287	Nr4a 2a	Nile tilapia	*Oreochromis niloticus*	Acanthopterygii	Perciformes	Cichlidae
ACZ51350/AFNZ01005751	Nr4a 2a	Burton's mouthbrooder	*Haplochromis burtoni*	Acanthopterygii	Perciformes	Cichlidae
ENSGACP00000007725	Nr4a 2a	Three-spined stickleback	*Gasterosteus aculeatus*	Acanthopterygii	Gasterosteiformes	Gasterosteidae
ENSORLP00000020881	Nr4a 2a	Japanese medaka	*Oryzias latipes*	Acanthopterygii	Beloniformes	Adrianichthyidae
ENSXMAP00000011308	Nr4a 2a	Southern platyfish	*Xiphophorus maculatus*	Acanthopterygii	Cyprinodontiformes	Poeciliidae
ENSGMOP00000020670	Nr4a 2a	Atlantic cod	*Gadus morhua*	Paracanthopterygii	Gadiformes	Gadidae
AGKD01109531	Nr4a 2a1	Atlantic salmon	*Salmo salar*	Protacanthopterygii	Salmoniformes	Salmonidae
AGKD01018107	Nr4a 2a2	Atlantic salmon	*Salmo salar*	Protacanthopterygii	Salmoniformes	Salmonidae
FD303027/FD303026	Nr4a 1a	Channel catfish	*Ictalurus punctatus*	Ostariophysi	Siluriformes	Ictaluridae
ENSONIP00000015276	Nr4a 2b	Nile tilapia	*Oreochromis niloticus*	Acanthopterygii	Perciformes	Cichlidae
AFNZ01007421	Nr4a 2b	Burton's mouthbrooder	*Haplochromis burtoni*	Acanthopterygii	Perciformes	Cichlidae
ENSORLP00000000058	Nr4a 2b	Japanese medaka	*Oryzias latipes*	Acanthopterygii	Beloniformes	Adrianichthyidae
ENSXMAP00000008226	Nr4a 2b	Southern platyfish	*Xiphophorus maculatus*	Acanthopterygii	Cyprinodontiformes	Poeciliidae
ENSGMOP00000004000	Nr4a 2b	Atlantic cod	*Gadus morhua*	Paracanthopterygii	Gadiformes	Gadidae
CA357044/BX874121	Nr4a 2b	Rainbow trout	*Oncorhynchus mykiss*	Protacanthopterygii	Salmoniformes	Salmonidae
AGKD01017366	Nr4a 2b	Atlantic salmon	*Salmo salar*	Protacanthopterygii	Salmoniformes	Salmonidae
ABV25607	Nr4a 2b	Goldfish	*Carassius auratus*	Ostariophysi	Cypriniformes	Cyprinidae
ENSDARP00000065384	Nr4a 2b	Zebrafish	*Danio rerio*	Ostariophysi	Cypriniformes	Cyprinidae
AHAT01028886	Nr4a 2	Spotted gar	*Lepisosteus oculatus*	Holostei	Semionotiformes	Lepisosteidae
AESE012502553/AESE012520301	Nr4a 2	Little skate	*Leucoraja erinacea*	Chondrichthyes	Rajiformes	Rajidae

NR4A3

ENSP00000378531	NR4A 3	Human	*Homo sapiens*	Euarchontoglires	Primates	Hominidae
ENSMUSP00000030025	NR4A 3	Mouse	*Mus musculus*	Euarchontoglires	Rodentia	Muridae
ENSBTAP00000002429	NR4A 3	Cow	*Bos taurus*	Laurasiatheria	Ruminantia	Bovidae
ENSCAFP00000003694	NR4A 3	Dog	*Canis lupus familiaris*	Laurasiatheria	Carnivora	Canidae
ENSLAFP00000021940	NR4A 3	African savanna elephant	*Loxodonta africana*	Afrotheria	Proboscidea	Elephantidae
ENSSHAP00000015832	NR4A 3	Tasmanian devil	*Sarcophilus harrisii*	Metatheria	Dasyuromorphia	Dasyuridae
ENSMODP00000015838	NR4A 3	Gray short-tailed opossum	*Monodelphis domestica*	Metatheria	Didelphimorphia	Didelphidae
ENSOANP00000003373	NR4A 3	Platypus	*Ornithorhynchus anatinus*	Prototheria	Monotremata	Ornithorhynchidae
ENSGALP00000022048	NR4A 3	Chicken	*Gallus gallus*	Aves	Galliformes	Phasianidae
ENSMGAP00000007931	NR4A 3	Turkey	*Meleagris gallopavo*	Aves	Galliformes	Phasianidae
ENSAPLP00000011022	NR4A 3	Mallard	*Anas platyrhynchos*	Aves	Anseriformes	Anatidae
AGAI01067383	NR4A 3	Budgerigar	*Melopsittacus undulatus*	Aves	Psittaciformes	Psittacidae
AKZB01060854	NR4A 3	Medium ground finch	*Geospiza fortis*	Aves	Passeriformes	Fringillidae
ENSTGUP00000009130	NR4A 3	Zebra finch	*Taeniopygia guttata*	Aves	Passeriformes	Estrildidae
AKHW01013932	NR4A 3	American alligator	*Alligator mississippiensis*	Archosauria	Crocodilia	Crocodylidae
AGCU01019063/AGCU01019064/AGCU01019065	NR4A 3	Chinese softshell turtle	*Pelodiscus sinensis*	Sauropsida	Testudines	Trionychidae
AHGY01112567/AHGY01112566	NR4A 3	Western painted turtle	*Chrysemys picta bellii*	Sauropsida	Testudines	Emydidae
ENSACAP000000 8845	NR4A 3	Green anole	*Anolis carolinensis*	Lepidosauria	Squamata	Iguanidae
AEQU010308861/AEQU010066210/AEQU010987533/ AEQU010894250/AEQU010852718/AEQU011256081	NR4A 3	Burmese python	*Python molurus bivittatus*	Lepidosauria	Squamata	Pythonidae
ENSXETP00000026170	NR4A 3	Western clawed frog	*Xenopus (Silurana) tropicalis*	Amphibia	Anura	Pipidae
ENSLACP00000005055	Nr4a 3	Coelacanth	*Latimeria chalumnae*	Actinistia	Coelacanthiformes	Coelacanthidae
ENSTRUP00000028376	Nr4a 3	Torafugu	*Takifugu rubripes*	Acanthopterygii	Tetraodontiformes	Tetraodontidae
ENSTNIP00000021548	Nr4a 3	Green-spotted pufferfish	*Tetraodon nigroviridis*	Acanthopterygii	Tetraodontiformes	Tetraodontidae
DC606759	Nr4a 3	Red seabream	*Pagrus major*	Acanthopterygii	Perciformes	Sparidae
GR476111	Nr4a 3	Mandarin fish	*Siniperca chuatsi*	Acanthopterygii	Perciformes	Sinipercidae
FG228041	Nr4a 3	Long-jawed mudsucker	*Gillichthys mirabilis*	Acanthopterygii	Perciformes	Gobiidae
BB997430	Nr4a 3	Japanese amberjack	*Seriola quinqueradiata*	Acanthopterygii	Perciformes	Carangidae
ENSONIP00000007589	Nr4a 3	Nile tilapia	*Oreochromis niloticus*	Acanthopterygii	Perciformes	Cichlidae
CN470555/AFNZ01028643/AFNZ01028644	Nr4a 3	Burton's mouthbrooder	*Haplochromis burtoni*	Acanthopterygii	Perciformes	Cichlidae
ENSGACP00000011928	Nr4a 3	Three-spined stickleback	*Gasterosteus aculeatus*	Acanthopterygii	Gasterosteiformes	Gasterosteidae
ENSORLP00000010966	Nr4a 3	Japanese medaka	*Oryzias latipes*	Acanthopterygii	Beloniformes	Adrianichthyidae
ENSXMAP00000013222	Nr4a 3	Southern platyfish	*Xiphophorus maculatus*	Acanthopterygii	Cyprinodontiformes	Poeciliidae
ENSGMOP00000015497	Nr4a 3	Atlantic cod	*Gadus morhua*	Paracanthopterygii	Gadiformes	Gadidae
AGKD01206918/AGKD01129286/AGKD01244778/ AGKD01243533/AGKD01021221	Nr4a 3	Atlantic salmon	*Salmo salar*	Protacanthopterygii	Salmoniformes	Salmonidae
DT193732/DT193733	Nr4a 3	Fathead minnow	*Pimephales promelas*	Ostariophysi	Cypriniformes	Cyprinidae
ENSDARP00000072712	Nr4a 3	Zebrafish	*Danio rerio*	Ostariophysi	Cypriniformes	Cyprinidae
AHAT01022080/AHAT01022081	Nr4a 3	Spotted gar	*Lepisosteus oculatus*	Holostei	Semionotiformes	Lepisosteidae
ES788719/DV496959	Nr4a 3	Spiny dogfish	*Squalus acanthias*	Chondrichthyes	Squaliformes	Squalidae
AESE011542765/AESE010066944/AESE012545728/ AESE010636233/AESE010171286	Nr4a 3	Little skate	*Leucoraja erinacea*	Chondrichthyes	Rajiformes	Rajidae
AAVX01042683/AAVX01511734/AAVX01462501	Nr4a 3	Ghost shark	*Callorhinchus milii*	Chondrichthyes	Chimaeriformes	Callorhinchidae

AEFG01035220/AEFG01016042/AEFG01016043/ AEFG01052555/ENSPMAP00000005123/ ENSPMAP00000011133	Nr4a	Sea lamprey	*Petromyzon marinus*	Hyperoartia	Petromyzontiformes	Petromyzontidae
APJL01006429	Nr4a	Arctic lamprey	*Lethenteron camtschaticum*	Hyperoartia	Petromyzontiformes	Petromyzontidae
ENSCINP00000030359	Nr4a	Vase tunicate	*Ciona intestinalis*	Tunicata	Enterogona	Cionidae
ENSCSAVP00000008226	Nr4a	Pacific transparent sea squirt	*Ciona savignyi*	Tunicata	Enterogona	Cionidae
XP_002605189	Nr4a	Florida lancelet	*Branchiostoma floridae*	Cephalochordata	Amphioxiformes	Branchiostomidae
XP_002735608	Nr4a	Acorn worm	*Saccoglossus kowalevskii*	Hemichordata		Harrimaniidae
XP_786266	Nr4a	Purple sea urchin	*Strongylocentrotus purpuratus*	Echinodermata	Echinozoa	Strongylocentroti ae
AGCV01018735	Nr4a	Green sea urchin	*Lytechinus variegatus*	Echinodermata	Echinozoa	Toxopneustidae
AKZP01068386/AKZP01068388	Nr4a	Bat star	*Patiria miniata*	Echinodermata	Asterozoa	Asterinidae
