# Supplementary material for: The Lineage-Specific Evolution of Aquaporin Gene Clusters Facilitated Tetrapod Terrestrial Adaptation
Source: PLoS One. 2014 Nov 26;9(11):e113686. doi: 10.1371/journal.pone.0113686 (PMC4245216; doi:10.1371/journal.pone.0113686)
Supplement: Table S4 — Conservation of residues that putatively determine channel transport selectivities of aquaporins and aquaglyceroporins in Deuterostomia. (PDF) [file pone.0113686.s025.pdf]

Table S4: Conservation of residues that putatively determine channel transport selectivities of aquaporins and aquaglyceroporins in Deuterostomia

	AQP4					AQP14					AQP1				
	P1	P2	P3	P4	P5	P1	P2	P3	P4	P5	P1	P2	P3	P4	P5
Eutheria	T	S	A	Y	W	L	S	A	Y	W	T	S	SA	F	W
Metatheria	T	S	A	Y	W	L	S	A	YC	W	T	S	A	F	W
Sauropsida	T	S	A	Y	W	L	S	A	Y	W	T	S	A	F	W
Amphibia	T	S	A	Y	W	M	S	A	Y	W	T	S	A	F	W
Dipnoi											I	S	A	Y	W
Actinistia	T	S	A	Y	W	L	S	A	F	W	T	S	A	Y	W
Teleostei (a/aa)	T	S	A	Y	W	L	S	A	Y	W	RT	S	A	Y	W
Teleostei (b/ab)	T	S	A	Y	W						RSGF	S	A	YF	W
Holostei	T	S	A	Y	W	L	S	A	Y	W	T	S	A	Y	W
Chondrichthyes	T	S	A	Y	W	L	S	A	Y	W	T	S	A	Y	W
Cyclostomata	T	S	A	Y	W	L	S	A	Y	W					
Basal Deuterostomia	TLF	ST	A	Y	WI										

	AQP15					AQP0					AQP2				
	P1	P2	P3	P4	P5	P1	P2	P3	P4	P5	P1	P2	P3	P4	P5
Eutheria						T	S	A	Y	W	T	S	A	F	W
Metatheria						T	S	A	Y	W	T	S	A	F	W
Sauropsida	TI	S	A	F	W	T	S	A	Y	W	T	S	A	F	W
Amphibia						T	S	A	Y	W	T	S	A	F	W
Dipnoi						T	S	A	Y	W	T	S	A	F	W
Actinistia	T	S	A	F	W	T	S	A	Y	W	T	S	SA	FY	W
Teleostei (a/aa)	TMVA	S	A	FY	W	T	S	A	Y	W					
Teleostei (b/ab)						T	S	A	Y	W					
Holostei	T	S	A	F	W	T	S	A	Y	W					
Chondrichthyes	T	S	A	F	W	T	S	A	Y	W					
Cyclostomata						T	S	A	F	W					
Basal Deuterostomia															

	AQP5					AQP5L					AQP6				
	P1	P2	P3	P4	P5	P1	P2	P3	P4	P5	P1	P2	P3	P4	P5
Eutheria	A	S	AS	F	W						MTI	S	A	F	W
Metatheria	TA	S	A	F	W						T	S	A	F	W
Sauropsida	T	S	AS	F	W	T	S	A	F	W	T	S	A	F	W
Amphibia	A	S	A	F	W	V	S	A	F	W	TA	S	A	F	W
Dipnoi															
Actinistia															
Teleostei (a/aa)															
Teleostei (b/ab)															
Holostei															
Chondrichthyes															
Cyclostomata															
Basal Deuterostomia															

	AQP12					AQP11					AQP8				
	P1	P2	P3	P4	P5	P1	P2	P3	P4	P5	P1	P2	P3	P4	P5
Eutheria	ATS	A	T	Y	WC	S	A	H	Y	WC	S	A	A	Y	W
Metatheria	S	A	T	Y	WC	S	A	H	Y	C	S	A	A	Y	W
Sauropsida	S	A	T	Y	WC	SRG	A	H	Y	W	AT	A	A	Y	W
Amphibia	AS	A	T	Y	WC	LM	A	NL	Y	W	ST	A	A	Y	W
Actinistia	S	A	T	Y	W	S	A	H	Y	W	T	A	A	Y	W
Teleostei (a/aa)	STEAG	AS	T	Y	W	S	A	Q	Y	W	SFYTV	A	A	YF	W
Teleostei (b/ab)						GA	A	Q	Y	W	ST	A	A	Y	W
Holostei	S	A	T	Y	W	S	A	C	Y	W	ST	A	A	Y	W
Chondrichthyes	S	A	T	Y	W	T	A	T	Y	W					
Cyclostomata		A	T	Y	G						S	A	A	Y	W
Basal Deuterostomia	W	AG	TG	YF	W						YLGP	STA	A	YFW	W

	AQP16					AQP3					AQP7				
	P1	P2	P3	P4	P5	P1	P2	P3	P4	P5	P1	P2	P3	P4	P5
Eutheria						Y	D	R	P	I	F	D	R	P	V
Metatheria						Y	D	R	P	I	FY	D	R	P	V
Sauropsida	VM	A	A	Y	W	Y	D	R	P	IV	Y	D	R	P	LVI
Amphibia	A	A	A	Y	W	Y	D	R	P	I	Y	D	R	P	I
Actinistia						Y	D	R	P	I	Y	D	R	P	I
Teleostei (a/aa)						Y	D	R	P	LVIT	Y	D	R	P	LVI
Teleostei (b/ab)						Y	D	R	P	I					
Holostei						Y	D	R	P	I					
Chondrichthyes						Y	D	R	P	LVI					
Cyclostomata						Y	D	R	P	V					
Basal Deuterostomia						Y	D	R	P	TIL					

	AQP9					AQP10					AQP13				
	P1	P2	P3	P4	P5	P1	P2	P3	P4	P5	P1	P2	P3	P4	P5
Eutheria	Y	D	R	P	VI	Y	D	R	P	V					
Metatheria	Y	D	R	P	VT	Y	D	R	P	V					
Sauropsida	NY	D	R	P	VTI	Y	D	R	P	LVI					
Amphibia	Y	D	R	P	V	Y	D	R	P	V	Y	D	R	P	V
Actinistia	Y	D	R	P	V	Y	D	R	P	VI					
Teleostei (a/aa)	Y	D	R	P	V	Y	D	R	P	LVIM					
Teleostei (b/ab)	Y	D	R	P	V	Y	D	R	P	LI					
Holostei	Y	D	R	P	V	Y	D	R	P	LV					
Chondrichthyes	Y	D	R	P	V	Y	D	R	P	LVI					
Cyclostomata						Y	D	R	P	VI					
Basal Deuterostomia															

Residues P1 - P5 are identified after Froger et al. (1998) following alignment of orthologs to
human AQP4 (ENSP00000311165): P1 = T137; P2 = S217; P3 = A221; P4 = Y233; P5 = W234
human AQP8 (ENSP00000219660): P1 = S132; P2 = A214; P3 = A218; P4 = Y230; P5 = W231
human AQP11 (ENSP00000318770): P1 = S140; P2 = A220; P3 = H224; P4 = Y239; P5 = W240
human AQP10 (ENSP00000318355): P1 = Y119; P2 = D218; P3 = R222; P4 = P245; P5 = V246
