# Supplementary material for: The Lineage-Specific Evolution of Aquaporin Gene Clusters Facilitated Tetrapod Terrestrial Adaptation
Source: PLoS One. 2014 Nov 26;9(11):e113686. doi: 10.1371/journal.pone.0113686 (PMC4245216; doi:10.1371/journal.pone.0113686)
Supplement: Table S5 — Conservation of AQP2-S256 (P1), S261 (P2) in Sarcopterygii. (PDF) [file pone.0113686.s026.pdf]

Table S5: Conservation of AQP2-S256 (P1), S261 (P2) in Sarcopterygii

	AQP0		AQP2		AQP5		AQP5L		AQP6[a]		AQP6vs1/2	
	P1	P2	P1	P2	P1	P2	P1	P2	P1	P2	P1	P2
Eutheria	P	T	S	S	T	A-T			S-P	G-E		
Metatheria	P	T	S	S	T	C			P	G		
Aves	P	T	S	S	S	P						
Crocodylia	P	T	S	S	S	S						
Testudines	P	T	S	S	S	S			P	N		
Squamata	P	T	S	S	S	S	S-T	S-H	S-A	S		
Amphibia	P	T	S	S	S	S	S	D	S	T	S	S
Dipnoi	P	T	T	S								
Actinistia	P	T	S	S								

[a] Indicates amphibian AQP6ub
