# Supplementary material for: The Lineage-Specific Evolution of Aquaporin Gene Clusters Facilitated Tetrapod Terrestrial Adaptation
Source: PLoS One. 2014 Nov 26;9(11):e113686. doi: 10.1371/journal.pone.0113686 (PMC4245216; doi:10.1371/journal.pone.0113686)
Supplement: Text S1 — Additional text concerning the unexpected diversity of prokaryotic and basal metazoan aquaporins, the broader repertoire of aquaglyceroporins and the ubiquity of unorthodox aquaporins in Vertebrata, and the observation of eight Aqp8s in tetraploid teleostei vs one and a novel subfamily in diploid Tetrapoda. (DOC) [file pone.0113686.s027.doc]

Supplementary text S1

The lineage-specific evolution of aquaporin gene clusters facilitated tetrapod terrestrial radiation

**Roderick Nigel Finn** ^1,2*^, **François Chauvigné** ^1,3^, **Jón Baldur Hlidberg** ^4^, **Christopher P Cutler** ^5,6^, **Joan Cerdà** ^3*^

##### Unexpected Diversity of Prokaryotic and Basal Metazoan Aquaporins

Bayesian analyses of 133 archaeal and bacterial sequences provided robust statistical support for four major clades of prokaryotic aquaporin, which we term AqpZ, AqpN, AqpM and GlpF (Figure S5). Archaeal and bacterial gene products are represented within each of these four clades, with some bacterial members (Firmicutes) encoding six paralogs (five GlpF and an AqpZ).

The data for Porifera and Cnidaria also revealed expansion of the aquaporin superfamily within these Phyla, with Cnidaria encoding up to four *aqp4*-like, an *aqp12*-like unorthodox aquaporin and three *glps*, while an *aqp8*-like gene and multiple *glps* were found in Porifera (Figure S3). A fourth set of homologs (*aqpH1-5*), that could potentially represent cnidarian *aqp8*-like orthologs was identified in stony corals (Anthozoa), and an additional possible *aqp8*-like gene noted in the genome of the starlet sea anemone (*Nematostella vectensis*). To determine whether the multiplicity of these parazoan-metazoan aquaporins could explain the observed diversity of the basal deuterostome orthologs, we combined and realigned the two data sets and examined their interrelationships using Bayesian protocols. These separate analyses recovered the original topologies of the different phyla without intermixing of the parazoan, cnidarian or basal deuterostome subclusters (Figure S4). However, the anomalous clustering of the *aqpH1-5* orthologs between the unorthodox aquaporins and *glps*, prompted us to search for potential matches within Alveolata WGS and TSAs. Blast hits of up to 96% identity were found within TSAs for *Symbiodinium* species, and we suggest that the *aqpH1-5* orthologs are likely derived from zooanthellate endosymbiotic dinoflagellates. Similarly, the divergent starlet sea anemone sequence (EDO28361) encoded in a single exon is more closely related (80% amino acid identity) to bacterial (*Pedobacter sp*.) AqpN than echinoderm *aqp8L1* or *-8L2* (21 - 24% amino acid identity), while the extra *glp2a-c* sequences found in the genome of the sea sponge (*Amphimedon queenslandica*) clustered with bacterial (*Candidatus entotheonella sp*.) *glpF*, indicating that these orthologs may also be derived from endosymbionts. Further searches for *aqp8*-like orthologs in Cnidaria identified a putative sequence (FP978959) in the thecate hydroid *Clytia hemisphaerica*, however, this ortholog is also found in different metazoan phyla, including Hexapoda, Mollusca and Annelida, and is almost identical (99% amino acid identity) to plant plasma membrane intrinsic proteins (PIP), suggesting that it is not a wild-type cnidarian aquaporin. We did, however identify multiple *aqp8*-like orthologs in the genomes of several metazoan phyla, including Annelida, Nematoda, and Mollusca (see Supplementary Table S1, Supplementary Material online for a full listing of accession numbers). Taken together, these analyses revealed independent duplication of aquaporins in parazoan, cnidarian, protostome and basal deuterostome animals, and that the expansion of the superfamily into four major grades occurred prior to the evolution of Bilateria.

##### A Broader Repertoire of Aquaglyceroporins in Vertebrata

All gnathostome orthologs (N = 824) in the initial deuterostome data set clustered as polyphyletic submembers of the four major grades of water channel (Figure 1, main text). In each subfamily, the branching topology was highly congruent with speciation events, which due to the broad sampling strategy, could be discerned at the levels of clade and order. The most widely sampled forms of aquaglyceroporin were *aqp3*, *-7*, *-9* and *-10*, with *aqp3* on a sister branch to *aqp9*, and *aqp7* clustering with *aqp10* (Figure S6). In each subclass, the sarcopterygian aquaglyceroporins were robustly separated from the actinopterygian counterparts, with single orthologs retrieved from the genomes of most sarcopterygian lineages. Some exceptions were noted however. For example, the green anole encodes two divergent forms of *AQP7* (69% amino acid identity), chiropteran bats retain multiple copies of *AQP9* (68 - 72% amino acid identity) encoded by 1 - 6 exons, and humans evolved redundant *AQP7* genes with four pseudogenes (*AQP7p1* *-7p4*) encoded between the 42 - 70 Mb loci on LG9. Surprisingly, only single orthologs of *aqp7* were found for Teleostei, including the tetraploid salmonids, and to date, none in the genome of the holostean spotted gar, a representative of a more ancient lineage of actinopterygian fish that diverged prior to the fish-specific genome duplication event (R3 WGD) at the root of the crown-clade of Teleostei [1]. In contrast we found two tandemly arranged *aqp10* paralogs in the spotted gar genome (6 Mb locus; LG24), one of which is a full-length transcript (encoding 307 amino acids) that clustered basal to teleost *aqp10b* orthologs, and the other, a partial transcript encoding 144 amino acids of the C-terminal hemipore, that clustered basal to teleost *aqp10a* orthologs. Interestingly, in some teleost orders, two tandemly arranged *aqp10aa*, *-10ab* paralogs in addition to a third *aqp10b* paralog were found encoded in the genomes of Tetraodontiformes (7.9 Mb locus, LG8: green-spotted pufferfish) and Cyprinodontiformes (0.3 Mb locus, Scaffold JH556909: Southern platyfish, *Xiphophorus maculatus*; and contig AYCK01006466: Amazon molly, *Poecilia formosa*), while the third *aqp10b* paralog is encoded on separate LGs. Tandem replicates were also found for teleost *aqp3a* channels such that certain families (Cichlidae) in the order Perciformes (e.g. 1.2 - 1.3 Mb locus, Scaffold GL831329: Nile tilapia, *Oreochromis niloticus*) as well as members of the order Scorpaeniformes (sablefish, *Anoplopoma fimbria*) retain closely linked *aqp3aa*, and *-3ab* paralogs. In the latter case, the sablefish also encodes the *aqp3b* paralog, while we have not yet identified the *aqp3b* in Cichlidae.

Amongst more ancestral phyla, two tandemly arranged *aqp3* paralogs (*aqp3* and *-3L*, e.g. contig 16279 of the ghost shark), as well as single forms of *aqp9* and *-10*, but not *aqp7* were identified in Chondrichthyes. In Cyclostomata, five aquaglyceroporin orthologs were identified: four in sea lamprey, and one isolated from the esophagus of the Atlantic hagfish. The two sequenced sea lamprey paralogs isolated from the eyes clustered as *aqp3*-like channels (*aqp3L1*, *-3L2*), while the other two paralogs assembled from the genome clustered as *aqp10*-like channels (*aqp10L1*, *-10L2*). Subsequent assembly of the aquaporin superfamily encoded in the recently released Arctic lamprey (*Lethenteron camtschaticum*) genome confirmed these results, but further revealed that the full-length *aqp10*-like transcripts (82% nucleotide and 75% deduced amino acid identity) are tandemly arranged on contig 000280, while the full-length *aqp3*-like transcripts (72% nucleotide and 74% deduced amino acid identity) are respectively encoded on separate contigs (108287 and 024229).

The novel full-length transcript isolated from the esophagus of the Atlantic hagfish (271 amino acids) clustered with a fifth type of aquaglyceroporin first identified in African clawed frog (*Xenopus laevis*) oocytes (AQPxlo; [2]; also termed AQPa1, [3]) and that we have previously reported to be present in Prototheria (Finn and Cerdà 2011). Subsequent genomic searches confirmed the presence of this ortholog in the genomes of Anura, Caudata and Prototheria, but not in Metatheria, Eutheria or Sauropsida. Comparison of the loci in the platypus (*Ornithorhynchus anatinus*) and Western clawed frog indicated that the genes are syntenic (Figure S7). Since we show that the genomes of Amphibia and Prototheria also encode the canonical *AQP3*, *-7*, *-9* and *-10* orthologs, we named this fifth class of aquaglyceroporin AQP13. The co-clustering of the Atlantic hagfish sequence with *AQP13* suggested that the hagfish channel could be an ancestral form of this subfamily. However, the low level of structural conservation between the hagfish and amphibian forms (50% amino acid and 53% nucleotide identity), and the absence of the *AQP13* ortholog in non-amphibian/prototherian genomes, suggests that long-branch attraction might underlie the topology. We are therefore uncertain of the orthology of the hagfish aquaglyceroporin, so annotated it as *glp*. The above analyses thus revealed that at least five aquaglyceroporin paralogs are found in Cyclostomata, four in Chondrichthyes, up to five in Amphibia and Prototheria, four in Metatheria and most eutherian lineages, five in some sauropsids, four in Actinistia, four in holostean fishes and potentially up to nine paralogs in some diploid Teleostei. The retention of a such broad repertoire of aquaglyceroporins over more than 500 million years of evolution is clearly indicative of positive selection. By contrast, negative selection may only have occurred recurrently for *aqp7* orthologs, which may be lost in chondrichthyan and holostean fishes as well the duplicated teleost paralogs that should have arisen from WGD.

##### Unorthodox Aquaporins are Ubiquitous in Gnathostomata

Genomic searches and Bayesian analyses of unorthodox aquaporins identified single genes in the majority of lineages examined, except two tandemly arranged *aqp11* paralogs in spotted gar (9.9 Mb locus, LG3), two previously identified genomic duplicates (*aqp11a*, *-11b*) in Teleostei [4,5], and two tandemly linked *AQP12* paralogs in gorillas and humans (e.g. *AQP12A*, *-12B*; 130 Mb locus, LG2b: Western lowland gorilla, *Gorilla gorilla*). Short *aqp12*-like fragments were identified in the Arctic lamprey genome, which, when concatenated and submitted to Bayesian inference, clustered between the gnathostome unorthodox channels and the basal deuterostome *aqp12*-like orthologs (Figure S8). The arctic lamprey fragments could not be found in the sea lamprey genome, but full-length transcripts were assembled from distantly related chondrichthyan fishes, which cluster with the two species of actinistian coelacanth studied. Amongst Actinopterygii, single *aqp12* orthologs were identified in the holostean spotted gar and Teleostei. In contrast to the broader phylogenetic distribution of the unorthodox *aqp12* channels, *aqp11* orthologs were only found in gnathostome genomes, including a single chondrichthyan transcript (little skate), which also clustered with the actinistian orthologs below the included spotted gar ortholog and the duplicated teleost forms. These analyses thus revealed that the unorthodox aquaporins, *aqp11* and *-12*, are encoded in the genomes of every extant clade of gnathostome vertebrate, while *aqp12* is also found in more ancestral lineages.

##### Eight Aqp8s in Tetraploid Teleostei vs One and a Novel Subfamily in Diploid Tetrapoda

Assembly of the *aqp8* sequences revealed that the split gene structures are conserved between Echinodermata and Vertebrata with 5-6 exons. The molecular phylogenetic analyses revealed a topology that is highly congruous with speciation events, and thus vertical transfer of *aqp8* genes (Figure S9). However, although we show that basal Deuterostomia have multiple forms of *aqp8*, we only found evidence for single *aqp8* genes in Cyclostomata and Sarcopterygii, and to date, none in Chondrichthyes. In contrast, we have previously shown that Teleostei encode three *aqp8* paralogs: *aqp8aa*, *-8ab* and *-8b* [4-7]. In the present context, we assembled the genomic repertoire of aquaporins in the protacanthopterygian Atlantic salmon (*Salmo salar*), and found that this tetraploid organism encodes the highest copy number of aquaporins of any vertebrate studied to date with complete or partial fragments of at least 42 paralogs. We further verified the existance of 35 paralogs in the first draft of the rainbow trout genome. Amongst this broad diversity, we show that eight of the Atlantic salmon paralogs cluster as *aqp8*-type water channels. We further found that the genome of the spotted gar encodes two tandemly arranged *aqp8aa* and *-8ab* paralogs at the 10.2 Mb locus on LG13. These latter tandem replicates clustered below dual sets of the teleost orthologs revealing a fourth group of orthologs (*aqp8ba*) in the older teleost lineages. To validate these observations, we re-examined the syntenic relationships of the teleost *aqp8* genes in relation to the spotted gar loci (Figure S10). These new data confirmed the syntenic arrangement of the tandemly replicated *aqp8aa* and *-8ab* paralogs in the spotted gar, the ostariophysan zebrafish and members of the Acanthomorpha as well as a dual set of twin tandem duplicates (*aqp8aa1*, *-8ab1*, and *aqp8aa2*, *-8ab2*) in the protacanthopterygian Atlantic salmon (Figure S10A). The data further revealed related paralogons harbouring four extra paralogs in the Atlantic salmon (full-length *aqp8bb1*, and *-8bb2*, and fragments of *-8ba1* and *-8ba2*) that are partially conserved as twin tandem duplicates in the ostariophysan Mexican tetra (*Astyanax mexicanus*) or single paralogs in some lineages of Acanthomorpha (Figure S10B). Only the zebrafish genome currently shows an exceptional arrangement with two near identical duplicates resulting from an intrachromosomal segmental duplication (*aqp8bb1*, *-8bb2*: 100% amino acid identity, except for a 21 amino acid extended N-terminus at the 18.3 - 18.4 Mb locus, LG3). By integrating the phylogenetic results with the observed syntenic relationships, it became apparent that tandem replication of actinopterygian *aqp8* genes occurred prior to the fish-specific WGD (R3) at the base of the crown clade of Teleostei, such that the actinopterygian *aqp8aa* and *-8ab* paralogs already existed prior to the evolution of holstean fishes, and continued to evolve via vertical transfer, while two novel paralogs arose during the R3 WGD event. One of these novel R3 products has been named *aqp8b* in previous works [4-8], however, since we now show that it likely evolved in conjunction with the *aqp8ba* paralog, a more congruous nomenclature is *aqp8bb* for the *aqp8b* tandem duplicate found in Teleostei.

Amongst amphibian *AQP8*-like orthologs, we noted that an African clawed frog sequence (NP_001089643) clustered together with the cyclostome transcripts, rather than the congeneric *AQP8*-like sequence identified in the Western clawed frog. Considering the theory that serial rounds of WGD occurred during early chordate evolution [9-12], and the present evidence that only single *aqp8* orthologs exist in sarcopterygian lineages, we hypothesised that the divergent African clawed frog sequence might represent a genomic duplicate that is lost in other vertebrates. To investigate this possibility, we searched for related orthologs and found that the Western clawed frog has both a canonical *AQP8* gene and the novel African clawed frog *AQP8*-like ortholog. We subsequently identified this latter gene in turtles (Testudines) and alligators (Crocodylia), which also retain canonical *AQP8* genes. Based upon the ancestral topological position of the novel *AQP8*-like transcripts, and the observation that they are encoded in addition to canonical *AQP8* paralogs in the genomes of diverse orders of tetrapod, including Anura, Testudines and Crocodylia, it is evident that they form a hitherto unknown subclass of aquaporins. We annotated members of this novel subclass *AQP16* as putative products of the second round (R2) of chordate WGD.

###### Supplementary References

1. Amores A, Catchen J, Ferrara A, Fontenot Q, Postlethwait JH (2011) Genome evolution and meiotic maps by massively parallel DNA sequencing: Spotted gar, an outgroup for the teleost genome duplication. Genetics 188: 799-U79.

2. Virkki LV, Cooper GJ, Boron WF (2001) Cloning and functional expression of an MIP (AQP0) homolog from killifish (*Fundulus heteroclitus*) lens. Am J Physiol-reg I 281: R1994-R2003.

3. Suzuki M, Tanaka S (2009) Molecular and cellular regulation of water homeostasis in anuran amphibians by aquaporins. Comp Biochem Phys 153A: 231-241.

4. Cerdà J, Finn RN (2010) Piscine Aquaporins: An Overview of Recent Advances. J Exp Zool 313A: 623-650.

5. Tingaud-Sequeira A, Calusinska M, Chauvigné F, Lozano J, Finn RN, Cerdà J (2010) The zebrafish genome encodes the largest vertebrate repertoire of functional aquaporins with dual parology and substrate specificities similar to tetrapods. BMC Evol Biol 10: 38.

6. Finn RN, Cerdà J (2011) Aquaporin evolution in fishes. Front Physiol 2: 44.

7. Engelund MB, Chauvigne F, Christensen BM, Finn RN, Cerda J, Madsen SS (2013) Differential expression and novel permeability properties of three aquaporin 8 paralogs from seawater-challenged Atlantic salmon smolts. J Exp Biol 216: 3873-3885.

8. Chauvigne F, Boj M, Vilella S, Finn RN, Cerda J (2013) Subcellular Localization of Selectively Permeable Aquaporins in the Male Germ Line of a Marine Teleost Reveals Spatial Redistribution in Activated Spermatozoa. Biol Reprod 89:

9. Meyer A, Schartl M (1999) Gene and genome duplications in vertebrates: the one-to-four (-to-eight in fish) rule and the evolution of novel gene functions. Curr Opin Cell Biol 11: 699-704.

10. Robinson-Rechavi M, Boussau B, Laudet V (2004) Phylogenetic dating and characterization of gene duplications in vertebrates: The cartilaginous fish reference. Mol Biol Evol 21: 580-586.

11. Dehal P, Boore JL (2005) Two rounds of whole genome duplication in the ancestral vertebrate. PLoS Biol 3: 1700-1708.

12. Kuraku S, Meyer A, Kuratani S (2009) Timing of genome duplications relative to the origin of the vertebrates: Did Cyclostomes diverge before or after? Mol Biol Evol 26: 47-59.
